# Supplementary material for: Integrating artificial intelligence and optogenetics for Parkinson’s disease diagnosis and therapeutics in male mice
Source: Nat Commun. 2025 Aug 21;16:7797. doi: 10.1038/s41467-025-63025-w (PMC12370958; doi:10.1038/s41467-025-63025-w)
Supplement: Supplementary file 1 — Supplementary Information [file 41467_2025_63025_MOESM1_ESM.pdf]

Supplementary Information

Integrating artificial intelligence and optogenetics for Parkinson's disease diagnosis and therapeutics in male mice

Bobae Hyeon[1,2,3], Jaehyun Shin[4#], Jae-Hun Lee[5#], Woori Kim[2,3], Jea Kwon[6], Heeyoung Lee[1], Dae-gun Kim[7,8], Choong Yeon Kim[9,10], Sang Kun Choi[1], Jae-Woong Jeong[9,11,12], Kwang-Soo Kim[2,3], C. Justin Lee[5*], Daesoo Kim[7*] and Won Do Heo[1,7,13*]

[#] These authors contributed equally
[*] Corresponding authors

C. Justin Lee: cjl@ibs.re.kr
Daesoo Kim: daesoo@kaist.ac.kr
Won Do Heo: wondo@kaist.ac.kr

This PDF includes the following;

high-resolution Supplementary Figure file is available via Figshare (see Data Availability).

Supplementary Figures (45)

Supplementary Tables (22)

Supplementary Notes (8)

Supplementary References

Supplementary Figures

List of Supplementary Figures:

1. Unilateral hA53T PD mouse model failed to show dose-dependent PD severity.
2. Immunohistochemistry (IHC) images of the CT and PD groups.
3. Bilateral hA53T PD mouse model exhibited diverse phenotypes.
4. Comparison temporal variabilities with t-SNE datasets.
5. Description of features engineered to develop AI models for PD diagnosis in mice.
6. Development and comparison of AI models for PD diagnosis.
7. Comparison of cross-view (CV) and cross-subject (CS) validation methods.
8. Comparison of APS, RRS, and BWS for early PD detection at 2 wk.
9. Validation of APS for PD specificity using an amyotrophic lateral sclerosis (ALS) mouse model.
10. Detailed plots of correlation analysis with varied dataset filtering at group level.
11. Summary of correlation analysis.
12. Comparison train-validation (TV) and unseen datasets for top 20 feature importances.
13. Comparison of body lengths across non-PD (NP) and PD groups.
14. CT group's longitudinal analysis of the top 20 features (XGB model) in the KDE plots.
15. PD (A5) group's longitudinal analysis of the top 20 features (XGB model) in the KDE plots.
16. Longitudinal comparison of KL divergences (XGB model) between the CT and PD (A5) groups.
17. Longitudinal comparison of feature values (XGB model) between the CT and PD (A5) groups.
18. NALS group's longitudinal analysis of the top 20 features (XGB model) in the KDE plots.
19. ALS group's longitudinal analysis of the top 20 features (XGB model) in the KDE plots.
20. Longitudinal comparison of KL divergences (XGB model) between the NALS and ALS groups.
21. Longitudinal comparison of feature values (XGB model) between the NALS and ALS groups.
22. Endpoint comparison of PD and ALS groups for the top 20 features identified by the XGB model.
23. SHAP dependence plots of the top 20 features (XGB model).
24. Summary table of SHAP dependence plots for the top 20 features (XGB model).
25. Behavioural assessments (RRS, BWS, and APS) in PD mice treated with optogenetic stimulation (optoRET).
26. Behavioural assessments (RRS, BWS, and APS) in A1 PD mice treated with L-DOPA or RET agonist (BT44).
27. Group comparison of the decision tree plots (XGB model) at 10 wk.
28. Group comparison of the top 20 features (XGB model) in the KDE plots at 10 wk.
29. Comparison of KL divergences of the top 20 features (XGB model) between groups at 10 wk.
30. Group comparisons of the feature values (XGB model) at 10 wk.
31. Comparisons of treatment response and PD symptomatic evaluations in A1 PD mice with different treatments.
32. Summary table of the features engineered and extracted for the spectro-temporal analysis.
33. Model evaluation and SHAP dependence plots of top 20 features (TSFEL model).
34. CT group's longitudinal analysis of the top 20 features (TSFEL model) in the KDE plots.
35. PD (A5) group's longitudinal analysis of the top 20 features (TSFEL model) in the KDE plots.
36. Longitudinal comparison of KL divergences (TSFEL model) between the CT and PD (A5) groups.
37. Longitudinal comparison of feature values (TSFEL model) between the CT and PD (A5) groups.
38. Group comparison of the KDE plots of the top 20 features (TSFEL model) at 10 wk.
39. Group comparison of the top 20 features (TSFEL model) at 10 wk.
40. Group comparison of the turning features at 10 wk.
41. Group comparison of the rearing features at 10 wk.
42. Model evaluation and SHAP dependence plots of top 20 features (Gait model).
43. Group comparison of the top 20 features (Gait model) in KDE plots at 10 wk.
44. Group comparison of the top 20 features (Gait model) in bar plots at 10 wk.
45. Snapshot video images of CT and PD mice in walking.

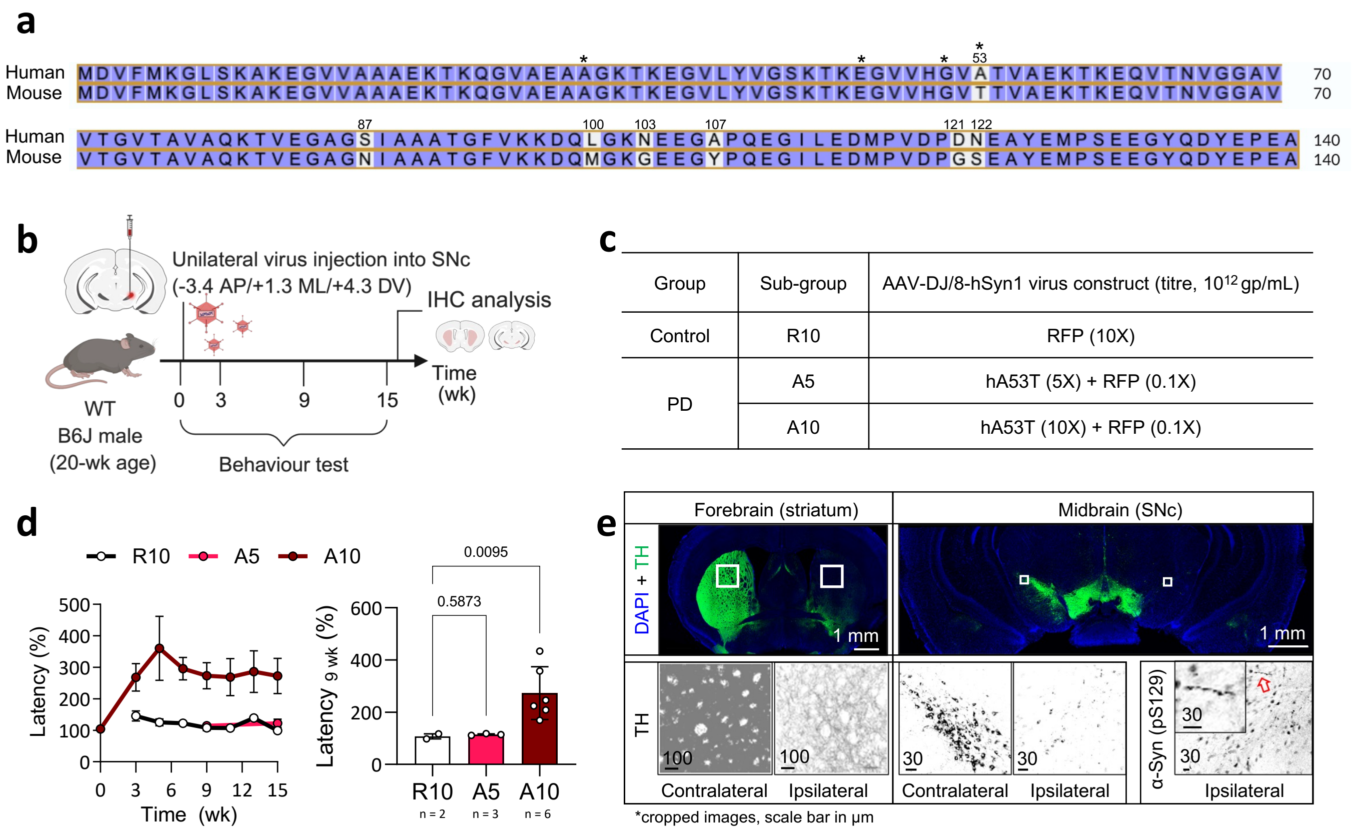

Supplementary Figure 1. Unilateral hA53T PD mouse model failed to show dose-dependent PD severity.

(a) Sequence alignment of human (UniProt ID: P37840) and mouse (UniProt ID: O55042) alpha-synuclein (aSyn) amino acid sequences, highlighting seven mismatches (A53T, S87N, L100M, N103G, A107Y, D121G, and N122S). Positions of familial PD-associated substitutions (A30P, E46K, G51D, A53E, and A53T) are indicated by asterisks (*). Notably, substitutions in the C-terminal region of mouse aSyn (G121 and S122) effectively dampen membrane-induced aSyn aggregation and vesicle permeabilisation, protecting rodents from the deleterious effects exerted by the A53T mutation in humans. **(b)** Schematic illustration of the experimental setup used to characterise the unilateral hA53T PD mouse model. **(c)** Table summarising three experimental groups injected with RFP or different doses of hA53T virus. **(d)** Line and bar graphs showing traverse latency in the elevated-beam walking test (BWT), analysed longitudinally and at the midpoint (9 wk), respectively (R10 = 107.4% ± 9.38, A5 = 114.5% ± 2.17, A10 = 273.0% ± 41.26; traverse time normalised by mean baseline of A10). **(e)** Representative TH-stained immunohistochemistry (IHC) images of forebrain and midbrain sections, highlighting the striatum and SNc following unilateral injection of hA53T at the 5X dose (A5 group). An additional SNc image stained for phospho-aSyn (pS129) reveals aSyn aggregates and Lewy-like fibrils (indicated by a red arrow). Data are shown as mean ± SEM; sample sizes (n) are indicated in plots. Schematic was created in Schematics were created in BioRender. Heo, W. (2025) https://BioRender.com/cykyauz. Statistical analysis was performed using Brown–Forsythe and Welch one-way ANOVA tests followed by Dunnett's T3 post hoc corrections for multiple comparisons. Source data are provided in the Source Data file.

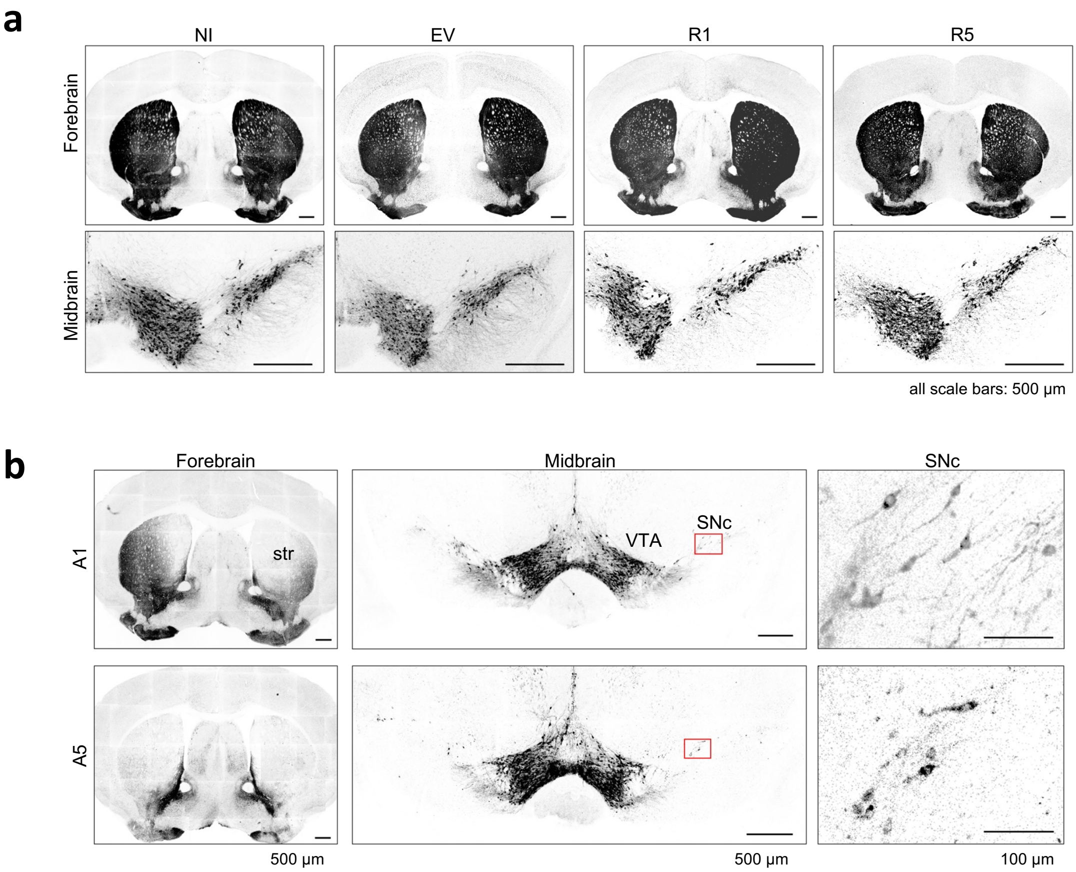

Supplementary Figure 2. Immunohistochemistry (IHC) images of the CT and PD groups.

(a) Representative TH-stained IHC images of the forebrain and midbrain from CT subgroups. **(b)** Representative TH-stained IHC images of the forebrain and midbrain from PD subgroups. Red boxes indicate the SNc regions shown in the enlarged images on the right. Abbreviations: CT, control; NI, no virus injection; EV, empty vector; R1, RFP (1X); R5, RFP (5X); A1, hA53T (1X); A5, hA53T (5X); str, striatum; SNc, substantia nigra pars compacta; VTA, ventral tegmental area; TH, tyrosine hydroxylase.

a

Observed phenotype	Behaviour assessment	Observation in A5	Examples
Hyperactivity	OFT	61% (11/18)	CT 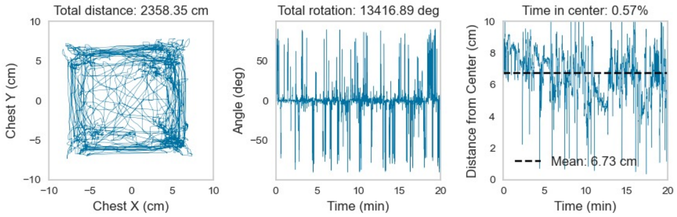
Directional movement (rotation)	OFT	56% (10/18)	A1 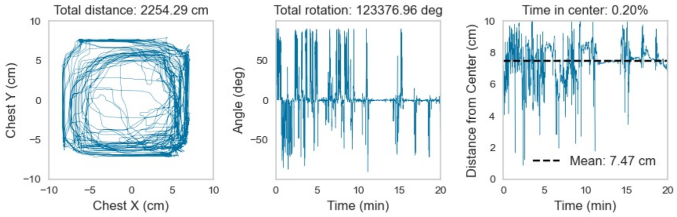
Thigmotaxis	OFT	67% (12/18)	A5 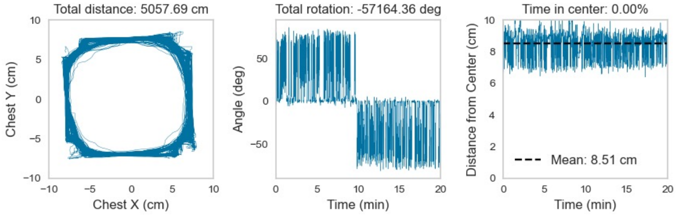
Tremor (hands)	OFT	100% (16/16)	Supplementary_Movie_01_Tremor
Increased wall-touching in rearing	OFT	94% (15/16)	Supplementary_Movie_02_Rearing
Lagging/trailing feet in walking	OFT	63% (10/16)	Supplementary_Movie_03_Trailing
Distracted (stop and look downward)	BWT	87% (13/15)	Distracted / Sandwiching / Collapsed 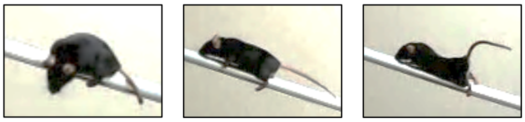
Collapsed (plantar on the beam over 50% of total testing distance)	BWT	67% (10/15)	
Freezing or dragging feet on the beam	BWT	87% (13/15)	
Thighs sandwiching the beam	BWT	80% (12/15)	Supplementary_Movie_04_EBWT
Hindlimb clasping	TST	88% (7/8)	Class 0 / Class 1 / Class 2 / Class 3 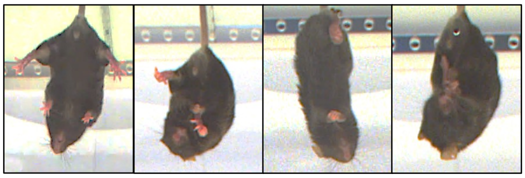

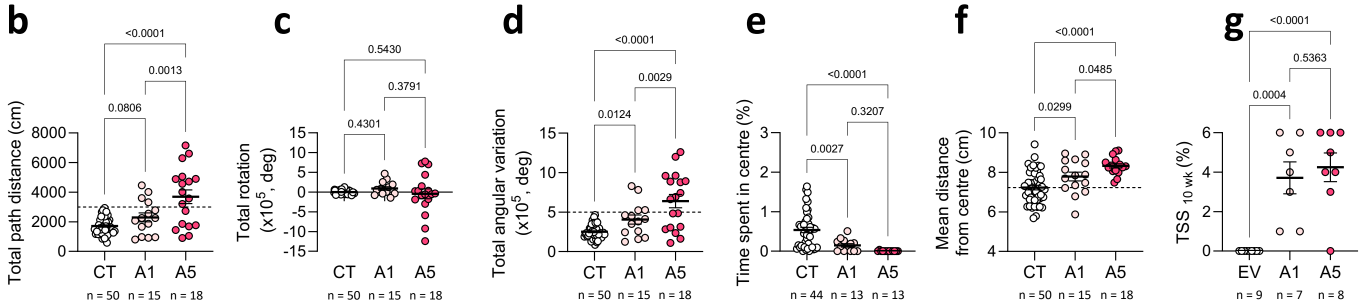

Supplementary Figure 3. Bilateral hA53T PD mouse model exhibited diverse phenotypes.

(a-g) Altered locomotion, motor coordination, tremor, and dystonia observed during the open-field test (OFT), elevated-beam walking test (BWT), and tail suspension test (TST). In panel a, percentages represent the proportion of mice in the A5 group exhibiting each behavioural phenotype, determined by defined thresholds. **(b-f)** Metrics of body centre position measured in the OFT, assessing altered locomotion. Dashed horizontal lines in panels (b, d, f) indicate the thresholds for hyperactivity, rotation, and thigmotaxis in PD, respectively. **(g)** Assessment of hindlimb clasping measured as TST score (TSS, see Methods). Abbreviations: Control (CT), EV (non-PD), A1 (mild) and A5 (severe) PD groups. Data are shown as mean ± SEM; sample sizes (n) are indicated in plots. All statistical analyses were performed using one-way ANOVA followed by Holm–Sidak post hoc corrections for multiple comparisons. Source data are provided in the Source Data file.

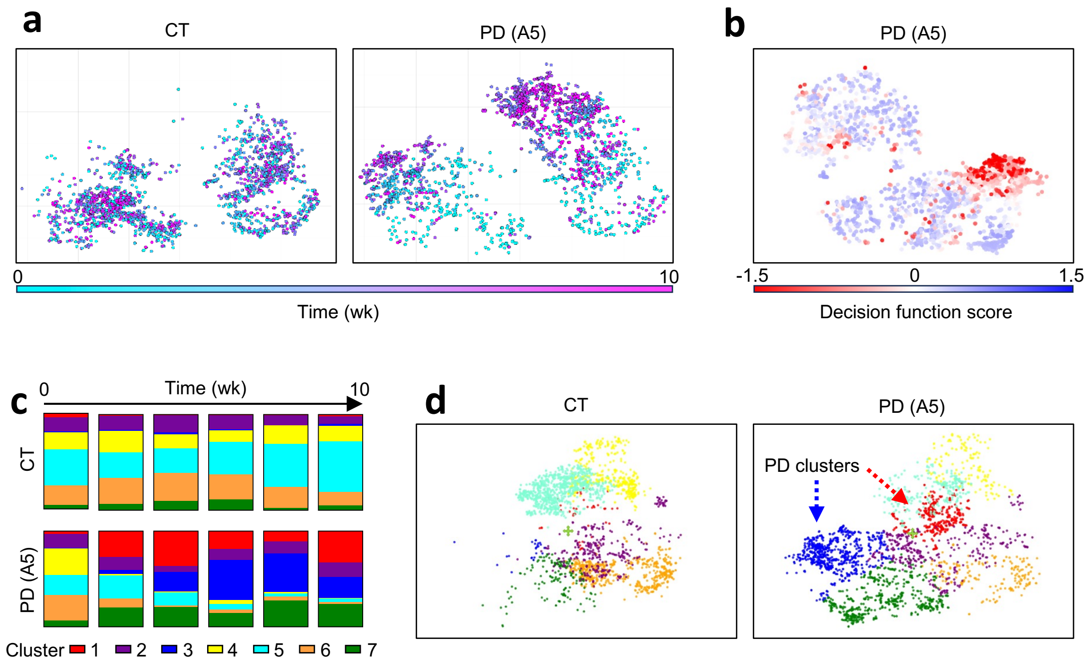

Supplementary Figure 4. Comparison temporal variabilities with t-SNE datasets.

(a) Temporal dynamics of t-SNE datasets (3D) for the Control (CT) and PD (A5) groups, shown in 2D flatted plots. **(b)** Anomaly detection results for the PD (A5) group, compared to the CT group. Each group contains dataset, covering the entire experimental period (0–10 wk). **(c)** Time-series bar graphs of the proportionality of *k*-means clustering labels for the CT and PD groups. **(d)** *K*-means clustering results, labelled with cluster 1–7 for the CT and A5 groups (0–10 wk). The PD specific clusters (dots in red [label 1] and blues [label 5]) are indicated with the dashed arrows.

a

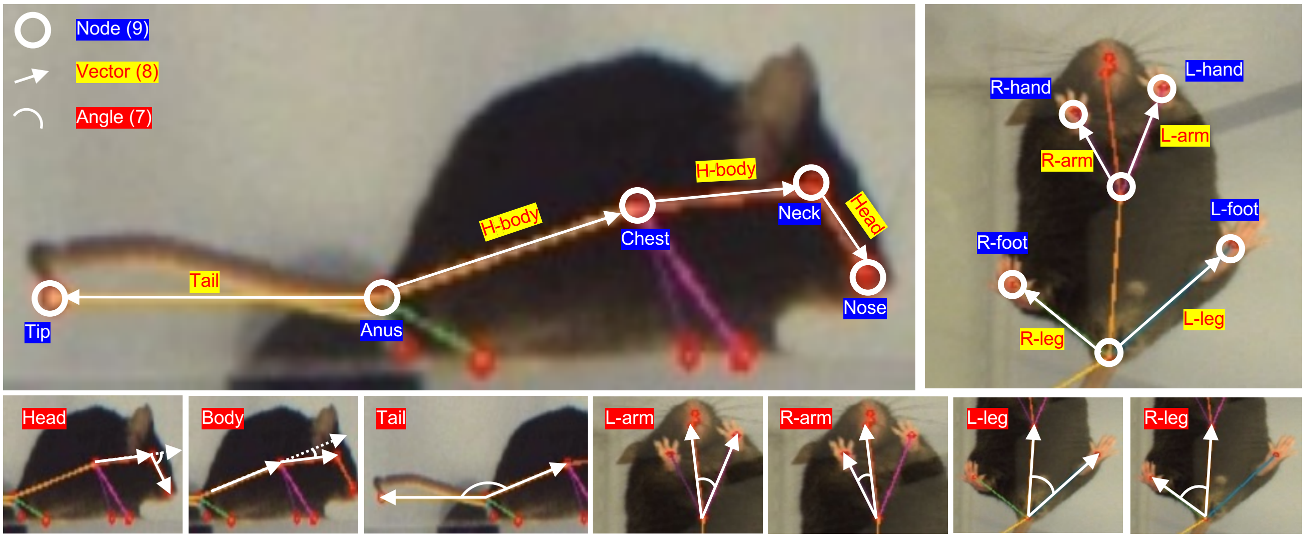

b

Name of Engineered feature	Node velocity	Angle	Paired node distance	Paired angle difference	Episode temporal domain parameters
	Nose	Head	Nose and anus [3]	R-leg and L-leg [17, 28]	Duration [2]
	Neck [9]	Body [7]	Neck and anus [25, 29, 30]	R-leg and R-arm [13]	Interval [6]
	Anus [19]	Tail [4]	Chest and anus [27]	R-leg and L-arm [26]	Period
	Chest [11, 16, 22]	L-arm	R-foot and anus	R-arm and L-arm [5, 20]	Frequency
	R-foot	R-arm	R-hand and anus	L-leg and L-arm [15]	
	L-foot	L-leg	L-hand and anus	L-leg and R-arm [23]	
	R-hand	R-leg	L-foot and anus	Body and head	
	L-hand		Neck and chest	Body and tail	
	Tip [24]		Nose and chest [12]		
			R-foot and L-foot [8, 10, 18, 21]		
			R-hand and L-hand [1, 14]		
			R-foot and L-hand		
			R-foot and R-hand		
			L-foot and L-hand		
1D (z)	Yes	No	Yes	No	Not applicable
2D (xy)	Yes	No	Yes	No	Not applicable
3D (xyz)	Yes	Yes	Yes	Yes	Not applicable
Statistics	Yes	Yes	Yes	Yes	Not applicable
Sub-total	108	28	168	32	4
Total	340 features created				

Supplementary Figure 5. Description of features engineered to develop AI models for PD diagnosis in mice.

(a) Snapshot images of mouse posture, illustrating the key nodes (depicted by circles, n = 9) detected by AVATARnet, and action skeletons (depicted by arrows, n = 8) derived from pairs of key nodes, and angles (open circles, n = 7), computed by AVATARpy. The arrows, representing the action skeletons as vectors, forming the angles. The dashed arrows indicate the respective vector shifted in parallel to itself, letting a pair of tails of vectors could join to form an angle. **(b)** A table, summarising the list of parent features engineered in five feature categories. These include four kinematic groups for motion clips – node velocity, node angle, paired node distance, and paired angle difference – and a temporal domain parameter for motion episodes. These kinematic features were then aggregated into statistical metrics. The parent features of the optimal features are marked with the superscripts, corresponding to the feature ranks from 1 to 30. Statistics include mean, min, max, std for each clip.

a

	CT	A5
Number of mice used for ML	10	11
Dataset included wk	0 - 10 wk	4 - 10 wk
Number of clips	5,855	11,066
+ Additional clips (0 wk)	5,249	0
Number of clips for ML (train/validation)	7,661/3,444	7,616/3,449

b

Parameter setting for training Extreme Gradient Boosting (XGB)

- learning_rate: 0.3
- n_estimators: 100
- max_depth: 6
- Subsample: 1
- Colsample_bytree: 1
- gamma: 0
- booster: "gbtree"
- reg_lambda: 1
- reg_alpha: 0

c

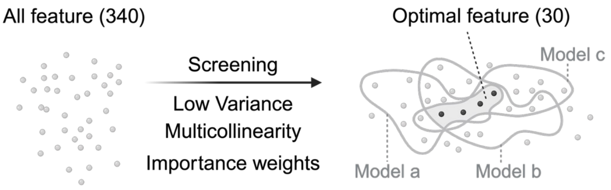

d

Model	All features	Optimal features
Extreme Gradient Boosting	0.93	0.90
Extra Trees Classifier	0.89	0.90
Random Forest Classifier	0.90	0.89
Light Gradient Boosting Machine	0.91	0.89
Gradient Boosting Classifier	0.88	0.86
Ada Boost Classifier	0.85	0.83
Decision Tree Classifier	0.81	0.81
Logistic Regression	0.87	0.81
Quadratic Discriminant Analysis	0.79	0.81
Linear Discriminant Analysis	0.88	0.81
Ridge Classifier	0.88	0.81
K Neighbors Classifier	0.75	0.80
SVM - Linear Kernel	0.82	0.76
Naive Bayes	0.73	0.76
Dummy Classifier	0.49	0.50

e **f**

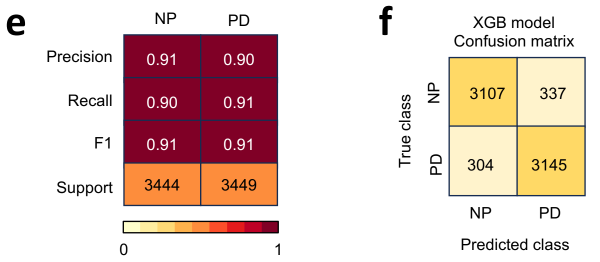

g

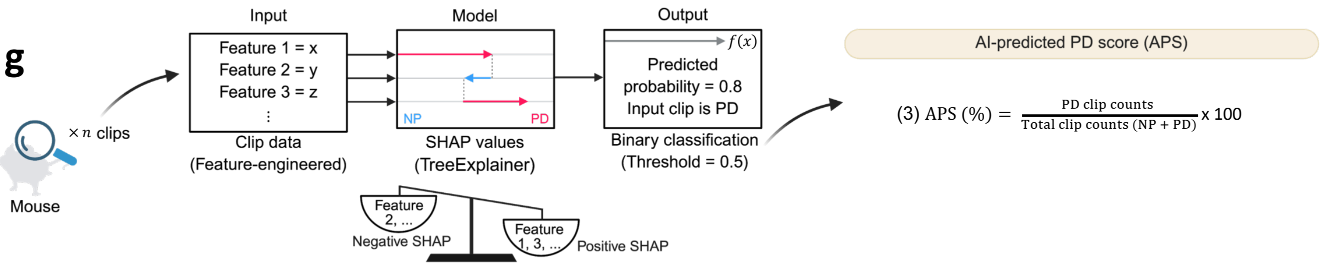

$$\text{(3) APS (\%)} = \frac{\text{PD clip counts}}{\text{Total clip counts (NP + PD)}} \times 100$$

Supplementary Figure 6. Development and comparison of AI models for PD diagnosis.

(a) Summary table of balanced dataset compositions, prepared from Control (CT) and PD (A5) groups for AI model development for PD diagnosis. **(b)** Summary table of parameter settings applied for XGB model training with PyCaret (version 2.3.10). See Supplementary Table 2 for default parameter settings of other classifiers. Default parameters may differ in more recent PyCaret versions. **(c)** Schematic illustration of the feature selection process, outlining the screening criteria and how the optimal features were identified from the comparison of various classifiers. **(d)** Comparison of various classifiers' performance when trained with full feature set (n = 340) and the optimal feature set (n = 30), detailing accuracy on the classification of validation dataset. **(e)** Summary of the XGB model evaluation metrics. **(f)** Confusion matrix of the XGB model in the classification of the cross-view (CV) validation dataset. **(g)** Schematic illustration, detailing the derivation of PD scores from the AI model's predictions on motion clips (as non-PD [NP] or PD), for each mouse. Abbreviations: ML, machine learning; NP, non-PD. Schematics were created in BioRender. Heo, W. (2025) https://BioRender.com/cykyauz.

a

Cross-view (CV)

	Model	Accuracy	AUC	Recall	Prec.	F1	Kappa	MCC	TT (Sec)
xgboost	Extreme Gradient Boosting	0.9029	0.9635	0.9086	0.8978	0.9031	0.8057	0.8059	4.218
et	Extra Trees Classifier	0.8951	0.9618	0.8713	0.9142	0.8921	0.7901	0.7911	0.854
rf	Random Forest Classifier	0.8942	0.959	0.8895	0.8972	0.8933	0.7883	0.7884	2.208
lightgbm	Light Gradient Boosting Machine	0.8884	0.9566	0.8836	0.8915	0.8875	0.7768	0.7769	0.466
gbc	Gradient Boosting Classifier	0.8621	0.9373	0.8514	0.8693	0.8602	0.7243	0.7246	5.591
ada	Ada Boost Classifier	0.8293	0.9127	0.8205	0.8343	0.8272	0.6585	0.6589	1.248
dt	Decision Tree Classifier	0.811	0.8111	0.8366	0.795	0.8152	0.6221	0.6231	0.633
lr	Logistic Regression	0.8101	0.8851	0.7991	0.816	0.8073	0.6202	0.6205	2.631
qda	Quadratic Discriminant Analysis	0.8069	0.8836	0.731	0.8606	0.7903	0.6136	0.6208	0.195
lda	Linear Discriminant Analysis	0.8064	0.8818	0.7909	0.8151	0.8027	0.6127	0.6132	0.214
ridge	Ridge Classifier	0.8058	0	0.7915	0.8137	0.8023	0.6115	0.612	0.222
knn	K Neighbors Classifier	0.7973	0.873	0.7554	0.8234	0.7878	0.5946	0.5968	0.475
svm	SVM - Linear Kernel	0.764	0	0.7624	0.7775	0.762	0.5281	0.5391	0.338
nb	Naive Bayes	0.757	0.8422	0.657	0.8196	0.7292	0.5136	0.524	0.193
dummy	Dummy Classifier	0.5017	0.5	0	0	0	0	0	0.176

Cross-subject (CS)

	Model	Accuracy	AUC	Recall	Prec.	F1	Kappa	MCC	TT (Sec)
xgboost	Extreme Gradient Boosting	0.8959	0.9612	0.8882	0.8996	0.8913	0.7916	0.7948	5.068
rf	Random Forest Classifier	0.89	0.9551	0.8722	0.901	0.8839	0.7797	0.7828	2.831
et	Extra Trees Classifier	0.8892	0.9574	0.8451	0.9227	0.8787	0.7781	0.7836	0.993
lightgbm	Light Gradient Boosting Machine	0.8762	0.9516	0.8642	0.8814	0.8693	0.7521	0.7559	0.553
gbc	Gradient Boosting Classifier	0.8553	0.9318	0.8377	0.8627	0.846	0.7103	0.7144	6.558
ada	Ada Boost Classifier	0.8287	0.9085	0.8141	0.8331	0.8203	0.6571	0.6605	1.601
dt	Decision Tree Classifier	0.8105	0.8106	0.8222	0.7995	0.8096	0.6209	0.6226	0.611
lr	Logistic Regression	0.8066	0.88	0.7926	0.8088	0.7982	0.613	0.6153	2.045
qda	Quadratic Discriminant Analysis	0.8045	0.8798	0.7223	0.8538	0.7765	0.6081	0.6175	0.314
lda	Linear Discriminant Analysis	0.8025	0.8773	0.783	0.8077	0.7923	0.6047	0.6073	0.294
ridge	Ridge Classifier	0.8019	0	0.7839	0.8061	0.7922	0.6035	0.6059	0.313
svm	SVM - Linear Kernel	0.7872	0	0.7787	0.7848	0.7757	0.5741	0.58	0.617
knn	K Neighbors Classifier	0.7865	0.8649	0.7446	0.8059	0.7708	0.5726	0.5765	0.552
nb	Naive Bayes	0.7574	0.8382	0.6582	0.8088	0.721	0.5137	0.5236	0.326
dummy	Dummy Classifier	0.5053	0.5	0	0	0	0	0	0.295

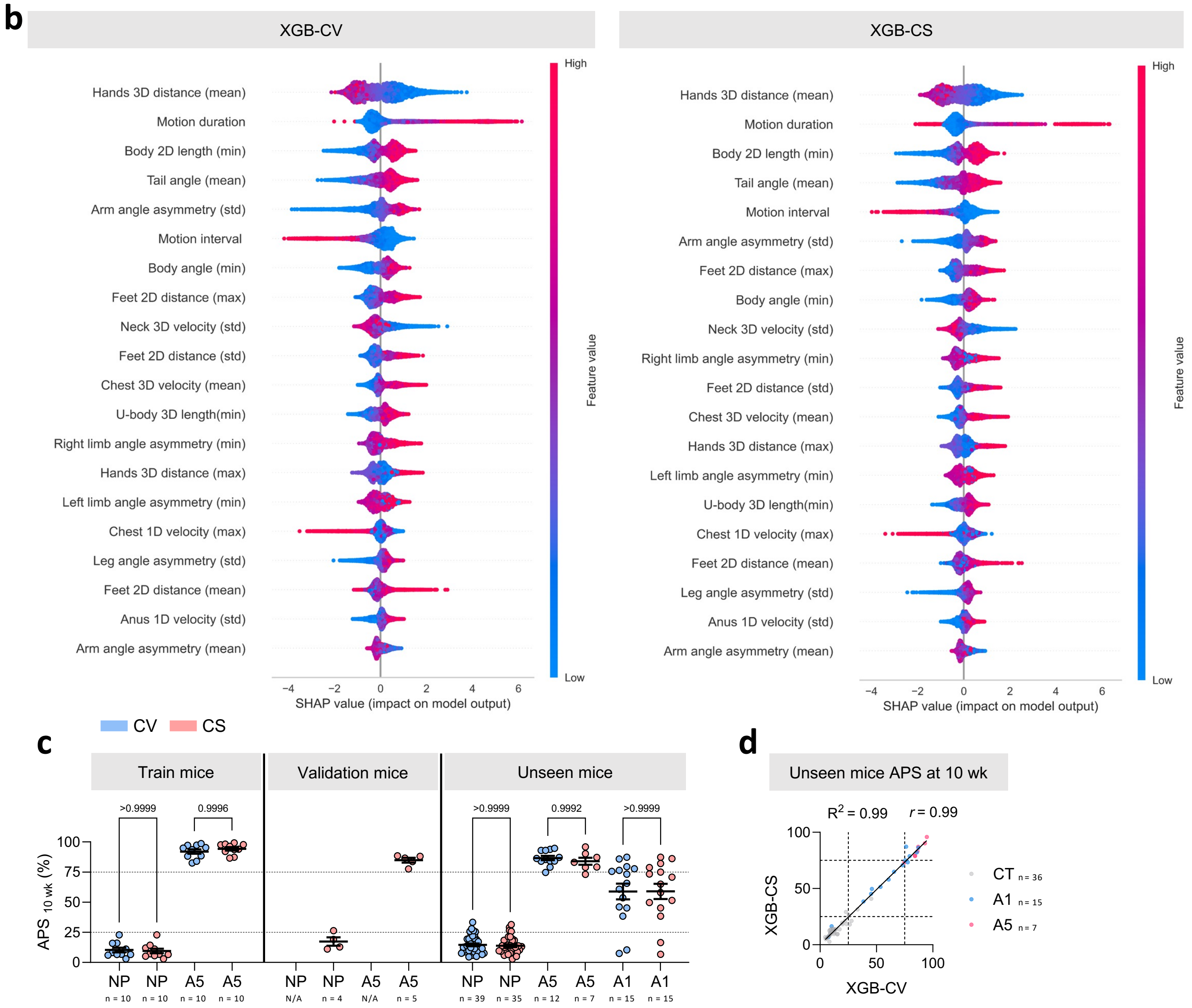

Supplementary Figure 7. Comparison of cross-view (CV) and cross-subject (CS) validation methods.

(a) Tables summarising the evaluation metrics for the models trained and validated using CV (left) and CS (right) methods. **(b)** SHAP summary plots of the XGB models computed on the CV (left) and CS (right) validation datasets. The top 4 features are identically ranked, with 100% of the top 20 features overlapping between the two methods. Comparisons of the feature order, Spearman correlation was ≈ 0.9789 (*p* ≈ 7.15e-14) and of the absolute SHAP values, the Pearson correlation was ≈ 0.9999 (*p* ≈ 4.81e-114). **(c)** Prediction results (AI-predicted PD scores; APS) for the mice across datasets, as predicted by the XGB-CV model (blue) and the XGB-CS model (red). No significant differences were found across all groups. Importantly, XGB-CV model assessed all unseen A5 mice into the severe PD category (APS > 75% in rounded values). **(d)** Comparison of the APS for each mouse by the two models. The dashed line indicates the simple linear regression of the all data plotted. Two model predictions were highly correlated (Pearson's correlation, *r* = 0.99). Collectively, these findings robustly confirm the generalisability and stability of the CV-based XGB model. Data are shown as mean ± SEM; sample sizes (n) are indicated in plots. Statistical analysis was performed using one-way ANOVA followed by Holm–Sidak post hoc corrections for multiple comparisons. Source data are provided in the Source Data file.

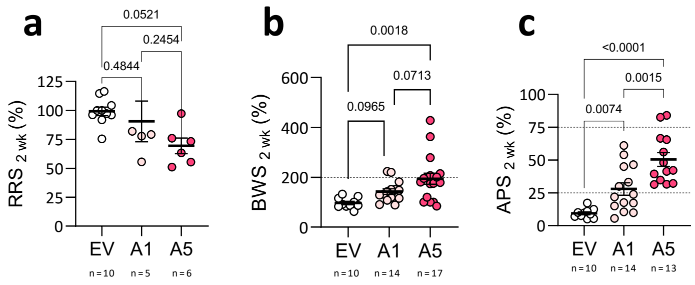

Supplementary Figure 8. Comparison of APS, RRS, and BWS for early PD detection at 2 wk.

(a–c) Comparisons of RRS, BWS and APS assessments for the EV, A1 and A5 groups at 2 wk post-surgery (RRS [%]: EV = 99.22 ± 3.67, A1 = 90.56 ± 17.62, A5 = 69.37 ± 6.84; BWS [%]: EV = 96.74 ± 6.69, A1 = 142.9 ± 11.16, A5 = 194.2 ± 22.37; APS [%]: EV = 9.42 ± 1.15, A1 = 27.94 ± 4.70, A5 = 50.41 ± 5.23). Data are shown as mean ± SEM; sample sizes (n) are indicated in plots. Statistical analysis was performed using one-way ANOVA followed by Holm–Sidak post hoc corrections for multiple comparisons. Source data are provided in the Source Data file. Abbreviations: RRS, rotarod test score; BWS, elevated beam walking test score; APS, AI-predicted PD score.

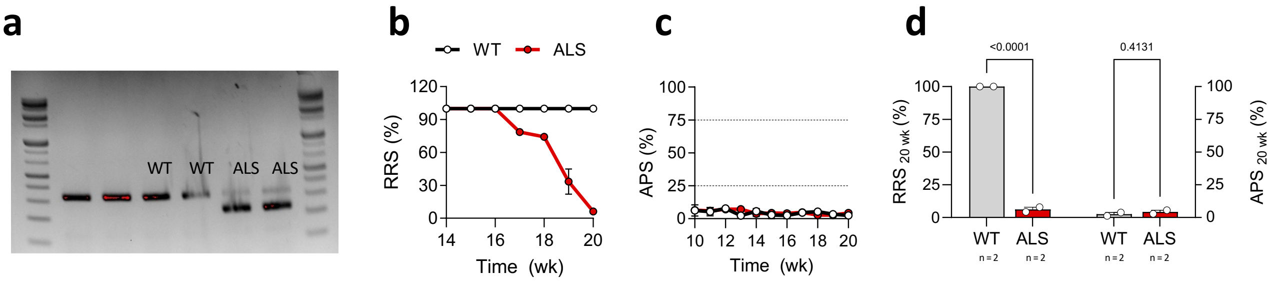

Supplementary Figure 9. Validation of APS for PD specificity using an amyotrophic lateral sclerosis (ALS) mouse model.

(a) Genotyping results of wild-type (WT) and ALS model (SOD1-G93A heterozygous transgenic) mice. **(b–d)** Comparisons of rotarod test score (RRS) and AI-predicted (XGB model) PD score (APS) assessments between WT and ALS mice, shown as fitted line and dot graphs for both longitudinal (10 or 14 - 20 wk of age) and endpoint (20 wk of age) analyses (RRS [%]: WT = 100 ± 0.00, ALS = 6.11 ± 1.85; APS: WT = 2.52 ± 1.33, ALS = 4.24 ± 1.38; n = 2 per group). ALS mice exhibit clear motor dysfunction as assessed by RRS, while APS remains comparable to WT. Note that ALS mice have shorter lifespans (50% survival at 128.9 ± 9.1 days), so the experimental scheme was adjusted to the 10–20 wk age range. Data are shown as mean ± SEM; sample sizes (n) are indicated in plots. Statistical analysis was performed using two-way ANOVA followed by Fisher's LSD post hoc tests without correction for multiple comparisons. Source data are provided in the Source Data file.

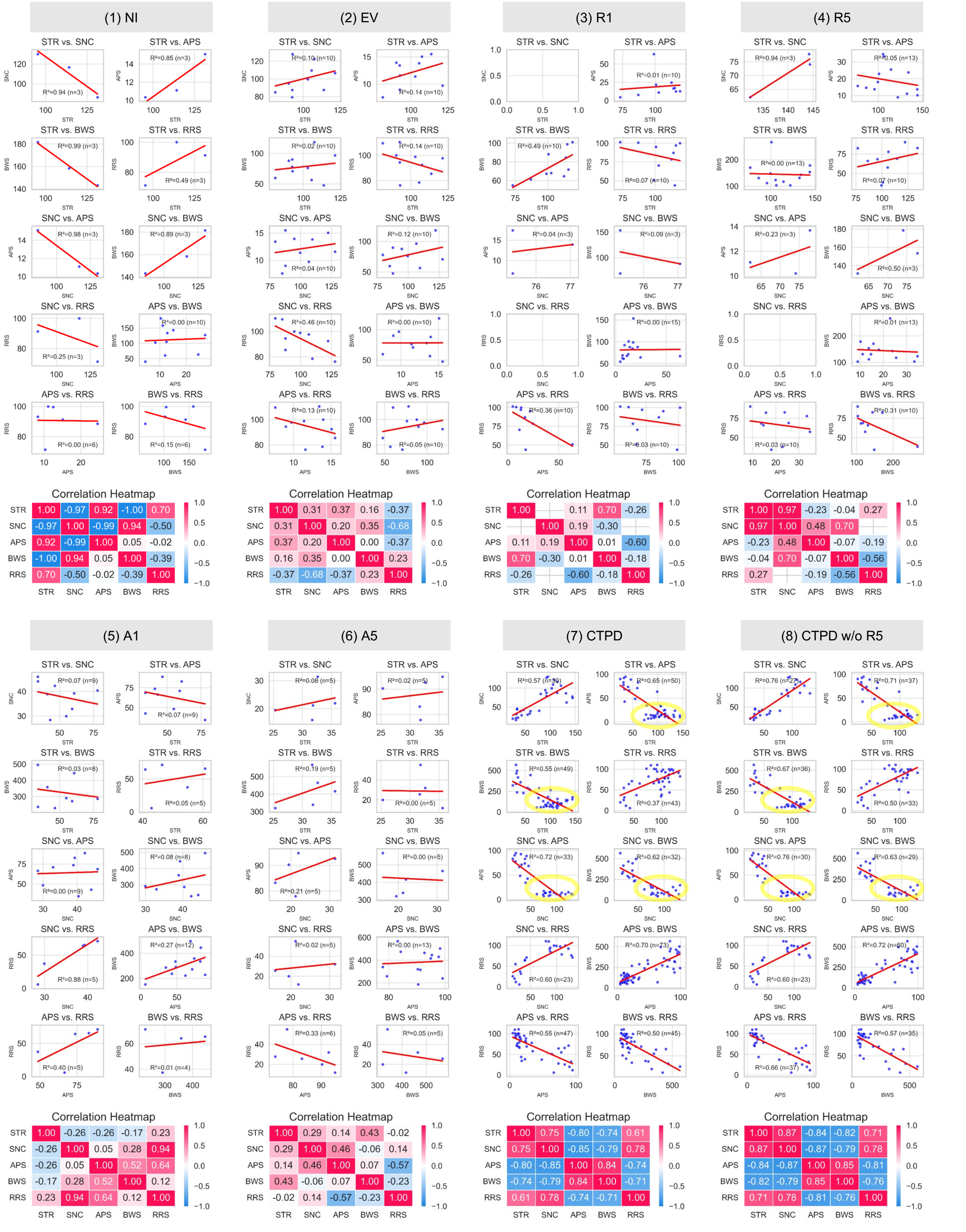

Supplementary Figure 10. Detailed plots of correlation analysis with varied dataset filtering at group level.

Line graphs (fitting line in red) of pairs of metrics and a heatmap of Pearson's correlation values. Datasets, including all CT and PD groups were filtered by group(s) and then filtered by each pair of metrics for the computation of Pearson's correlation (not by all metrics). Individual group filtered plot sets (#1-6), highlight variabilities in the correlation of different pairs of metrics as a characteristic for the group. Plot set #7 shows the overall data correlations between metrics. In plot set #8, the R5 group was excluded to show the effect of this group on decreasing the magnitude of correlations, particularly STR vs SNc. Yellow ovals on set #7 and #8 highlight the buffering capacity of DA system, reflected in flat clusters of APS and BWS. Abbreviations: STR, striatal TH intensity; SNC, SNc TH cell counts, RRS, rotarod test score; BWS, elevated beam walking test score; APS, AI-predicted PD score.

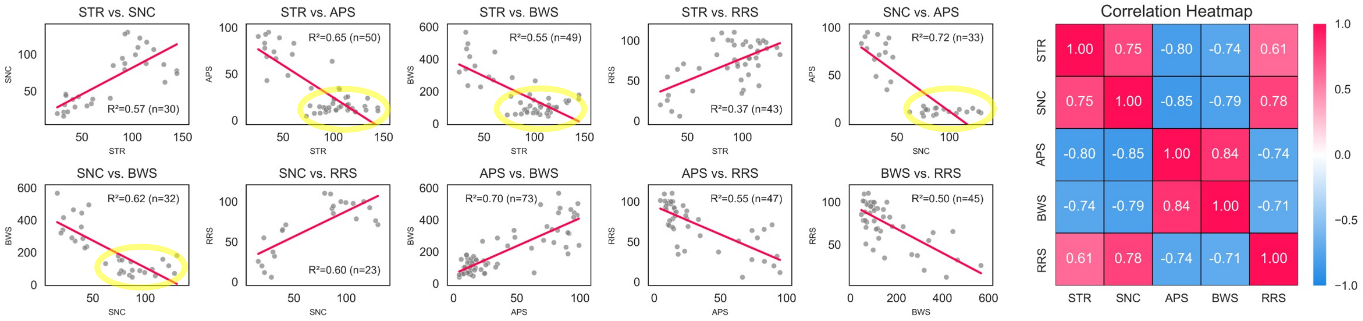

Supplementary Figure 11. Summary of correlation analysis.

Line graphs (fitting line in red) of pairs of metrics and a heatmap of Pearson's correlation values. For each computation, dataset (CT and PD groups) are filtered for each pair of metrics. Yellow ovals highlight the buffering capacity of DA system, reflected in flat clusters of APS and BWS. Abbreviations: STR, striatal TH intensity; SNC, SNc TH cell counts, RRS, rotarod test score; BWS, elevated beam walking test score; APS, AI-predicted PD score.

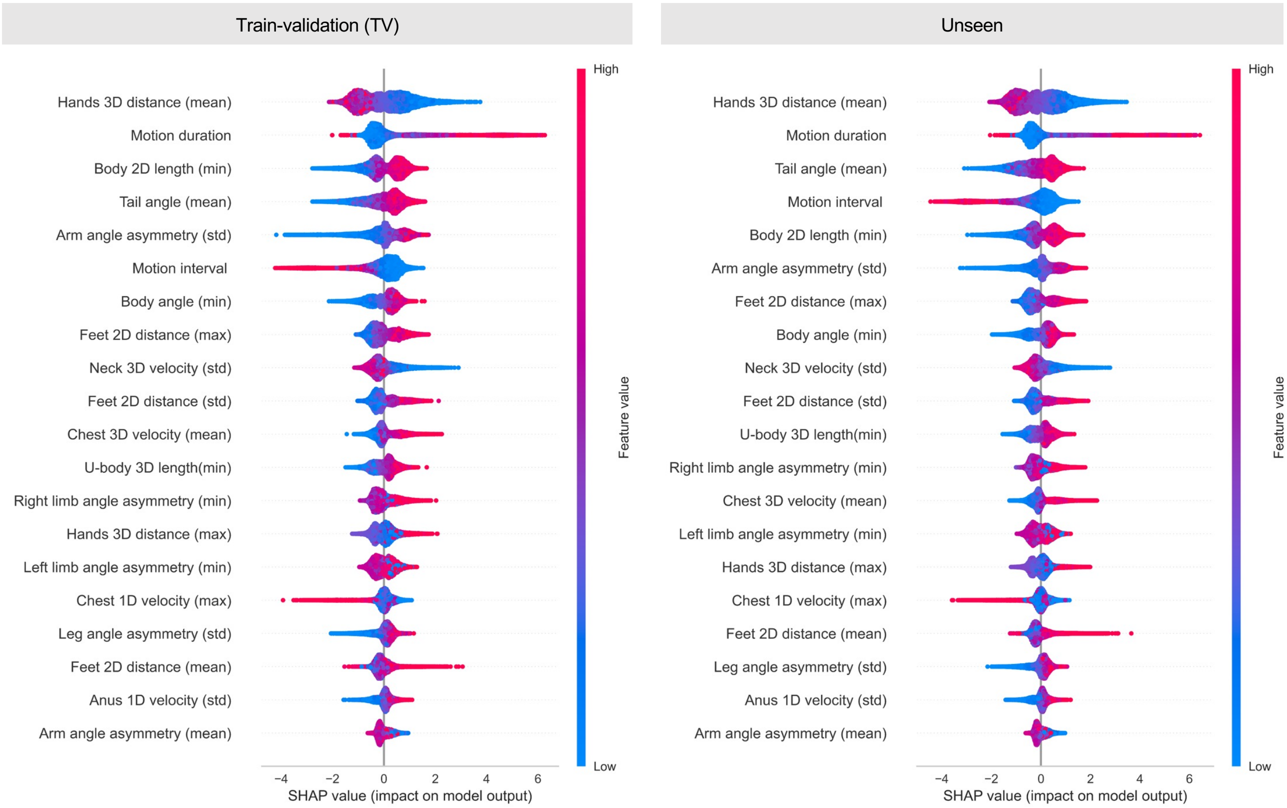

Supplementary Figure 12. Comparison train-validation (TV) and unseen datasets for top 20 feature importances.

Bar charts display the absolute mean SHAP values of the XGB models computed on the TV (left; NP = 10, A5 = 11 mice) and the unseen (right; NP = 40, A5 = 13 mice) datasets. The analysis revealed a 100% overlap in the top 20 features, with a Spearman correlation of ≈ 0.9835 ($p \approx$ 8.29e-15). A separate comparison of the absolute SHAP values for these top 20 features yielded a Pearson correlation of about 0.2013 ($p \approx$ 0.3948), indicating some variability in the absolute magnitudes; however, given the near-perfect consistency in ranking, this variation in magnitudes is considered of lesser concern. Together, these results provide robust quantitative evidence that the top 20 features remain highly relevant across the TV and unseen datasets, thereby confirming the stability and generalisability of the feature selection approach.
Abbreviations: NP, non-PD

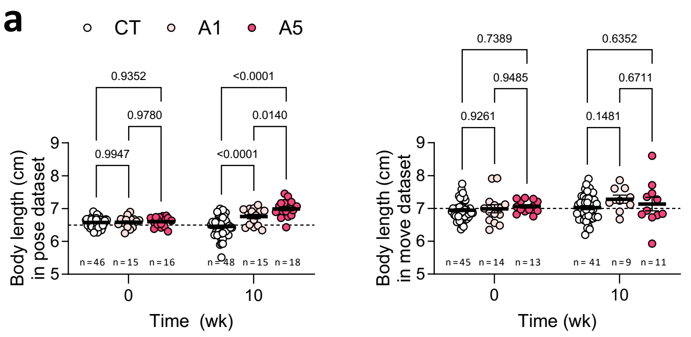

b

Parameters	P-value in 2-way ANOVA test	
	Pose dataset	Move dataset
Time	0.0006	0.0456
Group	<0.0001	0.2201
Time x Group	<0.0001	0.4359

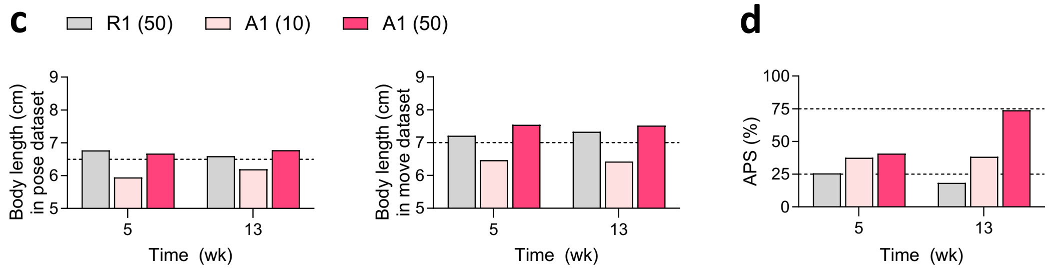

Supplementary Figure 13. Comparison of body lengths across non-PD (NP) and PD groups.

(a) Dot plots comparing body lengths (computed as the sum of 3D forebody [fbody; neck-chest] and hindbody [hbody; chest-anus] distances, averaged from frames with body angles within ±10°) across CT, A1, and A5 groups from both the raw pose and filtered move datasets at 0 and 10 wk post-injection. Please note that this measure differs from the 'body length (neck-anus distance)' presented in our original manuscript (see, Fig. 2a and Supplementary Fig. 5b). Pose dataset 0 wk: CT = 6.58 ± 0.02, A1 = 6.58 ± 0.04, A5 = 6.6 ± 0.04 cm; 10 wk: NP = 6.45 ± 0.04, A1 = 6.76 ± 0.07, A5 = 6.99 ± 0.06 cm; move dataset 0 wk: CT = 6.95 ± 0.05, A1: 6.99 ± 0.12, A5: 7.06 ± 0.05 cm; 10 wk: CT: 7.02 ± 0.06, A1: 7.28 ± 0.12, A5: 7.13 ± 0.21 cm. Two-way ANOVA tests were performed, and p-values were adjusted using Tukey's multiple comparisons method. In the raw pose dataset (left panel), significant differences in body lengths emerged at 10 wk, likely reflecting posture variations during non-moving (freeze) periods. In contrast, the move dataset (right panel) revealed no significant differences in body lengths between groups at either timepoint, indicating minimal confounds related to body size differences. **(b)** Table summarising the statistical results from two-way ANOVA tests comparing effects of group, time, and their interactions for both datasets. Significant effects were observed in the pose dataset whereas the move dataset showed no significant differences for group parameter, reinforcing the robustness of analyses based on this dataset. **(c)** Bar graphs depicting body lengths from the pose and move datasets for preliminary analyses of individual control (R1) and PD (A1) mice injected with virus at different ages (weeks indicated in parentheses). **(d)** APS measurements for the preliminary mice (R1[50], A1[10], A1[50]). Despite variations in body lengths related to age differences, APS remained robust and correctly reflected the expected PD status. APS remained normal in the R1 mouse despite larger body size, whereas APS increased to mild PD levels in A1 mice regardless of body length differences. Collectively, these results provide robust evidence that the distance-based features are unlikely to be confounded by body size (neck-chest-anus) differences, particularly within the move dataset used in current study. Statistical analysis was performed using two-way ANOVA followed by Tukey's post hoc correction for multiple comparisons.

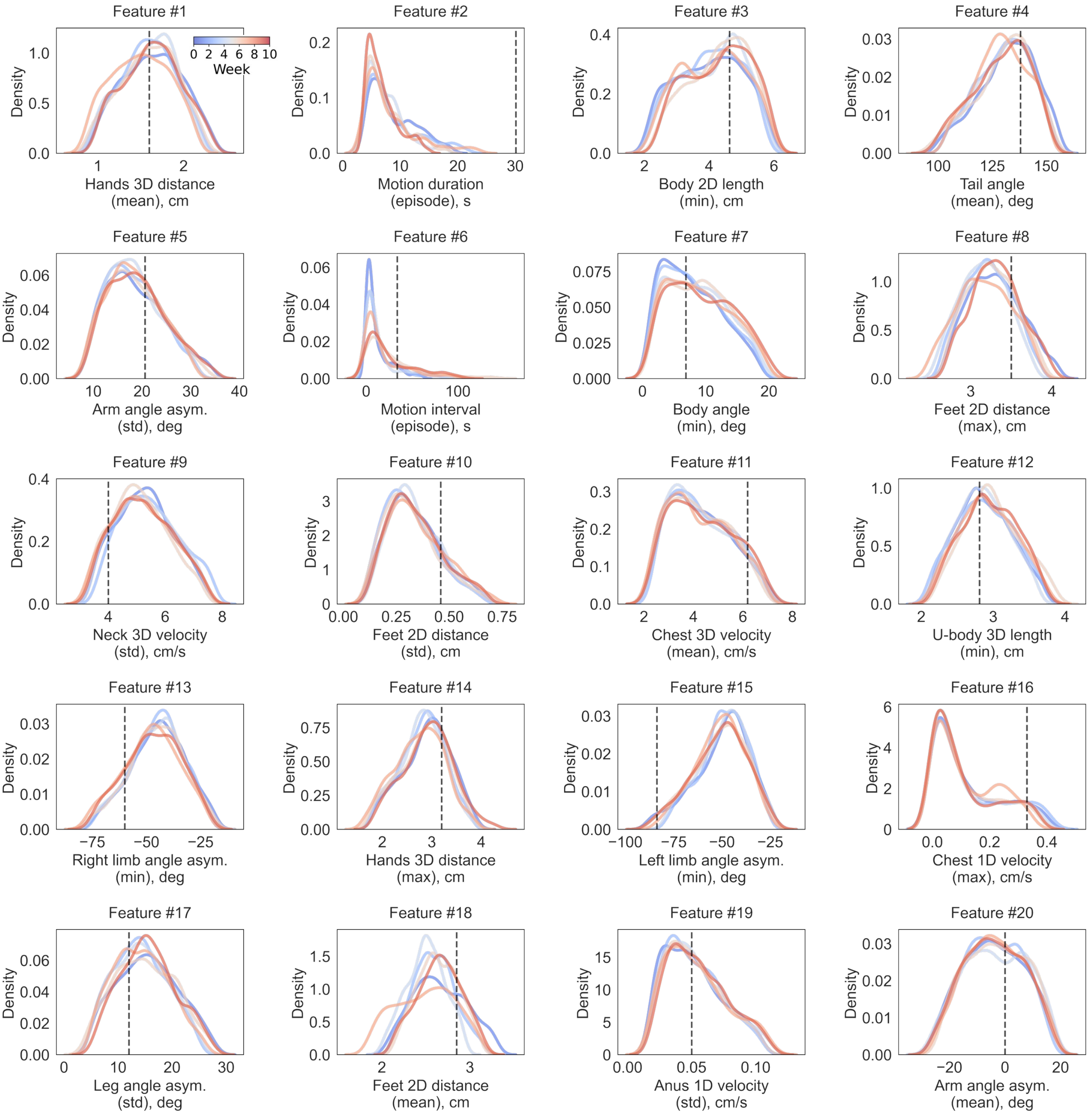

Supplementary Figure 14. CT group's longitudinal analysis of the top 20 features (XGB model) in the KDE plots.

For each feature, kernel density estimate (KDE) plots illustrate distribution shifts at distinct time points (0, 2, 4, 6, 8 and 10 wk). Vertical dashed lines indicate the key values.

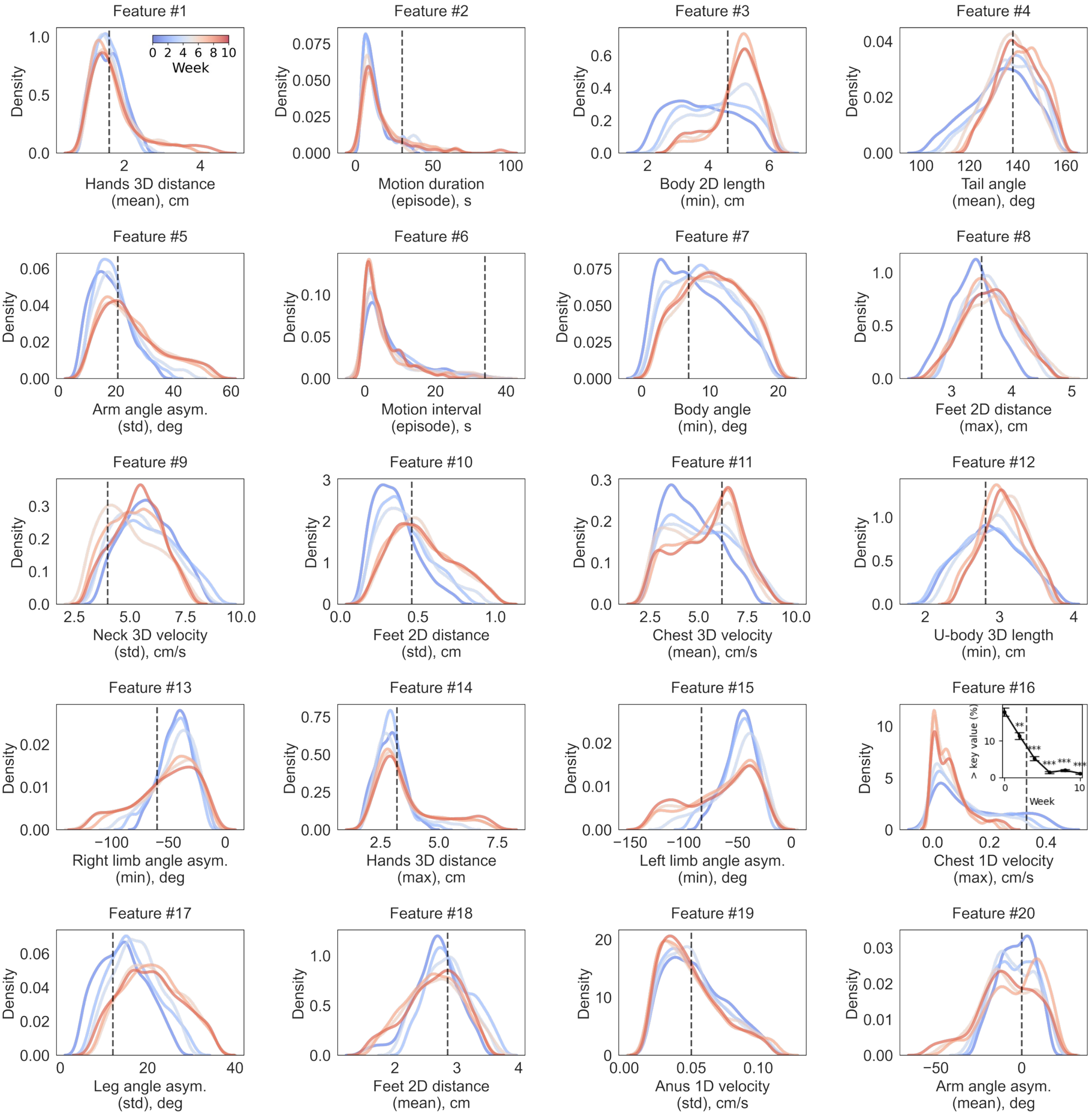

Supplementary Figure 15. PD (A5) group's longitudinal analysis of the top 20 features (XGB model) in the KDE plots.

For each feature, KDE plots illustrate distribution shifts at distinct time points (0, 2, 4, 6, 8 and 10 wk). Vertical dashed lines indicate the key values. For feature #16, a small line graph is shown above the KDE plot, representing the percentage of the data above the key value 0.33 cm (mean ± SEM, t-test compared to 0 wk) and source data are provided in the Source Data file.

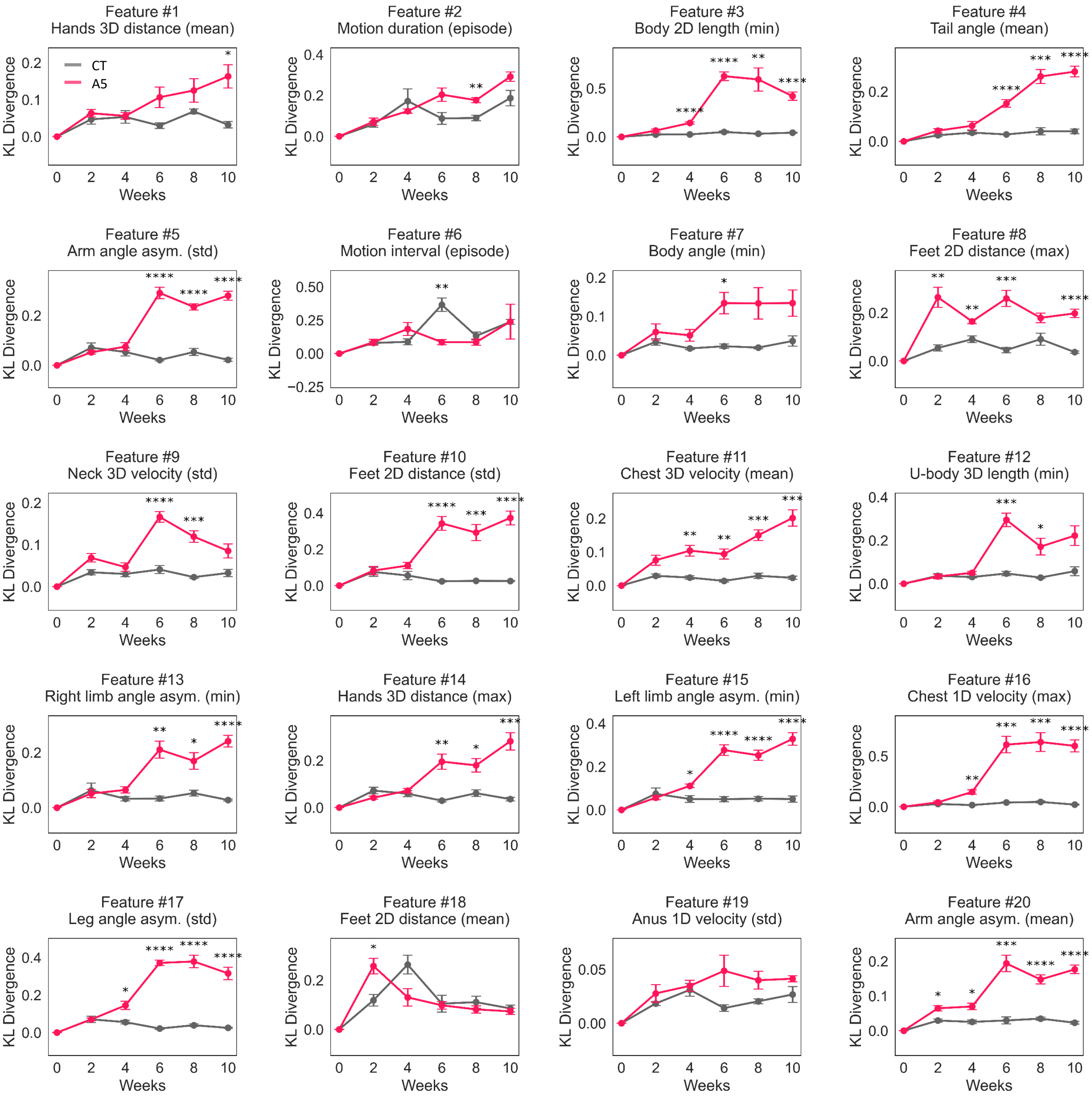

Supplementary Figure 16. Longitudinal comparison of KL divergences (XGB model) between the CT and PD (A5) groups.

For each group, the KL divergences of each feature from the respective baseline (0 wk) were computed over the experimental duration (0–10 wk) and plotted as line graphs (mean ± SEM. For each group, the KL divergence of each feature relative to its baseline value (0 wk) was computed across the experimental duration (0–10 wk). Each group consisted of 6 sampling sets, each containing 150 randomly selected clips. A two-way ANOVA (group × week) was performed for each feature, followed by weekly t-tests between groups (CT vs. A5). The resulting *p*-values from the weekly t-tests were corrected for multiple comparisons using the Bonferroni method. Detailed statistical results are provided in Supplementary Table 3. Source data are provided in the Source Data file.

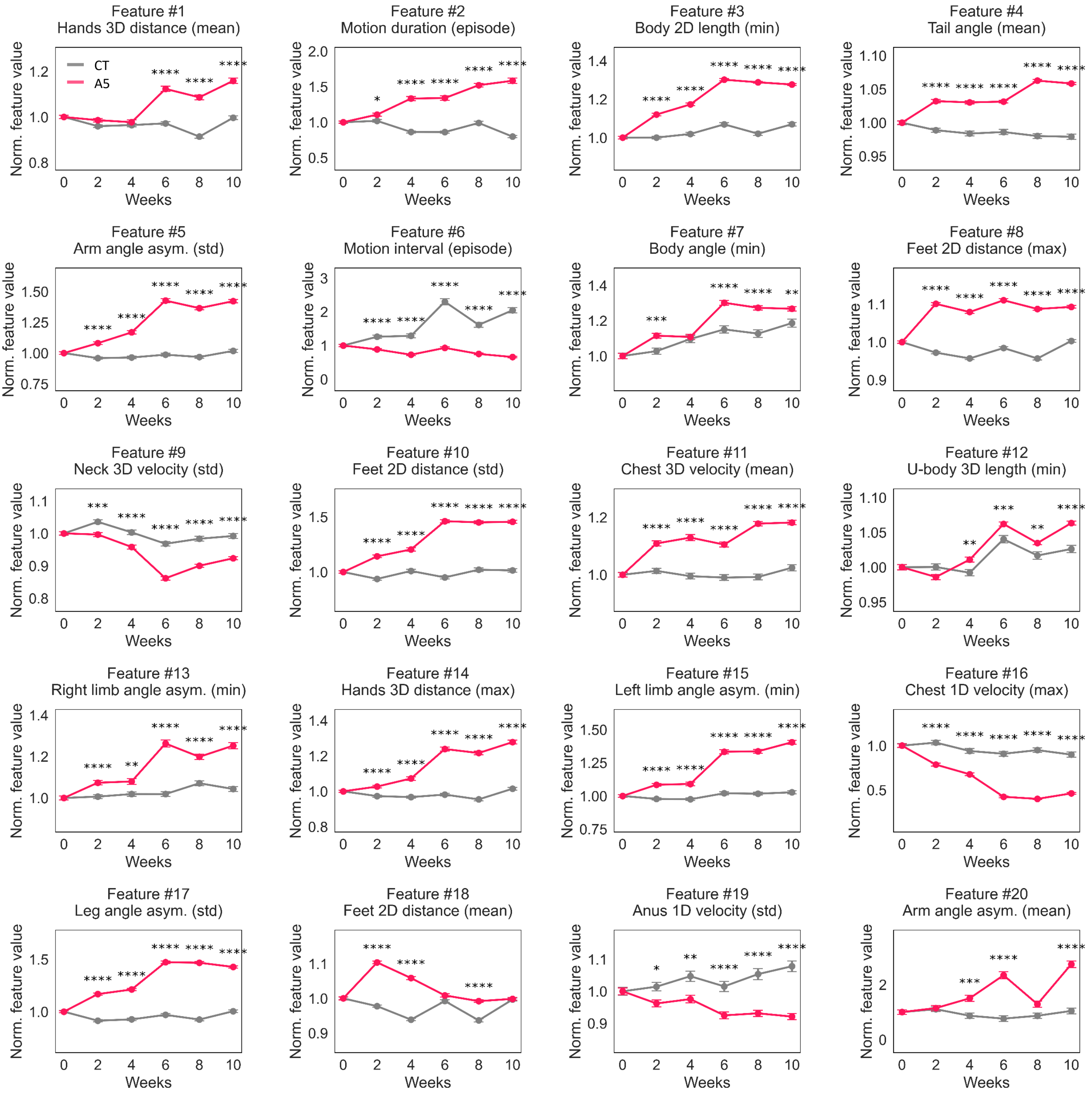

Supplementary Figure 17. Longitudinal comparison of feature values (XGB model) between the CT and PD (A5) groups.

(a) For each feature, the normalised feature values (mean ± SEM) were plotted over the experimental duration. For each feature, the values at each week were divided by the group's corresponding baseline (0 wk) mean value, and the normalised data were analysed over the experimental duration (0–10 wk). Each group consisted of 6 sampling sets, each containing 150 randomly selected clips. Two-way ANOVA (group × week) was performed for each feature on the normalised data, followed by weekly t-tests comparing the CT and A5 groups. *P*-values from weekly t-tests were corrected for multiple comparisons using the Bonferroni method. Detailed statistical results are provided in Supplementary Table 4. Source data are provided in the Source Data file.

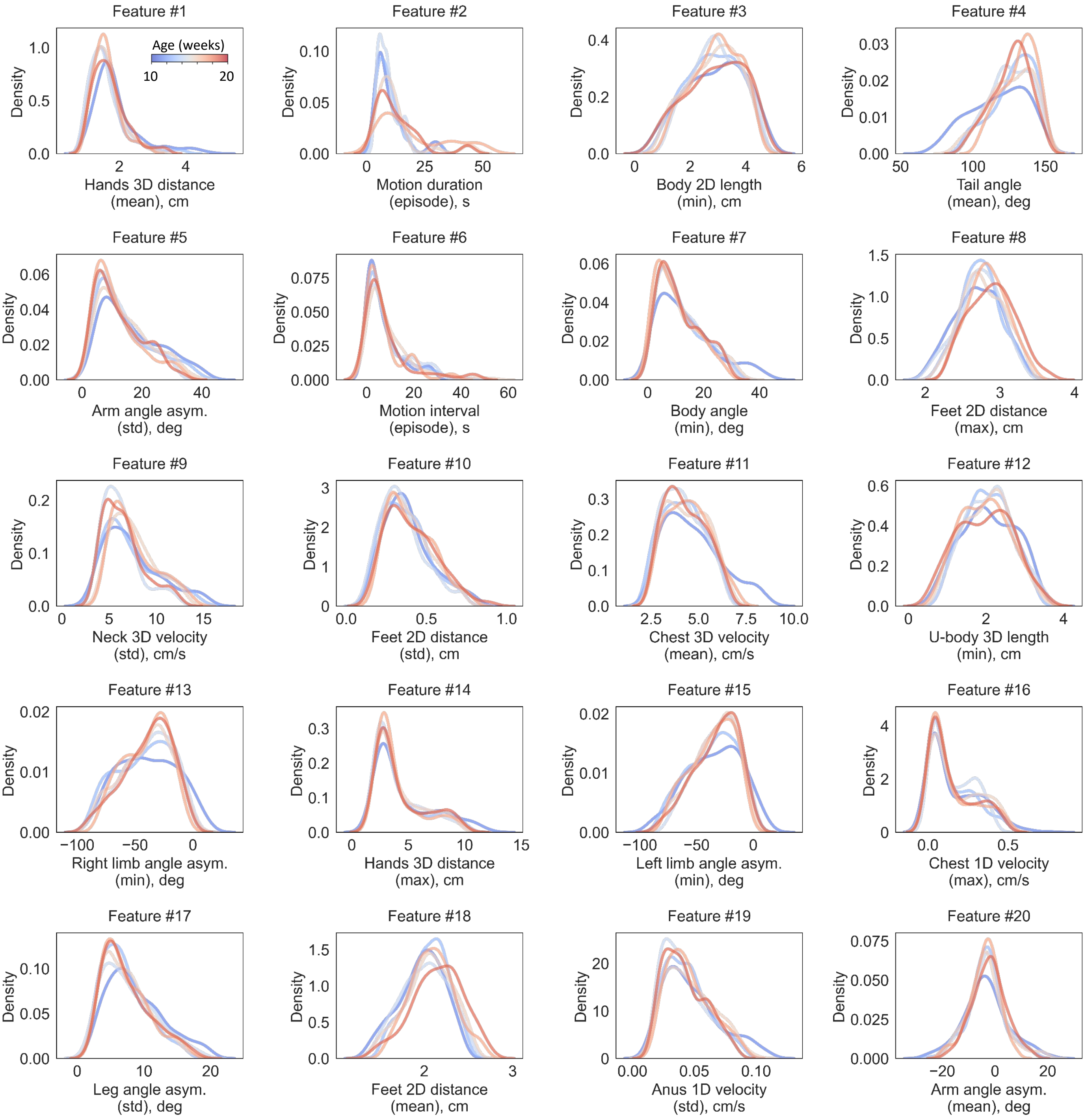

Supplementary Figure 18. NALS group's longitudinal analysis of the top 20 features (XGB model) in the KDE plots.

For each feature, KDE plots illustrate distribution shifts at distinct time points (10, 12, 14, 16, 18, and 20 wk [age]). Direct comparisons between CT (non-PD control in Supplementary Fig.14) and NALS (non-ALS control) groups are limited by age and gender differences.

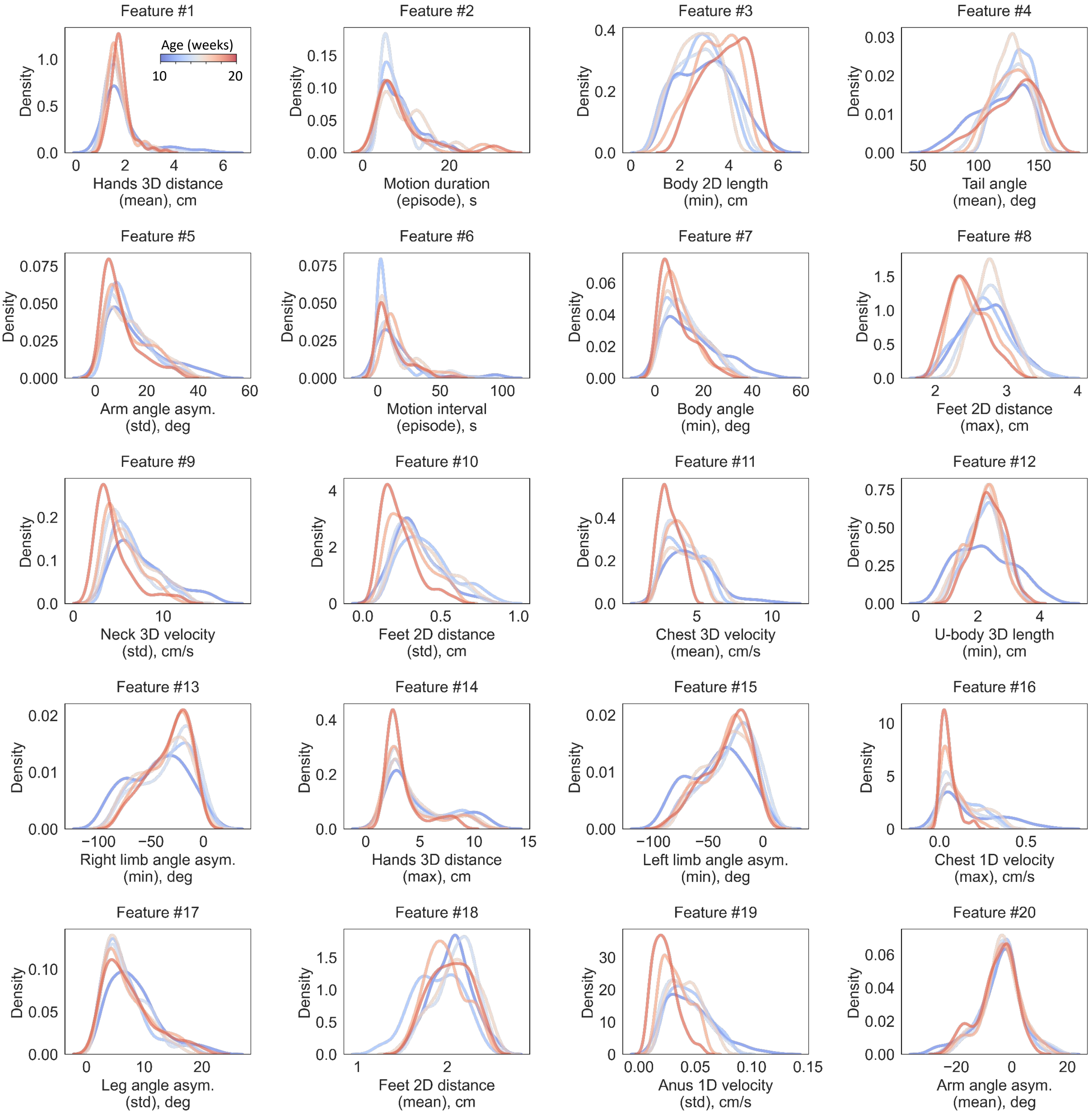

Supplementary Figure 19. ALS group's longitudinal analysis of the top 20 features (XGB model) in the KDE plots.

For each feature, KDE plots are shown at different time points (10, 12, 14, 16, 18, and 20 wk [age]). Direct comparisons between PD (A5 in Supplementary Fig.15) and ALS groups are limited by age and gender differences; however, the trends observed in the KDE plots are notable. For instance, limb asymmetry features appear less affected in ALS compared to PD mice, and generally, the feature distributions in ALS mice progressively exhibit increased positive kurtosis over time.

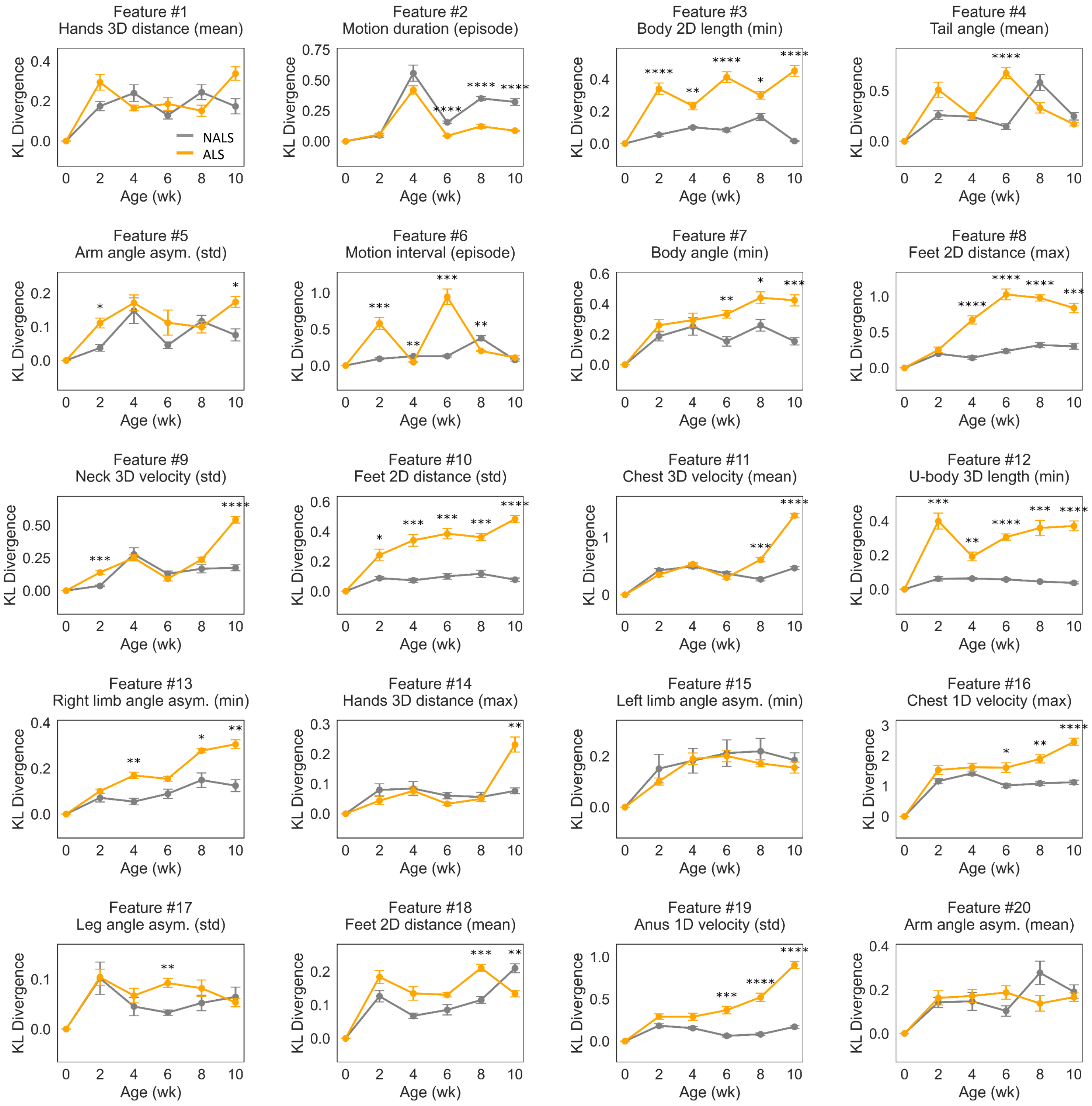

Supplementary Figure 20. Longitudinal comparison of KL divergences (XGB model) between the NALS and ALS groups.

For each group, the KL divergence of each feature relative to its baseline value (10 wk, non-symptomatic age) was computed across the experimental duration (10–20 wk [age]). Each group consisted of 6 sampling sets, each containing 150 randomly selected clips. A two-way ANOVA (group × week) was performed for each feature, followed by weekly t-tests between groups (NALS vs. ALS). The resulting p-values from the weekly t-tests were corrected for multiple comparisons using the Bonferroni method. Detailed statistical results are provided in Supplementary Table 5. Source data are provided in the Source Data file.

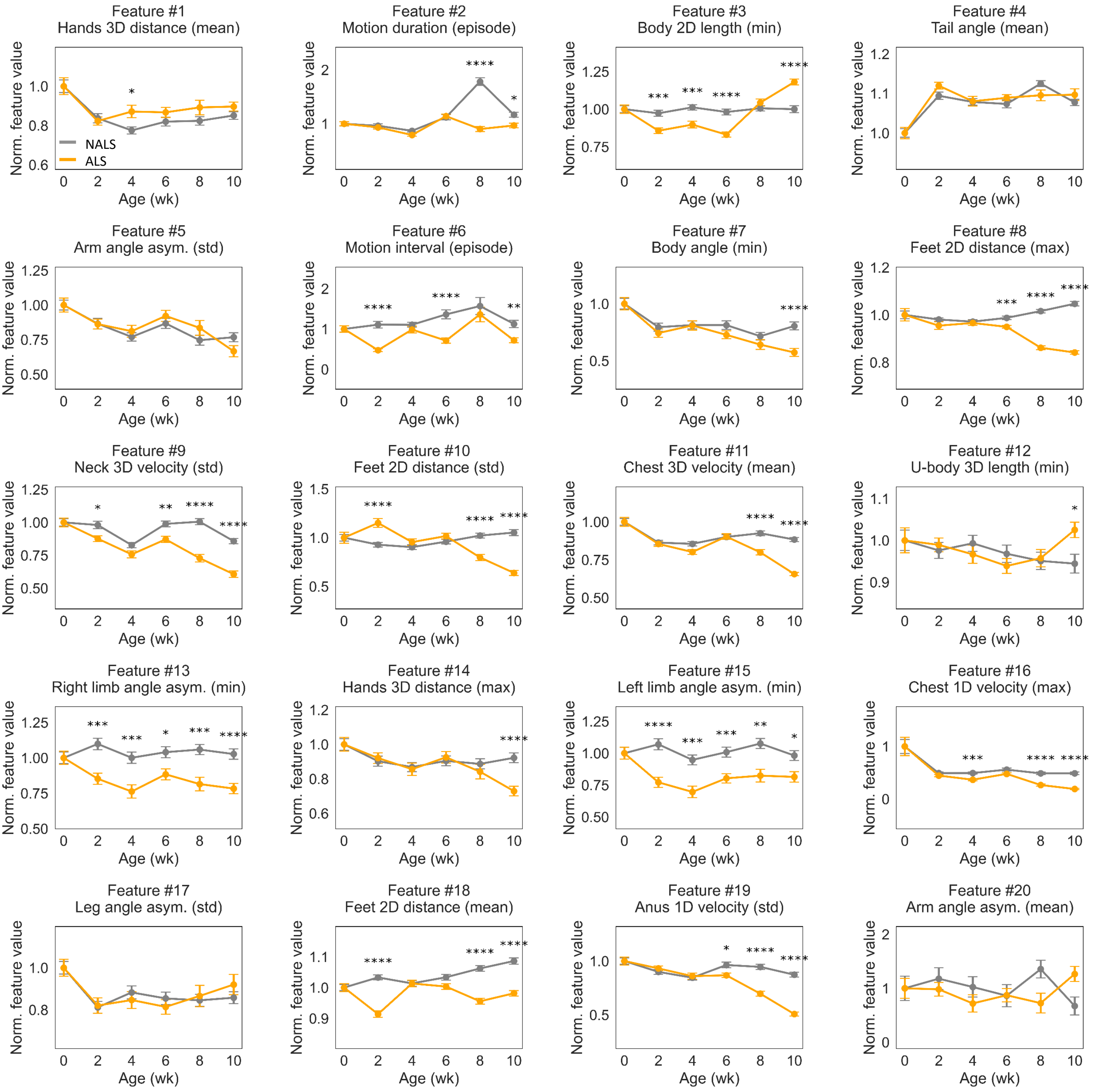

Supplementary Figure 21. Longitudinal comparison of feature values (XGB model) between the NALS and ALS groups.

For each feature, the normalised feature values (mean ± SEM) were plotted over the experimental duration. For each feature, the values at each week were divided by the group's corresponding baseline (10 wk, non-symptomatic age) mean value, and the normalised data were analysed over the experimental duration (10–20 wk [age]). Each group consisted of 6 sampling sets, each containing 150 randomly selected clips. Two-way ANOVA (group × week) was performed for each feature on the normalised data, followed by weekly t-tests comparing the NALS and ALS groups. P-values from weekly t-tests were corrected for multiple comparisons using the Bonferroni method. Detailed statistical results are provided in Supplementary Table 6. Source data are provided in the Source Data file.

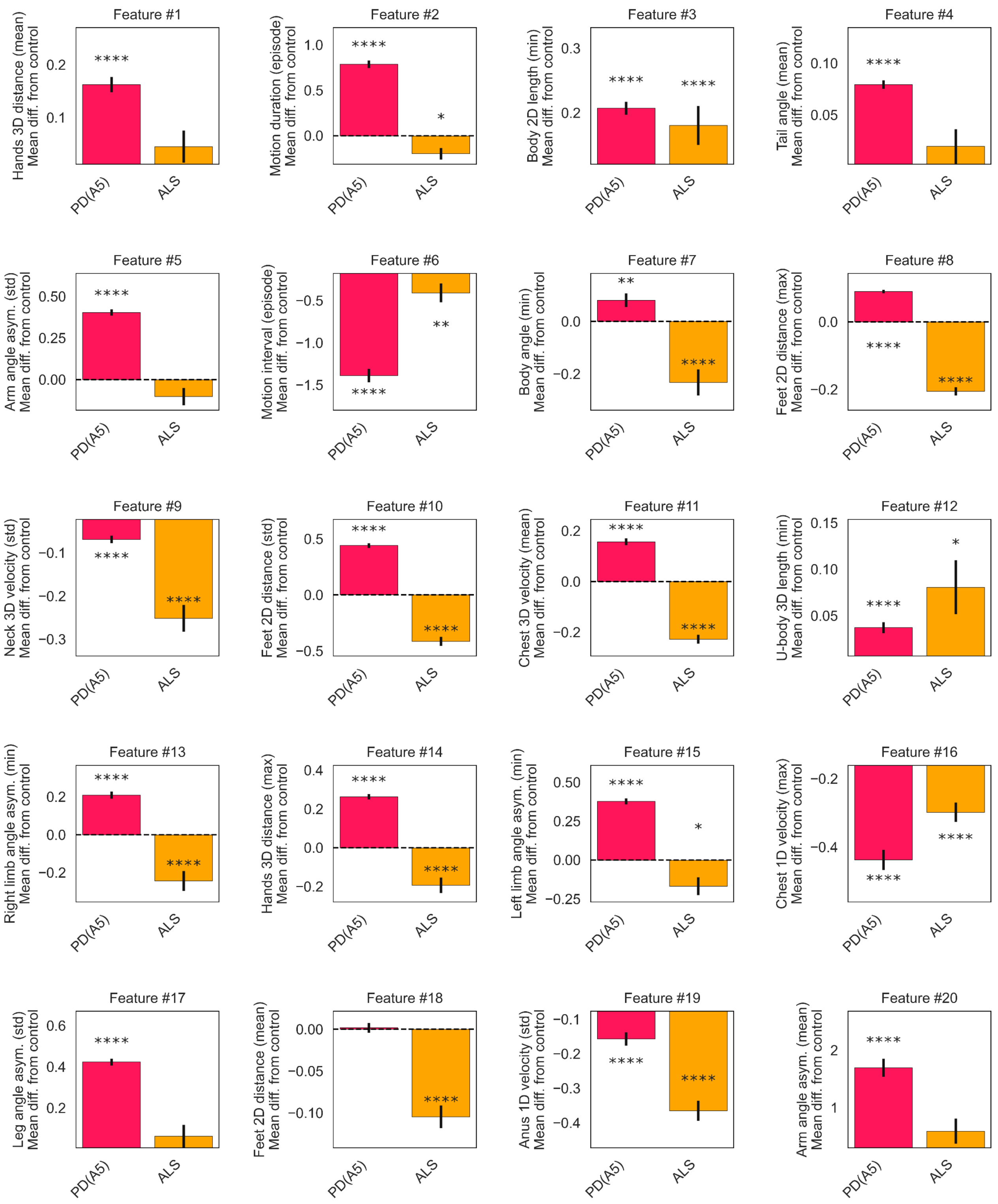

Supplementary Figure 22. Endpoint comparison of PD and ALS groups for the top 20 features identified by the XGB model.

For each feature, differences between the non-disease control and experimental groups (PD: CT vs A5; MND: NALS vs ALS) were compared at their respective experimental endpoints (PD: 10 wk post-surgery; MND: 20 wk of age). Feature values for each group were normalised to their respective baseline means (PD: 0 wk post-surgery; ALS: 10 wk of age). The bar graphs show mean differences between experimental and control groups, with error bars representing SEM differences computed by error propagation. Notably, the direction of changes relative to controls varied significantly between PD and ALS groups in 40% of features (#2, 7, 8, 10, 11, 13, 14, and 15), reflecting distinct symptomatic manifestations between the two diseases. For example, Feature #11 illustrates that PD mice exhibited a more stooped posture compared to non-PD mice, whilst ALS mice displayed a more stretched posture compared to non-ALS mice. Furthermore, fewer features significantly differed from controls in ALS (75%) compared to PD (95%), suggesting that these behavioural features are more sensitive indicators of PD symptoms. Statistical significance between control/experimental pairs is indicated by asterisks, based on Bonferroni-corrected *p*-values. See Supplementary Table 7 for statistical details and the Methods section for further explanation. Source data are provided in Supplementary Tables 4 and 6. Note: This figure compares two distinct datasets; individual-level data points are therefore not available for display.

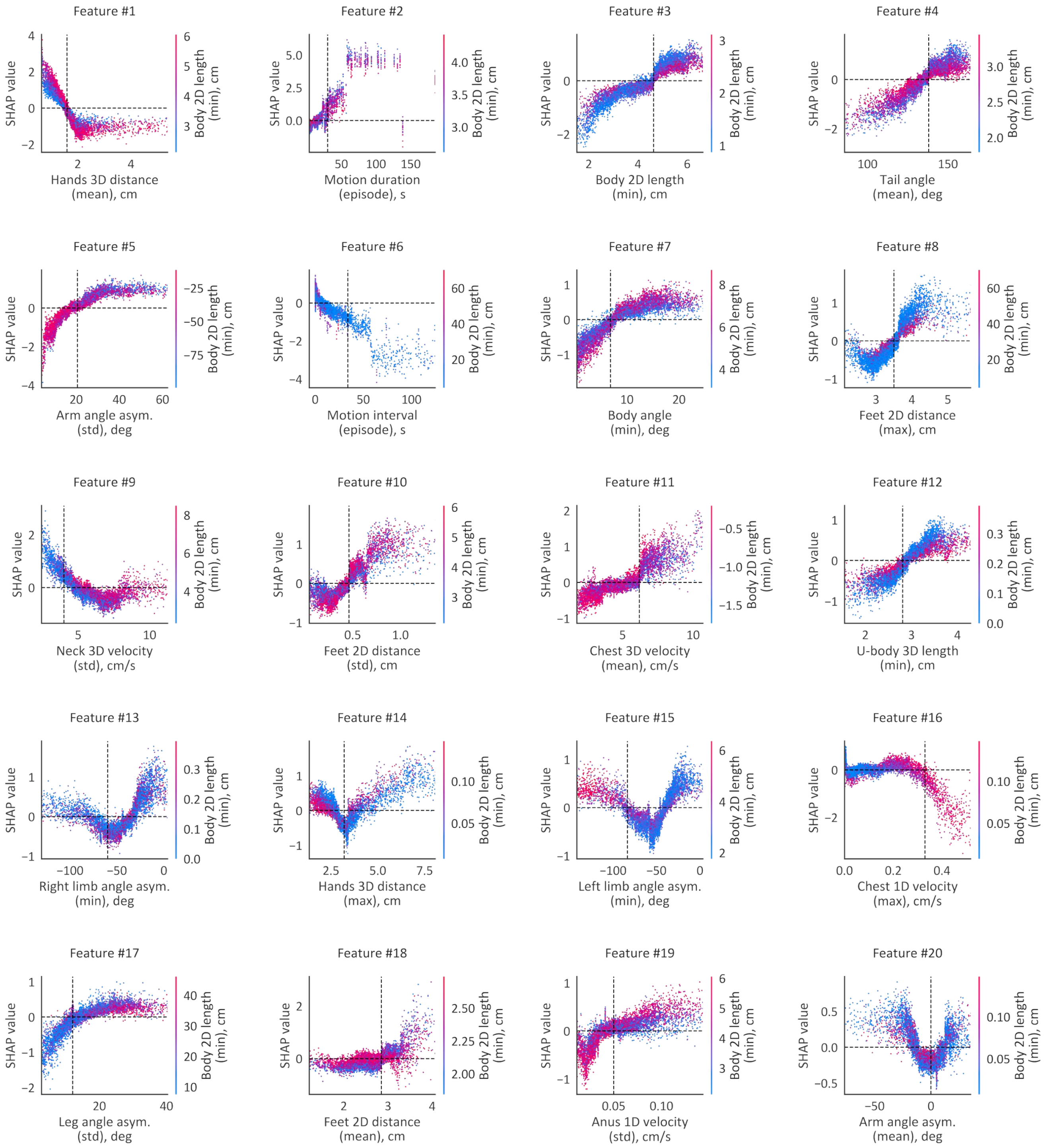

Supplementary Figure 23. SHAP dependence plots of the top 20 features (XGB model).

Each plot displays individual dots representing motion clips that contribute to the model's predictions. The x-axis shows the feature values, limited to the 1st–99th percentiles, whilst the y-axis indicates the SHAP value (log odds of PD likelihood), quantifying the feature's impact on model predictions as non-PD (negative SHAP values) or PD (positive SHAP values). The vertical dashed line intersects the SHAP value at zero, and the horizontal dashed line line intersects the key feature value. Dot colour indicates the value of the interacting feature for each plot is automatically determined by the SHAP library based on the highest correlation with the primary plotted feature's SHAP values. For instance, in subplot #1, the hands 3D distance dependence plot is color-coded by body 2D length (min), demonstrating that smaller hand distances have a greater impact on the prediction when the body length is longer. Similarly, subplot #8 shows that larger feet distance (max) more strongly influences the prediction during shorter motion durations. Linking the colour gradient to specific interacting features visually highlights that the primary feature's predictive influence is context-dependent rather than operating in isolation.

a

	Feature	Unit	Key value	Major range				Inflection range		Minor range			
				NP		PD				NP		PD	
1	Hands 3D distance (mean)	cm	1.60	2	3	0.5	1.7	-	-	-	-	-	-
2	Motion duration	s	30.10	0	22.65	23	23	-	-	-	-	-	-
3	Body 2D distance (min)	cm	4.63	1	3	4.63	6	-	-	-	-	-	-
4	Tail angle (mean)	deg	138.08	90	134	134	170	-	-	-	-	-	-
5	Arm angle asymmetry (std)	deg	20.63	0	10	30	60	-	-	-	-	-	-
6	Motion interval	s	33.95	0	150	4.9	34	-	-	-	-	-	-
7	Body angle (min)	deg	6.88	0	6	7.45	25	-	-	-	-	-	-
8	Feet 2D distance (max)	cm	3.50	2	3.62	3.62	5	2.5	3.5	-	-	1	2.5
9	Neck 3D velocity (std)	cm/s	4.00	4	11	2	4	6	8	-	-	8	12
10	Feet 2D distance (std)	cm	0.46	0	0.5	0.34	1.5	0.2	0.3	-	-	0	0.34
11	Chest 3D velocity (mean)	cm/s	6.19	1	6	6	10	-	-	-	-	-	-
12	Nose-chest 3D distance (min)	cm	2.81	1	3	3	5	-	-	-	-	-	-
13	Right limb angle asymmetry (min)	deg	-60.00	-90	-30	-30	30	-90	30	-	-	-140	-90
14	Hands 3D distance (max)	cm	3.20	2	5	5	7	2.5	4	-	-	1	3
15	Left limb angle asymmetry (min)	deg	-83.65	-80	-30	-30	30	-90	-30	-	-	-15	-85
16	Chest 1D velocity (max)	cm/s	0.33	0.3	0.5	0.1	0.3	0.1	0.4	0	0.2	0	0.05
17	Leg angle asymmetry (std)	deg	12.03	0	11	17	34	-	-	-	-	-	-
18	Feet 2D distance (mean)	cm	2.85	3	4	3	4	3	3	1	3	1	3
19	Anus 1D velocity (std)	cm/s	0.05	0	0.05	0.05	0.15	-	-	-	-	-	-
20	Arm angle asymmetry (mean)	deg	0.00	-5	20	-20	-40	-20	20	-	-	10	40

b

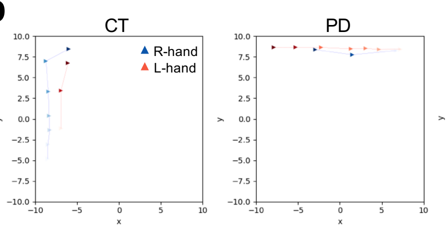

c

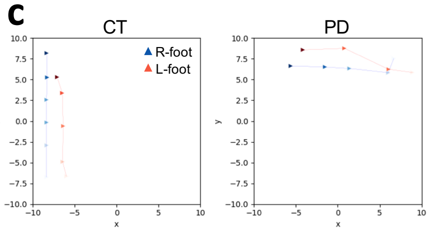

d

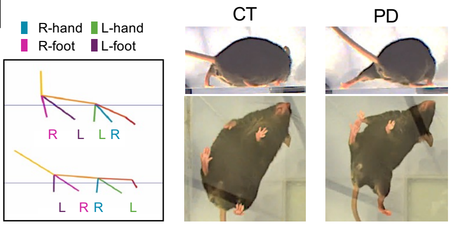

Supplementary Figure 24. Summary table of SHAP dependence plots for the top 20 features (XGB model).

(a) Key feature values are identified by analysing feature dependence plots and node split conditions within the decision trees. Key values near a SHAP value of zero indicate points where the feature's impact on model predictions shifts direction. The major feature value range is defined by clusters of high data density or high magnitude of absolute SHAP values for the data. For some features, a minor range with an inflection point is revealed through SHAP analysis, which otherwise remains unveiled. **(b, c)** Representative trajectories of the hands and feet in CT and PD mice. **(d)** Example skeleton images highlighting extreme limb angle asymmetries (Feature #13, 15), alongside snapshot images from CT and PD mice.

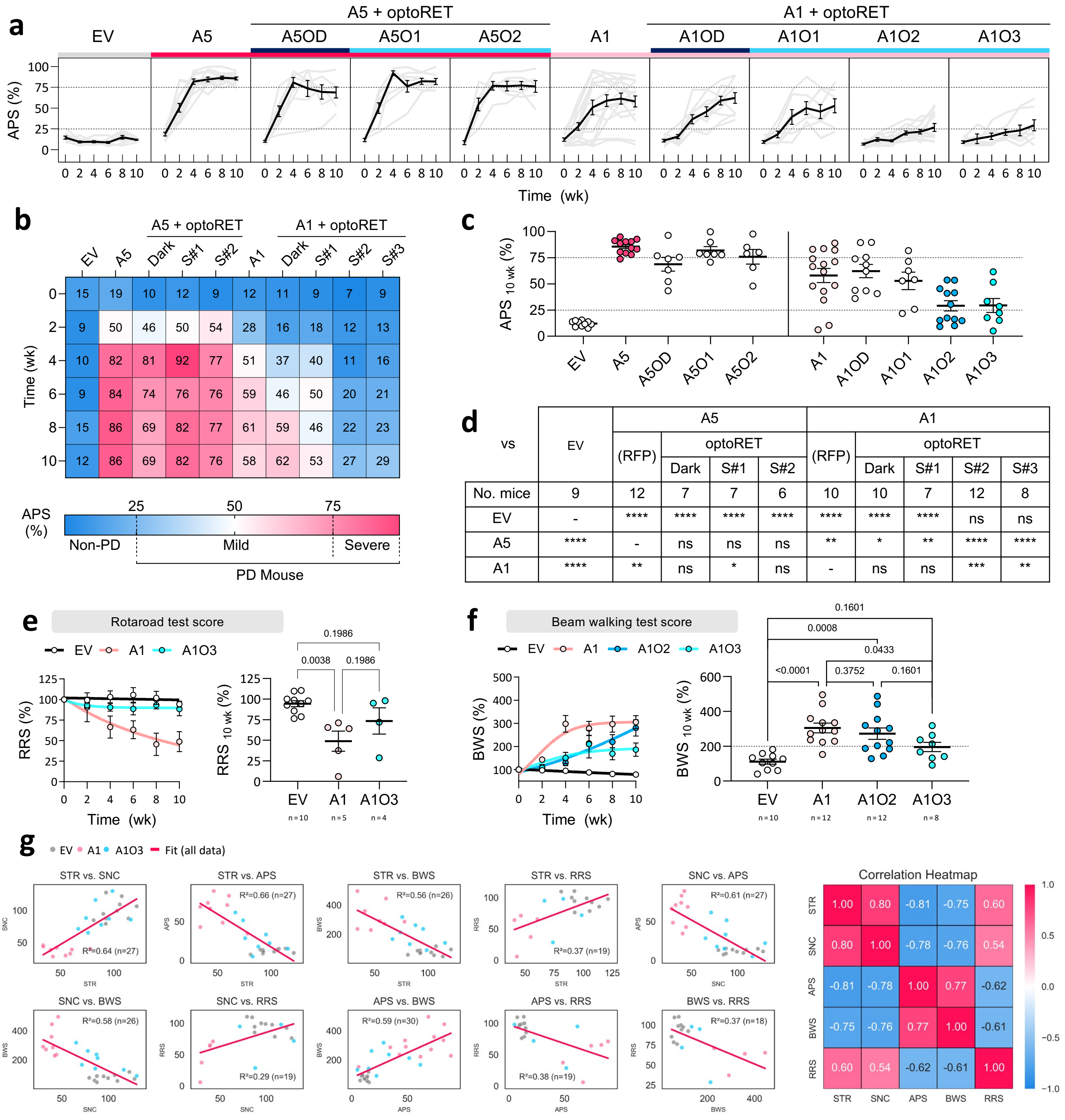

vs	EV	A5				A1				
		(RFP)	optoRET			(RFP)	optoRET			
			Dark	S#1	S#2		Dark	S#1	S#2	S#3
No. mice	9	12	7	7	6	10	10	7	12	8
EV	-	****	****	****	****	****	****	****	ns	ns
A5	****	-	ns	ns	ns	**	*	**	****	****
A1	****	**	ns	*	ns	-	ns	ns	***	**

Supplementary Figure 25. Behavioural assessments (RRS, BWS, and APS) in PD mice treated with optogenetic stimulation (optoRET).

(a) Line graphs of APS for each mouse group over time. The black line represents mean ± SEM of the group, and the grey lines depict individual mice in the group. Groups with optoRET were unstimulated (dark) or stimulated with the pre-set long-term light schedules: daily (S#1), biweekly (S#2), or alternate days (S#3). Dashed lines on the graphs indicate *health status* categories: non-PD (0–25), mild PD (25–75), and severe PD (>75). **(b)** Heatmap visualization of panel (a) displays temporal trends in PD scores, showing the group mean at each time point. **(c)** Dot plots of APS for each group at 10 wk, detailed with mean ± SEM. **(d)** Statistical summary of multiple comparisons using one-way ANOVA followed by Bonferroni's multiple comparisons test. This analysis, based on the APS (XGB model-predicted PD scores) at 10 wk shown in panel (c), demonstrates the therapeutic efficacy of optoRET under various light schedules. Detailed statistical results are provided in Supplementary Table 8. **(e, f)** RRS and BWS comparisons between the EV, A1 and opto-treated groups, illustrated in line and dot graphs for the longitudinal (0-10 wk) and the endpoint (10 wk) analysis (RRS* [%]: EV = 94.40 ± 3.67, A1 = 48.84 ± 12.20, A1O3 = 73.42 ± 16.01; BWS [%]: EV = 78.12 ± 6.66, A1 = 305.0 ± 27.90, A1O2 = 272.1 ± 33.11, A1O3 = 195.4 ± 26.76). One-way ANOVA and post-hoc tests with Holm–Sidak multiple comparison corrections were performed. **(g)** Dot plots (with fitted regression lines in red) showing pairs of metrics and heatmap illustrating Pearson's correlation coefficients. For each correlation, datasets (CT and PD groups) were filtered based on each metric pair. Source data are provided in the Source Data file.

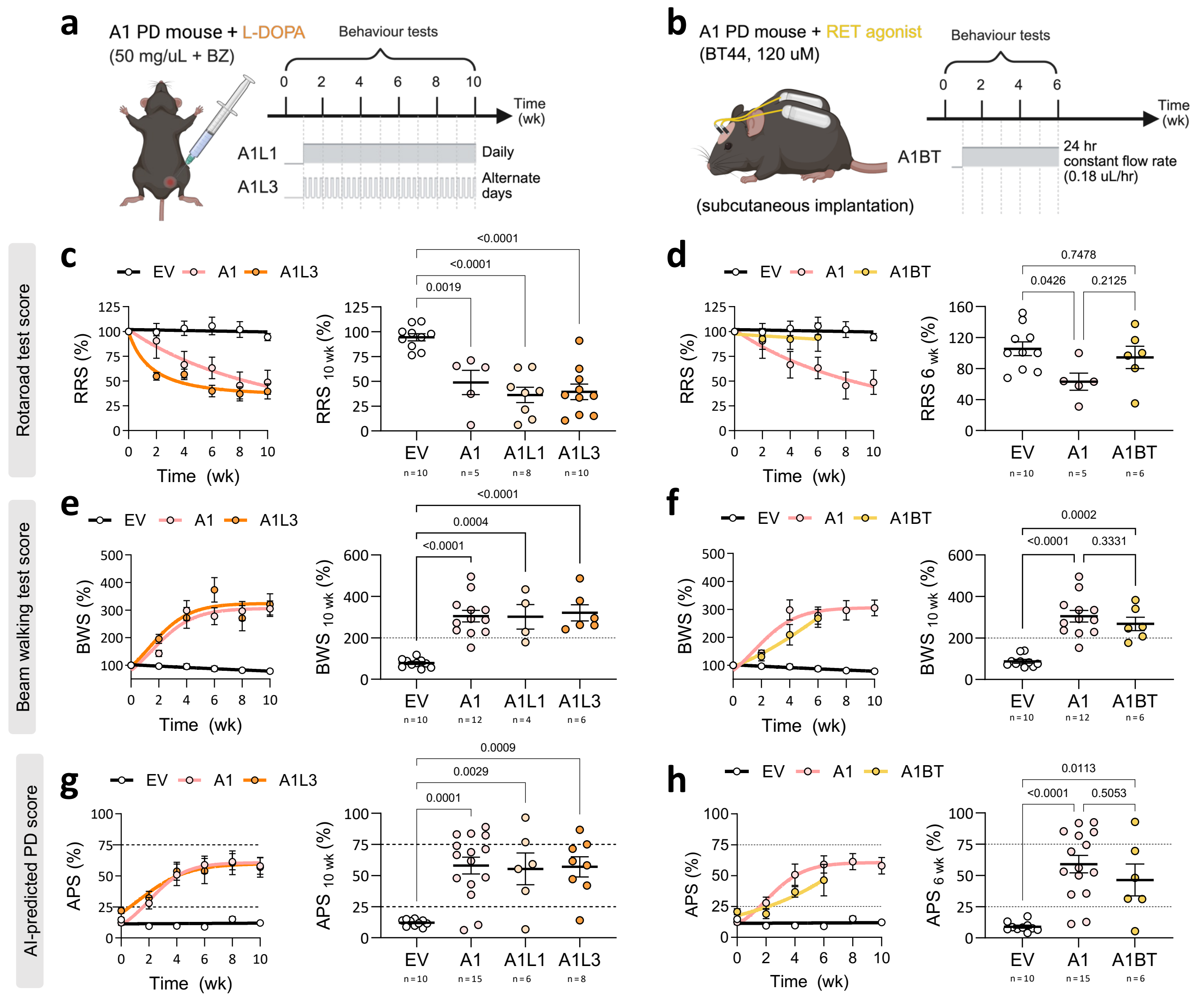

i

Endpoint	Treatment	Group	Mean +/- SEM (n)			*P*-value (compared to EV)			*P*-value (compared to A1)		
			RRS (%)	BWS (%)	APS (%)	RRS	BWS	APS	RRS	BWS	APS
10 wk	-	EV	94.40 ± 3.67 (10)	78.12 ± 6.66 (10)	12.11 ± 0.85 (10)	-	-	-	-	-	-
	-	A1	48.84 ± 12.20 (5)	305.0 ± 27.90 (12)	58.01 ± 6.69 (15)	0.0019	<0.0001	<0.0001	-	-	-
	optoRET	A1O2	-	272.1 ± 33.11 (12)		-	0.0008	0.1322	-	0.3752	0.0018
		A1O3	73.42 ± 16.01 (4)	195.4 ± 26.76 (8)		0.1986	0.1601	0.1322	0.1986	0.0433	0.0062
	L-DOPA	A1L1	36.31 ± 7.78 (8)	301.8 ± 59.20 (4)	55.38 ± 12.71 (6)	<0.0001	0.0004	0.0029	0.6679	0.9742	0.9643
		A1L3	39.44 ± 7.74 (10)	320.9 ± 38.76 (6)	56.98 ± 8.03 (8)	<0.0001	<0.0001	0.0009	0.6679	0.9742	0.9643
6 wk	RET agonist (BT44)	EV	105.5 ± 8.83 (10)	87.46 ± 8.88 (10)	8.95 ± 1.19 (10)	-	-	-	-	-	-
		A1	63.14 ± 11.02 (5)	305.0 ± 27.90 (12)	59.04 ± 6.95 (15)	0.0426	<0.0001	<0.0001	-	-	-
		A1BT	94.42 ± 14.29 (6)	267.9 ± 32.21 (6)	46.38 ± 12.70 (6)	0.7478	0.0002	0.0113	0.2125	0.3331	0.5053

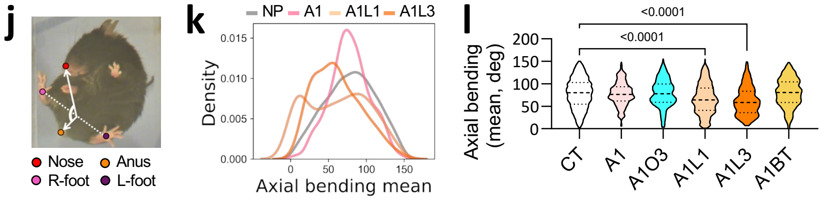

m

	CT	A1	A1O3	A1L1	A1L3	A1BT
n mouse	50	15	8	8	10	6
n clip	1385	367	270	1981	323	472
%	27.7	24.5	33.75	247.6	32.3	78.7
Mean	78.86	77.83	78.90	66.42	61.92	80.14
SEM	0.8900	1.353	1.832	0.7481	1.744	1.433

Supplementary Figure 26. Behavioural assessments (RRS, BWS, and APS) in A1 PD mice treated with L-DOPA or RET agonist (BT44).

(a, b) Schematic illustration of A1 mice treated with either L-DOPA or RET agonist (BT44). L-DOPA with Benserazide (BZ) was administered by intraperitoneal injection every day (A1L1) or alternate days (A1L3). BT44 was administered by direct brain infusion into the striatum at a constant flow rate (A1BT). In the latter group, the experimental endpoint was adjusted to 6 wk due to the limited volume of the osmotic pumps implanted. **(b-h)** Comparisons of behavioural assessments (RRS, BWS and APS) among A1 mice treated with L-DOPA or BT44, illustrated in line and dot graphs for the longitudinal and the endpoint. **(i)** Summary table of the behavioural assessments in A1 mice with optogenetic, L-DOPA or BT44 treatments. Significant *p*-values are highlighted. **(j)** Schematic illustration of axial bending angle measurement (the angle formed by the two vectors [arrows]). **(k)** KDE plots showing mean axial bending angles derived from threshold-filtered datasets (see Methods) for CT, A1, and L-DOPA groups. **(l, m)** Comparisons of axial bending angle across groups. Detailed interpretations of the BT44 and L-DOPA treatment groups are provided in Supplementary Notes 7 and 8, respectively. All statistics, one-way ANOVA and post-hoc tests with Holm–Sidak multiple comparison corrections were performed. Source data are provided in the Source Data file. Schematics were created in BioRender. Heo, W. (2025) https://BioRender.com/cykyauz.

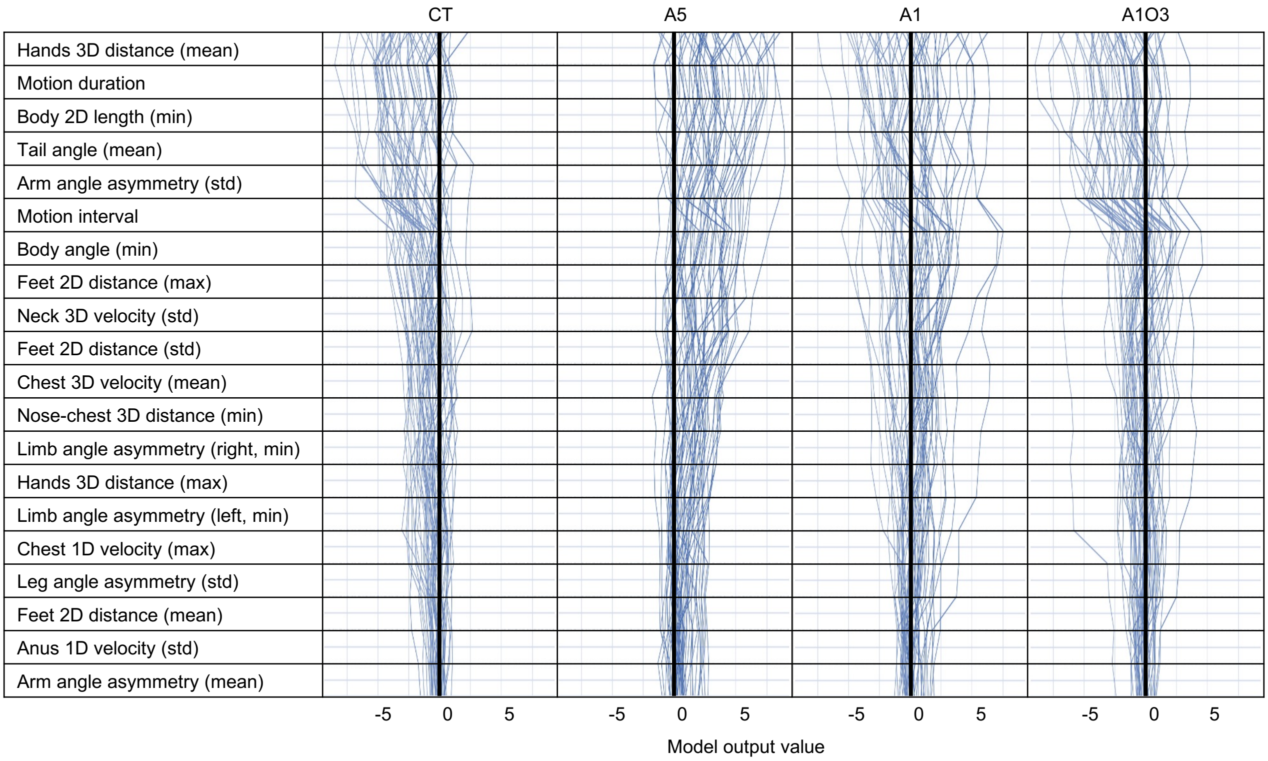

Supplementary Figure 27. Group comparison of the decision tree plots (XGB model) at 10 wk.

For the CT, A5, A1 and A1O3 groups, 50 clips from each group are randomly selected from the tested dataset at 10 wk and their decision plots are plotted. The top 20 features are only shown, in the order of the model's feature importance rank. The proportions of the sampled clips predicted as PD (model output value > 0) were 12% (6/50), 90% (5/50), 44% (22/50) and 16% (8/50) for the respective groups.

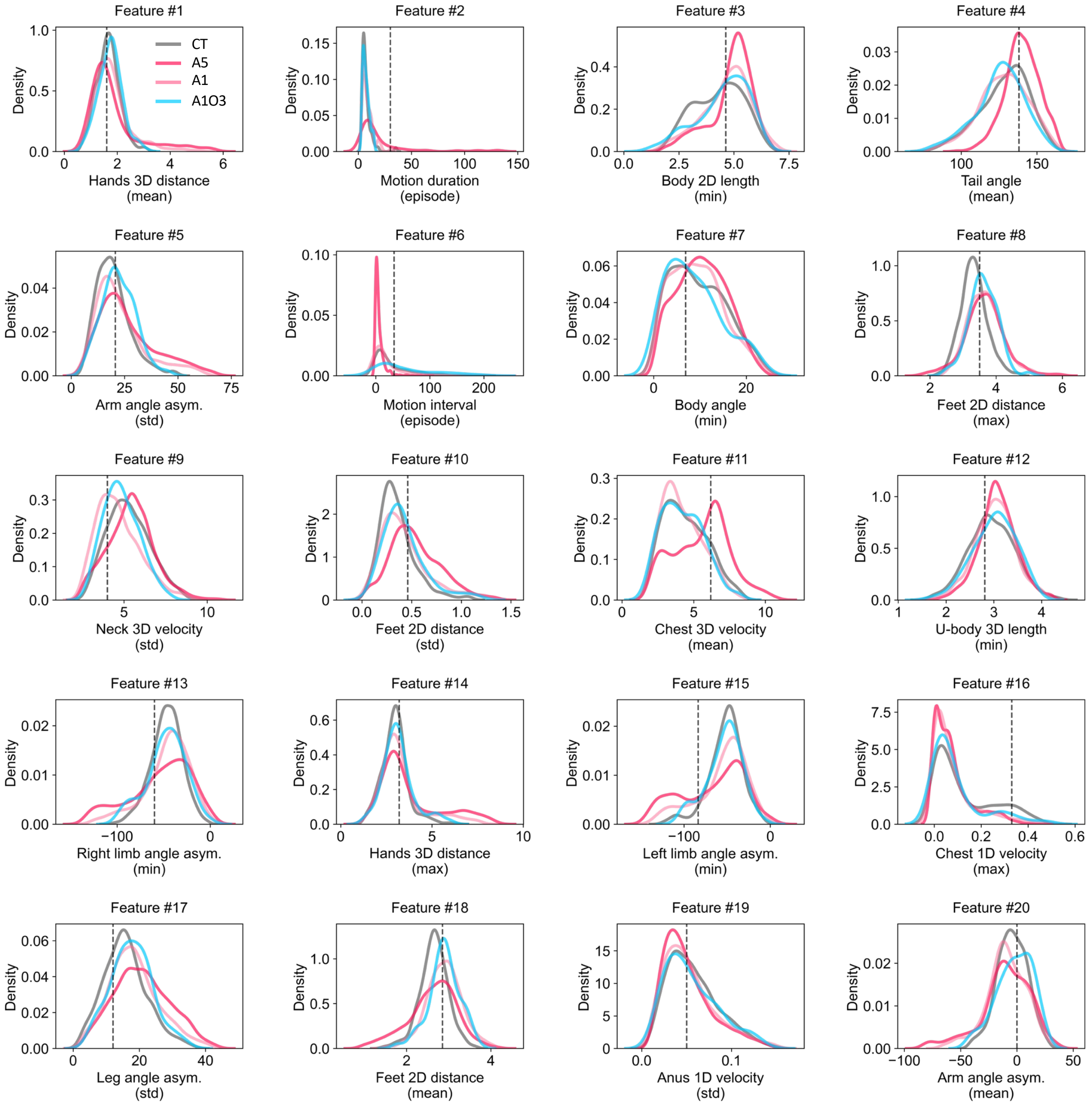

Supplementary Figure 28. Group comparison of the top 20 features (XGB model) in the KDE plots at 10 wk.

Kernel density estimate (KDE) plots for each feature compare distribution patterns across the CT, A5, A1, and A1O3 groups at 10 wk, with vertical dashed lines highlighting key feature values.

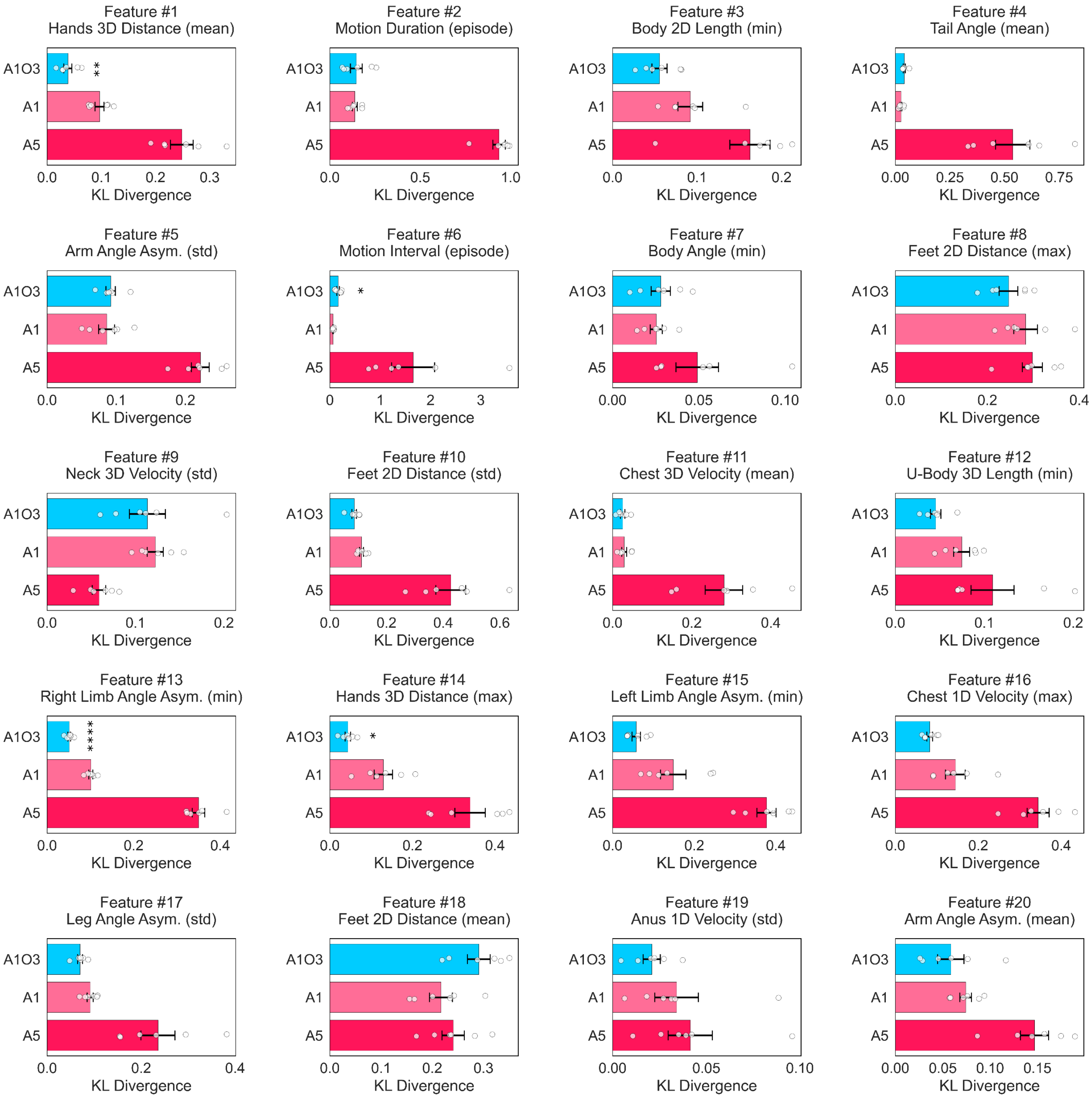

Supplementary Figure 29. Comparison of KL divergences of the top 20 features (XGB model) between groups at 10 wk.

For each group, the KL divergence of each feature from the CT counterpart was computed at 10 wk, and the results are shown as bar graphs (mean ± SEM). Each group consists of 6 sets of the sampling datasets, each comprising 150 randomly selected clips. When comparing the magnitude of the differences to CT, the A1O3 group showed less significant differences for 7 features, more significant differences for 4 features, and similar differences for 9 features compared to the A1 group. In addition, for 4 features, the divergence in the A1O3 group differed significantly from that of the A1 group. See Supplementary Table 9 for detailed statistical results. All statistics, one-way ANOVA and post-hoc tests with Holm–Sidak multiple comparison corrections were performed. Source data are provided in the Source Data file.

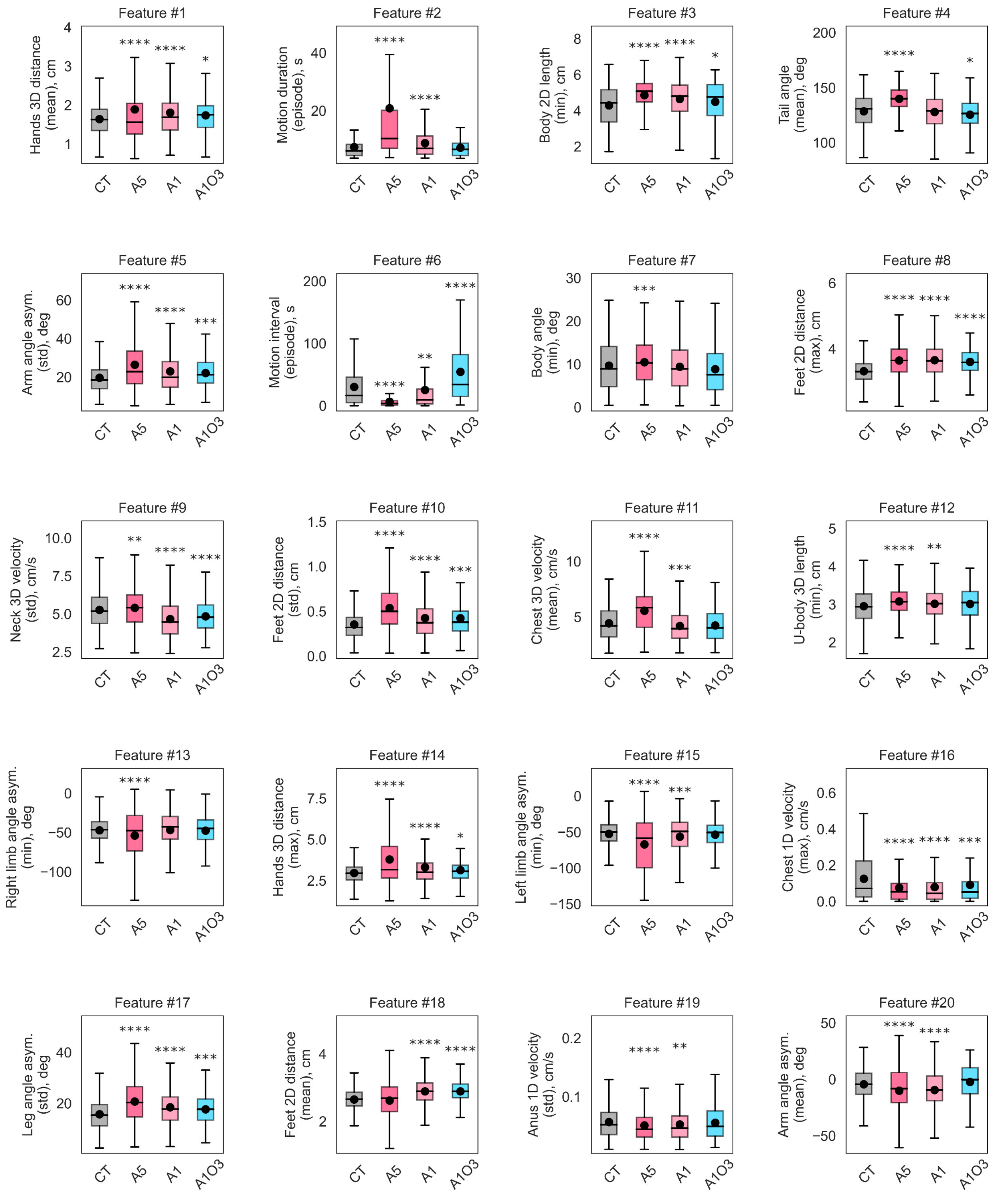

Supplementary Figure 30. Group comparisons of the feature values (XGB model) at 10 wk.

Box plots show the median (black line), mean (black circle), and interquartile range (box). Whiskers extend to the most extreme data points within 1.5 × the interquartile range from the lower and upper quartiles (Tukey method). Individual outliers beyond this range are omitted from the plot for clarity but are retained in the dataset and included in the calculation of the mean and median. Statistical analysis was performed by one-way ANOVA followed by Holm–Sidak-corrected Welch's t-tests comparing each group to CT. Significant differences are indicated by asterisks. Exact p-values and sample sizes are provided in Supplementary Table 10. Source data are provided in the Source Data file.

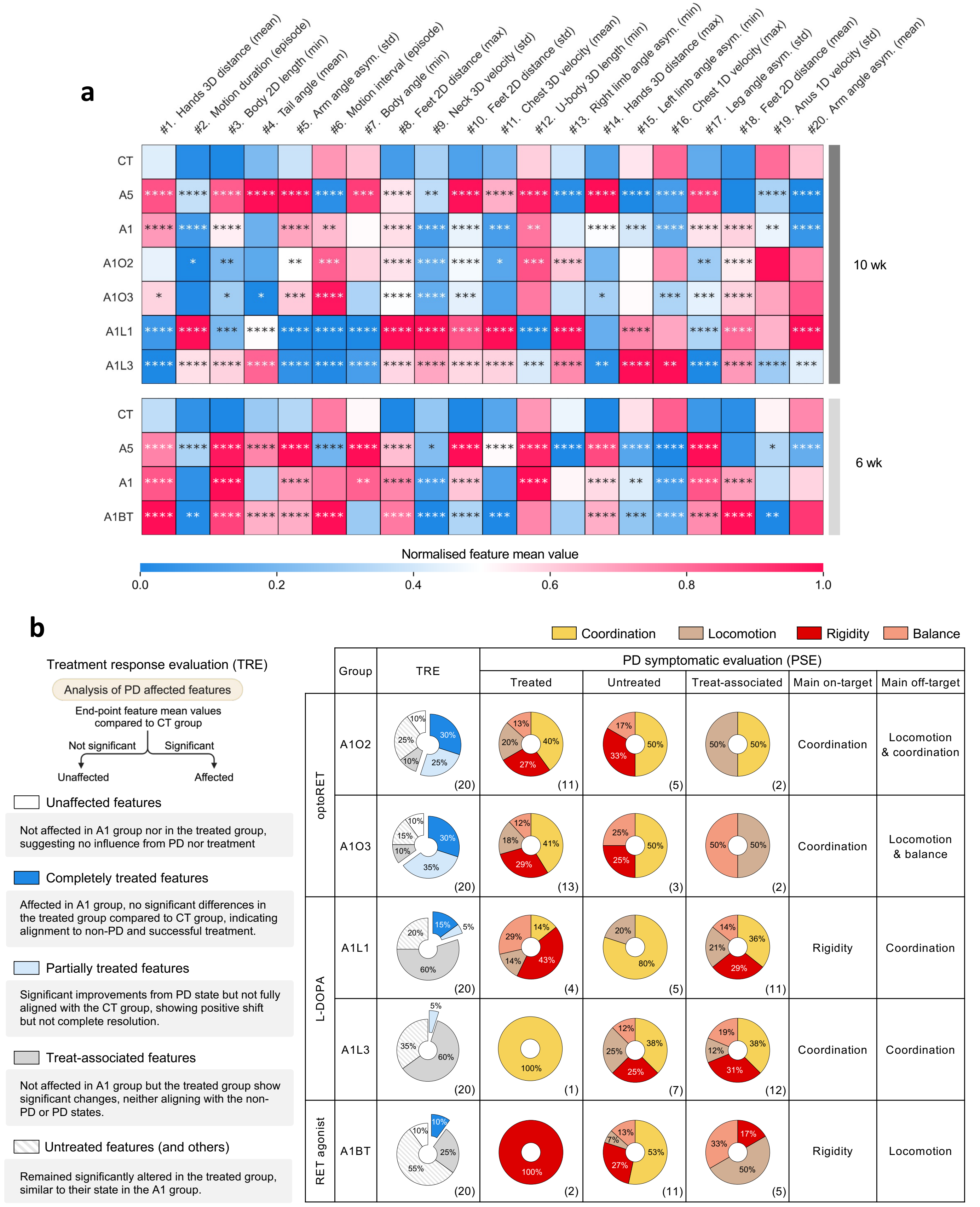

Supplementary Figure 31. Comparisons of treatment response and PD symptomatic evaluations in A1 PD mice with different treatments.

(a) Heatmaps of the mean feature values for each group at endpoint. Statistical analysis was performed using one-way ANOVA, followed by post-hoc comparisons using Welch's t-tests comparing each group to the reference (CT) group. Statistical significance is indicated by asterisks after Holm–Sidak correction for multiple comparisons. Detailed statistical results are provided in Supplementary Tables 10–15. **(b)** Comparisons of three treatment groups for the treatment response evaluation (TRE) and PD symptomatic evaluation (PSE) at 10 wk, except for the A1BT group (assessed at 6 wk). The descriptions of each TRE category are shown on the left, and the feature numbers included for each evaluation are indicated in brackets.

		Node velocity (cm/s)	Pair distance (cm)		Pair angle difference (rad)
Parent features from XGB model		Neck	nose-chest	Body	rleg-lleg
		Anus	neck-anus	Tail	rleg-rarm
		Chest	chest-anus		rleg-larm
		Tip	rfoot-lfoot		rarm-larm
			rhand-lhand		lleg-larm
					lleg-rarm
Feature transform	Velocity (cm/s)	N/A	No	Yes	No
	Acceleration (cm/s^{-2})	Yes	No	No	No
Sub-total (core features)	23 features created				

TSFEL feature extraction	Statistical domain	Spectral domain	Temporal domain
	Absolute energy	FFT mean coefficient	Area under the curve
	Average power	Fundamental frequency	Autocorrelation
	ECDF	Human range energy	Centroid
	ECDF Percentile	LPCC	Mean absolute diff
	ECDF Percentile Count	MFCC	Mean diff
	Entropy	Max power spectrum	Median absolute diff
	Histogram	Maximum frequency	Median diff
	Interquartile range	Median frequency	Negative turning points
	Kurtosis	Power bandwidth	Peak to peak distance
	Max	Spectral centroid	Positive turning points
	Mean	Spectral decrease	Signal distance
	Mean absolute deviation	Spectral distance	Slope
	Median	Spectral entropy	Sum absolute diff
	Median absolute deviation	Spectral kurtosis	Zero crossing rate
	Min	Spectral positive turning points	Neighbourhood peaks
	Root mean square	Spectral roll-off	
	Skewness	Spectral roll-on	
	Standard deviation	Spectral skewness	
	Variance	Spectral slope	
		Spectral spread	
		Spectral variation	
		Wavelet absolute mean	
		Wavelet energy	
		Wavelet standard deviation	
		Wavelet entropy	
		Wavelet variance	
Total	4485 features created		
AutoML feature selection	332 features selected		

Supplementary Figure 32. Summary table of the features engineered and extracted for the spectro-temporal analysis.

Using the parent features from XGB model, total 23 kinematic features in 3D were created as a list of core features. By implementing TSFEL (Time Series Feature Extraction Library), total 4485 features from statistical, spectral and temporal domains, were created. Total 332 of them were selected through an automated ML processes using PyCaret library, as the key features for PD behaviour phenotyping.

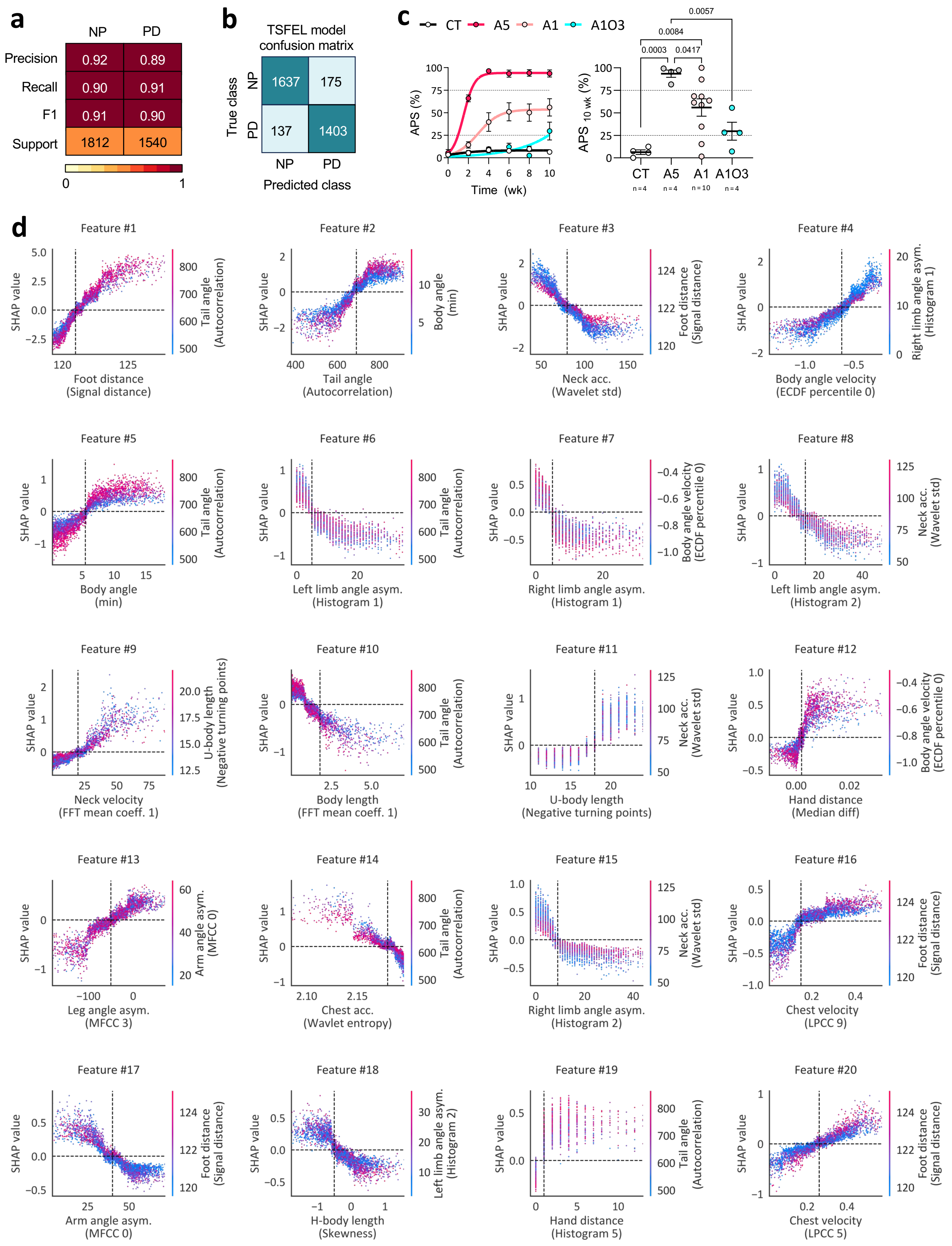

Supplementary Figure 33. Model evaluation and SHAP dependence plots of top 20 features (TSFEL model).

(a) Summary of the TSFEL model evaluation metrics. **(b)** Confusion matrix of the TSFEL model in the classification of validation dataset. **(c)** TSFEL model predicted PD scores for the CT, A5, A1 and A1O3 groups (n = 4, 4, 10 and 4, respectively). **(d)** SHAP dependence plots of the top 20 features (TSFEL model). See Supplementary Figure 23 legend for more detailed description on SHAP dependence plots. Statistical analyses was performed using one-way ANOVA followed by Holm–Sidak post hoc corrections for multiple comparisons. Source data are provided in the Source Data file.

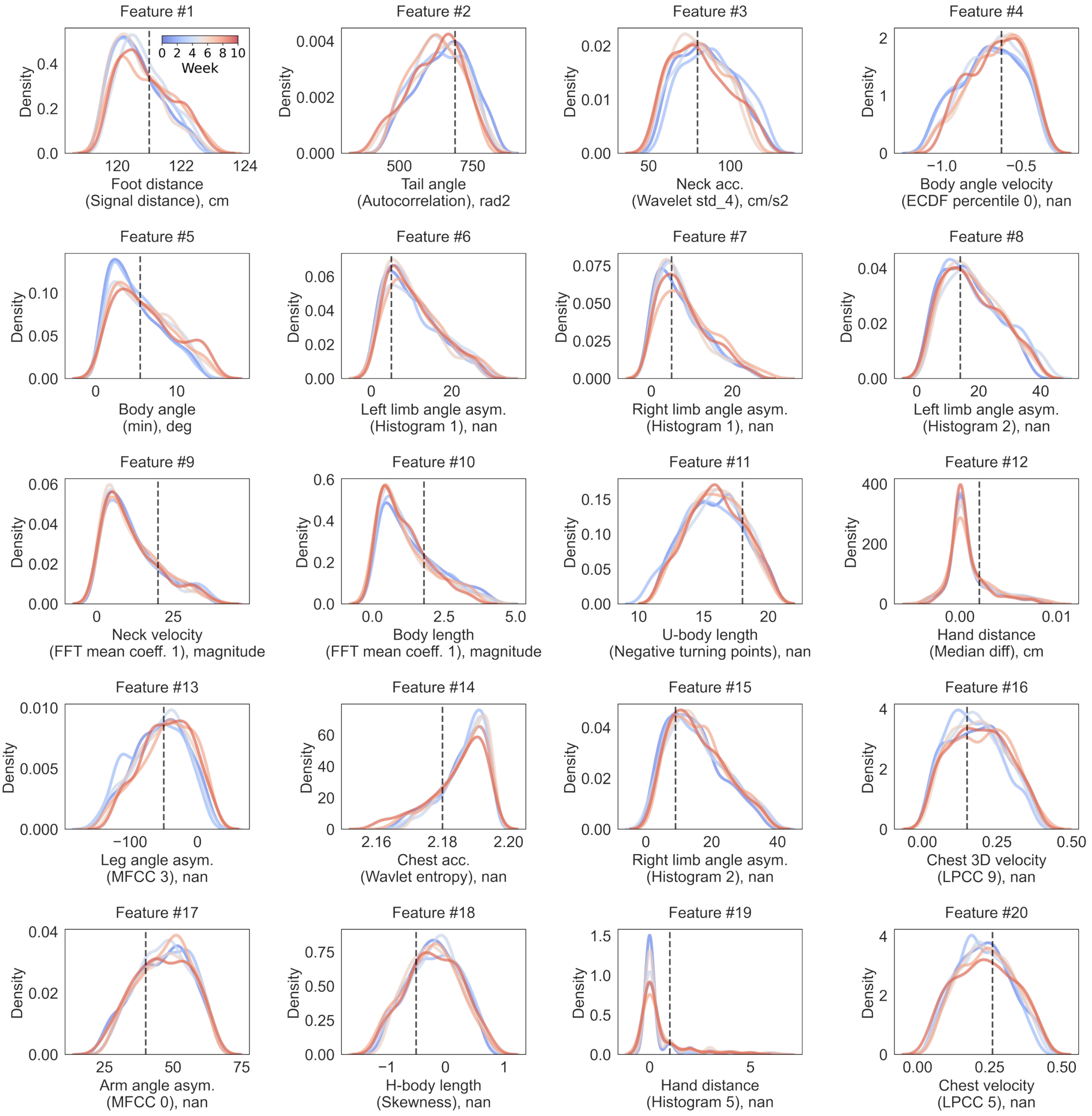

Supplementary Figure 34. CT group's longitudinal analysis of the top 20 features (TSFEL model) in the KDE plots.

For each feature, KDE plots of each week (0, 2, 4, 6, 8 and 10 wk) were plotted. Vertical dashed lines indicate the key values.

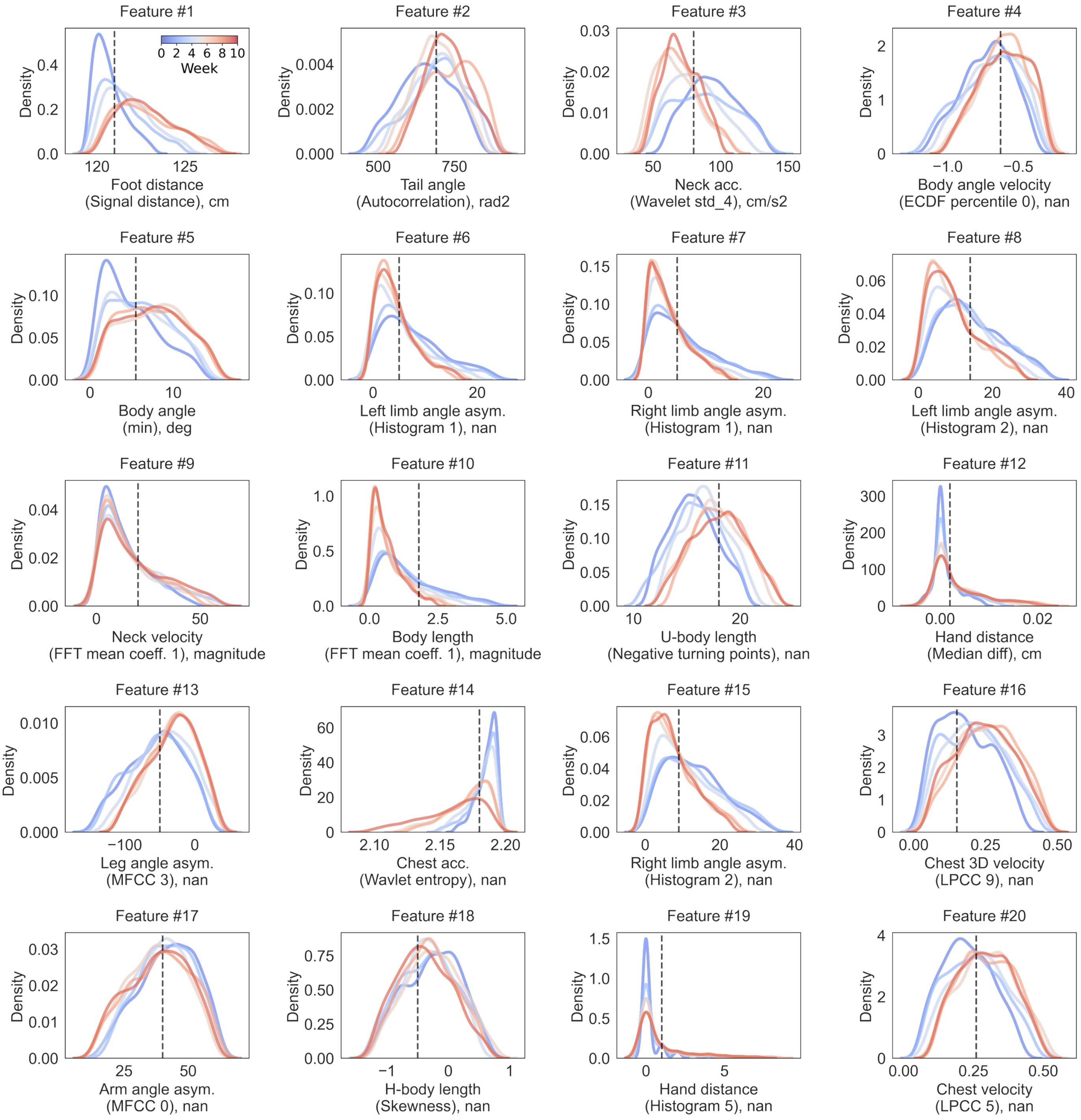

Supplementary Figure 35. PD (A5) group's longitudinal analysis of the top 20 features (TSFEL model) in the KDE plots.

For each feature, KDE plots of each week (0, 2, 4, 6, 8 and 10 wk) were plotted. Vertical dashed lines indicate the key values.

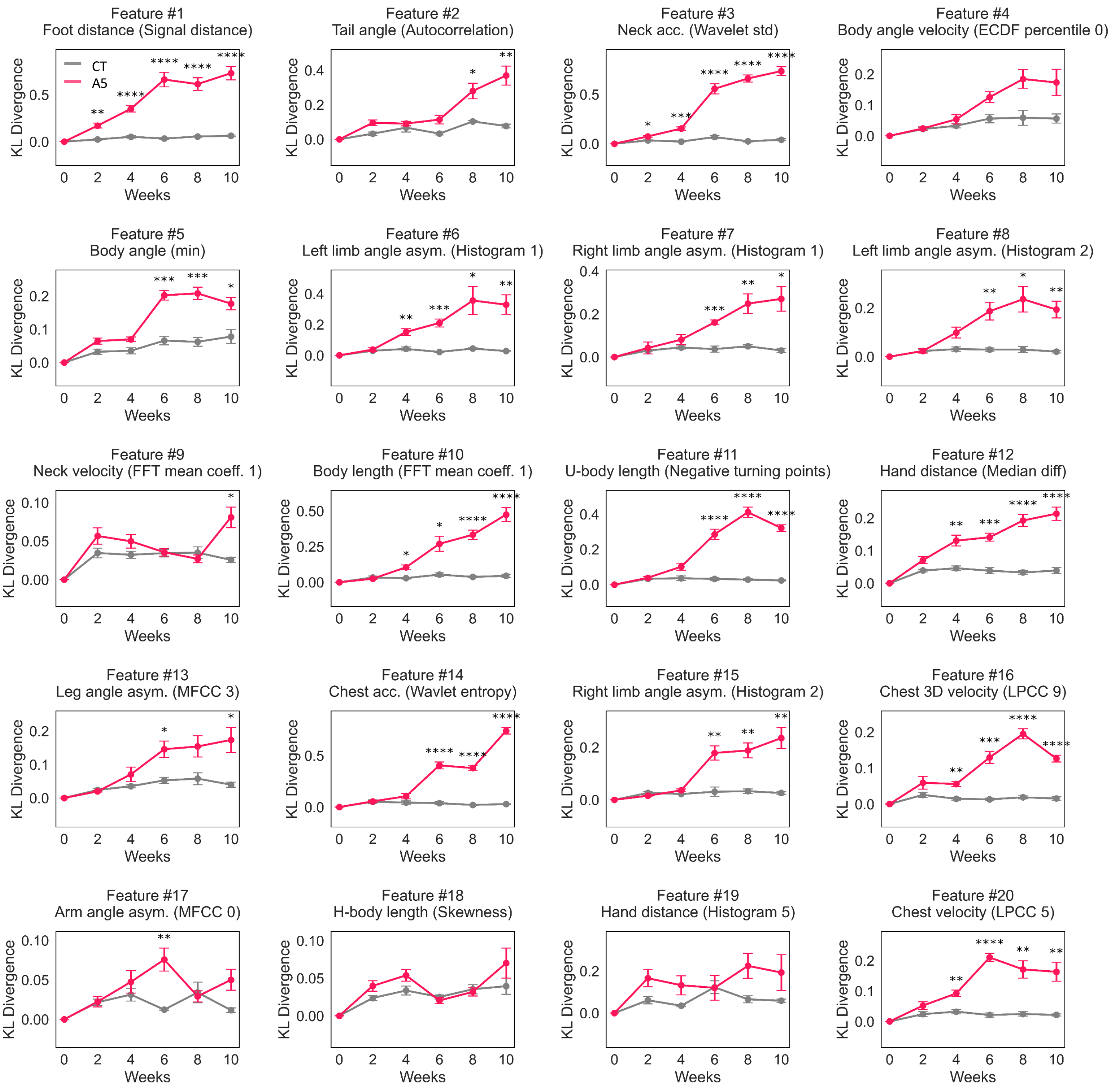

Supplementary Figure 36. Longitudinal comparison of KL divergences (TSFEL model) between the CT and PD (A5) groups.

For each group, the KL divergences of each feature from the respective baseline (0 wk) were computed over the experimental duration (0-10 wk) and plotted as line graphs (mean ± SEM. For each group, the KL divergence of each feature relative to its baseline value (0 wk) was computed across the experimental duration (0–10 wk). Each group consisted of 6 sampling sets, each containing 150 randomly selected clips. A two-way ANOVA (group × week) was performed for each feature, followed by weekly t-tests between groups (CT vs. A5). The resulting p-values from the weekly t-tests were corrected for multiple comparisons using the Bonferroni method. Detailed statistical results are provided in Supplementary Table 16. Source data are provided in the Source Data file.

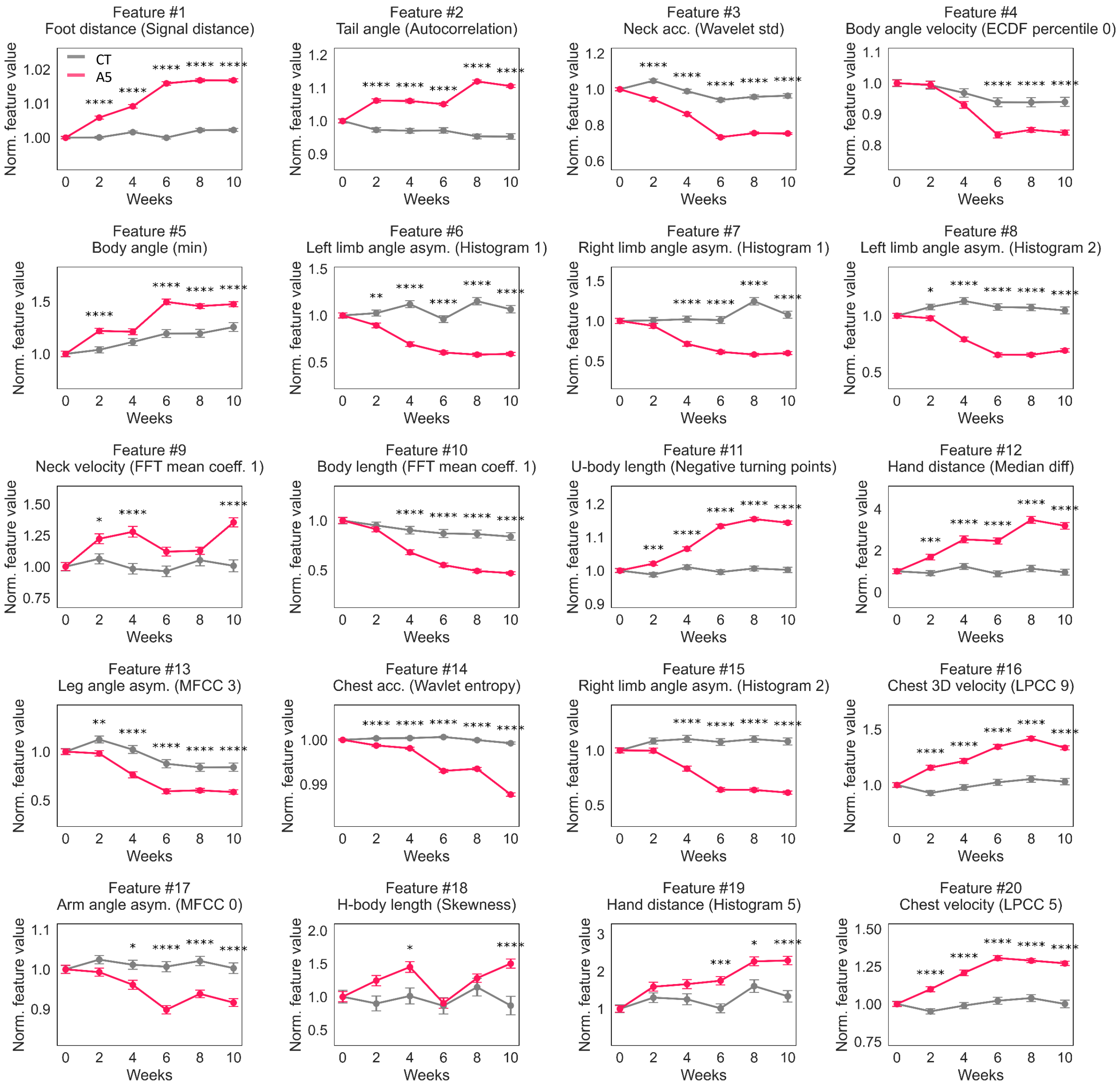

Supplementary Figure 37. Longitudinal comparison of feature values (TSFEL model) between the CT and PD (A5) groups.

For each group, the KL divergences of each feature from the respective baseline (0 wk) were computed over the experimental duration (0-10 wk) and plotted as line graphs (mean ± SEM) over time. For each feature, the values at each week were divided by the group's corresponding baseline (0 wk) mean value, and the normalised data were analysed over the experimental duration (0–10 wk). Each group consisted of 6 sampling sets, each containing 150 randomly selected clips. Two-way ANOVA (group × week) was performed for each feature on the normalised data, followed by weekly t-tests comparing the CT and A5 groups. P-values from weekly t-tests were corrected for multiple comparisons using the Bonferroni method. Detailed statistical results are provided in Supplementary Table 17. Source data are provided in the Source Data file.

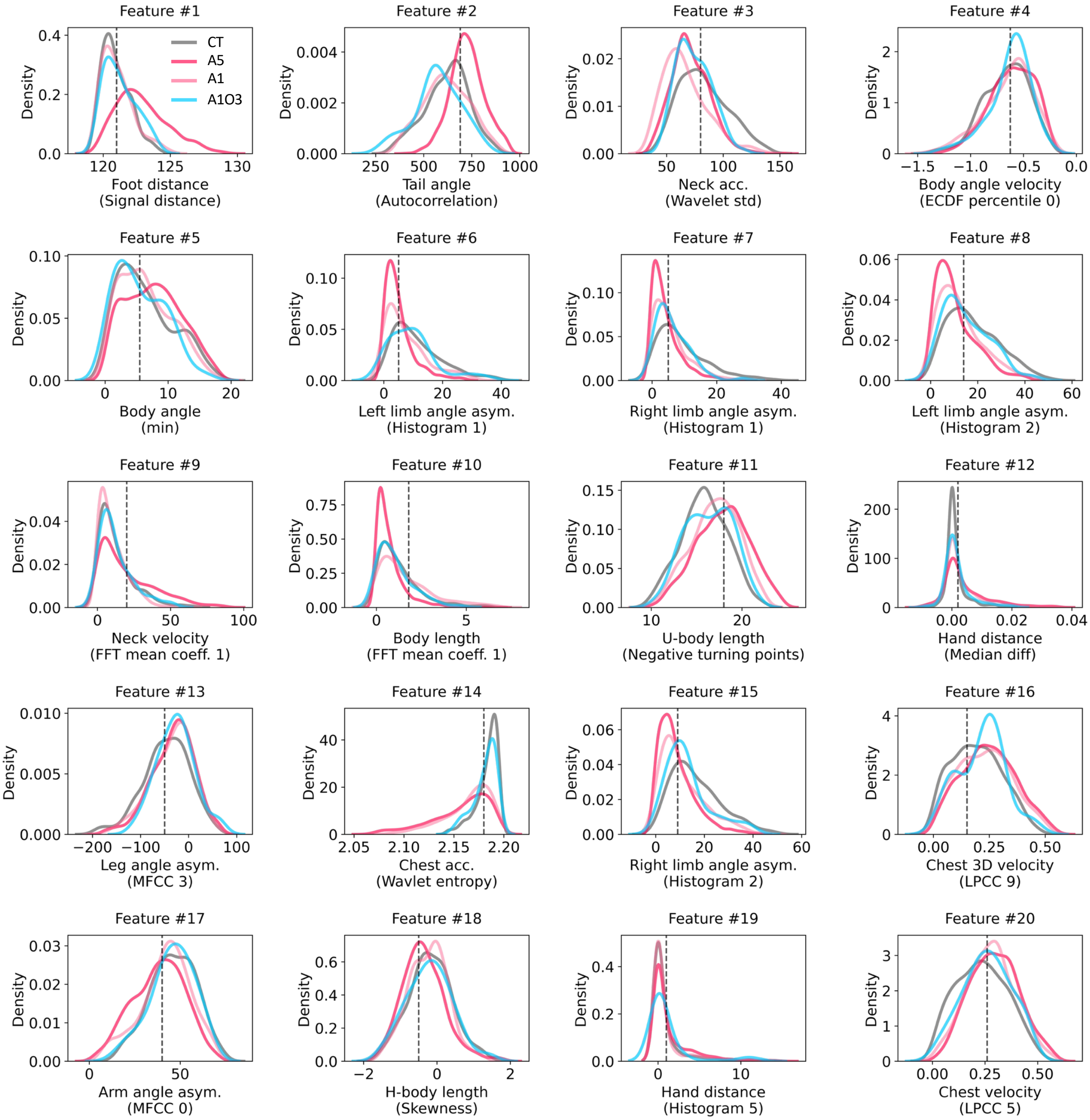

Supplementary Figure 38. Group comparison of the KDE plots of the top 20 features (TSFEL model) at 10 wk.

For each feature, KDE plots of the CT, A5, A1 and A1O3 groups were plotted. Vertical dashed lines indicate the key values.

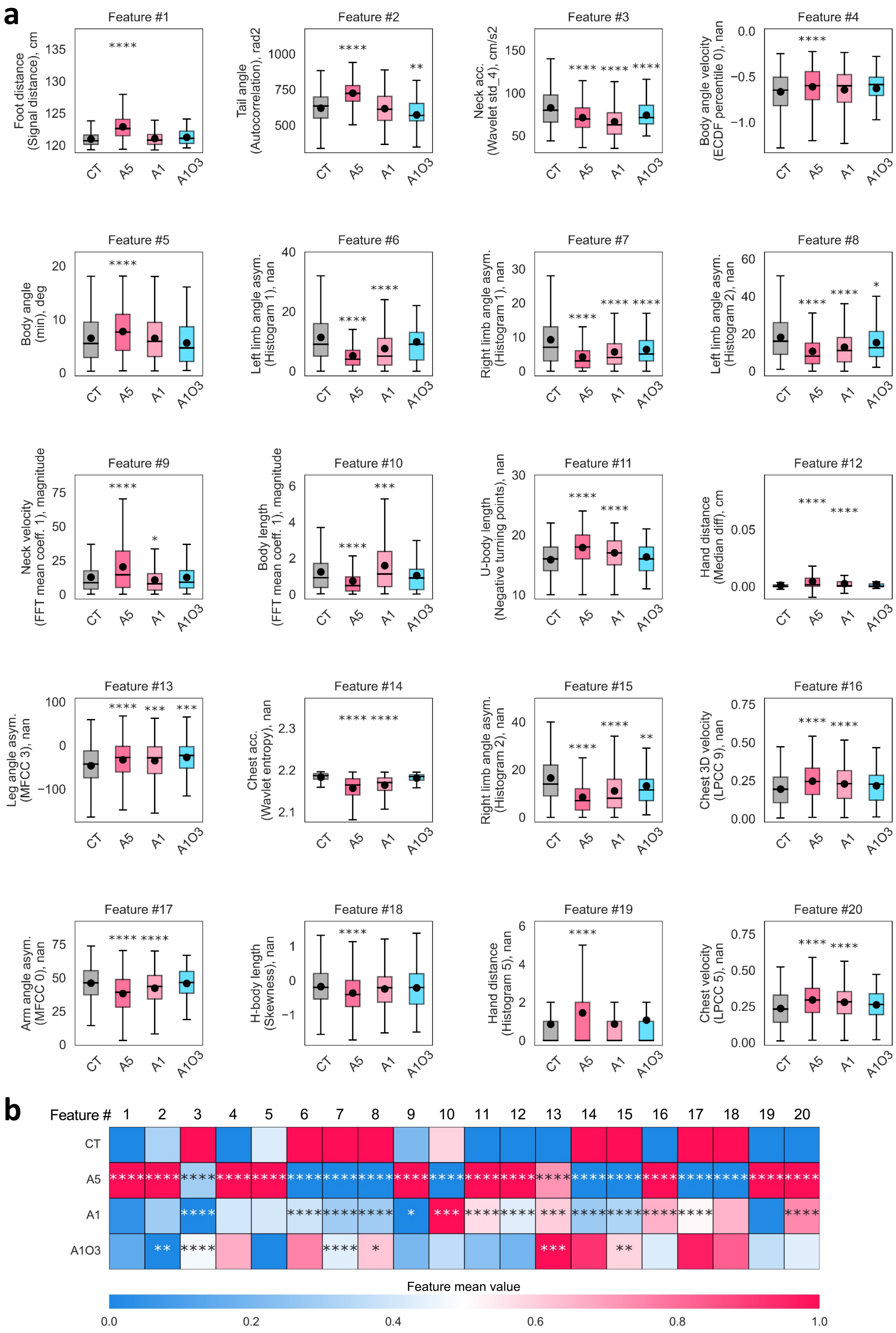

Supplementary Figure 39. Group comparison of the top 20 features (TSFEL model) at 10 wk.

(a) Box plots show the median (black line), mean (black circle), and interquartile range (box). Whiskers extend to the most extreme data points within 1.5 × the interquartile range from the lower and upper quartiles (Tukey method). Individual outliers beyond this range are omitted from the plot for clarity but are retained in the dataset and included in the calculation of the mean and median. The same data for Feature #14, #3 and #2 are also presented in panels e, h and k of Figure 4, respectively. **(b)** Heatmaps of the mean feature values for each group at 10 wk. Statistical analysis was performed by one-way ANOVA followed by Holm–Sidak-corrected Welch's t-tests comparing each group to CT. Significant differences are indicated by asterisks. Exact p-values and sample sizes are provided in Supplementary Table 18. Source data are provided in the Source Data file.

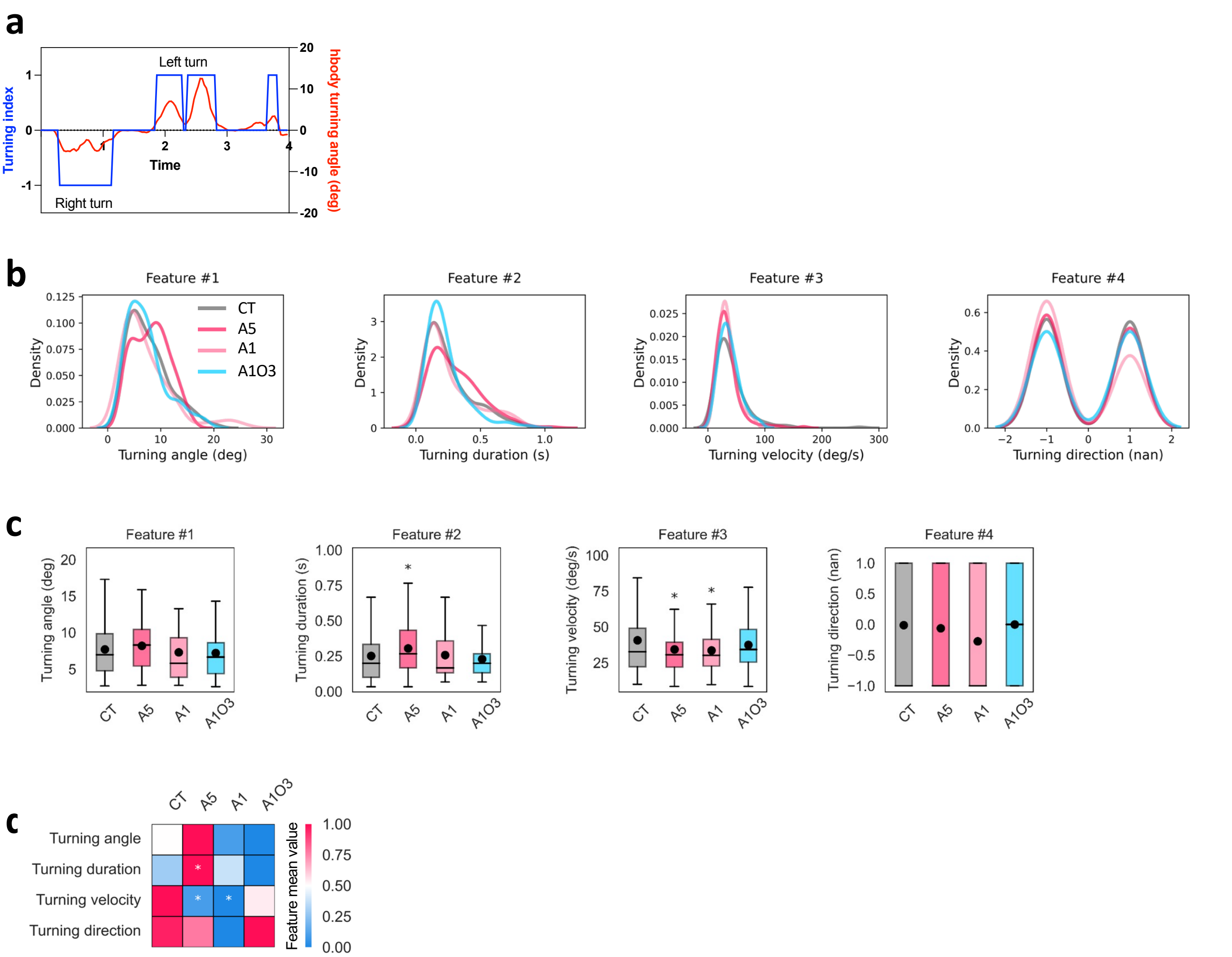

Supplementary Figure 40. Group comparison of the turning features at 10 wk.

(a) Detection of turning events based on the hind-body (hbody) angle. **(b)** For each feature, KDE plots of the CT, A5, A1 and A1O3 groups were plotted. **(c)** Box plots show the median (black line), mean (black circle), and interquartile range (box). Whiskers extend to the most extreme data points within 1.5 × the interquartile range from the lower and upper quartiles (Tukey method). Individual outliers beyond this range are omitted from the plot for clarity but are retained in the dataset and included in the calculation of the mean and median. The same data for Feature #2 and #3 are also presented in panels c and d of Figure 5, respectively. **(d)** Heatmaps of the mean feature values for each group at 10 wk. Statistical analysis was performed by one-way ANOVA followed by Benjamini–Hochberg corrected Welch's t-tests comparing to CT. Significant differences are indicated by asterisks. Exact p-values and sample sizes are provided in Supplementary Table 19. Source data are provided in the Source Data file.

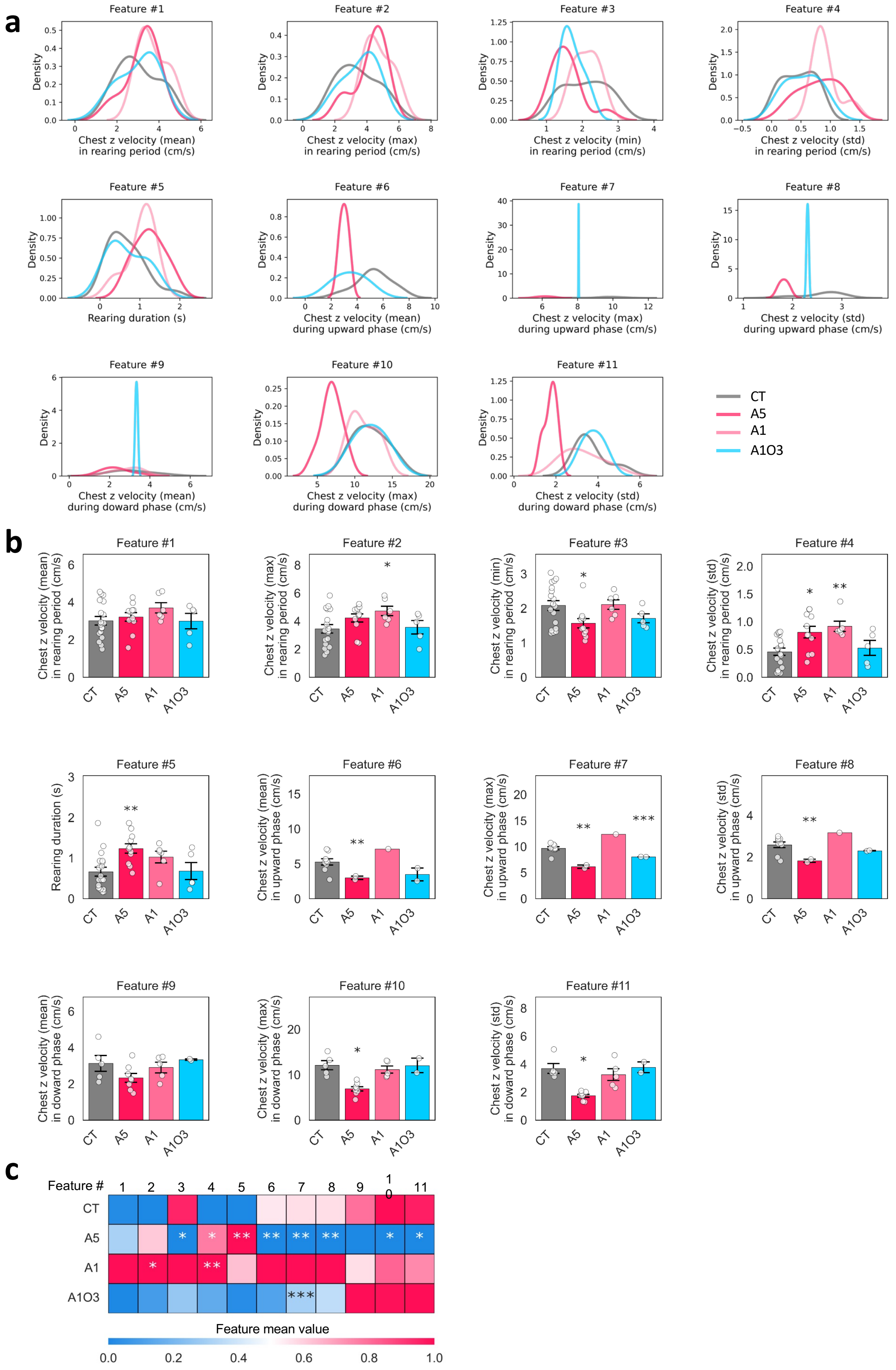

Supplementary Figure 41. Group comparison of the rearing features at 10 wk.

(a) For each feature, KDE plots of the Normal, A5X, A1X and A1X+S#3 groups were plotted. **(b)** Data for each feature are shown as bar graphs (mean ± SEM). The same data for Feature #4 is also presented in panel e of Figure 5.**(c)** Heatmaps of the mean feature values for each group at endpoint. Statistical analysis was performed using one-way ANOVA, followed by post-hoc comparisons using Welch's t-tests comparing each group to the reference (CT) group. Statistical significance is indicated by asterisks after Holm–Sidak correction for multiple comparisons. Detailed statistical results are provided in Supplementary Table 20. Source data are provided in the Source Data file.

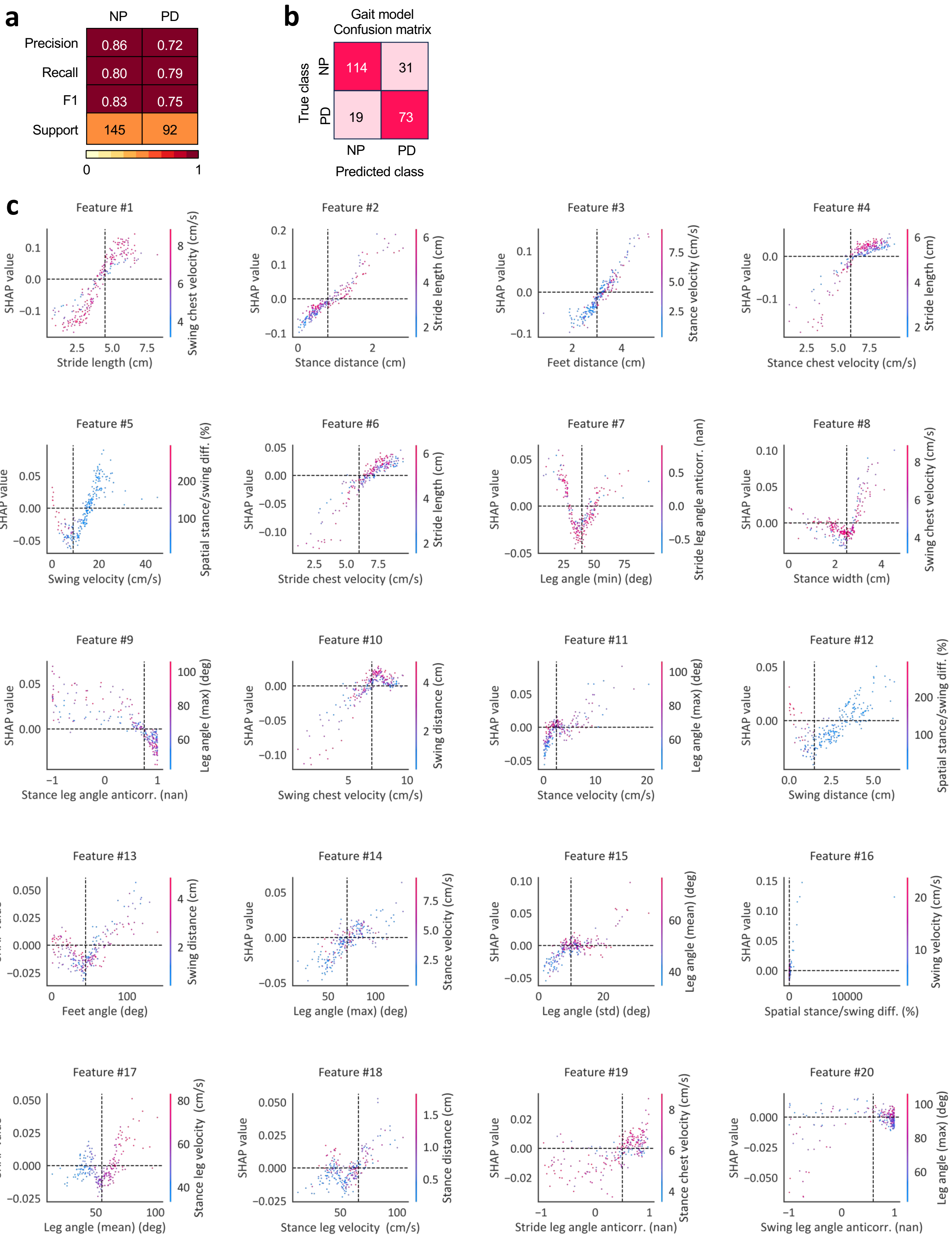

Supplementary Figure 42. Model evaluation and SHAP dependence plots of top 20 features (Gait model).

(a) Summary of the Gait model evaluation metrics. **(b)** Confusion matrix of the Gait model in the classification of validation dataset. **(c)** SHAP dependence plots of the top 20 features (Gait model). See Supplementary Figure 23 legend for more detailed description on SHAP dependence plots.

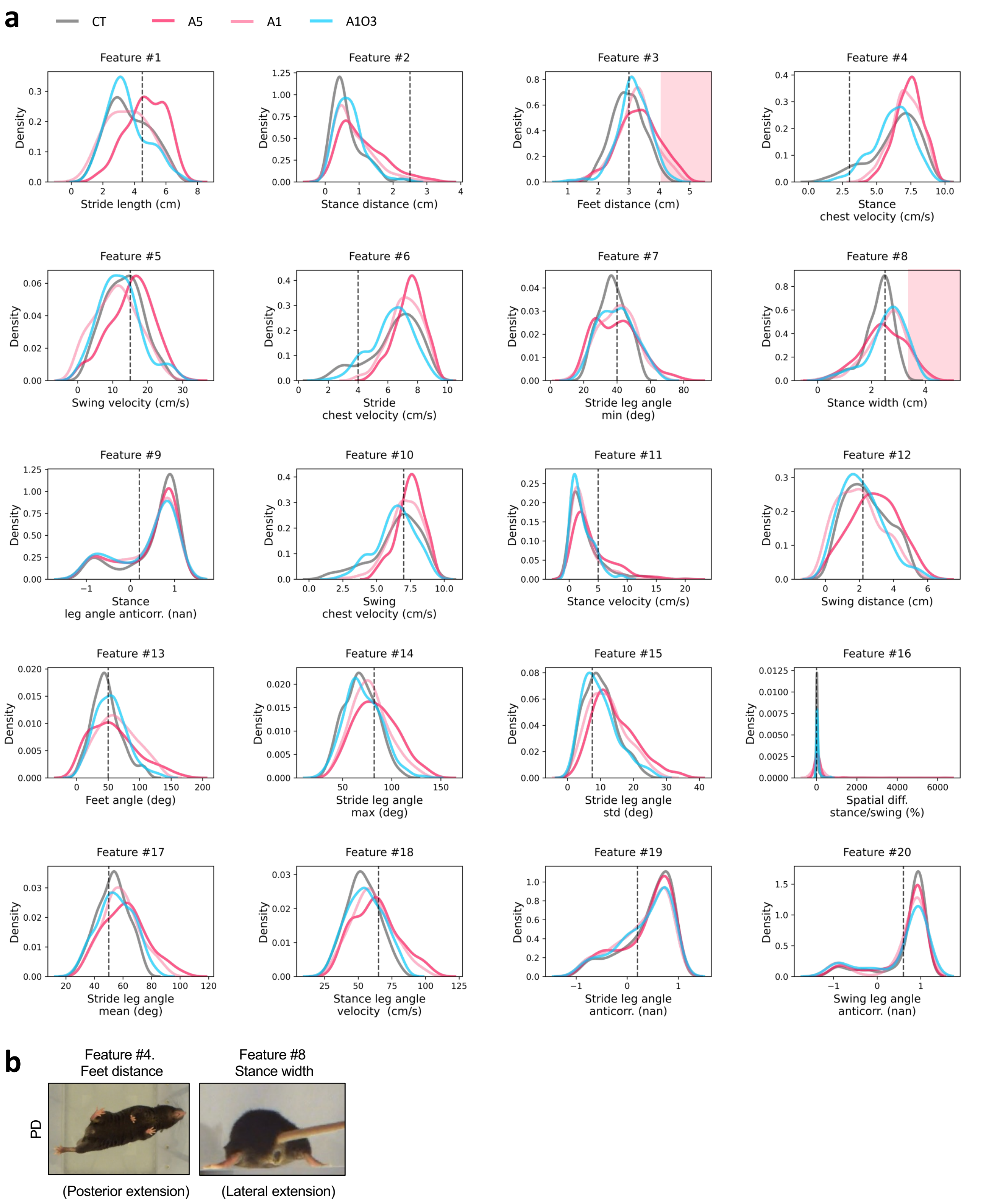

Supplementary Figure 43. Group comparison of the top 20 features (Gait model) in KDE plots at 10 wk.

(a) Kernel density estimate (KDE) plots for each feature compare distribution patterns across the CT, A5, A1, and A1O3 groups at 10 wk, with vertical dashed lines highlighting key feature values. The pink background in features #3 and #8 highlights the subpopulation of PD mice exhibiting abnormally high feet distance and stance width. **(b)** Snapshot video images capture instances of abnormally high feet distance and stance width observed in PD mice.

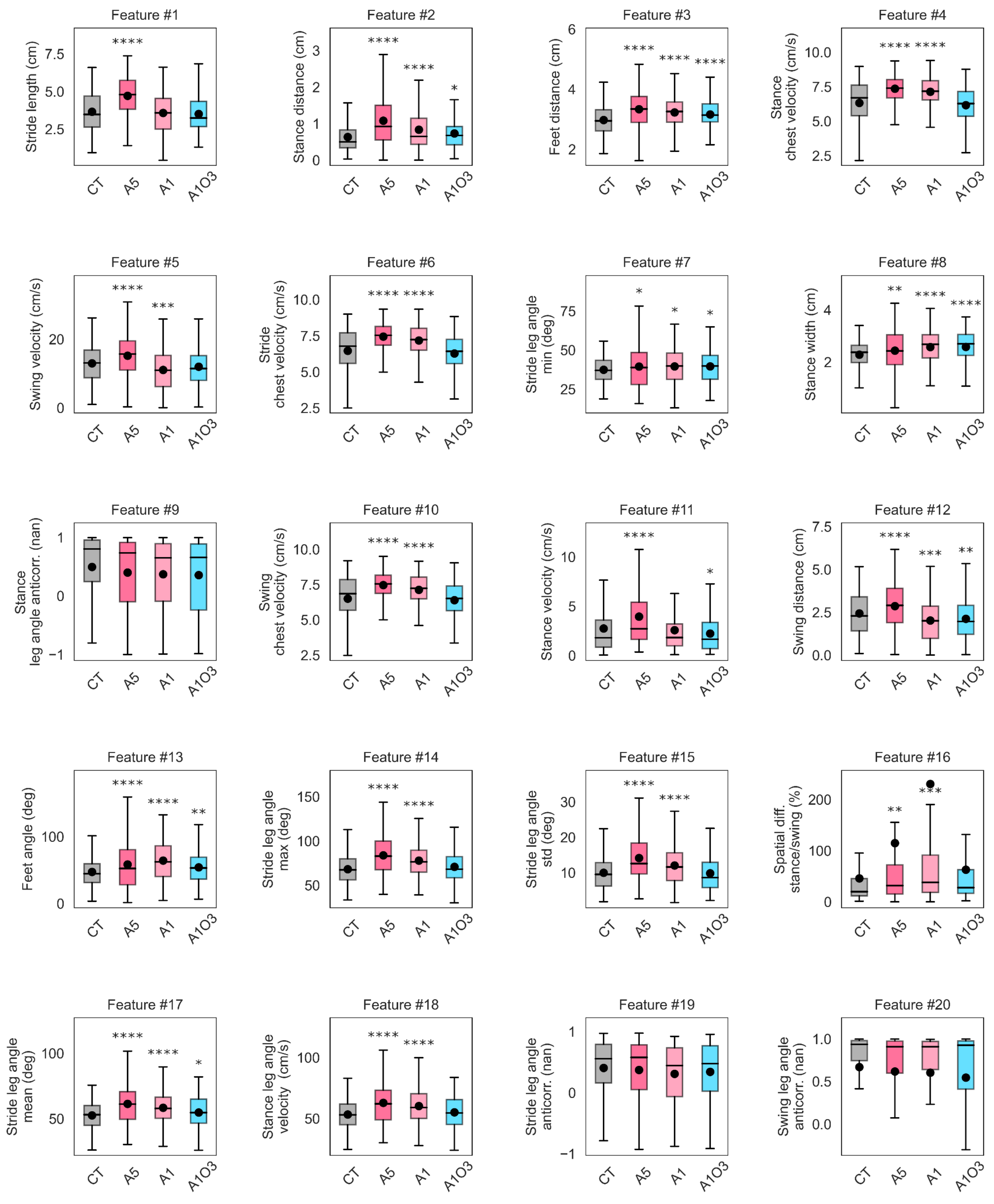

Supplementary Figure 44. Group comparison of the top 20 features (Gait model) in bar plots at 10 wk.

Box plots show the median (black line), mean (black circle), and interquartile range (box). Whiskers extend to the most extreme data points within 1.5 × the interquartile range from the lower and upper quartiles (Tukey method). Individual outliers beyond this range are omitted from the plot for clarity but are retained in the dataset and included in the calculation of the mean and median. Statistical analysis was performed by one-way ANOVA followed by Holm–Sidak-corrected Welch's t-tests comparing each group to CT. Significant differences are indicated by asterisks. Exact p-values and sample sizes are provided in Supplementary Table 22. Source data are provided in the Source Data file.

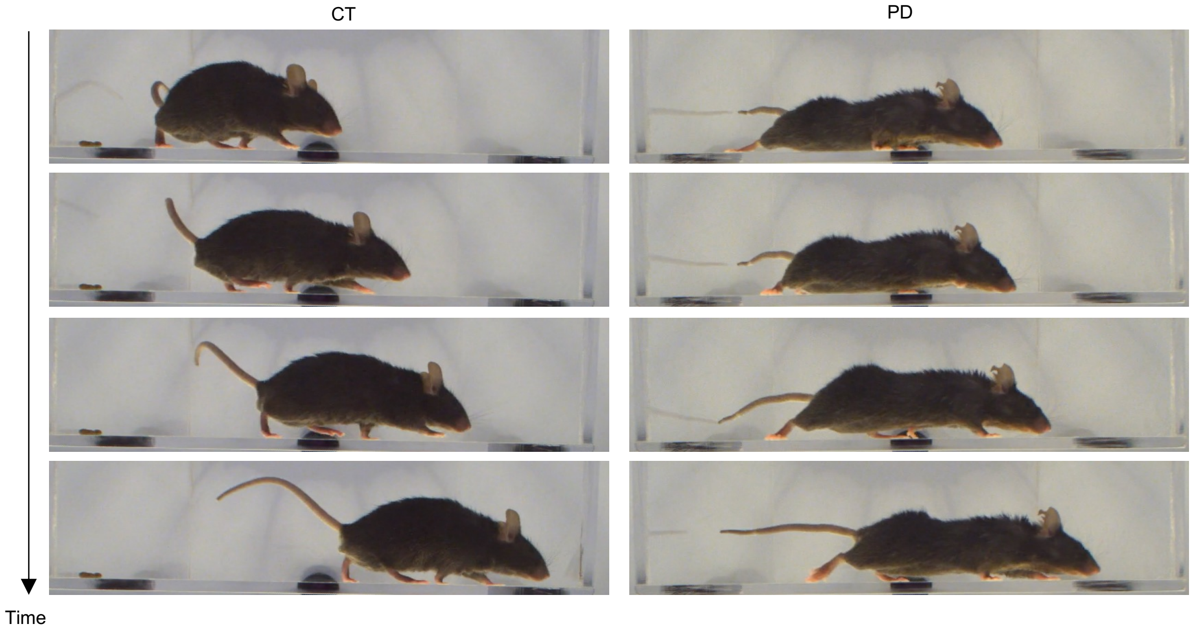

Supplementary Figure 45. Snapshot video images of CT and PD mice in walking.

Images were taken from OFT recordings at a manually decided cut-off to highlight differences in hindlimb positions between CT and PD mice during walking. For PD mice, images were captured during extreme hindlimb lagging/trailing (freezing gait). See Supplementary Movie 3.

Supplementary Tables

List of Supplementary Tables:

1. Summary of details for the key experimental groups.
2. Classifier training parameter by PyCaret default setting.
3. Statistical summary of the KL divergences for the top 20 features identified by the XGB model comparing CT and PD (A5) groups.
4. Statistical summary of the feature values for the top 20 features identified by the XGB model, comparing the CT and PD (A5) groups.
5. Statistical summary of the KL divergences for the top 20 features identified by the XGB model comparing NALS and ALS groups.
6. Statistical summary of the feature values for the top 20 features identified by the XGB model, comparing the NALS and ALS groups.
7. Statistical comparisons of the top 20 features identified by the XGB model comparing PD (A5) and ALS groups.
8. Statistical summary of the APS (XGB model-predicted PD scores) at 10 wk across groups.
9. Statistical summary of the KL divergences for the top 20 features identified by the XGB model.
10. Statistical summary of group comparisons (A1O3) for the top 20 features (identified by the XGB model) at 10 wk.
11. Statistical summary of group comparisons (A1O2) for the top 20 features (identified by the XGB model) at 10 wk.
12. Statistical summary of group comparisons (A1L1) for the top 20 features (identified by the XGB model) at 10 wk.
13. Statistical summary of group comparisons (A1L1) for the top 20 features (identified by the XGB model) at 10 wk.
14. Statistical summary of group comparisons (A1BT) for the top 20 features (identified by the XGB model) at 6 wk.
15. Statistical summary of treatment group comparisons for the top 20 features (identified by the XGB model) at endpoints.
16. Statistical summary of the KL divergences for the top 20 features identified by the TSFEL model comparing CT and PD (A5) groups.
17. Statistical summary of the feature values for the top 20 features identified by the TSFEL model, comparing the CT and PD (A5) groups.
18. Statistical summary of group comparisons (A1O3) for the top 20 features (TSFEL model) at 10 wk.
19. Statistical summary of group comparisons (A1O3) for the turning features at 10 wk.
20. Statistical summary of group comparisons (A1O3) for the rearing features at 10 wk.
21. Definitions of the Gait model top 20 features.
22. Statistical summary of group comparisons (A1O3) for the top 20 features (Gait model) at 10 wk.

Supplementary Table 1. Summary of details for the key experimental groups. Experimental groups are detailed by subgroup classification, description of experimental interventions, genetic backgrounds, gender, genotypes, experimental timelines, viral constructs (AAV-DJ/8-hSyn1; titres indicated), and the numbers of mice utilised for the total cohort, machine-learning (ML) dataset, unseen dataset for AI-predicted PD score (APS) analysis, and independent experimental replications. The total mouse n indicated in the table represents all mice included in this study; however, subsets of mice were variably involved across individual analyses, with specific numbers (n) described in the relevant text. Any groups not listed in this table or with amended details are described in the relevant text. DAT-CRE transgenic mice and SOD1-G93A transgenic mice were obtained from The Jackson Laboratory (strain #006302 and #002726, respectively).

Group	#	Sub-group	Description	Genetic background	Gender	Genotype	Experimental timeline	AAV-DJ/8-hSyn1 vrius construct (titre, gp/mL)	Total mouse n	ML mouse n	Unseen mouse n	Experiment n
CT	0	NI	No virus injection	C57BL/6J	Male	WT	20-30 wk old (10 wk)	-	12	4	8	3
	1	EV	Empty vector injection	C57BL/6J	Male	WT		DIO-RFP (1E+11)	10	0	10	2
	2	R1	low dose RFP expression	C57BL/6J	Male	WT		RFP (1E+12)	15	0	10	1
				B6SJL × C57BL/6J hybrid	Male	DAT-CRE		DIO-RFP (1E+12)		4	1	2
	3	R5	high dose RFP expression	C57BL/6J	Male	WT		RFP (5E+12)	13	0	10	1
				B6SJL × C57BL/6J hybrid	Male	DAT-CRE		DIO-RFP (5E+12)		2	1	1
PD	4	A1	low dose hA53T expression	C57BL/6J	Male	WT		hA53T (1E+12) + RFP (1E+11)	15	0	5	1
				B6SJL × C57BL/6J hybrid	Male	DAT-CRE		hA53T (1E+12) + DIO-RFP (1E+11)		0	10	5
	5	A5	high dose hA53T expression	C57BL/6J	Male	WT		hA53T (5E+12) + RFP (1E+11)	22	0	8	2
				B6SJL × C57BL/6J hybrid	Male	DAT-CRE		hA53T (5E+12) + DIO-RFP (1E+11)		11	3	5
MND	6	NALS	ALS control	B6SJL × C57BL/6J hybrid	Female	WT	10-20 wk old (10 wk)	-	2		2	1
	7	ALS	ALS model	B6SJL × C57BL/6J hybrid	Female	SOD1-G93A		-	2		2	1
optoRET	8	A1OD	A1 + optoRET + dark	B6SJL × C57BL/6J hybrid	Male	DAT-CRE	20-30 wk old (10 wk)	hA53T (1E+12) + DIO-optoRET (1E+11)	10		10	3
	9	A1O1	A1 + optoRET + light (daily schedule)	B6SJL × C57BL/6J hybrid	Male	DAT-CRE		hA53T (1E+12) + DIO-optoRET (1E+11)	7		7	3
	10	A1O2	A1 + optoRET + light (biweekly schedule)	B6SJL × C57BL/6J hybrid	Male	DAT-CRE		hA53T (1E+12) + DIO-optoRET (1E+11)	12		12	3
	11	A1O3	A1 + optoRET + light (alternate days schedule)	B6SJL × C57BL/6J hybrid	Male	DAT-CRE		hA53T (1E+12) + DIO-optoRET (1E+11)	8		8	2
	12	A5OD	A1 + optoRET + dark	B6SJL × C57BL/6J hybrid	Male	DAT-CRE		hA53T (5E+12) + DIO-optoRET (1E+11)	7		7	2
	13	A5O1	A1 + optoRET + light (everyday schedule)	B6SJL × C57BL/6J hybrid	Male	DAT-CRE		hA53T (5E+12) + DIO-optoRET (1E+11)	7		7	2
	14	A5O2	A1 + optoRET + light (biweekly schedule)	B6SJL × C57BL/6J hybrid	Male	DAT-CRE		hA53T (5E+12) + DIO-optoRET (1E+11)	6		6	2
L-DOPA	15	A1L1	A1 + L-DOPA (daily schedule)	C57BL/6J	Male	WT		hA53T (1E+12) + RFP (1E+11)	10		10	1
	16	A1L3	A1 + L-DOPA (alternate days schedule)	C57BL/6J	Male	WT		hA53T (1E+12) + RFP (1E+11)	10		10	2
RET agonist	17	A1BT	A1 + BT44 (direct brain infusion at a constant flow rate)	C57BL/6J	Male	WT	20-26 wk old (6 wk)	hA53T (1E+12) + RFP (1E+11)	6		6	1

Supplementary Table 2. Classifier training parameter by PyCaret default setting. These defaults are derived from the underlying libraries (e.g. scikit-learn, XGBoost, LightGBM) as used by PyCaret. In this study, these default parameter settings are applied during model training using PyCaret. Values reflect the defaults used in PyCaret version 2.3.10. The default parameters have changed in more recent PyCaret versions. For the latest details or changes, please refer to the official documentation for PyCaret (https://pycaret.org/), Scikit-learn (https://scikit-learn.org/stable/modules/classes.html), XGB (https://xgboost.readthedocs.io/) and LightGBM (https://lightgbm.readthedocs.io/).

Classifiers	Default parameters (PyCaret version 2.3.10)
Extreme Gradient Boosting (XGB)	'objective': 'binary:logistic', 'use_label_encoder': None, 'base_score': 0.5, 'booster': 'gbtree', 'callbacks': None, 'colsample_bylevel': 1, 'colsample_bynode': 1, 'colsample_bytree': 1, 'early_stopping_rounds': None, 'enable_categorical': False, 'eval_metric': None, 'feature_types': None, 'gamma': 0, 'gpu_id': -1, 'grow_policy': 'depthwise', 'importance_type': None, 'interaction_constraints': '', 'learning_rate': 0.300000012, 'max_bin': 256, 'max_cat_threshold': 64, 'max_cat_to_onehot': 4, 'max_delta_step': 0, 'max_depth': 6, 'max_leaves': 0, 'min_child_weight': 1, 'missing': nan, 'monotone_constraints': '()', 'n_estimators': 100, 'n_jobs': -1, 'num_parallel_tree': 1, 'predictor': 'auto', 'random_state': 101, 'reg_alpha': 0, 'reg_lambda': 1, 'sampling_method': 'uniform', 'scale_pos_weight': 1, 'subsample': 1, 'tree_method': 'auto', 'validate_parameters': 1, 'verbosity': 0
Extra Trees Classifier	'bootstrap': False, 'ccp_alpha': 0.0, 'class_weight': None, 'criterion': 'gini', 'max_depth': None, 'max_features': 'auto', 'max_leaf_nodes': None, 'max_samples': None, 'min_impurity_decrease': 0.0, 'min_impurity_split': None, 'min_samples_leaf': 1, 'min_samples_split': 2, 'min_weight_fraction_leaf': 0.0, 'n_estimators': 100, 'n_jobs': -1, 'oob_score': False, 'random_state': 101, 'verbose': 0, 'warm_start': False
Random Forest Classifier	'bootstrap': True, 'ccp_alpha': 0.0, 'class_weight': None, 'criterion': 'gini', 'max_depth': None, 'max_features': 'auto', 'max_leaf_nodes': None, 'max_samples': None, 'min_impurity_decrease': 0.0, 'min_impurity_split': None, 'min_samples_leaf': 1, 'min_samples_split': 2, 'min_weight_fraction_leaf': 0.0, 'n_estimators': 100, 'n_jobs': -1, 'oob_score': False, 'random_state': 101, 'verbose': 0, 'warm_start': False
LightGBM	'boosting_type': 'gbdt', 'class_weight': None, 'colsample_bytree': 1.0, 'importance_type': 'split', 'learning_rate': 0.1, 'max_depth': -1, 'min_child_samples': 20, 'min_child_weight': 0.001, 'min_split_gain': 0.0, 'n_estimators': 100, 'n_jobs': -1, 'num_leaves': 31, 'objective': None, 'random_state': 101, 'reg_alpha': 0.0, 'reg_lambda': 0.0, 'silent': 'warn', 'subsample': 1.0, 'subsample_for_bin': 200000, 'subsample_freq': 0
Gradient Boosting Classifier	ccp_alpha': 0.0, 'criterion': 'friedman_mse', 'init': None, 'learning_rate': 0.1, 'loss': 'deviance', 'max_depth': 3, 'max_features': None, 'max_leaf_nodes': None, 'min_impurity_decrease': 0.0, 'min_impurity_split': None, 'min_samples_leaf': 1, 'min_samples_split': 2, 'min_weight_fraction_leaf': 0.0, 'n_estimators': 100, 'n_iter_no_change': None, 'presort': 'deprecated', 'random_state': 101, 'subsample': 1.0, 'tol': 0.0001, 'validation_fraction': 0.1, 'verbose': 0, 'warm_start': False
AdaBoost Classifier	'algorithm': 'SAMME.R', 'base_estimator': None, 'learning_rate': 1.0, 'n_estimators': 50, 'random_state': 101
Decision Tree Classifier	'ccp_alpha': 0.0, 'class_weight': None, 'criterion': 'gini', 'max_depth': None, 'max_features': None, 'max_leaf_nodes': None, 'min_impurity_decrease': 0.0, 'min_impurity_split': None, 'min_samples_leaf': 1, 'min_samples_split': 2, 'min_weight_fraction_leaf': 0.0, 'presort': 'deprecated', 'random_state': 101, 'splitter': 'best'
Logistic Regression	'C': 1.0, 'class_weight': None, 'dual': False, 'fit_intercept': True, 'intercept_scaling': 1, 'l1_ratio': None, 'max_iter': 1000, 'multi_class': 'auto', 'n_jobs': None, 'penalty': 'l2', 'random_state': 101, 'solver': 'lbfgs', 'tol': 0.0001, 'verbose': 0, 'warm_start': False
Quadratic Discriminant Analysis (QDA)	'priors': None, 'reg_param': 0.0, 'store_covariance': False, 'tol': 0.0001
Linear Discriminant Analysis (LDA)	'n_components': None, 'priors': None, 'shrinkage': None, 'solver': 'svd', 'store_covariance': False, 'tol': 0.0001
Ridge Classifier	'alpha': 1.0, 'class_weight': None, 'copy_X': True, 'fit_intercept': True, 'max_iter': None, 'normalize': False, 'random_state': 101, 'solver': 'auto', 'tol': 0.001
K-Neighbors Classifier (KNN)	'algorithm': 'auto', 'leaf_size': 30, 'metric': 'minkowski', 'metric_params': None, 'n_jobs': -1, 'n_neighbors': 5, 'p': 2, 'weights': 'uniform'
SVM – Linear Kernel	'alpha': 0.0001, 'average': False, 'class_weight': None, 'early_stopping': False, 'epsilon': 0.1, 'eta0': 0.001, 'fit_intercept': True, 'l1_ratio': 0.15, 'learning_rate': 'optimal', 'loss': 'hinge', 'max_iter': 1000, 'n_iter_no_change': 5, 'n_jobs': -1, 'penalty': 'l2', 'power_t': 0.5, 'random_state': 101, 'shuffle': True, 'tol': 0.001, 'validation_fraction': 0.1, 'verbose': 0, 'warm_start': False
Naive Bayes (GaussianNB)	'priors': None, 'var_smoothing': 1e-09
Dummy Classifier	'constant': None, 'random_state': 101, 'strategy': 'prior'

Supplementary Table 3. Statistical summary of the KL divergences for the top 20 features identified by the XGB model comparing CT and PD (A5) groups. For each group, the KL divergence of each feature relative to its baseline value (0 wk) was computed across the experimental duration (0–10 wk). Each group consisted of 6 sampling sets, each containing 150 randomly selected clips. A two-way ANOVA (group × week) was performed for each feature, followed by weekly t-tests between groups (CT vs. A5). The resulting p-values from the weekly t-tests were corrected for multiple comparisons using the Bonferroni method. Source data are provided in the Source Data file (See S_Figure 16).

Rank	Feature name	group	Week	n	Mean	SEM	raw_p	corrected_p	significant	ANOVA_p_Week	ANOVA_p_Group	ANOVA_p_Interaction
1	Hands 3D distance_mean	A5	0	6	0	0		1	ns	3.70E-06	1.47E-05	0.001887869
1	Hands 3D distance_mean	CT	0	6	0	0		1	ns	3.70E-06	1.47E-05	0.001887869
1	Hands 3D distance_mean	A5	2	6	0.06326374	0.01014952	0.34822232	1	ns	3.70E-06	1.47E-05	0.001887869
1	Hands 3D distance_mean	CT	2	6	0.0467916	0.01330755	0.34822232	1	ns	3.70E-06	1.47E-05	0.001887869
1	Hands 3D distance_mean	A5	4	6	0.0559817	0.0093035	0.89139966	1	ns	3.70E-06	1.47E-05	0.001887869
1	Hands 3D distance_mean	CT	4	6	0.05325403	0.01711037	0.89139966	1	ns	3.70E-06	1.47E-05	0.001887869
1	Hands 3D distance_mean	A5	6	6	0.10657	0.02795522	0.02396557	0.14379344	ns	3.70E-06	1.47E-05	0.001887869
1	Hands 3D distance_mean	CT	6	6	0.02967787	0.00742458	0.02396557	0.14379344	ns	3.70E-06	1.47E-05	0.001887869
1	Hands 3D distance_mean	A5	8	6	0.12496121	0.03171454	0.11088119	0.66528714	ns	3.70E-06	1.47E-05	0.001887869
1	Hands 3D distance_mean	CT	8	6	0.06820251	0.0068897	0.11088119	0.66528714	ns	3.70E-06	1.47E-05	0.001887869
1	Hands 3D distance_mean	A5	10	6	0.1631368	0.03157142	0.00250439	0.01502632	*	3.70E-06	1.47E-05	0.001887869
1	Hands 3D distance_mean	CT	10	6	0.03208499	0.00864933	0.00250439	0.01502632	*	3.70E-06	1.47E-05	0.001887869
2	Motion duration_episode	A5	0	6	0	0		1	ns	2.12E-11	0.004076711	0.011551432
2	Motion duration_episode	CT	0	6	0	0		1	ns	2.12E-11	0.004076711	0.011551432
2	Motion duration_episode	A5	2	6	0.07111439	0.01795587	0.58579887	1	ns	2.12E-11	0.004076711	0.011551432
2	Motion duration_episode	CT	2	6	0.05821458	0.01422924	0.58579887	1	ns	2.12E-11	0.004076711	0.011551432
2	Motion duration_episode	A5	4	6	0.12304998	0.01010413	0.42818587	1	ns	2.12E-11	0.004076711	0.011551432
2	Motion duration_episode	CT	4	6	0.17242783	0.05893497	0.42818587	1	ns	2.12E-11	0.004076711	0.011551432
2	Motion duration_episode	A5	6	6	0.20369362	0.03261895	0.02274363	0.1364618	ns	2.12E-11	0.004076711	0.011551432
2	Motion duration_episode	CT	6	6	0.08710899	0.02856457	0.02274363	0.1364618	ns	2.12E-11	0.004076711	0.011551432
2	Motion duration_episode	A5	8	6	0.1764069	0.01268369	0.00086766	0.00520593	**	2.12E-11	0.004076711	0.011551432
2	Motion duration_episode	CT	8	6	0.08971392	0.01350124	0.00086766	0.00520593	**	2.12E-11	0.004076711	0.011551432
2	Motion duration_episode	A5	10	6	0.29149972	0.02340172	0.03876793	0.23260761	ns	2.12E-11	0.004076711	0.011551432
2	Motion duration_episode	CT	10	6	0.18678527	0.03730938	0.03876793	0.23260761	ns	2.12E-11	0.004076711	0.011551432
3	Body 2D length_min	A5	0	6	0	0		1	ns	4.48E-14	6.64E-18	2.68E-12
3	Body 2D length_min	CT	0	6	0	0		1	ns	4.48E-14	6.64E-18	2.68E-12
3	Body 2D length_min	A5	2	6	0.06409272	0.01500922	0.03932401	0.23594406	ns	4.48E-14	6.64E-18	2.68E-12
3	Body 2D length_min	CT	2	6	0.02358804	0.00818433	0.03932401	0.23594406	ns	4.48E-14	6.64E-18	2.68E-12
3	Body 2D length_min	A5	4	6	0.14214055	0.01264605	6.05E-06	3.63E-05	****	4.48E-14	6.64E-18	2.68E-12
3	Body 2D length_min	CT	4	6	0.02429108	0.00516854	6.05E-06	3.63E-05	****	4.48E-14	6.64E-18	2.68E-12
3	Body 2D length_min	A5	6	6	0.62480182	0.04535404	2.21E-07	1.33E-06	****	4.48E-14	6.64E-18	2.68E-12
3	Body 2D length_min	CT	6	6	0.04946249	0.01046577	2.21E-07	1.33E-06	****	4.48E-14	6.64E-18	2.68E-12
3	Body 2D length_min	A5	8	6	0.59102622	0.11906223	0.00085374	0.00512247	**	4.48E-14	6.64E-18	2.68E-12
3	Body 2D length_min	CT	8	6	0.03124369	0.00818214	0.00085374	0.00512247	**	4.48E-14	6.64E-18	2.68E-12
3	Body 2D length_min	A5	10	6	0.41894261	0.04233956	5.03E-06	3.02E-05	****	4.48E-14	6.64E-18	2.68E-12
3	Body 2D length_min	CT	10	6	0.04253695	0.00583053	5.03E-06	3.02E-05	****	4.48E-14	6.64E-18	2.68E-12
4	Tail angle_mean	A5	0	6	0	0		1	ns	4.66E-19	1.35E-19	3.23E-15
4	Tail angle_mean	CT	0	6	0	0		1	ns	4.66E-19	1.35E-19	3.23E-15
4	Tail angle_mean	A5	2	6	0.04282326	0.00789761	0.09239186	0.55435114	ns	4.66E-19	1.35E-19	3.23E-15
4	Tail angle_mean	CT	2	6	0.02446527	0.00591249	0.09239186	0.55435114	ns	4.66E-19	1.35E-19	3.23E-15
4	Tail angle_mean	A5	4	6	0.0626721	0.01716293	0.1694277	1	ns	4.66E-19	1.35E-19	3.23E-15
4	Tail angle_mean	CT	4	6	0.03505663	0.0072906	0.1694277	1	ns	4.66E-19	1.35E-19	3.23E-15
4	Tail angle_mean	A5	6	6	0.15211919	0.01546048	1.15E-05	6.91E-05	****	4.66E-19	1.35E-19	3.23E-15
4	Tail angle_mean	CT	6	6	0.02763181	0.00138379	1.15E-05	6.91E-05	****	4.66E-19	1.35E-19	3.23E-15
4	Tail angle_mean	A5	8	6	0.26063073	0.0281452	3.90E-05	0.00023411	***	4.66E-19	1.35E-19	3.23E-15
4	Tail angle_mean	CT	8	6	0.04007553	0.01456618	3.90E-05	0.00023411	***	4.66E-19	1.35E-19	3.23E-15
4	Tail angle_mean	A5	10	6	0.28013069	0.02141627	1.28E-06	7.67E-06	****	4.66E-19	1.35E-19	3.23E-15
4	Tail angle_mean	CT	10	6	0.04024065	0.00949229	1.28E-06	7.67E-06	****	4.66E-19	1.35E-19	3.23E-15
5	Arm angle asym._std	A5	0	6	0	0		1	ns	6.54E-19	3.24E-22	8.43E-20
5	Arm angle asym._std	CT	0	6	0	0		1	ns	6.54E-19	3.24E-22	8.43E-20
5	Arm angle asym._std	A5	2	6	0.05208557	0.00708974	0.38325495	1	ns	6.54E-19	3.24E-22	8.43E-20
5	Arm angle asym._std	CT	2	6	0.07087614	0.01934677	0.38325495	1	ns	6.54E-19	3.24E-22	8.43E-20
5	Arm angle asym._std	A5	4	6	0.07478935	0.01584264	0.36289443	1	ns	6.54E-19	3.24E-22	8.43E-20
5	Arm angle asym._std	CT	4	6	0.05337683	0.01592131	0.36289443	1	ns	6.54E-19	3.24E-22	8.43E-20
5	Arm angle asym._std	A5	6	6	0.29072932	0.02294471	3.95E-07	2.37E-06	****	6.54E-19	3.24E-22	8.43E-20
5	Arm angle asym._std	CT	6	6	0.02104382	0.00347032	3.95E-07	2.37E-06	****	6.54E-19	3.24E-22	8.43E-20
5	Arm angle asym._std	A5	8	6	0.23458031	0.0127636	2.58E-06	1.55E-05	****	6.54E-19	3.24E-22	8.43E-20
5	Arm angle asym._std	CT	8	6	0.05331553	0.01422925	2.58E-06	1.55E-05	****	6.54E-19	3.24E-22	8.43E-20
5	Arm angle asym._std	A5	10	6	0.28006733	0.01811415	9.58E-08	5.75E-07	****	6.54E-19	3.24E-22	8.43E-20
5	Arm angle asym._std	CT	10	6	0.02166987	0.00618071	9.58E-08	5.75E-07	****	6.54E-19	3.24E-22	8.43E-20
6	Motion interval_episode	A5	0	6	0	0		1	ns	1.28E-05	0.163170352	0.004161448
6	Motion interval_episode	CT	0	6	0	0		1	ns	1.28E-05	0.163170352	0.004161448
6	Motion interval_episode	A5	2	6	0.08445343	0.02221478	0.81834867	1	ns	1.28E-05	0.163170352	0.004161448
6	Motion interval_episode	CT	2	6	0.0785088	0.01191991	0.81834867	1	ns	1.28E-05	0.163170352	0.004161448
6	Motion interval_episode	A5	4	6	0.18331525	0.04861667	0.10432924	0.62597546	ns	1.28E-05	0.163170352	0.004161448
6	Motion interval_episode	CT	4	6	0.08752678	0.02261625	0.10432924	0.62597546	ns	1.28E-05	0.163170352	0.004161448
6	Motion interval_episode	A5	6	6	0.08466906	0.01909086	0.00040122	0.00240734	**	1.28E-05	0.163170352	0.004161448
6	Motion interval_episode	CT	6	6	0.36514645	0.05044505	0.00040122	0.00240734	**	1.28E-05	0.163170352	0.004161448
6	Motion interval_episode	A5	8	6	0.08427983	0.02323727	0.23443366	1	ns	1.28E-05	0.163170352	0.004161448
6	Motion interval_episode	CT	8	6	0.13145645	0.02915619	0.23443366	1	ns	1.28E-05	0.163170352	0.004161448
6	Motion interval_episode	A5	10	6	0.23841472	0.13105982	0.9924736	1	ns	1.28E-05	0.163170352	0.004161448
6	Motion interval_episode	CT	10	6	0.23713502	0.01819803	0.9924736	1	ns	1.28E-05	0.163170352	0.004161448
7	Body angle_min	A5	0	6	0	0		1	ns	0.000190308	3.80E-07	0.010806696
7	Body angle_min	CT	0	6	0	0		1	ns	0.000190308	3.80E-07	0.010806696
7	Body angle_min	A5	2	6	0.06000111	0.02059593	0.2758596	1	ns	0.000190308	3.80E-07	0.010806696
7	Body angle_min	CT	2	6	0.03426522	0.00862098	0.2758596	1	ns	0.000190308	3.80E-07	0.010806696
7	Body angle_min	A5	4	6	0.05134669	0.01501236	0.0506779	0.3040674	ns	0.000190308	3.80E-07	0.010806696
7	Body angle_min	CT	4	6	0.01739716	0.00290707	0.0506779	0.3040674	ns	0.000190308	3.80E-07	0.010806696
7	Body angle_min	A5	6	6	0.13442126	0.02768538	0.00277436	0.01664614	*	0.000190308	3.80E-07	0.010806696
7	Body angle_min	CT	6	6	0.02308279	0.00565822	0.00277436	0.01664614	*	0.000190308	3.80E-07	0.010806696
7	Body angle_min	A5	8	6	0.13380843	0.04071754	0.01891012	0.11346075	ns	0.000190308	3.80E-07	0.010806696
7	Body angle_min	CT	8	6	0.01962039	0.00307588	0.01891012	0.11346075	ns	0.000190308	3.80E-07	0.010806696
7	Body angle_min	A5	10	6	0.13454111	0.03367772	0.02218709	0.13312252	ns	0.000190308	3.80E-07	0.010806696
7	Body angle_min	CT	10	6	0.03655812	0.01339983	0.02218709	0.13312252	ns	0.000190308	3.80E-07	0.010806696
8	Feet 2D distance_max	A5	0	6	0	0		1	ns	1.48E-10	8.96E-16	1.72E-06
8	Feet 2D distance_max	CT	0	6	0	0		1	ns	1.48E-10	8.96E-16	1.72E-06
8	Feet 2D distance_max	A5	2	6	0.2644145	0.04157157	0.00067538	0.00405227	**	1.48E-10	8.96E-16	1.72E-06
8	Feet 2D distance_max	CT	2	6	0.05402271	0.01251777	0.00067538	0.00405227	**	1.48E-10	8.96E-16	1.72E-06
8	Feet 2D distance_max	A5	4	6	0.16415819	0.00923678	0.00132575	0.00795447	**	1.48E-10	8.96E-16	1.72E-06
8	Feet 2D distance_max	CT	4	6	0.0902789	0.01400128	0.00132575	0.00795447	**	1.48E-10	8.96E-16	1.72E-06
8	Feet 2D distance_max	A5	6	6	0.25957569	0.03346563	0.00012179	0.00073074	***	1.48E-10	8.96E-16	1.72E-06
8	Feet 2D distance_max	CT	6	6	0.04521612	0.01143702	0.00012179	0.00073074	***	1.48E-10	8.96E-16	1.72E-06
8	Feet 2D distance_max	A5	8	6	0.17904429	0.02008271	0.01966929	0.11801574	ns	1.48E-10	8.96E-16	1.72E-06
8	Feet 2D distance_max	CT	8	6	0.09045323	0.02483918	0.01966929	0.11801574	ns	1.48E-10	8.96E-16	1.72E-06
8	Feet 2D distance_max	A5	10	6	0.19775405	0.01728937	4.13E-06	2.48E-05	****	1.48E-10	8.96E-16	1.72E-06
8	Feet 2D distance_max	CT	10	6	0.03676453	0.00456398	4.13E-06	2.48E-05	****	1.48E-10	8.96E-16	1.72E-06
9	Neck 3D velocity_std	A5	0	6	0	0		1	ns	5.95E-14	3.17E-14	1.58E-08
9	Neck 3D velocity_std	CT	0	6	0	0		1	ns	5.95E-14	3.17E-14	1.58E-08
9	Neck 3D velocity_std	A5	2	6	0.06875228	0.01015042	0.01601466	0.09608796	ns	5.95E-14	3.17E-14	1.58E-08
9	Neck 3D velocity_std	CT	2	6	0.0341245	0.00634052	0.01601466	0.09608796	ns	5.95E-14	3.17E-14	1.58E-08
9	Neck 3D velocity_std	A5	4	6	0.04656228	0.010095	0.20657647	1	ns	5.95E-14	3.17E-14	1.58E-08
9	Neck 3D velocity_std	CT	4	6	0.03024082	0.00664203	0.20657647	1	ns	5.95E-14	3.17E-14	1.58E-08
9	Neck 3D velocity_std	A5	6	6	0.16645244	0.01251136	1.16E-05	6.98E-05	****	5.95E-14	3.17E-14	1.58E-08
9	Neck 3D velocity_std	CT	6	6	0.04077018	0.00946668	1.16E-05	6.98E-05	****	5.95E-14	3.17E-14	1.58E-08
9	Neck 3D velocity_std	A5	8	6	0.11927038	0.01380192	4.31E-05	0.00025877	***	5.95E-14	3.17E-14	1.58E-08
9	Neck 3D velocity_std	CT	8	6	0.02264524	0.00262936	4.31E-05	0.00025877	***	5.95E-14	3.17E-14	1.58E-08
9	Neck 3D velocity_std	A5	10	6	0.08531186	0.01671612	0.01846582	0.11079492	ns	5.95E-14	3.17E-14	1.58E-08
9	Neck 3D velocity_std	CT	10	6	0.0325462	0.00855018	0.01846582	0.11079492	ns	5.95E-14	3.17E-14	1.58E-08
10	Feet 2D distance_std	A5	0	6	0	0		1	ns	6.59E-12	1.10E-17	6.88E-13
10	Feet 2D distance_std	CT	0	6	0	0		1	ns	6.59E-12	1.10E-17	6.88E-13
10	Feet 2D distance_std	A5	2	6	0.0827779	0.02263045	0.82861064	1	ns	6.59E-12	1.10E-17	6.88E-13
10	Feet 2D distance_std	CT	2	6	0.07531385	0.02481912	0.82861064	1	ns	6.59E-12	1.10E-17	6.88E-13
10	Feet 2D distance_std	A5	4	6	0.11034303	0.01777957	0.08564207	0.51385244	ns	6.59E-12	1.10E-17	6.88E-13
10	Feet 2D distance_std	CT	4	6	0.05549901	0.02260691	0.08564207	0.51385244	ns	6.59E-12	1.10E-17	6.88E-13
10	Feet 2D distance_std	A5	6	6	0.34307724	0.03775398	7.62E-06	4.57E-05	****	6.59E-12	1.10E-17	6.88E-13
10	Feet 2D distance_std	CT	6	6	0.02368241	0.00431611	7.62E-06	4.57E-05	****	6.59E-12	1.10E-17	6.88E-13
10	Feet 2D distance_std	A5	8	6	0.29224545	0.04431305	0.00013264	0.00079587	***	6.59E-12	1.10E-17	6.88E-13
10	Feet 2D distance_std	CT	8	6	0.02603619	0.00262397	0.00013264	0.00079587	***	6.59E-12	1.10E-17	6.88E-13
10	Feet 2D distance_std	A5	10	6	0.37314355	0.03787602	3.55E-06	2.13E-05	****	6.59E-12	1.10E-17	6.88E-13
10	Feet 2D distance_std	CT	10	6	0.02511744	0.00325392	3.55E-06	2.13E-05	****	6.59E-12	1.10E-17	6.88E-13
11	Chest 3D velocity_mean	A5	0	6	0	0		1	ns	6.58E-12	5.17E-18	1.06E-08
11	Chest 3D velocity_mean	CT	0	6	0	0		1	ns	6.58E-12	5.17E-18	1.06E-08
11	Chest 3D velocity_mean	A5	2	6	0.07533779	0.01507015	0.01594906	0.09569436	ns	6.58E-12	5.17E-18	1.06E-08
11	Chest 3D velocity_mean	CT	2	6	0.02896287	0.00541882	0.01594906	0.09569436	ns	6.58E-12	5.17E-18	1.06E-08
11	Chest 3D velocity_mean	A5	4	6	0.10392427	0.01597309	0.00074429	0.00446577	**	6.58E-12	5.17E-18	1.06E-08
11	Chest 3D velocity_mean	CT	4	6	0.02360413	0.00520217	0.00074429	0.00446577	**	6.58E-12	5.17E-18	1.06E-08
11	Chest 3D velocity_mean	A5	6	6	0.09390074	0.0149346	0.00041498	0.00248986	**	6.58E-12	5.17E-18	1.06E-08
11	Chest 3D velocity_mean	CT	6	6	0.01377535	0.00406404	0.00041498	0.00248986	**	6.58E-12	5.17E-18	1.06E-08
11	Chest 3D velocity_mean	A5	8	6	0.15001469	0.01582964	4.55E-05	0.00027324	***	6.58E-12	5.17E-18	1.06E-08
11	Chest 3D velocity_mean	CT	8	6	0.02898825	0.00794776	4.55E-05	0.00027324	***	6.58E-12	5.17E-18	1.06E-08
11	Chest 3D velocity_mean	A5	10	6	0.20136695	0.02414726	2.81E-05	0.00016876	***	6.58E-12	5.17E-18	1.06E-08
11	Chest 3D velocity_mean	CT	10	6	0.02304223	0.00496618	2.81E-05	0.00016876	***	6.58E-12	5.17E-18	1.06E-08
12	U-body 3D length_min	A5	0	6	0	0		1	ns	1.09E-11	1.00E-10	3.07E-08
12	U-body 3D length_min	CT	0	6	0	0		1	ns	1.09E-11	1.00E-10	3.07E-08
12	U-body 3D length_min	A5	2	6	0.03328983	0.01025936	0.82321345	1	ns	1.09E-11	1.00E-10	3.07E-08
12	U-body 3D length_min	CT	2	6	0.03644877	0.00918908	0.82321345	1	ns	1.09E-11	1.00E-10	3.07E-08
12	U-body 3D length_min	A5	4	6	0.05008596	0.00738428	0.04874433	0.29246596	ns	1.09E-11	1.00E-10	3.07E-08
12	U-body 3D length_min	CT	4	6	0.03067043	0.00451562	0.04874433	0.29246596	ns	1.09E-11	1.00E-10	3.07E-08
12	U-body 3D length_min	A5	6	6	0.29517801	0.03083891	1.68E-05	0.0001007	***	1.09E-11	1.00E-10	3.07E-08
12	U-body 3D length_min	CT	6	6	0.04707094	0.00960665	1.68E-05	0.0001007	***	1.09E-11	1.00E-10	3.07E-08
12	U-body 3D length_min	A5	8	6	0.17123544	0.03852456	0.00422542	0.02535254	*	1.09E-11	1.00E-10	3.07E-08
12	U-body 3D length_min	CT	8	6	0.02830379	0.0046862	0.00422542	0.02535254	*	1.09E-11	1.00E-10	3.07E-08
12	U-body 3D length_min	A5	10	6	0.22214934	0.04594141	0.00849319	0.05095913	ns	1.09E-11	1.00E-10	3.07E-08
12	U-body 3D length_min	CT	10	6	0.05787398	0.02049267	0.00849319	0.05095913	ns	1.09E-11	1.00E-10	3.07E-08
13	Right limb angle asym._min	A5	0	6	0	0		1	ns	8.24E-11	2.72E-12	1.73E-09
13	Right limb angle asym._min	CT	0	6	0	0		1	ns	8.24E-11	2.72E-12	1.73E-09
13	Right limb angle asym._min	A5	2	6	0.05127703	0.01470565	0.71429505	1	ns	8.24E-11	2.72E-12	1.73E-09
13	Right limb angle asym._min	CT	2	6	0.0627374	0.02663646	0.71429505	1	ns	8.24E-11	2.72E-12	1.73E-09
13	Right limb angle asym._min	A5	4	6	0.06450111	0.01148595	0.04586696	0.27520178	ns	8.24E-11	2.72E-12	1.73E-09
13	Right limb angle asym._min	CT	4	6	0.03246968	0.00810089	0.04586696	0.27520178	ns	8.24E-11	2.72E-12	1.73E-09
13	Right limb angle asym._min	A5	6	6	0.21055329	0.03104624	0.00026615	0.00159691	**	8.24E-11	2.72E-12	1.73E-09
13	Right limb angle asym._min	CT	6	6	0.03301829	0.00908556	0.00026615	0.00159691	**	8.24E-11	2.72E-12	1.73E-09
13	Right limb angle asym._min	A5	8	6	0.16977854	0.0301356	0.00445325	0.02671951	*	8.24E-11	2.72E-12	1.73E-09
13	Right limb angle asym._min	CT	8	6	0.05286347	0.01082474	0.00445325	0.02671951	*	8.24E-11	2.72E-12	1.73E-09
13	Right limb angle asym._min	A5	10	6	0.24157123	0.02126199	1.75E-06	1.05E-05	****	8.24E-11	2.72E-12	1.73E-09
13	Right limb angle asym._min	CT	10	6	0.02779852	0.0037893	1.75E-06	1.05E-05	****	8.24E-11	2.72E-12	1.73E-09
14	Hands 3D distance_max	A5	0	6	0	0		1	ns	3.43E-11	3.74E-11	1.07E-10
14	Hands 3D distance_max	CT	0	6	0	0		1	ns	3.43E-11	3.74E-11	1.07E-10
14	Hands 3D distance_max	A5	2	6	0.04182894	0.00636427	0.0801269	0.48076139	ns	3.43E-11	3.74E-11	1.07E-10
14	Hands 3D distance_max	CT	2	6	0.07217149	0.01422425	0.0801269	0.48076139	ns	3.43E-11	3.74E-11	1.07E-10
14	Hands 3D distance_max	A5	4	6	0.07195629	0.01003378	0.4422395	1	ns	3.43E-11	3.74E-11	1.07E-10
14	Hands 3D distance_max	CT	4	6	0.05895477	0.01278182	0.4422395	1	ns	3.43E-11	3.74E-11	1.07E-10
14	Hands 3D distance_max	A5	6	6	0.19562954	0.03237317	0.00049446	0.00296678	**	3.43E-11	3.74E-11	1.07E-10
14	Hands 3D distance_max	CT	6	6	0.02931727	0.00580981	0.00049446	0.00296678	**	3.43E-11	3.74E-11	1.07E-10
14	Hands 3D distance_max	A5	8	6	0.17968456	0.02903418	0.00425971	0.02555824	*	3.43E-11	3.74E-11	1.07E-10
14	Hands 3D distance_max	CT	8	6	0.06117207	0.01397195	0.00425971	0.02555824	*	3.43E-11	3.74E-11	1.07E-10
14	Hands 3D distance_max	A5	10	6	0.28237674	0.03700409	6.76E-05	0.00040571	***	3.43E-11	3.74E-11	1.07E-10
14	Hands 3D distance_max	CT	10	6	0.03579513	0.00793728	6.76E-05	0.00040571	***	3.43E-11	3.74E-11	1.07E-10
15	Left limb angle asym._min	A5	0	6	0	0		1	ns	2.10E-16	2.63E-18	4.69E-14
15	Left limb angle asym._min	CT	0	6	0	0		1	ns	2.10E-16	2.63E-18	4.69E-14
15	Left limb angle asym._min	A5	2	6	0.05611124	0.00880741	0.54556278	1	ns	2.10E-16	2.63E-18	4.69E-14
15	Left limb angle asym._min	CT	2	6	0.07426418	0.02764587	0.54556278	1	ns	2.10E-16	2.63E-18	4.69E-14
15	Left limb angle asym._min	A5	4	6	0.11122671	0.00824466	0.0065996	0.03959762	*	2.10E-16	2.63E-18	4.69E-14
15	Left limb angle asym._min	CT	4	6	0.0502991	0.01582031	0.0065996	0.03959762	*	2.10E-16	2.63E-18	4.69E-14
15	Left limb angle asym._min	A5	6	6	0.27698244	0.02435668	7.70E-06	4.62E-05	****	2.10E-16	2.63E-18	4.69E-14
15	Left limb angle asym._min	CT	6	6	0.04962139	0.01183968	7.70E-06	4.62E-05	****	2.10E-16	2.63E-18	4.69E-14
15	Left limb angle asym._min	A5	8	6	0.25317066	0.02311507	1.23E-05	7.36E-05	****	2.10E-16	2.63E-18	4.69E-14
15	Left limb angle asym._min	CT	8	6	0.05165149	0.01030345	1.23E-05	7.36E-05	****	2.10E-16	2.63E-18	4.69E-14
15	Left limb angle asym._min	A5	10	6	0.32885166	0.02961174	7.22E-06	4.33E-05	****	2.10E-16	2.63E-18	4.69E-14
15	Left limb angle asym._min	CT	10	6	0.05017235	0.01446786	7.22E-06	4.33E-05	****	2.10E-16	2.63E-18	4.69E-14
16	Chest 1D velocity_max	A5	0	6	0	0		1	ns	1.59E-15	9.37E-20	5.08E-14
16	Chest 1D velocity_max	CT	0	6	0	0		1	ns	1.59E-15	9.37E-20	5.08E-14
16	Chest 1D velocity_max	A5	2	6	0.04357876	0.01194792	0.31837385	1	ns	1.59E-15	9.37E-20	5.08E-14
16	Chest 1D velocity_max	CT	2	6	0.0274224	0.0096927	0.31837385	1	ns	1.59E-15	9.37E-20	5.08E-14
16	Chest 1D velocity_max	A5	4	6	0.14555879	0.02351458	0.000312	0.00187198	**	1.59E-15	9.37E-20	5.08E-14
16	Chest 1D velocity_max	CT	4	6	0.01656027	0.00478585	0.000312	0.00187198	**	1.59E-15	9.37E-20	5.08E-14
16	Chest 1D velocity_max	A5	6	6	0.61160776	0.08085198	3.58E-05	0.00021478	***	1.59E-15	9.37E-20	5.08E-14
16	Chest 1D velocity_max	CT	6	6	0.04171792	0.00572391	3.58E-05	0.00021478	***	1.59E-15	9.37E-20	5.08E-14
16	Chest 1D velocity_max	A5	8	6	0.63740754	0.093519	9.18E-05	0.00055094	***	1.59E-15	9.37E-20	5.08E-14
16	Chest 1D velocity_max	CT	8	6	0.04747825	0.00938226	9.18E-05	0.00055094	***	1.59E-15	9.37E-20	5.08E-14
16	Chest 1D velocity_max	A5	10	6	0.59952935	0.0581671	1.74E-06	1.05E-05	****	1.59E-15	9.37E-20	5.08E-14
16	Chest 1D velocity_max	CT	10	6	0.02127263	0.00530976	1.74E-06	1.05E-05	****	1.59E-15	9.37E-20	5.08E-14
17	Leg angle asym._std	A5	0	6	0	0		1	ns	1.92E-19	3.75E-26	6.64E-20
17	Leg angle asym._std	CT	0	6	0	0		1	ns	1.92E-19	3.75E-26	6.64E-20
17	Leg angle asym._std	A5	2	6	0.06981462	0.00701969	0.96997417	1	ns	1.92E-19	3.75E-26	6.64E-20
17	Leg angle asym._std	CT	2	6	0.07053767	0.01737033	0.96997417	1	ns	1.92E-19	3.75E-26	6.64E-20
17	Leg angle asym._std	A5	4	6	0.14491879	0.02154037	0.00381564	0.02289385	*	1.92E-19	3.75E-26	6.64E-20
17	Leg angle asym._std	CT	4	6	0.05561112	0.01023372	0.00381564	0.02289385	*	1.92E-19	3.75E-26	6.64E-20
17	Leg angle asym._std	A5	6	6	0.37176468	0.01460637	6.39E-10	3.84E-09	****	1.92E-19	3.75E-26	6.64E-20
17	Leg angle asym._std	CT	6	6	0.02147196	0.005123	6.39E-10	3.84E-09	****	1.92E-19	3.75E-26	6.64E-20
17	Leg angle asym._std	A5	8	6	0.3791829	0.03375254	1.80E-06	1.08E-05	****	1.92E-19	3.75E-26	6.64E-20
17	Leg angle asym._std	CT	8	6	0.03900716	0.00706413	1.80E-06	1.08E-05	****	1.92E-19	3.75E-26	6.64E-20
17	Leg angle asym._std	A5	10	6	0.31591565	0.03292106	5.10E-06	3.06E-05	****	1.92E-19	3.75E-26	6.64E-20
17	Leg angle asym._std	CT	10	6	0.02510402	0.00315167	5.10E-06	3.06E-05	****	1.92E-19	3.75E-26	6.64E-20
18	Feet 2D distance_mean	A5	0	6	0	0		1	ns	3.24E-11	0.612695322	5.89E-05
18	Feet 2D distance_mean	CT	0	6	0	0		1	ns	3.24E-11	0.612695322	5.89E-05
18	Feet 2D distance_mean	A5	2	6	0.25673444	0.03126128	0.00526495	0.03158967	*	3.24E-11	0.612695322	5.89E-05
18	Feet 2D distance_mean	CT	2	6	0.11825592	0.0233246	0.00526495	0.03158967	*	3.24E-11	0.612695322	5.89E-05
18	Feet 2D distance_mean	A5	4	6	0.12998836	0.03567013	0.02945785	0.17674709	ns	3.24E-11	0.612695322	5.89E-05
18	Feet 2D distance_mean	CT	4	6	0.26262821	0.03819228	0.02945785	0.17674709	ns	3.24E-11	0.612695322	5.89E-05
18	Feet 2D distance_mean	A5	6	6	0.09771067	0.01497626	0.85969289	1	ns	3.24E-11	0.612695322	5.89E-05
18	Feet 2D distance_mean	CT	6	6	0.10450972	0.03436341	0.85969289	1	ns	3.24E-11	0.612695322	5.89E-05
18	Feet 2D distance_mean	A5	8	6	0.08121341	0.01440475	0.32696402	1	ns	3.24E-11	0.612695322	5.89E-05
18	Feet 2D distance_mean	CT	8	6	0.11055718	0.02455655	0.32696402	1	ns	3.24E-11	0.612695322	5.89E-05
18	Feet 2D distance_mean	A5	10	6	0.07340963	0.01249338	0.54623559	1	ns	3.24E-11	0.612695322	5.89E-05
18	Feet 2D distance_mean	CT	10	6	0.08513202	0.0140063	0.54623559	1	ns	3.24E-11	0.612695322	5.89E-05
19	Anus 1D velocity_std	A5	0	6	0	0		1	ns	5.25E-06	0.000451879	0.103016963
19	Anus 1D velocity_std	CT	0	6	0	0		1	ns	5.25E-06	0.000451879	0.103016963
19	Anus 1D velocity_std	A5	2	6	0.02764453	0.00816703	0.29112947	1	ns	5.25E-06	0.000451879	0.103016963
19	Anus 1D velocity_std	CT	2	6	0.01830622	0.00187212	0.29112947	1	ns	5.25E-06	0.000451879	0.103016963
19	Anus 1D velocity_std	A5	4	6	0.03463997	0.00511071	0.64767038	1	ns	5.25E-06	0.000451879	0.103016963
19	Anus 1D velocity_std	CT	4	6	0.03098544	0.00583573	0.64767038	1	ns	5.25E-06	0.000451879	0.103016963
19	Anus 1D velocity_std	A5	6	6	0.04860527	0.01449294	0.04203963	0.2522378	ns	5.25E-06	0.000451879	0.103016963
19	Anus 1D velocity_std	CT	6	6	0.01404688	0.00314849	0.04203963	0.2522378	ns	5.25E-06	0.000451879	0.103016963
19	Anus 1D velocity_std	A5	8	6	0.0398745	0.00823775	0.0459311	0.27558658	ns	5.25E-06	0.000451879	0.103016963
19	Anus 1D velocity_std	CT	8	6	0.02036767	0.00233625	0.0459311	0.27558658	ns	5.25E-06	0.000451879	0.103016963
19	Anus 1D velocity_std	A5	10	6	0.04124408	0.00275109	0.08927034	0.53562205	ns	5.25E-06	0.000451879	0.103016963
19	Anus 1D velocity_std	CT	10	6	0.02664164	0.00725613	0.08927034	0.53562205	ns	5.25E-06	0.000451879	0.103016963
20	Arm angle asym._mean	A5	0	6	0	0		1	ns	1.48E-16	2.14E-21	1.07E-12
20	Arm angle asym._mean	CT	0	6	0	0		1	ns	1.48E-16	2.14E-21	1.07E-12
20	Arm angle asym._mean	A5	2	6	0.06448882	0.00768231	0.00261162	0.01566973	*	1.48E-16	2.14E-21	1.07E-12
20	Arm angle asym._mean	CT	2	6	0.02907693	0.00449974	0.00261162	0.01566973	*	1.48E-16	2.14E-21	1.07E-12
20	Arm angle asym._mean	A5	4	6	0.06963367	0.00955676	0.00219802	0.0131881	*	1.48E-16	2.14E-21	1.07E-12
20	Arm angle asym._mean	CT	4	6	0.02532902	0.00513019	0.00219802	0.0131881	*	1.48E-16	2.14E-21	1.07E-12
20	Arm angle asym._mean	A5	6	6	0.19392683	0.02371782	8.21E-05	0.00049263	***	1.48E-16	2.14E-21	1.07E-12
20	Arm angle asym._mean	CT	6	6	0.02939541	0.01029664	8.21E-05	0.00049263	***	1.48E-16	2.14E-21	1.07E-12
20	Arm angle asym._mean	A5	8	6	0.14757785	0.01312113	1.04E-05	6.22E-05	****	1.48E-16	2.14E-21	1.07E-12
20	Arm angle asym._mean	CT	8	6	0.03463421	0.00463095	1.04E-05	6.22E-05	****	1.48E-16	2.14E-21	1.07E-12
20	Arm angle asym._mean	A5	10	6	0.17663468	0.01233792	3.22E-07	1.93E-06	****	1.48E-16	2.14E-21	1.07E-12
20	Arm angle asym._mean	CT	10	6	0.02284132	0.00392646	3.22E-07	1.93E-06	****	1.48E-16	2.14E-21	1.07E-12

Supplementary Table 4. Statistical summary of the feature values for the top 20 features identified by the XGB model, comparing the CT and PD (A5) groups. For each feature, the values at each week were divided by the group's corresponding baseline (0 wk) mean value, and the normalised data were analysed over the experimental duration (0–10 wk). Each group consisted of 6 sampling sets, each containing 150 randomly selected clips. Two-way ANOVA (group × week) was performed for each feature on the normalised data, followed by weekly t-tests comparing the CT and A5 groups. P-values from weekly t-tests were corrected for multiple comparisons using the Bonferroni method. Source data are provided in the Source Data file (See S_Figure 17).

Rank	Feature name	Week	group	n	Mean	SEM	raw_p	corrected_p	significant	ANOVA_p_Week	ANOVA_p_Group	ANOVA_p_Interaction
1	Hands 3D distance_mean	0	CT	1766	1	0.00624222	1	1	ns	1.00E+00	1.00E+00	0.715666923
1	Hands 3D distance_mean	2	CT	1485	0.9597192	0.00627342	0.01207393	0.07244356	ns	1.00E+00	1.00E+00	0.715666923
1	Hands 3D distance_mean	4	CT	1173	0.96435594	0.00710173	0.33098172	1	ns	1.00E+00	1.00E+00	0.715666923
1	Hands 3D distance_mean	6	CT	1069	0.97191978	0.00778373	3.5459E-18	2.1275E-17	****	1.00E+00	1.00E+00	0.715666923
1	Hands 3D distance_mean	8	CT	1056	0.91376941	0.0076422	5.3365E-22	3.2019E-21	****	1.00E+00	1.00E+00	0.715666923
1	Hands 3D distance_mean	10	CT	1003	0.99652569	0.00823853	3.2893E-15	1.9736E-14	****	1.00E+00	1.00E+00	0.715666923
1	Hands 3D distance_mean	0	A5	2197	1	0.00769333	1	1	ns	1.00E+00	1.00E+00	0.715666923
1	Hands 3D distance_mean	2	A5	2203	0.98595563	0.0074628	0.01207393	0.07244356	ns	1.00E+00	1.00E+00	0.715666923
1	Hands 3D distance_mean	4	A5	1784	0.97718664	0.00962751	0.33098172	1	ns	1.00E+00	1.00E+00	0.715666923
1	Hands 3D distance_mean	6	A5	2207	1.12362483	0.01147099	3.5459E-18	2.1275E-17	****	1.00E+00	1.00E+00	0.715666923
1	Hands 3D distance_mean	8	A5	2822	1.08641357	0.01050433	5.3365E-22	3.2019E-21	****	1.00E+00	1.00E+00	0.715666923
1	Hands 3D distance_mean	10	A5	2756	1.15921203	0.01203477	3.2893E-15	1.9736E-14	****	1.00E+00	1.00E+00	0.715666923
2	Motion duration_episode	0	CT	1740	1	0.01440406	1	1	ns	1.00E+00	1	0.001190587
2	Motion duration_episode	2	CT	1469	1.01858632	0.01877639	0.00361896	0.02171373	*	1.00E+00	1	0.001190587
2	Motion duration_episode	4	CT	1178	0.86228792	0.01499087	1.5629E-36	9.3772E-36	****	1.00E+00	1	0.001190587
2	Motion duration_episode	6	CT	1084	0.86079671	0.0154548	4.348E-23	2.6088E-22	****	1.00E+00	1	0.001190587
2	Motion duration_episode	8	CT	1072	0.98943932	0.02322775	1.0772E-31	6.463E-31	****	1.00E+00	1	0.001190587
2	Motion duration_episode	10	CT	1028	0.79694783	0.01604723	7.1383E-34	4.283E-33	****	1.00E+00	1	0.001190587
2	Motion duration_episode	0	A5	2179	1	0.01731228	1	1	ns	1.00E+00	1	0.001190587
2	Motion duration_episode	2	A5	2195	1.10730042	0.02152758	0.00361896	0.02171373	*	1.00E+00	1	0.001190587
2	Motion duration_episode	4	A5	1795	1.33359301	0.02816807	1.5629E-36	9.3772E-36	****	1.00E+00	1	0.001190587
2	Motion duration_episode	6	A5	2250	1.34042835	0.03255006	4.348E-23	2.6088E-22	****	1.00E+00	1	0.001190587
2	Motion duration_episode	8	A5	2778	1.52159763	0.02649018	1.0772E-31	6.463E-31	****	1.00E+00	1	0.001190587
2	Motion duration_episode	10	A5	2826	1.58415246	0.03831381	7.1383E-34	4.283E-33	****	1.00E+00	1	0.001190587
3	Body 2D length_min	0	CT	1756	1	0.00692289	1	1	ns	1.00E+00	1.00E+00	4.39E-01
3	Body 2D length_min	2	CT	1480	1.00048019	0.00714572	4.8926E-32	2.9355E-31	****	1.00E+00	1.00E+00	4.39E-01
3	Body 2D length_min	4	CT	1160	1.01918047	0.00815912	1.7392E-44	1.0435E-43	****	1.00E+00	1.00E+00	4.39E-01
3	Body 2D length_min	6	CT	1076	1.06941962	0.00827668	3.318E-117	1.991E-116	****	1.00E+00	1.00E+00	4.39E-01
3	Body 2D length_min	8	CT	1067	1.02047984	0.00873362	2.30E-170	1.38E-169	****	1.00E+00	1.00E+00	4.39E-01
3	Body 2D length_min	10	CT	1013	1.06986767	0.0086857	2.35E-96	1.41E-95	****	1.00E+00	1.00E+00	4.39E-01
3	Body 2D length_min	0	A5	2131	1	0.0067836	1.00E+00	1.00E+00	ns	1.00E+00	1.00E+00	4.39E-01
3	Body 2D length_min	2	A5	2165	1.12039897	0.00675403	4.89E-32	2.94E-31	****	1.00E+00	1.00E+00	4.39E-01
3	Body 2D length_min	4	A5	1792	1.17337681	0.00693719	1.7392E-44	1.0435E-43	****	1.00E+00	1.00E+00	4.39E-01
3	Body 2D length_min	6	A5	2222	1.30199949	0.00543285	3.318E-117	1.991E-116	****	1.00E+00	1.00E+00	4.39E-01
3	Body 2D length_min	8	A5	2862	1.28854701	0.00455023	2.30E-170	1.38E-169	****	1.00E+00	1.00E+00	4.39E-01
3	Body 2D length_min	10	A5	2797	1.2770145	0.00489067	2.35E-96	1.41E-95	****	1.00E+00	1.00E+00	4.39E-01
4	Tail angle_mean	0	CT	1772	1	0.00295247	1	1	ns	1.00E+00	1.00E+00	1.83E-01
4	Tail angle_mean	2	CT	1479	0.98882041	0.00303378	4.4383E-29	2.663E-28	****	1.00E+00	1.00E+00	1.83E-01
4	Tail angle_mean	4	CT	1162	0.98377041	0.0035668	1.6603E-28	9.9619E-28	****	1.00E+00	1.00E+00	1.83E-01
4	Tail angle_mean	6	CT	1072	0.9860915	0.00389636	8.4212E-32	5.0527E-31	****	1.00E+00	1.00E+00	1.83E-01
4	Tail angle_mean	8	CT	1057	0.98007574	0.00350151	1.251E-113	7.503E-113	****	1.00E+00	1.00E+00	1.83E-01
4	Tail angle_mean	10	CT	1010	0.97906589	0.00385674	1.134E-102	6.805E-102	****	1.00E+00	1.00E+00	1.83E-01
4	Tail angle_mean	0	A5	2081	1	0.0025597	1.00E+00	1.00E+00	ns	1.00E+00	1.00E+00	1.83E-01
4	Tail angle_mean	2	A5	2146	1.03217487	0.00240624	4.44E-29	2.66E-28	****	1.00E+00	1.00E+00	1.83E-01
4	Tail angle_mean	4	A5	1793	1.03029653	0.0024201	1.66E-28	9.9619E-28	****	1.00E+00	1.00E+00	1.83E-01
4	Tail angle_mean	6	A5	2243	1.03143814	0.00187641	8.42E-32	5.0527E-31	****	1.00E+00	1.00E+00	1.83E-01
4	Tail angle_mean	8	A5	2853	1.06272858	0.00171213	1.25E-113	7.50E-113	****	1.00E+00	1.00E+00	1.83E-01
4	Tail angle_mean	10	A5	2784	1.05851444	0.00164117	1.13E-102	6.81E-102	****	1.00E+00	1.00E+00	1.83E-01
5	Arm angle asym._std	0	CT	1768	1	0.00981411	1	1	ns	1.00E+00	1.00E+00	8.03E-01
5	Arm angle asym._std	2	CT	1480	0.95869992	0.00941665	2.7947E-15	1.6768E-14	****	1.00E+00	1.00E+00	8.03E-01
5	Arm angle asym._std	4	CT	1168	0.96406887	0.01067851	7.2531E-26	4.3518E-25	****	1.00E+00	1.00E+00	8.03E-01
5	Arm angle asym._std	6	CT	1068	0.98690127	0.01213016	6.9989E-74	4.1994E-73	****	1.00E+00	1.00E+00	8.03E-01
5	Arm angle asym._std	8	CT	1064	0.96807004	0.01130133	3.78E-70	2.268E-69	****	1.00E+00	1.00E+00	8.03E-01
5	Arm angle asym._std	10	CT	1004	1.01770638	0.01291671	9.4284E-60	5.657E-59	****	1.00E+00	1.00E+00	8.03E-01
5	Arm angle asym._std	0	A5	2061	1	0.01053231	1.00E+00	1.00E+00	ns	1.00E+00	1.00E+00	8.03E-01
5	Arm angle asym._std	2	A5	2204	1.08014601	0.01083295	2.79E-15	1.68E-14	****	1.00E+00	1.00E+00	8.03E-01
5	Arm angle asym._std	4	A5	1794	1.16858984	0.01390237	7.25E-26	4.35E-25	****	1.00E+00	1.00E+00	8.03E-01
5	Arm angle asym._std	6	A5	2223	1.42655697	0.01526312	7.00E-74	4.20E-73	****	1.00E+00	1.00E+00	8.03E-01
5	Arm angle asym._std	8	A5	2847	1.36498588	0.01274748	3.78E-70	2.27E-69	****	1.00E+00	1.00E+00	8.03E-01
5	Arm angle asym._std	10	A5	2771	1.42209511	0.01390026	9.43E-60	5.66E-59	****	1.00E+00	1.00E+00	8.03E-01
6	Motion interval_episode	0	CT	1735	1	0.03547019	1	1	ns	1.00E+00	1	0.980372693
6	Motion interval_episode	2	CT	1435	1.26091159	0.04861972	5.2843E-13	3.1706E-12	****	1.00E+00	1	0.980372693
6	Motion interval_episode	4	CT	1141	1.28844409	0.05844416	1.1752E-20	7.0511E-20	****	1.00E+00	1	0.980372693
6	Motion interval_episode	6	CT	1007	2.30032307	0.08927596	1.5985E-67	9.5909E-67	****	1.00E+00	1	0.980372693
6	Motion interval_episode	8	CT	1019	1.61307697	0.07242427	1.3722E-45	8.2335E-45	****	1.00E+00	1	0.980372693
6	Motion interval_episode	10	CT	951	2.04801625	0.07752967	1.373E-124	8.239E-124	****	1.00E+00	1	0.980372693
6	Motion interval_episode	0	A5	2088	1	0.03121839	1	1	ns	1.00E+00	1	0.980372693
6	Motion interval_episode	2	A5	2099	0.88477145	0.02716546	5.2843E-13	3.1706E-12	****	1.00E+00	1	0.980372693
6	Motion interval_episode	4	A5	1730	0.72671304	0.02956831	1.1752E-20	7.0511E-20	****	1.00E+00	1	0.980372693
6	Motion interval_episode	6	A5	2169	0.93033157	0.03221267	1.5985E-67	9.5909E-67	****	1.00E+00	1	0.980372693
6	Motion interval_episode	8	A5	2752	0.7507241	0.02481409	1.3722E-45	8.2335E-45	****	1.00E+00	1	0.980372693
6	Motion interval_episode	10	A5	2790	0.65921153	0.01955832	1.373E-124	8.239E-124	****	1.00E+00	1	0.980372693
7	Body angle_min	0	CT	1771	1	0.0159485	1	1	ns	0.999999999	1.00E+00	0.552339291
7	Body angle_min	2	CT	1467	1.02748744	0.01739344	6.0527E-05	0.00036316	***	0.999999999	1.00E+00	0.552339291
7	Body angle_min	4	CT	1165	1.09639538	0.02027833	0.64362031	1	ns	0.999999999	1.00E+00	0.552339291
7	Body angle_min	6	CT	1078	1.15093813	0.0208656	1.072E-09	6.4322E-09	****	0.999999999	1.00E+00	0.552339291
7	Body angle_min	8	CT	1064	1.12687849	0.02172	1.2471E-09	7.4828E-09	****	0.999999999	1.00E+00	0.552339291
7	Body angle_min	10	CT	1007	1.18660792	0.0228174	0.00098049	0.00588294	**	0.999999999	1.00E+00	0.552339291
7	Body angle_min	0	A5	2088	1	0.01480109	1	1	ns	0.999999999	1.00E+00	0.552339291
7	Body angle_min	2	A5	2178	1.11532739	0.01360406	6.0527E-05	0.00036316	***	0.999999999	1.00E+00	0.552339291
7	Body angle_min	4	A5	1768	1.10801423	0.01539397	0.64362031	1	ns	0.999999999	1.00E+00	0.552339291
7	Body angle_min	6	A5	2229	1.30197654	0.01389698	1.072E-09	6.4322E-09	****	0.999999999	1.00E+00	0.552339291
7	Body angle_min	8	A5	2841	1.27322637	0.01225533	1.2471E-09	7.4828E-09	****	0.999999999	1.00E+00	0.552339291
7	Body angle_min	10	A5	2796	1.267765	0.01226713	0.00098049	0.00588294	**	0.999999999	1.00E+00	0.552339291
8	Feet 2D distance_max	0	CT	1776	1	0.00300005	1	1	ns	1.00E+00	1.00E+00	8.22E-02
8	Feet 2D distance_max	2	CT	1482	0.97220516	0.00294622	3.494E-137	2.096E-136	****	1.00E+00	1.00E+00	8.22E-02
8	Feet 2D distance_max	4	CT	1173	0.95684935	0.00332436	2.109E-106	1.266E-105	****	1.00E+00	1.00E+00	8.22E-02
8	Feet 2D distance_max	6	CT	1069	0.98434513	0.00353373	1.7398E-82	1.0439E-81	****	1.00E+00	1.00E+00	8.22E-02
8	Feet 2D distance_max	8	CT	1043	0.9567551	0.00387451	6.697E-100	4.018E-99	****	1.00E+00	1.00E+00	8.22E-02
8	Feet 2D distance_max	10	CT	1009	1.00303109	0.00374583	3.2811E-46	1.9687E-45	****	1.00E+00	1.00E+00	8.22E-02
8	Feet 2D distance_max	0	A5	2135	1	0.00344696	1	1	ns	1.00E+00	1.00E+00	8.22E-02
8	Feet 2D distance_max	2	A5	2210	1.10112522	0.00354377	3.494E-137	2.096E-136	****	1.00E+00	1.00E+00	8.22E-02
8	Feet 2D distance_max	4	A5	1799	1.07949877	0.00375815	2.109E-106	1.266E-105	****	1.00E+00	1.00E+00	8.22E-02
8	Feet 2D distance_max	6	A5	2206	1.11057863	0.00409477	1.7398E-82	1.0439E-81	****	1.00E+00	1.00E+00	8.22E-02
8	Feet 2D distance_max	8	A5	2833	1.08754929	0.00334017	6.70E-100	4.02E-99	****	1.00E+00	1.00E+00	8.22E-02
8	Feet 2D distance_max	10	A5	2786	1.09306807	0.00349103	3.28E-46	1.97E-45	****	1.00E+00	1.00E+00	8.22E-02
9	Neck 3D velocity_std	0	CT	1774	1	0.00553029	1	1	ns	1.00E+00	1.00E+00	9.06E-01
9	Neck 3D velocity_std	2	CT	1473	1.03567681	0.00615863	1.6742E-05	0.00010045	***	1.00E+00	1.00E+00	9.06E-01
9	Neck 3D velocity_std	4	CT	1174	1.00319024	0.0068272	2.258E-06	1.3548E-05	****	1.00E+00	1.00E+00	9.06E-01
9	Neck 3D velocity_std	6	CT	1071	0.96808526	0.00694727	7.2649E-29	4.359E-28	****	1.00E+00	1.00E+00	9.06E-01
9	Neck 3D velocity_std	8	CT	1055	0.98359819	0.00731176	2.4899E-20	1.494E-19	****	1.00E+00	1.00E+00	9.06E-01
9	Neck 3D velocity_std	10	CT	1005	0.9920473	0.00745398	4.7096E-15	2.8257E-14	****	1.00E+00	1.00E+00	9.06E-01
9	Neck 3D velocity_std	0	A5	2133	1	0.00526206	1.00E+00	1.00E+00	ns	1.00E+00	1.00E+00	9.06E-01
9	Neck 3D velocity_std	2	A5	2172	0.99655732	0.00619829	1.67E-05	1.00E-04	***	1.00E+00	1.00E+00	9.06E-01
9	Neck 3D velocity_std	4	A5	1792	0.95815297	0.00625972	2.26E-06	1.3548E-05	****	1.00E+00	1.00E+00	9.06E-01
9	Neck 3D velocity_std	6	A5	2218	0.86175001	0.00564273	7.26E-29	4.359E-28	****	1.00E+00	1.00E+00	9.06E-01
9	Neck 3D velocity_std	8	A5	2806	0.90026014	0.00476374	2.4899E-20	1.494E-19	****	1.00E+00	1.00E+00	9.06E-01
9	Neck 3D velocity_std	10	A5	2779	0.92348689	0.00449456	4.7096E-15	2.8257E-14	****	1.00E+00	1.00E+00	9.06E-01
10	Feet 2D distance_std	0	CT	1780	1	0.01171072	1	1	ns	1.00E+00	1.00E+00	8.34E-01
10	Feet 2D distance_std	2	CT	1480	0.93755416	0.01274896	2.8469E-29	1.7081E-28	****	1.00E+00	1.00E+00	8.34E-01
10	Feet 2D distance_std	4	CT	1166	1.00968799	0.01431052	6.955E-19	4.173E-18	****	1.00E+00	1.00E+00	8.34E-01
10	Feet 2D distance_std	6	CT	1072	0.95136255	0.01533082	3.964E-101	2.379E-100	****	1.00E+00	1.00E+00	8.34E-01
10	Feet 2D distance_std	8	CT	1051	1.02095268	0.01627548	2.9108E-73	1.7465E-72	****	1.00E+00	1.00E+00	8.34E-01
10	Feet 2D distance_std	10	CT	1003	1.01482686	0.01623753	5.8438E-73	3.5063E-72	****	1.00E+00	1.00E+00	8.34E-01
10	Feet 2D distance_std	0	A5	2144	1	0.01167641	1.00E+00	1.00E+00	ns	1.00E+00	1.00E+00	8.34E-01
10	Feet 2D distance_std	2	A5	2196	1.14214503	0.01208058	2.85E-29	1.71E-28	****	1.00E+00	1.00E+00	8.34E-01
10	Feet 2D distance_std	4	A5	1788	1.20471555	0.01495089	6.955E-19	4.173E-18	****	1.00E+00	1.00E+00	8.34E-01
10	Feet 2D distance_std	6	A5	2215	1.46126515	0.01422182	3.964E-101	2.379E-100	****	1.00E+00	1.00E+00	8.34E-01
10	Feet 2D distance_std	8	A5	2842	1.45096258	0.01279393	2.91E-73	1.75E-72	****	1.00E+00	1.00E+00	8.34E-01
10	Feet 2D distance_std	10	A5	2784	1.45575897	0.01308489	5.84E-73	3.51E-72	****	1.00E+00	1.00E+00	8.34E-01
11	Chest 3D velocity_mean	0	CT	1771	1	0.00815669	1	1	ns	1.00E+00	1.00E+00	7.05E-01
11	Chest 3D velocity_mean	2	CT	1468	1.01362003	0.008637	2.3975E-13	1.4385E-12	****	1.00E+00	1.00E+00	7.05E-01
11	Chest 3D velocity_mean	4	CT	1170	0.99508811	0.00994973	2.6263E-19	1.5758E-18	****	1.00E+00	1.00E+00	7.05E-01
11	Chest 3D velocity_mean	6	CT	1060	0.99032271	0.010161	1.9524E-15	1.1714E-14	****	1.00E+00	1.00E+00	7.05E-01
11	Chest 3D velocity_mean	8	CT	1066	0.9922996	0.01039418	4.673E-40	2.8038E-39	****	1.00E+00	1.00E+00	7.05E-01
11	Chest 3D velocity_mean	10	CT	1017	1.02453013	0.01099013	6.4997E-27	3.8998E-26	****	1.00E+00	1.00E+00	7.05E-01
11	Chest 3D velocity_mean	0	A5	2130	1	0.00759552	1	1	ns	1.00E+00	1.00E+00	7.05E-01
11	Chest 3D velocity_mean	2	A5	2189	1.10910899	0.00892063	2.3975E-13	1.4385E-12	****	1.00E+00	1.00E+00	7.05E-01
11	Chest 3D velocity_mean	4	A5	1752	1.12958932	0.01017169	2.63E-19	1.5758E-18	****	1.00E+00	1.00E+00	7.05E-01
11	Chest 3D velocity_mean	6	A5	2202	1.10513481	0.00869643	1.95E-15	1.1714E-14	****	1.00E+00	1.00E+00	7.05E-01
11	Chest 3D velocity_mean	8	A5	2820	1.17815184	0.00756858	4.67E-40	2.8038E-39	****	1.00E+00	1.00E+00	7.05E-01
11	Chest 3D velocity_mean	10	A5	2807	1.18191098	0.00779369	6.50E-27	3.8998E-26	****	1.00E+00	1.00E+00	7.05E-01
12	U-body 3D length_min	0	CT	1788	1	0.00393496	1	1	ns	1.00E+00	1.00E+00	6.50E-01
12	U-body 3D length_min	2	CT	1459	1.00026364	0.00446982	0.01376045	0.08256269	ns	1.00E+00	1.00E+00	6.50E-01
12	U-body 3D length_min	4	CT	1164	0.99205008	0.00463483	0.00151058	0.00906347	**	1.00E+00	1.00E+00	6.50E-01
12	U-body 3D length_min	6	CT	1075	1.04022876	0.00526272	6.6282E-05	0.00039769	***	1.00E+00	1.00E+00	6.50E-01
12	U-body 3D length_min	8	CT	1056	1.01681389	0.00527785	0.00052487	0.0031492	**	1.00E+00	1.00E+00	6.50E-01
12	U-body 3D length_min	10	CT	1010	1.02614662	0.00524594	3.8107E-12	2.2864E-11	****	1.00E+00	1.00E+00	6.50E-01
12	U-body 3D length_min	0	A5	2153	1	0.00376613	1.00E+00	1	ns	1.00E+00	1.00E+00	6.50E-01
12	U-body 3D length_min	2	A5	2147	0.98590253	0.00372097	1.38E-02	0.08256269	ns	1.00E+00	1.00E+00	6.50E-01
12	U-body 3D length_min	4	A5	1773	1.01092559	0.00373302	0.00151058	0.00906347	**	1.00E+00	1.00E+00	6.50E-01
12	U-body 3D length_min	6	A5	2249	1.06204212	0.0028157	6.6282E-05	0.00039769	***	1.00E+00	1.00E+00	6.50E-01
12	U-body 3D length_min	8	A5	2861	1.03476372	0.00246552	0.00052487	0.0031492	**	1.00E+00	1.00E+00	6.50E-01
12	U-body 3D length_min	10	A5	2786	1.06339181	0.00259722	3.8107E-12	2.2864E-11	****	1.00E+00	1.00E+00	6.50E-01
13	Right limb angle asym._min	0	CT	1764	1	0.00884524	1	1	ns	1.00E+00	1.00E+00	6.12E-01
13	Right limb angle asym._min	2	CT	1469	1.00648601	0.00922726	8.614E-06	5.1684E-05	****	1.00E+00	1.00E+00	6.12E-01
13	Right limb angle asym._min	4	CT	1167	1.01890137	0.01066097	0.00155494	0.00932963	**	1.00E+00	1.00E+00	6.12E-01
13	Right limb angle asym._min	6	CT	1077	1.01870168	0.01152953	2.6333E-22	1.58E-21	****	1.00E+00	1.00E+00	6.12E-01
13	Right limb angle asym._min	8	CT	1060	1.07109727	0.01167798	2.0637E-08	1.2382E-07	****	1.00E+00	1.00E+00	6.12E-01
13	Right limb angle asym._min	10	CT	1015	1.0433047	0.01163656	2.3659E-16	1.4195E-15	****	1.00E+00	1.00E+00	6.12E-01
13	Right limb angle asym._min	0	A5	2124	1	0.00978322	1	1	ns	1.00E+00	1.00E+00	6.12E-01
13	Right limb angle asym._min	2	A5	2203	1.07374352	0.01068062	8.614E-06	5.1684E-05	****	1.00E+00	1.00E+00	6.12E-01
13	Right limb angle asym._min	4	A5	1786	1.07955903	0.013825	0.00155494	0.00932963	**	1.00E+00	1.00E+00	6.12E-01
13	Right limb angle asym._min	6	A5	2195	1.26308244	0.01655332	2.6333E-22	1.58E-21	****	1.00E+00	1.00E+00	6.12E-01
13	Right limb angle asym._min	8	A5	2844	1.19873139	0.01316787	2.06E-08	1.24E-07	****	1.00E+00	1.00E+00	6.12E-01
13	Right limb angle asym._min	10	A5	2748	1.25278473	0.01484099	2.37E-16	1.42E-15	****	1.00E+00	1.00E+00	6.12E-01
14	Hands 3D distance_max	0	CT	1762	1	0.00565675	1	1	ns	1.00E+00	1.00E+00	5.99E-01
14	Hands 3D distance_max	2	CT	1488	0.97309214	0.00579577	4.2981E-08	2.5788E-07	****	1.00E+00	1.00E+00	5.99E-01
14	Hands 3D distance_max	4	CT	1175	0.96746171	0.00678629	1.3575E-14	8.1449E-14	****	1.00E+00	1.00E+00	5.99E-01
14	Hands 3D distance_max	6	CT	1062	0.98168417	0.0074669	2.629E-49	1.5774E-48	****	1.00E+00	1.00E+00	5.99E-01
14	Hands 3D distance_max	8	CT	1061	0.95453808	0.00757269	8.6345E-51	5.1807E-50	****	1.00E+00	1.00E+00	5.99E-01
14	Hands 3D distance_max	10	CT	1004	1.01493284	0.00791276	5.0629E-44	3.0377E-43	****	1.00E+00	1.00E+00	5.99E-01
14	Hands 3D distance_max	0	A5	2169	1	0.00669862	1	1	ns	1.00E+00	1.00E+00	5.99E-01
14	Hands 3D distance_max	2	A5	2203	1.02706347	0.00706815	4.2981E-08	2.5788E-07	****	1.00E+00	1.00E+00	5.99E-01
14	Hands 3D distance_max	4	A5	1781	1.07259569	0.01008358	1.3575E-14	8.1449E-14	****	1.00E+00	1.00E+00	5.99E-01
14	Hands 3D distance_max	6	A5	2217	1.23918795	0.01132086	2.629E-49	1.5774E-48	****	1.00E+00	1.00E+00	5.99E-01
14	Hands 3D distance_max	8	A5	2833	1.21670579	0.01015988	8.63E-51	5.1807E-50	****	1.00E+00	1.00E+00	5.99E-01
14	Hands 3D distance_max	10	A5	2766	1.27850028	0.01089068	5.06E-44	3.0377E-43	****	1.00E+00	1.00E+00	5.99E-01
15	Left limb angle asym._min	0	CT	1759	1	0.00922314	1	1	ns	1.00E+00	1.00E+00	9.50E-02
15	Left limb angle asym._min	2	CT	1484	0.97761304	0.00850426	3.2633E-13	1.958E-12	****	1.00E+00	1.00E+00	9.50E-02
15	Left limb angle asym._min	4	CT	1172	0.97520194	0.0099066	6.5084E-09	3.905E-08	****	1.00E+00	1.00E+00	9.50E-02
15	Left limb angle asym._min	6	CT	1067	1.02019899	0.01165576	1.8627E-36	1.1176E-35	****	1.00E+00	1.00E+00	9.50E-02
15	Left limb angle asym._min	8	CT	1057	1.01722967	0.01072043	5.699E-41	3.4194E-40	****	1.00E+00	1.00E+00	9.50E-02
15	Left limb angle asym._min	10	CT	1013	1.02746852	0.01233405	6.9812E-48	4.1887E-47	****	1.00E+00	1.00E+00	9.50E-02
15	Left limb angle asym._min	0	A5	2129	1	0.00975301	1.00E+00	1.00E+00	ns	1.00E+00	1.00E+00	9.50E-02
15	Left limb angle asym._min	2	A5	2206	1.08434133	0.01052016	3.26E-13	1.96E-12	****	1.00E+00	1.00E+00	9.50E-02
15	Left limb angle asym._min	4	A5	1790	1.09053027	0.01466264	6.51E-09	3.91E-08	****	1.00E+00	1.00E+00	9.50E-02
15	Left limb angle asym._min	6	A5	2216	1.33319717	0.01606098	1.86E-36	1.12E-35	****	1.00E+00	1.00E+00	9.50E-02
15	Left limb angle asym._min	8	A5	2809	1.33646205	0.01386274	5.70E-41	3.42E-40	****	1.00E+00	1.00E+00	9.50E-02
15	Left limb angle asym._min	10	A5	2750	1.40511372	0.01486431	6.98E-48	4.19E-47	****	1.00E+00	1.00E+00	9.50E-02
16	Chest 1D velocity_max	0	CT	1784	1	0.02286593	1	1	ns	1.00E+00	1.00E+00	8.28E-01
16	Chest 1D velocity_max	2	CT	1465	1.0342773	0.0262721	1.3657E-17	8.1942E-17	****	1.00E+00	1.00E+00	8.28E-01
16	Chest 1D velocity_max	4	CT	1176	0.93929381	0.0271268	8.5597E-19	5.1358E-18	****	1.00E+00	1.00E+00	8.28E-01
16	Chest 1D velocity_max	6	CT	1089	0.90850039	0.02705245	5.3253E-85	3.1952E-84	****	1.00E+00	1.00E+00	8.28E-01
16	Chest 1D velocity_max	8	CT	1080	0.94936385	0.02599664	2.005E-135	1.203E-134	****	1.00E+00	1.00E+00	8.28E-01
16	Chest 1D velocity_max	10	CT	1023	0.89878786	0.02836015	7.2164E-76	4.3298E-75	****	1.00E+00	1.00E+00	8.28E-01
16	Chest 1D velocity_max	0	A5	2120	1	0.01891912	1.00E+00	1	ns	1.00E+00	1.00E+00	8.28E-01
16	Chest 1D velocity_max	2	A5	2197	0.78579017	0.01588212	1.37E-17	8.1942E-17	****	1.00E+00	1.00E+00	8.28E-01
16	Chest 1D velocity_max	4	A5	1792	0.67671564	0.01589966	8.56E-19	5.1358E-18	****	1.00E+00	1.00E+00	8.28E-01
16	Chest 1D velocity_max	6	A5	2227	0.42284088	0.01049301	5.33E-85	3.1952E-84	****	1.00E+00	1.00E+00	8.28E-01
16	Chest 1D velocity_max	8	A5	2835	0.39927889	0.0086889	2.00E-135	1.20E-134	****	1.00E+00	1.00E+00	8.28E-01
16	Chest 1D velocity_max	10	A5	2798	0.46114719	0.00946406	7.22E-76	4.33E-75	****	1.00E+00	1.00E+00	8.28E-01
17	Leg angle asym._std	0	CT	1764	1	0.01040602	1	1	ns	1.00E+00	1.00E+00	8.41E-01
17	Leg angle asym._std	2	CT	1482	0.91414303	0.01006072	3.8378E-59	2.3027E-58	****	1.00E+00	1.00E+00	8.41E-01
17	Leg angle asym._std	4	CT	1175	0.92635731	0.01156275	3.7517E-65	2.251E-64	****	1.00E+00	1.00E+00	8.41E-01
17	Leg angle asym._std	6	CT	1071	0.969676	0.01342135	4.362E-134	2.617E-133	****	1.00E+00	1.00E+00	8.41E-01
17	Leg angle asym._std	8	CT	1049	0.92481023	0.01240172	1.01E-164	6.06E-164	****	1.00E+00	1.00E+00	8.41E-01
17	Leg angle asym._std	10	CT	1011	1.0044586	0.0130436	4.2818E-95	2.5691E-94	****	1.00E+00	1.00E+00	8.41E-01
17	Leg angle asym._std	0	A5	2085	1	0.01010935	1.00E+00	1.00E+00	ns	1.00E+00	1.00E+00	8.41E-01
17	Leg angle asym._std	2	A5	2197	1.16781174	0.01063661	3.84E-59	2.30E-58	****	1.00E+00	1.00E+00	8.41E-01
17	Leg angle asym._std	4	A5	1791	1.21192475	0.01084767	3.75E-65	2.25E-64	****	1.00E+00	1.00E+00	8.41E-01
17	Leg angle asym._std	6	A5	2196	1.47276828	0.01191898	4.36E-134	2.62E-133	****	1.00E+00	1.00E+00	8.41E-01
17	Leg angle asym._std	8	A5	2838	1.46744419	0.01052975	1.01E-164	6.06E-164	****	1.00E+00	1.00E+00	8.41E-01
17	Leg angle asym._std	10	A5	2793	1.42729756	0.01097794	4.28E-95	2.57E-94	****	1.00E+00	1.00E+00	8.41E-01
18	Feet 2D distance_mean	0	CT	1744	1	0.00349889	1	1	ns	1.00E+00	0.999999992	4.42E-01
18	Feet 2D distance_mean	2	CT	1492	0.9775173	0.00306873	2.367E-116	1.42E-115	****	1.00E+00	0.999999992	4.42E-01
18	Feet 2D distance_mean	4	CT	1172	0.9387463	0.00324482	2.4828E-82	1.4897E-81	****	1.00E+00	0.999999992	4.42E-01
18	Feet 2D distance_mean	6	CT	1073	0.99237449	0.00353962	0.02926285	0.17557708	ns	1.00E+00	0.999999992	4.42E-01
18	Feet 2D distance_mean	8	CT	1059	0.93664005	0.0046818	1.4593E-14	8.7556E-14	****	1.00E+00	0.999999992	4.42E-01
18	Feet 2D distance_mean	10	CT	1012	0.99677668	0.00398505	0.80688421	1	ns	1.00E+00	0.999999992	4.42E-01
18	Feet 2D distance_mean	0	A5	2168	1	0.00436756	1	1	ns	1.00E+00	0.999999992	4.42E-01
18	Feet 2D distance_mean	2	A5	2196	1.10347189	0.00383711	2.367E-116	1.42E-115	****	1.00E+00	0.999999992	4.42E-01
18	Feet 2D distance_mean	4	A5	1794	1.05855926	0.00439731	2.4828E-82	1.4897E-81	****	1.00E+00	0.999999992	4.42E-01
18	Feet 2D distance_mean	6	A5	2196	1.00859486	0.0049025	0.02926285	0.17557708	ns	1.00E+00	0.999999992	4.42E-01
18	Feet 2D distance_mean	8	A5	2833	0.99205306	0.00402376	1.4593E-14	8.7556E-14	****	1.00E+00	0.999999992	4.42E-01
18	Feet 2D distance_mean	10	A5	2782	0.99857704	0.00419831	0.80688421	1	ns	1.00E+00	0.999999992	4.42E-01
19	Anus 1D velocity_std	0	CT	1778	1	0.01242058	1	1	ns	1.00E+00	1	0.541044742
19	Anus 1D velocity_std	2	CT	1465	1.0144533	0.01343447	0.00234294	0.01405762	*	1.00E+00	1	0.541044742
19	Anus 1D velocity_std	4	CT	1160	1.04705276	0.0159897	0.00025932	0.00155592	**	1.00E+00	1	0.541044742
19	Anus 1D velocity_std	6	CT	1073	1.01530915	0.01582641	1.9728E-06	1.1837E-05	****	1.00E+00	1	0.541044742
19	Anus 1D velocity_std	8	CT	1059	1.05358017	0.01712121	2.1672E-10	1.3003E-09	****	1.00E+00	1	0.541044742
19	Anus 1D velocity_std	10	CT	1017	1.07810781	0.01667299	2.14E-17	1.284E-16	****	1.00E+00	1	0.541044742
19	Anus 1D velocity_std	0	A5	2175	1	0.01059296	1	1	ns	1.00E+00	1	0.541044742
19	Anus 1D velocity_std	2	A5	2173	0.96210261	0.01082726	0.00234294	0.01405762	*	1.00E+00	1	0.541044742
19	Anus 1D velocity_std	4	A5	1781	0.97564406	0.01182528	0.00025932	0.00155592	**	1.00E+00	1	0.541044742
19	Anus 1D velocity_std	6	A5	2219	0.92495172	0.01073923	1.9728E-06	1.1837E-05	****	1.00E+00	1	0.541044742
19	Anus 1D velocity_std	8	A5	2831	0.93131688	0.0098472	2.1672E-10	1.3003E-09	****	1.00E+00	1	0.541044742
19	Anus 1D velocity_std	10	A5	2790	0.92115643	0.00930418	2.14E-17	1.284E-16	****	1.00E+00	1	0.541044742
20	Arm angle asym._mean	0	CT	1757	1	0.08158362	1	1	ns	1.00E+00	1.00E+00	3.53E-01
20	Arm angle asym._mean	2	CT	1483	1.10481104	0.08444737	0.76631496	1	ns	1.00E+00	1.00E+00	3.53E-01
20	Arm angle asym._mean	4	CT	1171	0.86703845	0.10008087	5.8356E-05	0.00035014	***	1.00E+00	1.00E+00	3.53E-01
20	Arm angle asym._mean	6	CT	1072	0.76359767	0.09977669	4.2384E-16	2.543E-15	****	1.00E+00	1.00E+00	3.53E-01
20	Arm angle asym._mean	8	CT	1069	0.86723348	0.09895088	0.02573895	0.15443368	ns	1.00E+00	1.00E+00	3.53E-01
20	Arm angle asym._mean	10	CT	1000	1.04261259	0.10529433	1.7553E-16	1.0532E-15	****	1.00E+00	1.00E+00	3.53E-01
20	Arm angle asym._mean	0	A5	2140	1	0.08286873	1.00E+00	1	ns	1.00E+00	1.00E+00	3.53E-01
20	Arm angle asym._mean	2	A5	2206	1.14457362	0.09385351	7.66E-01	1	ns	1.00E+00	1.00E+00	3.53E-01
20	Arm angle asym._mean	4	A5	1800	1.50029181	0.10891253	5.84E-05	3.50E-04	***	1.00E+00	1.00E+00	3.53E-01
20	Arm angle asym._mean	6	A5	2187	2.33742026	0.1256161	4.24E-16	2.54E-15	****	1.00E+00	1.00E+00	3.53E-01
20	Arm angle asym._mean	8	A5	2821	1.28730595	0.10966958	2.57E-02	1.54E-01	ns	1.00E+00	1.00E+00	3.53E-01
20	Arm angle asym._mean	10	A5	2746	2.73695962	0.11744278	1.76E-16	1.05E-15	****	1.00E+00	1.00E+00	3.53E-01

Supplementary Table 5. Statistical summary of the KL divergences for the top 20 features identified by the XGB model comparing NALS and ALS groups. For each group, the KL divergence of each feature relative to its baseline value (10 wk, non-symptomatic age) was computed across the experimental duration (10–20 wk [age]). Each group consisted of 6 sampling sets, each containing 150 randomly selected clips. A two-way ANOVA (group × week) was performed for each feature, followed by weekly t-tests between groups (NALS vs. ALS). The resulting p-values from the weekly t-tests were corrected for multiple comparisons using the Bonferroni method. Source data are provided in the Source Data file (See S_Figure 20).

Rank	Feature name	group	Week	n	Mean	SEM	raw_p	corrected_p	significant	ANOVA_p_Week	ANOVA_p_Group	ANOVA_p_Interaction
1	Hands 3D distance_mean	ALS	0	6	0	0		1	ns	1.05E-11	8.78E-02	8.00166E-05
1	Hands 3D distance_mean	NALS	0	6	0	0		1	ns	1.05E-11	8.78E-02	8.00166E-05
1	Hands 3D distance_mean	ALS	2	6	0.29477218	0.03925967	0.02524964	0.15149783	ns	1.05E-11	8.78E-02	8.00166E-05
1	Hands 3D distance_mean	NALS	2	6	0.17497459	0.02316707	0.02524964	0.15149783	ns	1.05E-11	8.78E-02	8.00166E-05
1	Hands 3D distance_mean	ALS	4	6	0.16548393	0.01416023	0.11949613	0.71697676	ns	1.05E-11	8.78E-02	8.00166E-05
1	Hands 3D distance_mean	NALS	4	6	0.24049446	0.04172261	0.11949613	0.71697676	ns	1.05E-11	8.78E-02	8.00166E-05
1	Hands 3D distance_mean	ALS	6	6	0.18647739	0.03180216	0.14991381	0.89948286	ns	1.05E-11	8.78E-02	8.00166E-05
1	Hands 3D distance_mean	NALS	6	6	0.12891805	0.01872714	0.14991381	0.89948286	ns	1.05E-11	8.78E-02	8.00166E-05
1	Hands 3D distance_mean	ALS	8	6	0.15172574	0.02822428	0.07520344	0.45122066	ns	1.05E-11	8.78E-02	8.00166E-05
1	Hands 3D distance_mean	NALS	8	6	0.24456502	0.03728465	0.07520344	0.45122066	ns	1.05E-11	8.78E-02	8.00166E-05
1	Hands 3D distance_mean	ALS	10	6	0.33935222	0.03386703	0.00878057	0.05268339	ns	1.05E-11	8.78E-02	8.00166E-05
1	Hands 3D distance_mean	NALS	10	6	0.17390332	0.0380928	0.00878057	0.05268339	ns	1.05E-11	8.78E-02	8.00166E-05
2	Motion duration_episode	ALS	0	6	0	0		1	ns	6.82E-28	1.79724E-11	1.50823E-06
2	Motion duration_episode	NALS	0	6	0	0		1	ns	6.82E-28	1.79724E-11	1.50823E-06
2	Motion duration_episode	ALS	2	6	0.05523906	0.00871457	0.68030263	1	ns	6.82E-28	1.79724E-11	1.50823E-06
2	Motion duration_episode	NALS	2	6	0.04788315	0.01498523	0.68030263	1	ns	6.82E-28	1.79724E-11	1.50823E-06
2	Motion duration_episode	ALS	4	6	0.41884256	0.03404672	0.09208465	0.55250791	ns	6.82E-28	1.79724E-11	1.50823E-06
2	Motion duration_episode	NALS	4	6	0.55731567	0.06607893	0.09208465	0.55250791	ns	6.82E-28	1.79724E-11	1.50823E-06
2	Motion duration_episode	ALS	6	6	0.04392515	0.00428502	4.6008E-06	2.7605E-05	****	6.82E-28	1.79724E-11	1.50823E-06
2	Motion duration_episode	NALS	6	6	0.15476994	0.01170247	4.6008E-06	2.7605E-05	****	6.82E-28	1.79724E-11	1.50823E-06
2	Motion duration_episode	ALS	8	6	0.12163967	0.01731961	2.3526E-06	1.4116E-05	****	6.82E-28	1.79724E-11	1.50823E-06
2	Motion duration_episode	NALS	8	6	0.35059822	0.01646972	2.3526E-06	1.4116E-05	****	6.82E-28	1.79724E-11	1.50823E-06
2	Motion duration_episode	ALS	10	6	0.08611061	0.00327844	6.9736E-06	4.1842E-05	****	6.82E-28	1.79724E-11	1.50823E-06
2	Motion duration_episode	NALS	10	6	0.32162771	0.02754786	6.9736E-06	4.1842E-05	****	6.82E-28	1.79724E-11	1.50823E-06
3	Body 2D length_min	ALS	0	6	0	0		1	ns	2.06E-17	1.00E-25	1.71E-14
3	Body 2D length_min	NALS	0	6	0	0		1	ns	2.06E-17	1.00E-25	1.71E-14
3	Body 2D length_min	ALS	2	6	0.34081236	0.03584905	1.4861E-05	8.9167E-05	****	2.06E-17	1.00E-25	1.71E-14
3	Body 2D length_min	NALS	2	6	0.05428077	0.00824195	1.4861E-05	8.9167E-05	****	2.06E-17	1.00E-25	1.71E-14
3	Body 2D length_min	ALS	4	6	0.23385881	0.02358315	2.52E-04	1.51E-03	**	2.06E-17	1.00E-25	1.71E-14
3	Body 2D length_min	NALS	4	6	0.10008738	0.00541491	2.52E-04	1.51E-03	**	2.06E-17	1.00E-25	1.71E-14
3	Body 2D length_min	ALS	6	6	0.41289836	0.03427806	3.77E-06	2.26E-05	****	2.06E-17	1.00E-25	1.71E-14
3	Body 2D length_min	NALS	6	6	0.08441036	0.01141276	3.77E-06	2.26E-05	****	2.06E-17	1.00E-25	1.71E-14
3	Body 2D length_min	ALS	8	6	0.29927264	0.02345884	0.00207545	0.01245272	*	2.06E-17	1.00E-25	1.71E-14
3	Body 2D length_min	NALS	8	6	0.16576221	0.02235048	0.00207545	0.01245272	*	2.06E-17	1.00E-25	1.71E-14
3	Body 2D length_min	ALS	10	6	0.45229024	0.0336092	1.47E-07	8.82E-07	****	2.06E-17	1.00E-25	1.71E-14
3	Body 2D length_min	NALS	10	6	0.01528039	0.0041456	1.47E-07	8.82E-07	****	2.06E-17	1.00E-25	1.71E-14
4	Tail angle_mean	ALS	0	6	0	0		1	ns	1.67E-14	4.92E-03	4.60E-11
4	Tail angle_mean	NALS	0	6	0	0		1	ns	1.67E-14	4.92E-03	4.60E-11
4	Tail angle_mean	ALS	2	6	0.50944576	0.0749561	0.0154873	0.09292381	ns	1.67E-14	4.92E-03	4.60E-11
4	Tail angle_mean	NALS	2	6	0.2579788	0.04282723	0.0154873	0.09292381	ns	1.67E-14	4.92E-03	4.60E-11
4	Tail angle_mean	ALS	4	6	0.24943636	0.03421283	0.90517021	1	ns	1.67E-14	4.92E-03	4.60E-11
4	Tail angle_mean	NALS	4	6	0.24303598	0.03966443	0.90517021	1	ns	1.67E-14	4.92E-03	4.60E-11
4	Tail angle_mean	ALS	6	6	0.67456466	0.05470606	6.00E-06	3.60E-05	****	1.67E-14	4.92E-03	4.60E-11
4	Tail angle_mean	NALS	6	6	0.1446031	0.02782626	6.00E-06	3.60E-05	****	1.67E-14	4.92E-03	4.60E-11
4	Tail angle_mean	ALS	8	6	0.33051161	0.05161464	2.45E-02	0.14676044	ns	1.67E-14	4.92E-03	4.60E-11
4	Tail angle_mean	NALS	8	6	0.58206128	0.07981544	2.45E-02	0.14676044	ns	1.67E-14	4.92E-03	4.60E-11
4	Tail angle_mean	ALS	10	6	0.1690129	0.01679103	7.79E-02	4.67E-01	ns	1.67E-14	4.92E-03	4.60E-11
4	Tail angle_mean	NALS	10	6	0.24733419	0.03617091	7.79E-02	4.67E-01	ns	1.67E-14	4.92E-03	4.60E-11
5	Arm angle asym._std	ALS	0	6	0	0		1	ns	3.85E-09	1.01E-03	4.01E-02
5	Arm angle asym._std	NALS	0	6	0	0		1	ns	3.85E-09	1.01E-03	4.01E-02
5	Arm angle asym._std	ALS	2	6	0.11163662	0.01452356	0.00175145	0.01050868	*	3.85E-09	1.01E-03	4.01E-02
5	Arm angle asym._std	NALS	2	6	0.0372696	0.00992761	0.00175145	0.01050868	*	3.85E-09	1.01E-03	4.01E-02
5	Arm angle asym._std	ALS	4	6	0.17122284	0.02334808	0.61767012	1	ns	3.85E-09	1.01E-03	4.01E-02
5	Arm angle asym._std	NALS	4	6	0.14810527	0.03832734	0.61767012	1	ns	3.85E-09	1.01E-03	4.01E-02
5	Arm angle asym._std	ALS	6	6	0.11231627	0.03733435	1.13E-01	6.80E-01	ns	3.85E-09	1.01E-03	4.01E-02
5	Arm angle asym._std	NALS	6	6	0.04525491	0.00994064	1.13E-01	6.80E-01	ns	3.85E-09	1.01E-03	4.01E-02
5	Arm angle asym._std	ALS	8	6	0.09907821	0.01781828	5.03E-01	1.00E+00	ns	3.85E-09	1.01E-03	4.01E-02
5	Arm angle asym._std	NALS	8	6	0.11663689	0.01789293	5.03E-01	1.00E+00	ns	3.85E-09	1.01E-03	4.01E-02
5	Arm angle asym._std	ALS	10	6	0.17405952	0.01624253	2.25E-03	1.35E-02	*	3.85E-09	1.01E-03	4.01E-02
5	Arm angle asym._std	NALS	10	6	0.07578548	0.0178749	2.25E-03	1.35E-02	*	3.85E-09	1.01E-03	4.01E-02
6	Motion interval_episode	ALS	0	6	0	0		1	ns	1.89E-19	3.40904E-10	1.11048E-18
6	Motion interval_episode	NALS	0	6	0	0		1	ns	1.89E-19	3.40904E-10	1.11048E-18
6	Motion interval_episode	ALS	2	6	0.57866976	0.08120349	0.00015079	0.00090471	***	1.89E-19	3.40904E-10	1.11048E-18
6	Motion interval_episode	NALS	2	6	0.09219923	0.0141898	0.00015079	0.00090471	***	1.89E-19	3.40904E-10	1.11048E-18
6	Motion interval_episode	ALS	4	6	0.04614524	0.00953707	0.00043645	0.00261867	**	1.89E-19	3.40904E-10	1.11048E-18
6	Motion interval_episode	NALS	4	6	0.12746286	0.01261477	0.00043645	0.00261867	**	1.89E-19	3.40904E-10	1.11048E-18
6	Motion interval_episode	ALS	6	6	0.94316796	0.10627281	1.8714E-05	0.00011229	***	1.89E-19	3.40904E-10	1.11048E-18
6	Motion interval_episode	NALS	6	6	0.12988464	0.01422652	1.8714E-05	0.00011229	***	1.89E-19	3.40904E-10	1.11048E-18
6	Motion interval_episode	ALS	8	6	0.2007618	0.00852867	0.00053534	0.00321204	**	1.89E-19	3.40904E-10	1.11048E-18
6	Motion interval_episode	NALS	8	6	0.37545146	0.03386281	0.00053534	0.00321204	**	1.89E-19	3.40904E-10	1.11048E-18
6	Motion interval_episode	ALS	10	6	0.10931975	0.02404702	0.27446847	1	ns	1.89E-19	3.40904E-10	1.11048E-18
6	Motion interval_episode	NALS	10	6	0.0764879	0.01510203	0.27446847	1	ns	1.89E-19	3.40904E-10	1.11048E-18
7	Body angle_min	ALS	0	6	0	0		1	ns	2.08809E-13	4.39E-08	0.001652424
7	Body angle_min	NALS	0	6	0	0		1	ns	2.08809E-13	4.39E-08	0.001652424
7	Body angle_min	ALS	2	6	0.25973726	0.03805759	0.16137865	0.96827187	ns	2.08809E-13	4.39E-08	0.001652424
7	Body angle_min	NALS	2	6	0.18672403	0.02970353	0.16137865	0.96827187	ns	2.08809E-13	4.39E-08	0.001652424
7	Body angle_min	ALS	4	6	0.29383061	0.04461753	0.57377618	1	ns	2.08809E-13	4.39E-08	0.001652424
7	Body angle_min	NALS	4	6	0.25166909	0.05715069	0.57377618	1	ns	2.08809E-13	4.39E-08	0.001652424
7	Body angle_min	ALS	6	6	0.33273807	0.02562017	0.0012464	0.00747841	**	2.08809E-13	4.39E-08	0.001652424
7	Body angle_min	NALS	6	6	0.15345771	0.0311605	0.0012464	0.00747841	**	2.08809E-13	4.39E-08	0.001652424
7	Body angle_min	ALS	8	6	0.44141468	0.03884845	0.00806747	0.0484048	*	2.08809E-13	4.39E-08	0.001652424
7	Body angle_min	NALS	8	6	0.25958839	0.03916642	0.00806747	0.0484048	*	2.08809E-13	4.39E-08	0.001652424
7	Body angle_min	ALS	10	6	0.42481561	0.03683686	0.00011124	0.00066744	***	2.08809E-13	4.39E-08	0.001652424
7	Body angle_min	NALS	10	6	0.15409785	0.02436254	0.00011124	0.00066744	***	2.08809E-13	4.39E-08	0.001652424
8	Feet 2D distance_max	ALS	0	6	0	0		1	ns	1.99E-24	2.90E-25	5.26E-15
8	Feet 2D distance_max	NALS	0	6	0	0		1	ns	1.99E-24	2.90E-25	5.26E-15
8	Feet 2D distance_max	ALS	2	6	0.25192894	0.03920875	0.22656741	1	ns	1.99E-24	2.90E-25	5.26E-15
8	Feet 2D distance_max	NALS	2	6	0.19782187	0.01502965	0.22656741	1	ns	1.99E-24	2.90E-25	5.26E-15
8	Feet 2D distance_max	ALS	4	6	0.67097317	0.0582002	8.9696E-06	5.3818E-05	****	1.99E-24	2.90E-25	5.26E-15
8	Feet 2D distance_max	NALS	4	6	0.14083052	0.02721313	8.9696E-06	5.3818E-05	****	1.99E-24	2.90E-25	5.26E-15
8	Feet 2D distance_max	ALS	6	6	1.02687058	0.07539651	1.6682E-06	1.0009E-05	****	1.99E-24	2.90E-25	5.26E-15
8	Feet 2D distance_max	NALS	6	6	0.23580453	0.02528969	1.6682E-06	1.0009E-05	****	1.99E-24	2.90E-25	5.26E-15
8	Feet 2D distance_max	ALS	8	6	0.97866533	0.04464448	3.1515E-07	1.8909E-06	****	1.99E-24	2.90E-25	5.26E-15
8	Feet 2D distance_max	NALS	8	6	0.31891464	0.0328442	3.1515E-07	1.8909E-06	****	1.99E-24	2.90E-25	5.26E-15
8	Feet 2D distance_max	ALS	10	6	0.83741499	0.06686554	4.93E-05	2.96E-04	***	1.99E-24	2.90E-25	5.26E-15
8	Feet 2D distance_max	NALS	10	6	0.30252347	0.0421235	4.93E-05	2.96E-04	***	1.99E-24	2.90E-25	5.26E-15
9	Neck 3D velocity_std	ALS	0	6	0	0		1	ns	3.57E-22	2.78E-07	6.52E-12
9	Neck 3D velocity_std	NALS	0	6	0	0		1	ns	3.57E-22	2.78E-07	6.52E-12
9	Neck 3D velocity_std	ALS	2	6	0.13878081	0.01604262	0.00014306	0.00085838	***	3.57E-22	2.78E-07	6.52E-12
9	Neck 3D velocity_std	NALS	2	6	0.03679226	0.00611491	0.00014306	0.00085838	***	3.57E-22	2.78E-07	6.52E-12
9	Neck 3D velocity_std	ALS	4	6	0.25040807	0.02340489	0.63431936	1	ns	3.57E-22	2.78E-07	6.52E-12
9	Neck 3D velocity_std	NALS	4	6	0.27788926	0.0508958	0.63431936	1	ns	3.57E-22	2.78E-07	6.52E-12
9	Neck 3D velocity_std	ALS	6	6	0.09012832	0.01747463	2.01E-01	1.00E+00	ns	3.57E-22	2.78E-07	6.52E-12
9	Neck 3D velocity_std	NALS	6	6	0.12863375	0.02201317	2.01E-01	1.00E+00	ns	3.57E-22	2.78E-07	6.52E-12
9	Neck 3D velocity_std	ALS	8	6	0.23784279	0.01882023	8.00E-02	0.4801099	ns	3.57E-22	2.78E-07	6.52E-12
9	Neck 3D velocity_std	NALS	8	6	0.16710423	0.03105667	8.00E-02	0.4801099	ns	3.57E-22	2.78E-07	6.52E-12
9	Neck 3D velocity_std	ALS	10	6	0.54241326	0.02496469	8.4123E-07	5.0474E-06	****	3.57E-22	2.78E-07	6.52E-12
9	Neck 3D velocity_std	NALS	10	6	0.17478212	0.02353654	8.4123E-07	5.0474E-06	****	3.57E-22	2.78E-07	6.52E-12
10	Feet 2D distance_std	ALS	0	6	0	0		1	ns	1.71E-16	1.09E-23	4.42E-10
10	Feet 2D distance_std	NALS	0	6	0	0		1	ns	1.71E-16	1.09E-23	4.42E-10
10	Feet 2D distance_std	ALS	2	6	0.24325513	0.03993271	0.00359539	0.02157233	*	1.71E-16	1.09E-23	4.42E-10
10	Feet 2D distance_std	NALS	2	6	0.08769973	0.00989378	0.00359539	0.02157233	*	1.71E-16	1.09E-23	4.42E-10
10	Feet 2D distance_std	ALS	4	6	0.34191365	0.04048262	8.9958E-05	0.00053975	***	1.71E-16	1.09E-23	4.42E-10
10	Feet 2D distance_std	NALS	4	6	0.07341867	0.01348445	8.9958E-05	0.00053975	***	1.71E-16	1.09E-23	4.42E-10
10	Feet 2D distance_std	ALS	6	6	0.38620313	0.03456013	2.78E-05	1.67E-04	***	1.71E-16	1.09E-23	4.42E-10
10	Feet 2D distance_std	NALS	6	6	0.10002792	0.01915252	2.78E-05	1.67E-04	***	1.71E-16	1.09E-23	4.42E-10
10	Feet 2D distance_std	ALS	8	6	0.36304067	0.02471724	3.5348E-05	0.00021209	***	1.71E-16	1.09E-23	4.42E-10
10	Feet 2D distance_std	NALS	8	6	0.11731451	0.02463476	3.5348E-05	0.00021209	***	1.71E-16	1.09E-23	4.42E-10
10	Feet 2D distance_std	ALS	10	6	0.484339	0.02435	3.02E-08	1.81E-07	****	1.71E-16	1.09E-23	4.42E-10
10	Feet 2D distance_std	NALS	10	6	0.07757371	0.01097559	3.02E-08	1.81E-07	****	1.71E-16	1.09E-23	4.42E-10

Rank	Feature name	group	Week	n	Mean	SEM	raw_p	corrected_p	significant	ANOVA_p_Week	ANOVA_p_Group	ANOVA_p_Interaction
11	Chest 3D velocity_mean	ALS	0	6	0	0		1	ns	3.15E-35	1.35E-15	5.76E-25
11	Chest 3D velocity_mean	NALS	0	6	0	0		1	ns	3.15E-35	1.35E-15	5.76E-25
11	Chest 3D velocity_mean	ALS	2	6	0.34926148	0.02886476	0.13304847	0.79829082	ns	3.15E-35	1.35E-15	5.76E-25
11	Chest 3D velocity_mean	NALS	2	6	0.42361624	0.03513436	0.13304847	0.79829082	ns	3.15E-35	1.35E-15	5.76E-25
11	Chest 3D velocity_mean	ALS	4	6	0.53293559	0.0337395	0.51587971	1	ns	3.15E-35	1.35E-15	5.76E-25
11	Chest 3D velocity_mean	NALS	4	6	0.495618	0.04394936	0.51587971	1	ns	3.15E-35	1.35E-15	5.76E-25
11	Chest 3D velocity_mean	ALS	6	6	0.30023675	0.02860105	0.15456207	0.9273724	ns	3.15E-35	1.35E-15	5.76E-25
11	Chest 3D velocity_mean	NALS	6	6	0.36984622	0.03499823	0.15456207	0.9273724	ns	3.15E-35	1.35E-15	5.76E-25
11	Chest 3D velocity_mean	ALS	8	6	0.61177163	0.03699785	2.87E-05	0.00017216	***	3.15E-35	1.35E-15	5.76E-25
11	Chest 3D velocity_mean	NALS	8	6	0.271112	0.02931685	2.87E-05	0.00017216	***	3.15E-35	1.35E-15	5.76E-25
11	Chest 3D velocity_mean	ALS	10	6	1.38322205	0.0372135	2.07E-09	1.2403E-08	****	3.15E-35	1.35E-15	5.76E-25
11	Chest 3D velocity_mean	NALS	10	6	0.46590012	0.02651008	2.07E-09	1.2403E-08	****	3.15E-35	1.35E-15	5.76E-25
12	U-body 3D length_min	ALS	0	6	0	0		1	ns	3.92E-14	1.19E-24	1.30E-10
12	U-body 3D length_min	NALS	0	6	0	0		1	ns	3.92E-14	1.19E-24	1.30E-10
12	U-body 3D length_min	ALS	2	6	0.39949173	0.04723854	4.0931E-05	0.00024559	***	3.92E-14	1.19E-24	1.30E-10
12	U-body 3D length_min	NALS	2	6	0.06143787	0.01244805	4.0931E-05	0.00024559	***	3.92E-14	1.19E-24	1.30E-10
12	U-body 3D length_min	ALS	4	6	0.19172142	0.02602317	0.000719	0.00431399	**	3.92E-14	1.19E-24	1.30E-10
12	U-body 3D length_min	NALS	4	6	0.06293179	0.00643627	0.000719	0.00431399	**	3.92E-14	1.19E-24	1.30E-10
12	U-body 3D length_min	ALS	6	6	0.30557049	0.01915858	2.59E-07	1.5523E-06	****	3.92E-14	1.19E-24	1.30E-10
12	U-body 3D length_min	NALS	6	6	0.05746862	0.00703671	2.59E-07	1.5523E-06	****	3.92E-14	1.19E-24	1.30E-10
12	U-body 3D length_min	ALS	8	6	0.35934816	0.04474645	3.6562E-05	0.00021937	***	3.92E-14	1.19E-24	1.30E-10
12	U-body 3D length_min	NALS	8	6	0.04520711	0.00201223	3.6562E-05	0.00021937	***	3.92E-14	1.19E-24	1.30E-10
12	U-body 3D length_min	ALS	10	6	0.37071898	0.02900213	5.3015E-07	3.1809E-06	****	3.92E-14	1.19E-24	1.30E-10
12	U-body 3D length_min	NALS	10	6	0.03714926	0.00603463	5.3015E-07	3.1809E-06	****	3.92E-14	1.19E-24	1.30E-10
13	Right limb angle asym._min	ALS	0	6	0	0		1	ns	2.97E-19	2.09E-12	9.72E-06
13	Right limb angle asym._min	NALS	0	6	0	0		1	ns	2.97E-19	2.09E-12	9.72E-06
13	Right limb angle asym._min	ALS	2	6	0.10063516	0.00913912	0.18518961	1	ns	2.97E-19	2.09E-12	9.72E-06
13	Right limb angle asym._min	NALS	2	6	0.07139725	0.01840303	0.18518961	1	ns	2.97E-19	2.09E-12	9.72E-06
13	Right limb angle asym._min	ALS	4	6	0.16915763	0.01381195	0.0001848	0.00110882	**	2.97E-19	2.09E-12	9.72E-06
13	Right limb angle asym._min	NALS	4	6	0.05447599	0.01438122	0.0001848	0.00110882	**	2.97E-19	2.09E-12	9.72E-06
13	Right limb angle asym._min	ALS	6	6	0.15451532	0.01015566	0.01738824	0.10432941	ns	2.97E-19	2.09E-12	9.72E-06
13	Right limb angle asym._min	NALS	6	6	0.08884962	0.02072363	0.01738824	0.10432941	ns	2.97E-19	2.09E-12	9.72E-06
13	Right limb angle asym._min	ALS	8	6	0.27770197	0.00914972	0.00281342	0.01688051	*	2.97E-19	2.09E-12	9.72E-06
13	Right limb angle asym._min	NALS	8	6	0.14894173	0.03144694	0.00281342	0.01688051	*	2.97E-19	2.09E-12	9.72E-06
13	Right limb angle asym._min	ALS	10	6	0.30609512	0.01949928	2.35E-04	1.41E-03	**	2.97E-19	2.09E-12	9.72E-06
13	Right limb angle asym._min	NALS	10	6	0.12463794	0.02604565	2.35E-04	1.41E-03	**	2.97E-19	2.09E-12	9.72E-06
14	Hands 3D distance_max	ALS	0	6	0	0		1	ns	2.82E-13	1.28E-01	4.98E-08
14	Hands 3D distance_max	NALS	0	6	0	0		1	ns	2.82E-13	1.28E-01	4.98E-08
14	Hands 3D distance_max	ALS	2	6	0.04373834	0.01360037	0.17406094	1	ns	2.82E-13	1.28E-01	4.98E-08
14	Hands 3D distance_max	NALS	2	6	0.07968728	0.02045635	0.17406094	1	ns	2.82E-13	1.28E-01	4.98E-08
14	Hands 3D distance_max	ALS	4	6	0.07638522	0.01321977	0.77529513	1	ns	2.82E-13	1.28E-01	4.98E-08
14	Hands 3D distance_max	NALS	4	6	0.08425011	0.02333086	0.77529513	1	ns	2.82E-13	1.28E-01	4.98E-08
14	Hands 3D distance_max	ALS	6	6	0.03310328	0.00528623	0.04509476	0.27056858	ns	2.82E-13	1.28E-01	4.98E-08
14	Hands 3D distance_max	NALS	6	6	0.06045962	0.01071889	0.04509476	0.27056858	ns	2.82E-13	1.28E-01	4.98E-08
14	Hands 3D distance_max	ALS	8	6	0.04964546	0.00938545	0.73741659	1	ns	2.82E-13	1.28E-01	4.98E-08
14	Hands 3D distance_max	NALS	8	6	0.05600817	0.0158909	0.73741659	1	ns	2.82E-13	1.28E-01	4.98E-08
14	Hands 3D distance_max	ALS	10	6	0.23161635	0.0253208	1.92E-04	0.00114955	**	2.82E-13	1.28E-01	4.98E-08
14	Hands 3D distance_max	NALS	10	6	0.07631753	0.00973014	1.92E-04	0.00114955	**	2.82E-13	1.28E-01	4.98E-08
15	Left limb angle asym._min	ALS	0	6	0	0		1	ns	2.27E-07	2.60E-01	9.31E-01
15	Left limb angle asym._min	NALS	0	6	0	0		1	ns	2.27E-07	2.60E-01	9.31E-01
15	Left limb angle asym._min	ALS	2	6	0.10053941	0.01368944	0.40101776	1	ns	2.27E-07	2.60E-01	9.31E-01
15	Left limb angle asym._min	NALS	2	6	0.15100786	0.05588865	0.40101776	1	ns	2.27E-07	2.60E-01	9.31E-01
15	Left limb angle asym._min	ALS	4	6	0.18932593	0.02288421	0.8995361	1	ns	2.27E-07	2.60E-01	9.31E-01
15	Left limb angle asym._min	NALS	4	6	0.1823999	0.04834344	0.8995361	1	ns	2.27E-07	2.60E-01	9.31E-01
15	Left limb angle asym._min	ALS	6	6	0.20083742	0.02264782	8.49E-01	1.00E+00	ns	2.27E-07	2.60E-01	9.31E-01
15	Left limb angle asym._min	NALS	6	6	0.21200636	0.05257953	8.49E-01	1.00E+00	ns	2.27E-07	2.60E-01	9.31E-01
15	Left limb angle asym._min	ALS	8	6	0.17166985	0.0138705	3.82E-01	1.00E+00	ns	2.27E-07	2.60E-01	9.31E-01
15	Left limb angle asym._min	NALS	8	6	0.21947521	0.05039694	3.82E-01	1.00E+00	ns	2.27E-07	2.60E-01	9.31E-01
15	Left limb angle asym._min	ALS	10	6	0.15531178	0.02160939	4.31E-01	1.00E+00	ns	2.27E-07	2.60E-01	9.31E-01
15	Left limb angle asym._min	NALS	10	6	0.18481404	0.02876275	4.31E-01	1.00E+00	ns	2.27E-07	2.60E-01	9.31E-01
16	Chest 1D velocity_max	ALS	0	6	0	0		1	ns	7.82E-25	2.45E-13	1.60E-07
16	Chest 1D velocity_max	NALS	0	6	0	0		1	ns	7.82E-25	2.45E-13	1.60E-07
16	Chest 1D velocity_max	ALS	2	6	1.53380764	0.14636445	0.05161611	0.30969663	ns	7.82E-25	2.45E-13	1.60E-07
16	Chest 1D velocity_max	NALS	2	6	1.16185391	0.08318799	0.05161611	0.30969663	ns	7.82E-25	2.45E-13	1.60E-07
16	Chest 1D velocity_max	ALS	4	6	1.61896055	0.13746273	0.23437184	1	ns	7.82E-25	2.45E-13	1.60E-07
16	Chest 1D velocity_max	NALS	4	6	1.42199883	0.0729826	0.23437184	1	ns	7.82E-25	2.45E-13	1.60E-07
16	Chest 1D velocity_max	ALS	6	6	1.60969959	0.1605271	6.56E-03	0.03936465	*	7.82E-25	2.45E-13	1.60E-07
16	Chest 1D velocity_max	NALS	6	6	1.01406581	0.06771228	6.56E-03	0.03936465	*	7.82E-25	2.45E-13	1.60E-07
16	Chest 1D velocity_max	ALS	8	6	1.89664273	0.13507951	3.19E-04	0.00191686	**	7.82E-25	2.45E-13	1.60E-07
16	Chest 1D velocity_max	NALS	8	6	1.08900733	0.06682647	3.19E-04	0.00191686	**	7.82E-25	2.45E-13	1.60E-07
16	Chest 1D velocity_max	ALS	10	6	2.458658	0.11725093	2.13E-06	1.28E-05	****	7.82E-25	2.45E-13	1.60E-07
16	Chest 1D velocity_max	NALS	10	6	1.127333	0.07177783	2.13E-06	1.28E-05	****	7.82E-25	2.45E-13	1.60E-07
17	Leg angle asym._std	ALS	0	6	0	0		1	ns	2.07E-06	6.52E-02	2.62E-01
17	Leg angle asym._std	NALS	0	6	0	0		1	ns	2.07E-06	6.52E-02	2.62E-01
17	Leg angle asym._std	ALS	2	6	0.10410722	0.01622025	0.95221432	1	ns	2.07E-06	6.52E-02	2.62E-01
17	Leg angle asym._std	NALS	2	6	0.1018815	0.03238733	0.95221432	1	ns	2.07E-06	6.52E-02	2.62E-01
17	Leg angle asym._std	ALS	4	6	0.06712979	0.01469018	0.37543383	1	ns	2.07E-06	6.52E-02	2.62E-01
17	Leg angle asym._std	NALS	4	6	0.04526712	0.01842914	0.37543383	1	ns	2.07E-06	6.52E-02	2.62E-01
17	Leg angle asym._std	ALS	6	6	0.09238392	0.00916514	2.47E-04	1.48E-03	**	2.07E-06	6.52E-02	2.62E-01
17	Leg angle asym._std	NALS	6	6	0.0330233	0.00554408	2.47E-04	1.48E-03	**	2.07E-06	6.52E-02	2.62E-01
17	Leg angle asym._std	ALS	8	6	0.08189631	0.01647977	2.22E-01	1.00E+00	ns	2.07E-06	6.52E-02	2.62E-01
17	Leg angle asym._std	NALS	8	6	0.05239867	0.01552806	2.22E-01	1.00E+00	ns	2.07E-06	6.52E-02	2.62E-01
17	Leg angle asym._std	ALS	10	6	0.05344609	0.00784268	6.10E-01	1.00E+00	ns	2.07E-06	6.52E-02	2.62E-01
17	Leg angle asym._std	NALS	10	6	0.06455291	0.01958315	6.10E-01	1.00E+00	ns	2.07E-06	6.52E-02	2.62E-01
18	Feet 2D distance_mean	ALS	0	6	0	0		1	ns	2.15E-20	5.62328E-05	6.41E-08
18	Feet 2D distance_mean	NALS	0	6	0	0		1	ns	2.15E-20	5.62328E-05	6.41E-08
18	Feet 2D distance_mean	ALS	2	6	0.18373107	0.01923952	0.04971708	0.29830246	ns	2.15E-20	5.62328E-05	6.41E-08
18	Feet 2D distance_mean	NALS	2	6	0.12600594	0.01729216	0.04971708	0.29830246	ns	2.15E-20	5.62328E-05	6.41E-08
18	Feet 2D distance_mean	ALS	4	6	0.13453338	0.02062314	0.01232953	0.07397715	ns	2.15E-20	5.62328E-05	6.41E-08
18	Feet 2D distance_mean	NALS	4	6	0.0673034	0.00785794	0.01232953	0.07397715	ns	2.15E-20	5.62328E-05	6.41E-08
18	Feet 2D distance_mean	ALS	6	6	0.13038276	0.00659416	0.02274854	0.13649123	ns	2.15E-20	5.62328E-05	6.41E-08
18	Feet 2D distance_mean	NALS	6	6	0.08518833	0.01546126	0.02274854	0.13649123	ns	2.15E-20	5.62328E-05	6.41E-08
18	Feet 2D distance_mean	ALS	8	6	0.21084338	0.01069021	6.785E-05	0.0004071	***	2.15E-20	5.62328E-05	6.41E-08
18	Feet 2D distance_mean	NALS	8	6	0.11474721	0.01017018	6.785E-05	0.0004071	***	2.15E-20	5.62328E-05	6.41E-08
18	Feet 2D distance_mean	ALS	10	6	0.13415309	0.00974487	0.00099351	0.00596106	**	2.15E-20	5.62328E-05	6.41E-08
18	Feet 2D distance_mean	NALS	10	6	0.20993936	0.01332369	0.00099351	0.00596106	**	2.15E-20	5.62328E-05	6.41E-08
19	Anus 1D velocity_std	ALS	0	6	0	0		1	ns	1.25E-23	1.69543E-24	2.76862E-18
19	Anus 1D velocity_std	NALS	0	6	0	0		1	ns	1.25E-23	1.69543E-24	2.76862E-18
19	Anus 1D velocity_std	ALS	2	6	0.29138319	0.03261651	0.02408841	0.14453044	ns	1.25E-23	1.69543E-24	2.76862E-18
19	Anus 1D velocity_std	NALS	2	6	0.18302801	0.02452039	0.02408841	0.14453044	ns	1.25E-23	1.69543E-24	2.76862E-18
19	Anus 1D velocity_std	ALS	4	6	0.28935851	0.04129941	0.01365441	0.08192648	ns	1.25E-23	1.69543E-24	2.76862E-18
19	Anus 1D velocity_std	NALS	4	6	0.15425664	0.01845946	0.01365441	0.08192648	ns	1.25E-23	1.69543E-24	2.76862E-18
19	Anus 1D velocity_std	ALS	6	6	0.36728983	0.04264595	5.209E-05	0.00031254	***	1.25E-23	1.69543E-24	2.76862E-18
19	Anus 1D velocity_std	NALS	6	6	0.06306153	0.01512013	5.209E-05	0.00031254	***	1.25E-23	1.69543E-24	2.76862E-18
19	Anus 1D velocity_std	ALS	8	6	0.52038691	0.04829336	5.8604E-06	3.5163E-05	****	1.25E-23	1.69543E-24	2.76862E-18
19	Anus 1D velocity_std	NALS	8	6	0.081981	0.01524312	5.8604E-06	3.5163E-05	****	1.25E-23	1.69543E-24	2.76862E-18
19	Anus 1D velocity_std	ALS	10	6	0.90196825	0.04073917	1.5221E-08	9.1329E-08	****	1.25E-23	1.69543E-24	2.76862E-18
19	Anus 1D velocity_std	NALS	10	6	0.16988559	0.01857019	1.5221E-08	9.1329E-08	****	1.25E-23	1.69543E-24	2.76862E-18
20	Arm angle asym._mean	ALS	0	6	0	0		1	ns	1.12E-07	7.38E-01	1.67E-02
20	Arm angle asym._mean	NALS	0	6	0	0		1	ns	1.12E-07	7.38E-01	1.67E-02
20	Arm angle asym._mean	ALS	2	6	0.16279346	0.03263139	0.62484179	1	ns	1.12E-07	7.38E-01	1.67E-02
20	Arm angle asym._mean	NALS	2	6	0.14251678	0.02346437	0.62484179	1	ns	1.12E-07	7.38E-01	1.67E-02
20	Arm angle asym._mean	ALS	4	6	0.17206808	0.02991577	0.63682115	1	ns	1.12E-07	7.38E-01	1.67E-02
20	Arm angle asym._mean	NALS	4	6	0.14733447	0.04105411	0.63682115	1	ns	1.12E-07	7.38E-01	1.67E-02
20	Arm angle asym._mean	ALS	6	6	0.18764864	0.03018209	5.19E-02	0.31123413	ns	1.12E-07	7.38E-01	1.67E-02
20	Arm angle asym._mean	NALS	6	6	0.10315649	0.02356748	5.19E-02	0.31123413	ns	1.12E-07	7.38E-01	1.67E-02
20	Arm angle asym._mean	ALS	8	6	0.13745926	0.0353709	5.47E-02	3.28E-01	ns	1.12E-07	7.38E-01	1.67E-02
20	Arm angle asym._mean	NALS	8	6	0.27779548	0.05396679	5.47E-02	3.28E-01	ns	1.12E-07	7.38E-01	1.67E-02
20	Arm angle asym._mean	ALS	10	6	0.16637118	0.01938717	5.20E-01	1.00E+00	ns	1.12E-07	7.38E-01	1.67E-02
20	Arm angle asym._mean	NALS	10	6	0.19106088	0.03152378	5.20E-01	1.00E+00	ns	1.12E-07	7.38E-01	1.67E-02

Supplementary Table 6. Statistical summary of the feature values for the top 20 features identified by the XGB model, comparing the NALS and ALS groups. For each feature, the values at each week were divided by the group's corresponding baseline (10 wk, non-symptomatic age) mean value, and the normalised data were analysed over the experimental duration (10–20 wk [age]). Each group consisted of 6 sampling sets, each containing 150 randomly selected clips. Two-way ANOVA (group × week) was performed for each feature on the normalised data, followed by weekly t-tests comparing the NALS and ALS groups. P-values from weekly t-tests were corrected for multiple comparisons using the Bonferroni method. Source data are provided in the Source Data file (See S_Figure 21).

Rank	Feature name	Week	group	n	Mean	SEM	raw_p	corrected_p	significant	ANOVA_p_Week	ANOVA_p_Group	ANOVA_p_Interaction
1	Hands 3D distance_mean	0	NALS	376	1	0.03244642	1	1	ns	1	1	0.968137519
1	Hands 3D distance_mean	2	NALS	357	0.83680607	0.02335603	0.65439048	1	ns	1	1	0.968137519
1	Hands 3D distance_mean	4	NALS	380	0.77498829	0.01780398	0.00436138	0.02616829	*	1	1	0.968137519
1	Hands 3D distance_mean	6	NALS	339	0.81967807	0.02229885	1.63E-01	9.78E-01	ns	1	1	0.968137519
1	Hands 3D distance_mean	8	NALS	368	0.82389444	0.02166143	9.07E-02	5.44E-01	ns	1	1	0.968137519
1	Hands 3D distance_mean	10	NALS	362	0.8508868	0.01970933	1.32E-01	7.94E-01	ns	1	1	0.968137519
1	Hands 3D distance_mean	0	ALS	273	1	0.04294197	1	1	ns	1	1	0.968137519
1	Hands 3D distance_mean	2	ALS	347	0.82275724	0.02085528	0.65439048	1	ns	1	1	0.968137519
1	Hands 3D distance_mean	4	ALS	235	0.87091932	0.0314506	0.00436138	0.02616829	*	1	1	0.968137519
1	Hands 3D distance_mean	6	ALS	312	0.86709779	0.02579818	1.63E-01	9.78E-01	ns	1	1	0.968137519
1	Hands 3D distance_mean	8	ALS	196	0.89195996	0.03709987	9.07E-02	5.44E-01	ns	1	1	0.968137519
1	Hands 3D distance_mean	10	ALS	277	0.89635502	0.02295216	1.32E-01	7.94E-01	ns	1	1	0.968137519
2	Motion duration_episode	0	NALS	380	1	0.03965498	1	1	ns	1	1	0.727162473
2	Motion duration_episode	2	NALS	361	0.96253592	0.03621998	0.53473706	1	ns	1	1	0.727162473
2	Motion duration_episode	4	NALS	385	0.86921368	0.02288005	2.37E-02	1.42E-01	ns	1	1	0.727162473
2	Motion duration_episode	6	NALS	343	1.12059009	0.05022001	7.50E-01	1.00E+00	ns	1	1	0.727162473
2	Motion duration_episode	8	NALS	372	1.78080841	0.07150477	1.99E-16	1.19E-15	****	1	1	0.727162473
2	Motion duration_episode	10	NALS	366	1.16605373	0.04700521	2.47E-03	1.48E-02	*	1	1	0.727162473
2	Motion duration_episode	0	ALS	276	1	0.03733744	1	1	ns	1	1	0.727162473
2	Motion duration_episode	2	ALS	352	0.93307801	0.0304498	0.53473706	1	ns	1	1	0.727162473
2	Motion duration_episode	4	ALS	238	0.79188235	0.02261545	2.37E-02	1.42E-01	ns	1	1	0.727162473
2	Motion duration_episode	6	ALS	316	1.1411068	0.03920449	7.50E-01	1.00E+00	ns	1	1	0.727162473
2	Motion duration_episode	8	ALS	198	0.90223989	0.04603175	1.99E-16	1.19E-15	****	1	1	0.727162473
2	Motion duration_episode	10	ALS	280	0.96794945	0.04217254	2.47E-03	1.48E-02	*	1	1	0.727162473
3	Body 2D length_min	0	NALS	376	1	0.02258652	1	1	ns	1	1	0.276711492
3	Body 2D length_min	2	NALS	357	0.97268405	0.02012385	2.03E-05	1.22E-04	***	1	1	0.276711492
3	Body 2D length_min	4	NALS	380	1.01232621	0.01811582	8.65E-05	5.19E-04	***	1	1	0.276711492
3	Body 2D length_min	6	NALS	339	0.98139319	0.02010888	2.64E-08	1.59E-07	****	1	1	0.276711492
3	Body 2D length_min	8	NALS	368	1.00578691	0.01789499	2.07E-01	1.00E+00	ns	1	1	0.276711492
3	Body 2D length_min	10	NALS	362	1.00015527	0.0226384	1.14E-08	6.83E-08	****	1	1	0.276711492
3	Body 2D length_min	0	ALS	273	1	0.02817325	1	1	ns	1	1	0.276711492
3	Body 2D length_min	2	ALS	347	0.85665768	0.01799253	2.03E-05	1.22E-04	***	1	1	0.276711492
3	Body 2D length_min	4	ALS	235	0.89734101	0.02259957	8.65E-05	5.19E-04	***	1	1	0.276711492
3	Body 2D length_min	6	ALS	312	0.83094541	0.01725386	2.64E-08	1.59E-07	****	1	1	0.276711492
3	Body 2D length_min	8	ALS	196	1.04378928	0.02385434	2.07E-01	1.00E+00	ns	1	1	0.276711492
3	Body 2D length_min	10	ALS	277	1.18069167	0.01993549	1.14E-08	6.83E-08	****	1	1	0.276711492
4	Tail angle_mean	0	NALS	376	1	0.0109366	1	1	ns	1	1	0.692804224
4	Tail angle_mean	2	NALS	304	1.09435775	0.00918885	4.28E-02	2.57E-01	ns	1	1	0.692804224
4	Tail angle_mean	4	NALS	307	1.07865344	0.00852557	9.05E-01	1.00E+00	ns	1	1	0.692804224
4	Tail angle_mean	6	NALS	307	1.07357361	0.00984775	2.24E-01	1.00E+00	ns	1	1	0.692804224
4	Tail angle_mean	8	NALS	302	1.12516718	0.00743296	3.89E-02	2.33E-01	ns	1	1	0.692804224
4	Tail angle_mean	10	NALS	334	1.07759346	0.00802525	2.14E-01	1.00E+00	ns	1	1	0.692804224
4	Tail angle_mean	0	ALS	273	1	0.0142652	1	1	ns	1	1	0.692804224
4	Tail angle_mean	2	ALS	293	1.11991162	0.00857101	4.28E-02	2.57E-01	ns	1	1	0.692804224
4	Tail angle_mean	4	ALS	196	1.08038329	0.0123048	9.05E-01	1.00E+00	ns	1	1	0.692804224
4	Tail angle_mean	6	ALS	266	1.08923781	0.00788964	2.24E-01	1.00E+00	ns	1	1	0.692804224
4	Tail angle_mean	8	ALS	145	1.09560953	0.0135814	3.89E-02	2.33E-01	ns	1	1	0.692804224
4	Tail angle_mean	10	ALS	229	1.09697981	0.01473739	2.14E-01	1.00E+00	ns	1	1	0.692804224
5	Arm angle asym._std	0	NALS	376	1	0.03583617	1	1	ns	1	1	0.640671948
5	Arm angle asym._std	2	NALS	304	0.86535339	0.03826868	9.60E-01	1.00E+00	ns	1	1	0.640671948
5	Arm angle asym._std	4	NALS	307	0.76991516	0.03031707	4.22E-01	1.00E+00	ns	1	1	0.640671948
5	Arm angle asym._std	6	NALS	307	0.86744395	0.03588383	3.13E-01	1.00E+00	ns	1	1	0.640671948
5	Arm angle asym._std	8	NALS	302	0.74627313	0.03546427	1.62E-01	9.72E-01	ns	1	1	0.640671948
5	Arm angle asym._std	10	NALS	334	0.76774354	0.03220352	4.94E-02	2.97E-01	ns	1	1	0.640671948
5	Arm angle asym._std	0	ALS	273	1	0.05030177	1	1	ns	1	1	0.640671948
5	Arm angle asym._std	2	ALS	293	0.86270835	0.03590763	9.60E-01	1.00E+00	ns	1	1	0.640671948
5	Arm angle asym._std	4	ALS	196	0.81104219	0.04303269	4.22E-01	1.00E+00	ns	1	1	0.640671948
5	Arm angle asym._std	6	ALS	266	0.92168109	0.04015202	3.13E-01	1.00E+00	ns	1	1	0.640671948
5	Arm angle asym._std	8	ALS	145	0.83496942	0.053821	1.62E-01	9.72E-01	ns	1	1	0.640671948
5	Arm angle asym._std	10	ALS	229	0.66682907	0.04030837	4.94E-02	2.97E-01	ns	1	1	0.640671948
6	Motion interval_episode	0	NALS	370	1	0.07751083	1	1	ns	1	0.016810024	0.883070872
6	Motion interval_episode	2	NALS	347	1.10731603	0.08191388	4.09E-12	2.46E-11	****	1	0.016810024	0.883070872
6	Motion interval_episode	4	NALS	372	1.10504824	0.06181661	2.51E-01	1.00E+00	ns	1	0.016810024	0.883070872
6	Motion interval_episode	6	NALS	335	1.36149217	0.11241894	1.39E-06	8.35E-06	****	1	0.016810024	0.883070872
6	Motion interval_episode	8	NALS	370	1.5689031	0.20997573	5.16E-01	1.00E+00	ns	1	0.016810024	0.883070872
6	Motion interval_episode	10	NALS	359	1.12550314	0.09137512	5.89E-04	3.53E-03	**	1	0.016810024	0.883070872
6	Motion interval_episode	0	ALS	274	1	0.08221136	1	1	ns	1	0.016810024	0.883070872
6	Motion interval_episode	2	ALS	346	0.47406099	0.03636518	4.09E-12	2.46E-11	****	1	0.016810024	0.883070872
6	Motion interval_episode	4	ALS	232	0.98582055	0.08622122	2.51E-01	1.00E+00	ns	1	0.016810024	0.883070872
6	Motion interval_episode	6	ALS	308	0.71146282	0.06638936	1.39E-06	8.35E-06	****	1	0.016810024	0.883070872
6	Motion interval_episode	8	ALS	196	1.36242193	0.18236752	5.16E-01	1.00E+00	ns	1	0.016810024	0.883070872
6	Motion interval_episode	10	ALS	273	0.71813797	0.06201361	5.89E-04	3.53E-03	**	1	0.016810024	0.883070872
7	Body angle_min	0	NALS	376	1	0.04596831	1	1	ns	1	1	0.304225285
7	Body angle_min	2	NALS	304	0.79508354	0.03619024	3.08E-01	1	ns	1	1	0.304225285
7	Body angle_min	4	NALS	307	0.81305217	0.03482307	0.9594559	1	ns	1	1	0.304225285
7	Body angle_min	6	NALS	307	0.81299036	0.03817422	1.01E-01	6.08E-01	ns	1	1	0.304225285
7	Body angle_min	8	NALS	302	0.71577534	0.03378867	1.96E-01	1.00E+00	ns	1	1	0.304225285
7	Body angle_min	10	NALS	334	0.80505419	0.03500197	7.4957E-06	4.4974E-05	****	1	1	0.304225285
7	Body angle_min	0	ALS	273	1	0.05400547	1	1	ns	1	1	0.304225285
7	Body angle_min	2	ALS	293	0.74307857	0.03581768	3.08E-01	1	ns	1	1	0.304225285
7	Body angle_min	4	ALS	196	0.81028221	0.04086952	0.9594559	1	ns	1	1	0.304225285
7	Body angle_min	6	ALS	266	0.72754046	0.03444626	1.01E-01	6.08E-01	ns	1	1	0.304225285
7	Body angle_min	8	ALS	145	0.64179088	0.0427938	1.96E-01	1.00E+00	ns	1	1	0.304225285
7	Body angle_min	10	ALS	229	0.57309335	0.03509963	7.4957E-06	4.4974E-05	****	1	1	0.304225285
8	Feet 2D distance_max	0	NALS	376	1	0.01657013	1	1	ns	1	1	0.516958231
8	Feet 2D distance_max	2	NALS	358	0.98036509	0.00707125	1.40E-01	8.43E-01	ns	1	1	0.516958231
8	Feet 2D distance_max	4	NALS	380	0.97145055	0.00793301	6.65E-01	1.00E+00	ns	1	1	0.516958231
8	Feet 2D distance_max	6	NALS	339	0.9875502	0.00701031	3.51E-05	2.10E-04	***	1	1	0.516958231
8	Feet 2D distance_max	8	NALS	368	1.01592528	0.00735481	1.94E-35	1.17E-34	****	1	1	0.516958231
8	Feet 2D distance_max	10	NALS	362	1.04690109	0.00993356	7.78E-48	4.67E-47	****	1	1	0.516958231
8	Feet 2D distance_max	0	ALS	273	1	0.02588441	1	1	ns	1	1	0.516958231
8	Feet 2D distance_max	2	ALS	347	0.9546637	0.01611396	1.40E-01	8.43E-01	ns	1	1	0.516958231
8	Feet 2D distance_max	4	ALS	235	0.96579816	0.0105285	6.65E-01	1.00E+00	ns	1	1	0.516958231
8	Feet 2D distance_max	6	ALS	312	0.94955939	0.00568295	3.51E-05	2.10E-04	***	1	1	0.516958231
8	Feet 2D distance_max	8	ALS	196	0.86166204	0.00778082	1.94E-35	1.17E-34	****	1	1	0.516958231
8	Feet 2D distance_max	10	ALS	277	0.84152512	0.00717214	7.78E-48	4.67E-47	****	1	1	0.516958231
9	Neck 3D velocity_std	0	NALS	376	1	0.0278789	1	1	ns	1	1	0.107653309
9	Neck 3D velocity_std	2	NALS	304	0.98031819	0.02743405	3.75E-03	0.02250378	*	1	1	0.107653309
9	Neck 3D velocity_std	4	NALS	307	0.82747987	0.01961831	2.73E-02	1.64E-01	ns	1	1	0.107653309
9	Neck 3D velocity_std	6	NALS	307	0.98887865	0.02191371	3.16E-04	1.89E-03	**	1	1	0.107653309
9	Neck 3D velocity_std	8	NALS	302	1.00558224	0.02265851	2.62E-12	1.57E-11	****	1	1	0.107653309
9	Neck 3D velocity_std	10	NALS	334	0.85795612	0.0195485	2.20E-15	1.32E-14	****	1	1	0.107653309
9	Neck 3D velocity_std	0	ALS	273	1	0.03254331	1	1	ns	1	1	0.107653309
9	Neck 3D velocity_std	2	ALS	293	0.87661505	0.02252859	3.75E-03	0.02250378	*	1	1	0.107653309
9	Neck 3D velocity_std	4	ALS	196	0.7569271	0.02543006	2.73E-02	1.64E-01	ns	1	1	0.107653309
9	Neck 3D velocity_std	6	ALS	266	0.87139089	0.02394019	3.16E-04	1.89E-03	**	1	1	0.107653309
9	Neck 3D velocity_std	8	ALS	145	0.72835876	0.02936129	2.62E-12	1.57E-11	****	1	1	0.107653309
9	Neck 3D velocity_std	10	ALS	229	0.6064744	0.02391752	2.20E-15	1.32E-14	****	1	1	0.107653309
10	Feet 2D distance_std	0	NALS	376	1	0.03356456	1	1	ns	1	1	0.811100356
10	Feet 2D distance_std	2	NALS	357	0.92799108	0.0240249	1.14E-05	6.85E-05	****	1	1	0.811100356
10	Feet 2D distance_std	4	NALS	380	0.90313332	0.02483672	2.16E-01	1.00E+00	ns	1	1	0.811100356
10	Feet 2D distance_std	6	NALS	339	0.95895581	0.0233896	1.14E-01	6.84E-01	ns	1	1	0.811100356
10	Feet 2D distance_std	8	NALS	368	1.02144198	0.02553059	1.60E-07	9.59E-07	****	1	1	0.811100356
10	Feet 2D distance_std	10	NALS	362	1.05243534	0.03074214	1.58E-21	9.49E-21	****	1	1	0.811100356
10	Feet 2D distance_std	0	ALS	273	1	0.05668319	1	1	ns	1	1	0.811100356
10	Feet 2D distance_std	2	ALS	347	1.15120732	0.04486392	1.14E-05	6.85E-05	****	1	1	0.811100356
10	Feet 2D distance_std	4	ALS	235	0.95455645	0.0342047	2.16E-01	1.00E+00	ns	1	1	0.811100356
10	Feet 2D distance_std	6	ALS	312	1.0165472	0.02816934	1.14E-01	6.84E-01	ns	1	1	0.811100356
10	Feet 2D distance_std	8	ALS	196	0.79733981	0.03238438	1.60E-07	9.59E-07	****	1	1	0.811100356
10	Feet 2D distance_std	10	ALS	277	0.63849569	0.02603855	1.58E-21	9.49E-21	****	1	1	0.811100356
11	Chest 3D velocity_mean	0	NALS	376	1	0.02156116	1	1	ns	1	1	0.620057799
11	Chest 3D velocity_mean	2	NALS	304	0.86298819	0.01509727	7.03E-01	1.00E+00	ns	1	1	0.620057799
11	Chest 3D velocity_mean	4	NALS	307	0.85507111	0.01421542	1.42E-02	8.51E-02	ns	1	1	0.620057799
11	Chest 3D velocity_mean	6	NALS	307	0.90054065	0.01518301	9.21E-01	1.00E+00	ns	1	1	0.620057799
11	Chest 3D velocity_mean	8	NALS	302	0.92496029	0.01572826	1.49E-06	8.94E-06	****	1	1	0.620057799
11	Chest 3D velocity_mean	10	NALS	334	0.88330952	0.01398322	3.67E-29	2.20E-28	****	1	1	0.620057799
11	Chest 3D velocity_mean	0	ALS	273	1	0.02915419	1	1	ns	1	1	0.620057799
11	Chest 3D velocity_mean	2	ALS	293	0.85456281	0.01613098	7.03E-01	1.00E+00	ns	1	1	0.620057799
11	Chest 3D velocity_mean	4	ALS	196	0.80111808	0.01602561	1.42E-02	8.51E-02	ns	1	1	0.620057799
11	Chest 3D velocity_mean	6	ALS	266	0.90287087	0.01825411	9.21E-01	1.00E+00	ns	1	1	0.620057799
11	Chest 3D velocity_mean	8	ALS	145	0.79891128	0.01779593	1.49E-06	8.94E-06	****	1	1	0.620057799
11	Chest 3D velocity_mean	10	ALS	229	0.65620152	0.0108422	3.67E-29	2.20E-28	****	1	1	0.620057799
12	U-body 3D length_min	0	NALS	376	1	0.02432119	1	1	ns	1	1	0.942228388
12	U-body 3D length_min	2	NALS	357	0.97598784	0.01935181	0.60294466	1	ns	1	1	0.942228388
12	U-body 3D length_min	4	NALS	380	0.9930572	0.01918147	0.37624872	1	ns	1	1	0.942228388
12	U-body 3D length_min	6	NALS	339	0.9680305	0.02083948	2.88E-01	1	ns	1	1	0.942228388
12	U-body 3D length_min	8	NALS	368	0.95079404	0.0209082	0.83490091	1	ns	1	1	0.942228388
12	U-body 3D length_min	10	NALS	362	0.94442628	0.02252607	8.02E-03	4.81E-02	*	1	1	0.942228388
12	U-body 3D length_min	0	ALS	273	1	0.02989751	1	1	ns	1	1	0.942228388
12	U-body 3D length_min	2	ALS	347	0.98925294	0.01649443	0.60294466	1	ns	1	1	0.942228388
12	U-body 3D length_min	4	ALS	235	0.96668502	0.0217299	0.37624872	1	ns	1	1	0.942228388
12	U-body 3D length_min	6	ALS	312	0.93877102	0.01759829	2.88E-01	1	ns	1	1	0.942228388
12	U-body 3D length_min	8	ALS	196	0.95756265	0.02089785	0.83490091	1	ns	1	1	0.942228388
12	U-body 3D length_min	10	ALS	277	1.02513554	0.01834954	8.02E-03	4.81E-02	*	1	1	0.942228388
13	Right limb angle asym._min	0	NALS	376	1	0.04110313	1	1	ns	1	1	0.80659571
13	Right limb angle asym._min	2	NALS	304	1.09850295	0.04049394	1.71E-05	1.02E-04	***	1	1	0.80659571
13	Right limb angle asym._min	4	NALS	307	1.00080038	0.03934924	0.00012471	0.00074826	***	1	1	0.80659571
13	Right limb angle asym._min	6	NALS	307	1.04028227	0.03878293	4.51E-03	2.70E-02	*	1	1	0.80659571
13	Right limb angle asym._min	8	NALS	302	1.0586897	0.03544283	7.16E-05	4.30E-04	***	1	1	0.80659571
13	Right limb angle asym._min	10	NALS	334	1.02694719	0.03690332	7.91E-06	4.75E-05	****	1	1	0.80659571
13	Right limb angle asym._min	0	ALS	273	1	0.04686535	1	1	ns	1	1	0.80659571
13	Right limb angle asym._min	2	ALS	293	0.85238325	0.03972518	1.71E-05	1.02E-04	***	1	1	0.80659571
13	Right limb angle asym._min	4	ALS	196	0.76195857	0.04665055	0.00012471	0.00074826	***	1	1	0.80659571
13	Right limb angle asym._min	6	ALS	266	0.88358906	0.0384852	4.51E-03	2.70E-02	*	1	1	0.80659571
13	Right limb angle asym._min	8	ALS	145	0.8147155	0.04759504	7.16E-05	4.30E-04	***	1	1	0.80659571
13	Right limb angle asym._min	10	ALS	229	0.78298556	0.03702678	7.91E-06	4.75E-05	****	1	1	0.80659571
14	Hands 3D distance_max	0	NALS	376	1	0.0335389	1	1	ns	1	1	0.609716905
14	Hands 3D distance_max	2	NALS	357	0.9024908	0.02903106	6.67E-01	1.00E+00	ns	1	1	0.609716905
14	Hands 3D distance_max	4	NALS	380	0.86823346	0.02665607	7.86E-01	1.00E+00	ns	1	1	0.609716905
14	Hands 3D distance_max	6	NALS	339	0.90443754	0.02888204	6.36E-01	1.00E+00	ns	1	1	0.609716905
14	Hands 3D distance_max	8	NALS	368	0.88684209	0.0290095	3.68E-01	1.00E+00	ns	1	1	0.609716905
14	Hands 3D distance_max	10	NALS	362	0.92213668	0.02888026	3.02E-06	1.81E-05	****	1	1	0.609716905
14	Hands 3D distance_max	0	ALS	273	1	0.03987492	1	1	ns	1	1	0.609716905
14	Hands 3D distance_max	2	ALS	347	0.92077645	0.03100609	6.67E-01	1.00E+00	ns	1	1	0.609716905
14	Hands 3D distance_max	4	ALS	235	0.85620889	0.03615509	7.86E-01	1.00E+00	ns	1	1	0.609716905
14	Hands 3D distance_max	6	ALS	312	0.92509809	0.0329777	6.36E-01	1.00E+00	ns	1	1	0.609716905
14	Hands 3D distance_max	8	ALS	196	0.84181903	0.04150033	3.68E-01	1.00E+00	ns	1	1	0.609716905
14	Hands 3D distance_max	10	ALS	277	0.7290871	0.02774198	3.02E-06	1.81E-05	****	1	1	0.609716905
15	Left limb angle asym._min	0	NALS	376	1	0.0467893	1	1	ns	1.00E+00	1	0.743280976
15	Left limb angle asym._min	2	NALS	304	1.06999864	0.04338117	5.74E-07	3.44E-06	****	1.00E+00	1	0.743280976
15	Left limb angle asym._min	4	NALS	307	0.94778073	0.03882191	3.85E-05	2.31E-04	***	1.00E+00	1	0.743280976
15	Left limb angle asym._min	6	NALS	307	1.00856294	0.03895553	1.51E-04	9.08E-04	***	1.00E+00	1	0.743280976
15	Left limb angle asym._min	8	NALS	302	1.07605138	0.03977225	1.89E-04	1.13E-03	**	1.00E+00	1	0.743280976
15	Left limb angle asym._min	10	NALS	334	0.98187107	0.03953998	4.39E-03	2.64E-02	*	1.00E+00	1	0.743280976
15	Left limb angle asym._min	0	ALS	273	1	0.04589296	1	1	ns	1.00E+00	1	0.743280976
15	Left limb angle asym._min	2	ALS	293	0.77185119	0.03979295	5.74E-07	3.44E-06	****	1.00E+00	1	0.743280976
15	Left limb angle asym._min	4	ALS	196	0.69626255	0.04523548	3.85E-05	2.31E-04	***	1.00E+00	1	0.743280976
15	Left limb angle asym._min	6	ALS	266	0.803321	0.03632565	1.51E-04	9.08E-04	***	1.00E+00	1	0.743280976
15	Left limb angle asym._min	8	ALS	145	0.82398955	0.04969788	1.89E-04	1.13E-03	**	1.00E+00	1	0.743280976
15	Left limb angle asym._min	10	ALS	229	0.81431364	0.04098797	4.39E-03	2.64E-02	*	1.00E+00	1	0.743280976
16	Chest 1D velocity_max	0	NALS	379	1	0.13463781	1	1	ns	1.00E+00	1	0.983022387
16	Chest 1D velocity_max	2	NALS	361	0.49198807	0.02526344	1.48E-01	8.87E-01	ns	1.00E+00	1	0.983022387
16	Chest 1D velocity_max	4	NALS	384	0.49331164	0.02152096	1.12E-04	6.73E-04	***	1.00E+00	1	0.983022387
16	Chest 1D velocity_max	6	NALS	343	0.55845347	0.02828607	2.61E-02	1.57E-01	ns	1.00E+00	1	0.983022387
16	Chest 1D velocity_max	8	NALS	372	0.48715029	0.02632421	1.72E-08	1.03E-07	****	1.00E+00	1	0.983022387
16	Chest 1D velocity_max	10	NALS	366	0.48723923	0.02551524	1.72E-20	1.03E-19	****	1.00E+00	1	0.983022387
16	Chest 1D velocity_max	0	ALS	276	1	0.17665812	1	1	ns	1.00E+00	1	0.983022387
16	Chest 1D velocity_max	2	ALS	351	0.44513878	0.02001336	1.48E-01	8.87E-01	ns	1.00E+00	1	0.983022387
16	Chest 1D velocity_max	4	ALS	238	0.36632892	0.02270576	1.12E-04	6.73E-04	***	1.00E+00	1	0.983022387
16	Chest 1D velocity_max	6	ALS	316	0.47752768	0.02208554	2.61E-02	1.57E-01	ns	1.00E+00	1	0.983022387
16	Chest 1D velocity_max	8	ALS	198	0.2651377	0.01954777	1.72E-08	1.03E-07	****	1.00E+00	1	0.983022387
16	Chest 1D velocity_max	10	ALS	280	0.18914288	0.01213636	1.72E-20	1.03E-19	****	1.00E+00	1	0.983022387
17	Leg angle asym._std	0	NALS	376	1	0.03027278	1	1	ns	1	0.741834603	0.816010316
17	Leg angle asym._std	2	NALS	304	0.81165023	0.02808353	8.51E-01	1.00E+00	ns	1	0.741834603	0.816010316
17	Leg angle asym._std	4	NALS	307	0.88268709	0.03102684	4.71E-01	1.00E+00	ns	1	0.741834603	0.816010316
17	Leg angle asym._std	6	NALS	307	0.85416432	0.03016555	3.79E-01	1.00E+00	ns	1	0.741834603	0.816010316
17	Leg angle asym._std	8	NALS	302	0.84502092	0.02903317	7.07E-01	1.00E+00	ns	1	0.741834603	0.816010316
17	Leg angle asym._std	10	NALS	334	0.85744061	0.02806219	2.29E-01	1.00E+00	ns	1	0.741834603	0.816010316
17	Leg angle asym._std	0	ALS	273	1	0.04080125	1	1	ns	1	0.741834603	0.816010316
17	Leg angle asym._std	2	ALS	293	0.82030349	0.0367284	8.51E-01	1.00E+00	ns	1	0.741834603	0.816010316
17	Leg angle asym._std	4	ALS	196	0.84638498	0.03999281	4.71E-01	1.00E+00	ns	1	0.741834603	0.816010316
17	Leg angle asym._std	6	ALS	266	0.81384413	0.03469206	3.79E-01	1.00E+00	ns	1	0.741834603	0.816010316
17	Leg angle asym._std	8	ALS	145	0.86565944	0.05116832	7.07E-01	1.00E+00	ns	1	0.741834603	0.816010316
17	Leg angle asym._std	10	ALS	229	0.92009914	0.04766661	2.29E-01	1.00E+00	ns	1	0.741834603	0.816010316
18	Feet 2D distance_mean	0	NALS	376	1	0.00987995	1	1	ns	1	0.661460416	0.279372361
18	Feet 2D distance_mean	2	NALS	357	1.03287701	0.00794345	2.88E-19	1.73E-18	****	1	0.661460416	0.279372361
18	Feet 2D distance_mean	4	NALS	380	1.01354025	0.00921706	9.83E-01	1.00E+00	ns	1	0.661460416	0.279372361
18	Feet 2D distance_mean	6	NALS	339	1.03340482	0.00940107	0.02157304	0.12943824	ns	1	0.661460416	0.279372361
18	Feet 2D distance_mean	8	NALS	368	1.06199121	0.00914941	4.91E-13	2.94E-12	****	1	0.661460416	0.279372361
18	Feet 2D distance_mean	10	NALS	362	1.08608257	0.0103536	3.5717E-13	2.143E-12	****	1	0.661460416	0.279372361
18	Feet 2D distance_mean	0	ALS	273	1	0.01290542	1	1	ns	1	0.661460416	0.279372361
18	Feet 2D distance_mean	2	ALS	347	0.91411712	0.01015662	2.88E-19	1.73E-18	****	1	0.661460416	0.279372361
18	Feet 2D distance_mean	4	ALS	235	1.01323652	0.01052175	9.83E-01	1.00E+00	ns	1	0.661460416	0.279372361
18	Feet 2D distance_mean	6	ALS	312	1.00330974	0.00900799	0.02157304	0.12943824	ns	1	0.661460416	0.279372361
18	Feet 2D distance_mean	8	ALS	196	0.95555106	0.00964269	4.91E-13	2.94E-12	****	1	0.661460416	0.279372361
18	Feet 2D distance_mean	10	ALS	277	0.98156292	0.00869956	3.5717E-13	2.143E-12	****	1	0.661460416	0.279372361
19	Anus 1D velocity_std	0	NALS	376	1	0.02977514	1	1	ns	1	1	0.573825508
19	Anus 1D velocity_std	2	NALS	357	0.89929337	0.02294443	0.31869271	1	ns	1	1	0.573825508
19	Anus 1D velocity_std	4	NALS	380	0.84548516	0.02191241	0.68785341	1	ns	1	1	0.573825508
19	Anus 1D velocity_std	6	NALS	339	0.96307074	0.02625684	5.30E-03	3.18E-02	*	1	1	0.573825508
19	Anus 1D velocity_std	8	NALS	368	0.94560663	0.02368847	2.93E-11	1.76E-10	****	1	1	0.573825508
19	Anus 1D velocity_std	10	NALS	362	0.87273014	0.02228468	2.82E-30	1.69E-29	****	1	1	0.573825508
19	Anus 1D velocity_std	0	ALS	273	1	0.0372979	1	1	ns	1	1	0.573825508
19	Anus 1D velocity_std	2	ALS	347	0.93138166	0.02250909	0.31869271	1	ns	1	1	0.573825508
19	Anus 1D velocity_std	4	ALS	235	0.85980258	0.02821474	0.68785341	1	ns	1	1	0.573825508
19	Anus 1D velocity_std	6	ALS	312	0.86778683	0.02112923	5.30E-03	3.18E-02	*	1	1	0.573825508
19	Anus 1D velocity_std	8	ALS	196	0.69653109	0.02346075	2.93E-11	1.76E-10	****	1	1	0.573825508
19	Anus 1D velocity_std	10	ALS	277	0.50772062	0.01872052	2.82E-30	1.69E-29	****	1	1	0.573825508
20	Arm angle asym._mean	0	NALS	376	1	0.2247155	1	1	ns	1	1	0.972565258
20	Arm angle asym._mean	2	NALS	304	1.17876338	0.20383996	0.41365264	1	ns	1	1	0.972565258
20	Arm angle asym._mean	4	NALS	307	1.02470223	0.18912839	2.58E-01	1	ns	1	1	0.972565258
20	Arm angle asym._mean	6	NALS	307	0.86252527	0.20453886	9.70E-01	1.00E+00	ns	1	1	0.972565258
20	Arm angle asym._mean	8	NALS	302	1.35578143	0.16301428	0.01885519	0.11313112	ns	1	1	0.972565258
20	Arm angle asym._mean	10	NALS	334	0.6727184	0.16615215	1.08E-02	6.48E-02	ns	1	1	0.972565258
20	Arm angle asym._mean	0	ALS	273	1	0.18546912	1	1	ns	1	1	0.972565258
20	Arm angle asym._mean	2	ALS	293	0.98153099	0.12482021	0.41365264	1	ns	1	1	0.972565258
20	Arm angle asym._mean	4	ALS	196	0.71971952	0.16065561	2.58E-01	1	ns	1	1	0.972565258
20	Arm angle asym._mean	6	ALS	266	0.87187354	0.12511014	9.70E-01	1.00E+00	ns	1	1	0.972565258
20	Arm angle asym._mean	8	ALS	145	0.72510885	0.1838217	0.01885519	0.11313112	ns	1	1	0.972565258
20	Arm angle asym._mean	10	ALS	229	1.26599659	0.14043934	1.08E-02	6.48E-02	ns	1	1	0.972565258

Supplementary Table 7. Statistical comparisons of the top 20 features identified by the XGB model comparing PD (A5) and ALS groups. For each feature, values from PD (at 10 wk post-surgery) and ALS (at 20 wk of age) groups were normalised relative to their matched control group means (CT for PD, WT for ALS). Statistical significance was assessed using Welch's t-test (unequal variances) comparing each disease group to their respective control group. Source data are provided in Supplementary Tables 4 and 6.

Rank	Parameter	Group	n	Mean_diff	SEM_diff	corrected_p	Significance
1	Hands 3D distance_mean	PD(A5)	2756	0.162686334	0.014584544	1.97E-14	****
1	Hands 3D distance_mean	ALS	277	0.045468225	0.03025326	0.794408044	ns
2	Motion duration_episode	PD(A5)	2826	0.787204636	0.041538671	4.28E-33	****
2	Motion duration_episode	ALS	280	-0.198104276	0.063150715	1.48E-02	*
3	Body 2D length_min	PD(A5)	2797	0.207146827	0.009967946	1.41E-95	****
3	Body 2D length_min	ALS	277	0.180536399	0.030164893	6.83E-08	****
4	Tail angle_mean	PD(A5)	2784	0.079448551	0.004191403	6.81E-102	****
4	Tail angle_mean	ALS	229	0.01938635	0.016780802	1	ns
5	Arm angle asym._std	PD(A5)	2771	0.404388738	0.01897521	5.66E-59	****
5	Arm angle asym._std	ALS	229	-0.100914471	0.051592941	0.296609597	ns
6	Motion interval_episode	PD(A5)	2790	-1.388804715	0.079958603	8.24E-124	****
6	Motion interval_episode	ALS	273	-0.407365168	0.110431428	0.003531384	**
7	Body angle_min	PD(A5)	2796	0.081157082	0.025905914	0.005882938	**
7	Body angle_min	ALS	229	-0.231960833	0.04956936	4.49743E-05	****
8	Feet 2D distance_max	PD(A5)	2786	0.09003698	0.005120403	1.97E-45	****
8	Feet 2D distance_max	ALS	277	-0.205375963	0.01225215	4.67E-47	****
9	Neck 3D velocity_std	PD(A5)	2779	-0.068560416	0.008704187	2.82575E-14	****
9	Neck 3D velocity_std	ALS	229	-0.251481717	0.030889996	1.32E-14	****
10	Feet 2D distance_std	PD(A5)	2784	0.44093211	0.020853577	3.51E-72	****
10	Feet 2D distance_std	ALS	277	-0.413939657	0.040287533	9.49E-21	****
11	Chest 3D velocity_mean	PD(A5)	2807	0.157380852	0.013473108	3.90E-26	****
11	Chest 3D velocity_mean	ALS	229	-0.227108	0.017694173	2.20E-28	****
12	U-body 3D length_min	PD(A5)	2786	0.03724519	0.00585367	2.29E-11	****
12	U-body 3D length_min	ALS	277	0.08070926	0.029053906	4.81E-02	*
13	Right limb angle asym._min	PD(A5)	2748	0.209480031	0.01885907	1.42E-15	****
13	Right limb angle asym._min	ALS	229	-0.243961629	0.05227655	4.74756E-05	****
14	Hands 3D distance_max	PD(A5)	2766	0.263567435	0.013461755	3.04E-43	****
14	Hands 3D distance_max	ALS	277	-0.193049579	0.040046059	1.81E-05	****
15	Left limb angle asym._min	PD(A5)	2750	0.377645194	0.019315189	4.19E-47	****
15	Left limb angle asym._min	ALS	229	-0.167557435	0.056951063	0.026367472	*
16	Chest 1D velocity_max	PD(A5)	2798	-0.437640672	0.029897602	4.33E-75	****
16	Chest 1D velocity_max	ALS	280	-0.298096347	0.028254535	1.03E-19	****
17	Leg angle asym._std	PD(A5)	2793	0.422838958	0.017048476	2.57E-94	****
17	Leg angle asym._std	ALS	229	0.062658523	0.055313579	1	ns
18	Feet 2D distance_mean	PD(A5)	2782	0.001800359	0.005788474	1	ns
18	Feet 2D distance_mean	ALS	277	-0.104519653	0.013523291	2.14302E-12	****
19	Anus 1D velocity_std	PD(A5)	2790	-0.156951379	0.019093362	1.28E-16	****
19	Anus 1D velocity_std	ALS	277	-0.365009519	0.029104378	1.69E-29	****
20	Arm angle asym._mean	PD(A5)	2746	1.694347026	0.157733005	1.05E-15	****
20	Arm angle asym._mean	ALS	229	0.593278188	0.217554004	0.06476644	ns

Supplementary Table 8. Statistical summary of the APS (XGB model-predicted PD scores) at 10 wk across groups. Statistical analysis was performed using one-way ANOVA, followed by Bonferroni's correction for multiple comparisons. Source data are provided in the Source Data file (See S_Figure 25).

Group	Number of values	Mean	Std. Deviation	Std. Error of Mean
EV	10	12.11	2.698	0.8531
A5	12	85.61	6.729	1.942
A5OD	7	68.77	17.54	6.628
A5O1	7	82	9.655	3.649
A5O2	6	75.93	17.14	6.998
A1	15	58.01	25.9	6.688
A1OD	10	62.26	20.21	6.392
A1O1	7	52.88	22.27	8.415
A1O2	12	29.06	17.11	4.94
A1O3	8	29.41	18.97	6.708

ANOVA summary	
F	18.74
P value	<0.0001
P value summary	****
Significant diff. among means (P < 0.05)?	Yes
R squared	0.6676

Bonferroni's multiple comparisons test	Mean Diff.	95.00% CI of diff.	Summary	Adjusted P Value
EV vs. A5	-73.5	-95.03 to -51.96	****	<0.0001
EV vs. A5OD	-56.66	-81.44 to -31.88	****	<0.0001
EV vs. A5O1	-69.89	-94.67 to -45.10	****	<0.0001
EV vs. A5O2	-63.82	-89.79 to -37.85	****	<0.0001
EV vs. A1	-45.9	-66.43 to -25.37	****	<0.0001
EV vs. A1OD	-50.15	-72.64 to -27.66	****	<0.0001
EV vs. A1O1	-40.77	-65.55 to -15.98	****	<0.0001
EV vs. A1O2	-16.95	-38.48 to 4.583	ns	0.2492
EV vs. A1O3	-17.3	-41.15 to 6.559	ns	0.3791
A5 vs. EV	73.5	51.96 to 95.03	****	<0.0001
A5 vs. A5OD	16.84	-7.082 to 40.76	ns	0.435
A5 vs. A5O1	3.607	-20.31 to 27.53	ns	>0.9999
A5 vs. A5O2	9.673	-15.47 to 34.82	ns	>0.9999
A5 vs. A1	27.6	8.119 to 47.07	**	0.0011
A5 vs. A1OD	23.35	1.812 to 44.88	*	0.0247
A5 vs. A1O1	32.73	8.809 to 56.65	**	0.0018
A5 vs. A1O2	56.55	36.01 to 77.08	****	<0.0001
A5 vs. A1O3	56.2	33.24 to 79.15	****	<0.0001
A1 vs. EV	45.9	25.37 to 66.43	****	<0.0001
A1 vs. A5	-27.6	-47.07 to -8.119	**	0.0011
A1 vs. A5OD	-10.76	-33.78 to 12.26	ns	>0.9999
A1 vs. A5O1	-23.99	-47.01 to -0.9689	*	0.0353
A1 vs. A5O2	-17.92	-42.22 to 6.369	ns	0.3485
A1 vs. A1OD	-4.251	-24.78 to 16.28	ns	>0.9999
A1 vs. A1O1	5.131	-17.89 to 28.15	ns	>0.9999
A1 vs. A1O2	28.95	9.470 to 48.43	***	0.0005
A1 vs. A1O3	28.6	6.585 to 50.62	**	0.0035

Supplementary Table 9. Statistical summary of the KL divergences for the top 20 features identified by the XGB model. For each group, the KL divergence of each feature compared to the CT counterpart was computed at 10 wk. Each group consists of 6 sampling sets, each containing 150 randomly selected clips. A one-way ANOVA was performed across groups, followed by pairwise comparisons using t-tests against the reference groups (CT or A1). The p-values from pairwise comparisons were corrected for multiple comparisons using the Holm–Sidak method. Source data are provided in the Source Data file (See S_Figure 29).

Rank	Parameter	Group	n	Mean	SEM	ANOVA p-value	Reference	Raw Post-hoc p-value	Corrected Post-hoc p-value	Significance	Reference	Raw Post-hoc p-value	Corrected Post-hoc p-value	Significance
1	Hands 3D distance (mean)	CT	6	0	0	1.4657E-11	CT				A1	8.5978E-05	0.00034387	***
1	Hands 3D distance (mean)	A5	6	0.24853803	0.02091022	1.4657E-11	CT	7.4259E-05	0.000297	***	A1	0.00035378	0.00106097	**
1	Hands 3D distance (mean)	A1	6	0.09672699	0.0083876	1.4657E-11	CT	8.5978E-05	0.000297	***	A1			
1	Hands 3D distance (mean)	A1O3	6	0.03812882	0.00734181	1.4657E-11	CT	0.00348621	0.00696026	**	A1	0.00039152	0.00106097	**
2	Motion duration (episode)	CT	6	0	0	1.0213E-16	CT				A1	0.00016279	0.0004883	***
2	Motion duration (episode)	A5	6	0.93621279	0.03433908	1.0213E-16	CT	1.242E-06	4.9681E-06	****	A1	2.4896E-07	9.9584E-07	****
2	Motion duration (episode)	A1	6	0.13810015	0.01367004	1.0213E-16	CT	0.00016279	0.0004883	***	A1			
2	Motion duration (episode)	A1O3	6	0.14644016	0.03310061	1.0213E-16	CT	0.00686575	0.01368435	*	A1	0.82285053	0.96861806	
3	Body 2D length (min)	CT	6	0	0	1.7628E-06	CT				A1	0.00151666	0.00605287	**
3	Body 2D length (min)	A5	6	0.1625595	0.02377397	1.7628E-06	CT	0.00102104	0.00407792	**	A1	0.03412213	0.09891317	
3	Body 2D length (min)	A1	6	0.09186109	0.01465504	1.7628E-06	CT	0.00151666	0.00454309	**	A1			
3	Body 2D length (min)	A1O3	6	0.05542202	0.00901598	1.7628E-06	CT	0.00165607	0.00454309	**	A1	0.06577993	0.12723287	
4	Tail angle (mean)	CT	6	0	0	6.2023E-09	CT				A1	0.00202415	0.00606017	**
4	Tail angle (mean)	A5	6	0.53773734	0.07829356	6.2023E-09	CT	0.00100041	0.00299822	**	A1	0.00122854	0.00490512	**
4	Tail angle (mean)	A1	6	0.02546974	0.00433337	6.2023E-09	CT	0.00202415	0.0040442	**	A1			
4	Tail angle (mean)	A1O3	6	0.04029791	0.00467342	6.2023E-09	CT	0.00034628	0.00138442	**	A1	0.04243136	0.08306231	
5	Arm angle asym. (std)	CT	6	0	0	3.9614E-12	CT				A1	0.00065626	0.00196748	**
5	Arm angle asym. (std)	A5	6	0.22156443	0.01282172	3.9614E-12	CT	1.1887E-05	4.7548E-05	****	A1	1.4311E-05	5.7244E-05	****
5	Arm angle asym. (std)	A1	6	0.08598877	0.01142796	3.9614E-12	CT	0.00065626	0.00131208	**	A1			
5	Arm angle asym. (std)	A1O3	6	0.09182841	0.00676987	3.9614E-12	CT	3.9026E-05	0.00011707	***	A1	0.67165539	0.89218982	
6	Motion interval (episode)	CT	6	0	0	4.3377E-05	CT				A1	0.00023606	0.0009439	***
6	Motion interval (episode)	A5	6	1.65484272	0.42703004	4.3377E-05	CT	0.01169811	0.02325937	*	A1	0.01358664	0.03424944	*
6	Motion interval (episode)	A1	6	0.06267018	0.00670456	4.3377E-05	CT	0.00023606	0.0009439	***	A1			
6	Motion interval (episode)	A1O3	6	0.16419783	0.02674113	4.3377E-05	CT	0.00166435	0.00498473	**	A1	0.01154936	0.03424944	*
7	Body angle (min)	CT	6	0	0	0.00088124	CT				A1	0.00080964	0.00323464	**
7	Body angle (min)	A5	6	0.04903378	0.01228417	0.00088124	CT	0.01040966	0.02071095	*	A1	0.11349299	0.30329885	
7	Body angle (min)	A1	6	0.02524431	0.00351047	0.00088124	CT	0.00080964	0.00323464	**	A1			
7	Body angle (min)	A1O3	6	0.02791496	0.00559586	0.00088124	CT	0.00414537	0.01238462	*	A1	0.69609165	0.90763971	
8	Feet 2D distance (max)	CT	6	0	0	1.8123E-09	CT				A1	0.00011377	0.000455	***
8	Feet 2D distance (max)	A5	6	0.29763162	0.02183963	1.8123E-09	CT	3.8142E-05	0.00015256	***	A1	0.67686725	0.89558523	
8	Feet 2D distance (max)	A1	6	0.28304216	0.02600732	1.8123E-09	CT	0.00011377	0.00022752	***	A1			
8	Feet 2D distance (max)	A1O3	6	0.24577454	0.02015415	1.8123E-09	CT	6.5562E-05	0.00019667	***	A1	0.28537722	0.63505235	
9	Neck 3D velocity (std)	CT	6	0	0	1.019E-06	CT				A1	3.8947E-05	0.00015578	***
9	Neck 3D velocity (std)	A5	6	0.05858997	0.00767981	1.019E-06	CT	0.00061538	0.001845	**	A1	0.00033888	0.00101629	**
9	Neck 3D velocity (std)	A1	6	0.12224279	0.00900838	1.019E-06	CT	3.8947E-05	0.00015578	***	A1			
9	Neck 3D velocity (std)	A1O3	6	0.11340837	0.02028614	1.019E-06	CT	0.00252717	0.00504796	**	A1	0.70265108	0.91158362	
10	Feet 2D distance (std)	CT	6	0	0	2.4805E-09	CT				A1	1.4963E-05	5.9849E-05	****
10	Feet 2D distance (std)	A5	6	0.42658563	0.05275707	2.4805E-09	CT	0.00046883	0.00093744	***	A1	0.00175341	0.00525101	**
10	Feet 2D distance (std)	A1	6	0.11166519	0.00677095	2.4805E-09	CT	1.4963E-05	5.9849E-05	****	A1			
10	Feet 2D distance (std)	A1O3	6	0.08581155	0.00802658	2.4805E-09	CT	0.00012398	0.0003719	***	A1	0.03419018	0.06721139	
11	Chest 3D velocity (mean)	CT	6	0	0	1.2431E-07	CT				A1	0.00606837	0.01809487	*
11	Chest 3D velocity (mean)	A5	6	0.28126673	0.04730435	1.2431E-07	CT	0.00192244	0.00766762	**	A1	0.00291013	0.01158979	*
11	Chest 3D velocity (mean)	A1	6	0.02911746	0.00638831	1.2431E-07	CT	0.00606837	0.01809487	*	A1			
11	Chest 3D velocity (mean)	A1O3	6	0.02531245	0.00558812	1.2431E-07	CT	0.00622727	0.01809487	*	A1	0.66365413	0.88687146	
12	U-body 3D length (min)	CT	6	0	0	8.9539E-05	CT				A1	0.00040001	0.00159909	**
12	U-body 3D length (min)	A5	6	0.10925164	0.02419242	8.9539E-05	CT	0.00630634	0.0125729	*	A1	0.22456437	0.39869959	
12	U-body 3D length (min)	A1	6	0.07455505	0.00891448	8.9539E-05	CT	0.00040001	0.00159909	**	A1			
12	U-body 3D length (min)	A1O3	6	0.04529496	0.00580946	8.9539E-05	CT	0.00055601	0.0016671	**	A1	0.02343544	0.06867154	
13	Right limb angle asym. (min)	CT	6	0	0	4.3876E-18	CT				A1	3.5095E-06	1.2372E-05	****
13	Right limb angle asym. (min)	A5	6	0.34962419	0.01432611	4.3876E-18	CT	2.1536E-06	8.6142E-06	****	A1	3.0931E-06	1.2372E-05	****
13	Right limb angle asym. (min)	A1	6	0.100987	0.00456612	4.3876E-18	CT	3.5095E-06	1.0528E-05	****	A1			
13	Right limb angle asym. (min)	A1O3	6	0.05092985	0.0032422	4.3876E-18	CT	1.9009E-05	3.8018E-05	****	A1	8.8969E-06	1.7794E-05	****
14	Hands 3D distance (max)	CT	6	0	0	2.6677E-09	CT				A1	0.00220934	0.0066134	**
14	Hands 3D distance (max)	A5	6	0.33935577	0.03681653	2.6677E-09	CT	0.00025233	0.00100896	**	A1	0.00113205	0.00452054	**
14	Hands 3D distance (max)	A1	6	0.12945619	0.02246339	2.6677E-09	CT	0.00220934	0.00441381	**	A1			
14	Hands 3D distance (max)	A1O3	6	0.04340557	0.00675169	2.6677E-09	CT	0.00135259	0.00405227	**	A1	0.01080096	0.02148527	*
15	Left limb angle asym. (min)	CT	6	0	0	1.2285E-10	CT				A1	0.00504408	0.01505603	*
15	Left limb angle asym. (min)	A5	6	0.37697644	0.0234627	1.2285E-10	CT	1.7012E-05	6.8048E-05	****	A1	0.00021312	0.00085221	***
15	Left limb angle asym. (min)	A1	6	0.14843977	0.03116238	1.2285E-10	CT	0.00504408	0.01006271	*	A1			
15	Left limb angle asym. (min)	A1O3	6	0.05771553	0.01041517	1.2285E-10	CT	0.00262692	0.00786007	**	A1	0.0322279	0.06341716	
16	Chest 1D velocity (max)	CT	6	0	0	1.847E-10	CT				A1	0.00183718	0.00550141	**
16	Chest 1D velocity (max)	A5	6	0.34418727	0.02662771	1.847E-10	CT	4.9379E-05	0.0001975	***	A1	0.00024995	0.00099943	***
16	Chest 1D velocity (max)	A1	6	0.1446222	0.02407751	1.847E-10	CT	0.00183718	0.00367098	**	A1			
16	Chest 1D velocity (max)	A1O3	6	0.08281255	0.0067853	1.847E-10	CT	6.5302E-05	0.0001975	***	A1	0.04985402	0.09722262	
17	Leg angle asym. (std)	CT	6	0	0	2.1019E-07	CT				A1	2.6731E-05	0.00010692	***
17	Leg angle asym. (std)	A5	6	0.23570965	0.03626673	2.1019E-07	CT	0.00128725	0.00257285	**	A1	0.00998463	0.02965581	*
17	Leg angle asym. (std)	A1	6	0.09144966	0.00624036	2.1019E-07	CT	2.6731E-05	0.00010692	***	A1			
17	Leg angle asym. (std)	A1O3	6	0.06996137	0.00527154	2.1019E-07	CT	4.3415E-05	0.00013024	***	A1	0.02568758	0.05071531	
18	Feet 2D distance (mean)	CT	6	0	0	4.6966E-09	CT				A1	0.00020305	0.00081194	***
18	Feet 2D distance (mean)	A5	6	0.24056345	0.02176779	4.6966E-09	CT	0.00010565	0.00031691	***	A1	0.46583436	0.71466707	
18	Feet 2D distance (mean)	A1	6	0.21683894	0.0224773	4.6966E-09	CT	0.00020305	0.00040605	***	A1			
18	Feet 2D distance (mean)	A1O3	6	0.29076692	0.02228808	4.6966E-09	CT	4.7205E-05	0.0001888	***	A1	0.04166043	0.11984682	
19	Anus 1D velocity (std)	CT	6	0	0	0.01465619	CT				A1	0.03231505	0.1231285	
19	Anus 1D velocity (std)	A5	6	0.04147672	0.01181899	0.01465619	CT	0.01711293	0.05046525		A1	0.66708455	0.8891673	
19	Anus 1D velocity (std)	A1	6	0.0341328	0.01161596	0.01465619	CT	0.03231505	0.06358583		A1			
19	Anus 1D velocity (std)	A1O3	6	0.02087004	0.00462345	0.01465619	CT	0.00631789	0.02503308	*	A1	0.32633501	0.69427431	
20	Arm angle asym. (mean)	CT	6	0	0	7.664E-08	CT				A1	7.0033E-05	0.0002801	***
20	Arm angle asym. (mean)	A5	6	0.14679412	0.01491311	7.664E-08	CT	0.00018439	0.00055307	***	A1	0.00315564	0.00943709	**
20	Arm angle asym. (mean)	A1	6	0.07411869	0.00616103	7.664E-08	CT	7.0033E-05	0.0002801	***	A1			
20	Arm angle asym. (mean)	A1O3	6	0.05854297	0.0139324	7.664E-08	CT	0.00847379	0.01687578	*	A1	0.34115461	0.56592276	

Supplementary Table 10. Statistical summary of group comparisons (A1O3) for the top 20 features (identified by the XGB model) at 10 wk. One-way ANOVA was performed, followed by post-hoc comparisons using Welch's t-tests comparing each group to the reference (CT) group. Statistical significance is indicated by asterisks after Holm–Sidak correction for multiple comparisons. This PDF table displays only the top 20 features in ascending rank order for concise presentation. Additional features are included in the full version of the table, available in the Source Data file (see S_Table 10). The underlying source data for this table is provided in the same Source Data file (see S_Figure 30).

Rank	Parameter	Group	n	Mean	SEM	ANOVA p-value	Raw Post-hoc p-value	Corrected Post-hoc p-value	Significance
1	Hands 3D distance (mean)	CT	1003	1.644375387	0.013594462	2.57E-13			
1	Hands 3D distance (mean)	A5	2756	1.890718315	0.019629156	2.57E-13	1.27E-24	3.81E-24	****
1	Hands 3D distance (mean)	A1	954	1.806494177	0.02270016	2.57E-13	1.13E-09	2.26E-09	****
1	Hands 3D distance (mean)	A1O3	164	1.737294412	0.034592914	2.57E-13	0.01316317	0.01316317	*
2	Motion duration (episode)	CT	1028	7.456485084	0.150142723	3.90E-100			
2	Motion duration (episode)	A5	2826	20.90206416	0.505530649	3.90E-100	5.87E-131	1.76E-130	****
2	Motion duration (episode)	A1	968	8.811329201	0.166990617	3.90E-100	1.92E-09	3.83E-09	****
2	Motion duration (episode)	A1O3	170	7.267058823	0.230052177	3.90E-100	0.49096013	0.49096013	ns
3	Body 2D length (min)	CT	1013	4.29673504	0.034882946	1.77E-47			
3	Body 2D length (min)	A5	2797	4.858024465	0.018605098	1.77E-47	3.28E-43	9.85E-43	****
3	Body 2D length (min)	A1	959	4.654991922	0.035344274	1.77E-47	7.70E-13	1.54E-12	****
3	Body 2D length (min)	A1O3	165	4.492609563	0.090605563	1.77E-47	0.044885489	0.044885489	*
4	Tail angle (mean)	CT	1010	128.5831328	0.506514546	5.91E-183			
4	Tail angle (mean)	A5	2784	139.994748	0.217054871	5.91E-183	2.72E-83	8.16E-83	****
4	Tail angle (mean)	A1	961	128.0040494	0.524420471	5.91E-183	0.427143685	0.427143685	ns
4	Tail angle (mean)	A1O3	166	125.5143003	1.226791969	5.91E-183	0.021671597	0.042873535	*
5	Arm angle asym. (std)	CT	1004	19.70322	0.250072961	5.85E-52			
5	Arm angle asym. (std)	A5	2771	26.45002002	0.258535473	5.85E-52	2.30E-74	6.91E-74	****
5	Arm angle asym. (std)	A1	952	23.02053518	0.382920041	5.85E-52	6.22E-13	1.24E-12	****
5	Arm angle asym. (std)	A1O3	165	22.17509838	0.606602649	5.85E-52	0.000211064	0.000211064	***
6	Motion interval (episode)	CT	951	30.3361374	1.148404362	2.95E-208			
6	Motion interval (episode)	A5	2790	6.540011947	0.194037366	2.95E-208	7.20E-78	2.16E-77	****
6	Motion interval (episode)	A1	938	25.30167022	1.303416925	2.95E-208	0.003798395	0.003798395	**
6	Motion interval (episode)	A1O3	151	54.45871965	4.293563319	2.95E-208	1.92E-07	3.84E-07	****
7	Body angle (min)	CT	1007	9.707072693	0.186658265	1.09E-08			
7	Body angle (min)	A5	2796	10.47089411	0.101318344	1.09E-08	0.000332208	0.000996294	***
7	Body angle (min)	A1	960	9.410668841	0.179847705	1.09E-08	0.252963102	0.252963102	ns
7	Body angle (min)	A1O3	165	8.879004786	0.470058488	1.09E-08	0.103011896	0.195412342	ns
8	Feet 2D distance (max)	CT	1009	3.32565192	0.012419669	1.08E-58			
8	Feet 2D distance (max)	A5	2786	3.648510809	0.01165259	1.08E-58	1.78E-75	5.33E-75	****
8	Feet 2D distance (max)	A1	952	3.661384728	0.016485325	1.08E-58	1.35E-55	2.70E-55	****
8	Feet 2D distance (max)	A1O3	165	3.607997611	0.033290847	1.08E-58	1.12E-13	1.12E-13	****
9	Neck 3D velocity (std)	CT	1005	5.274273039	0.039629475	5.07E-49			
9	Neck 3D velocity (std)	A5	2779	5.409820275	0.026329312	5.07E-49	0.004433004	0.004433004	**
9	Neck 3D velocity (std)	A1	960	4.672596162	0.041934851	5.07E-49	8.18E-25	2.45E-24	****
9	Neck 3D velocity (std)	A1O3	169	4.8462384	0.080766051	5.07E-49	3.27E-06	6.55E-06	****
10	Feet 2D distance (std)	CT	1003	0.354993408	0.005679999	5.64E-102			
10	Feet 2D distance (std)	A5	2784	0.538039994	0.004836098	5.64E-102	2.42E-119	7.26E-119	****
10	Feet 2D distance (std)	A1	962	0.4260558	0.007958734	5.64E-102	5.48E-13	1.10E-12	****
10	Feet 2D distance (std)	A1O3	163	0.423828528	0.016975625	5.64E-102	0.000161749	0.000161749	***
11	Chest 3D velocity (mean)	CT	1017	4.436367595	0.047588901	2.00E-129			
11	Chest 3D velocity (mean)	A5	2807	5.569819495	0.036728201	2.00E-129	7.74E-74	2.32E-73	****
11	Chest 3D velocity (mean)	A1	967	4.19557509	0.045325613	2.00E-129	0.000254915	0.000509765	***
11	Chest 3D velocity (mean)	A1O3	169	4.239961771	0.110176932	2.00E-129	0.103070936	0.103070936	ns
12	U-body 3D length (min)	CT	1010	2.955434212	0.015108979	3.00E-15			
12	U-body 3D length (min)	A5	2786	3.081868406	0.007527144	3.00E-15	1.15E-13	3.46E-13	****
12	U-body 3D length (min)	A1	970	3.019004064	0.013368844	3.00E-15	0.001651656	0.003300585	**
12	U-body 3D length (min)	A1O3	165	3.01549293	0.034959859	3.00E-15	0.116180555	0.116180555	ns
13	Right limb angle asym. (min)	CT	1015	-47.02516898	0.524497915	1.86E-14			
13	Right limb angle asym. (min)	A5	2748	-53.62158509	0.635222849	1.86E-14	1.59E-15	4.76E-15	****
13	Right limb angle asym. (min)	A1	952	-46.56664432	0.809033294	1.86E-14	0.634449719	0.866372992	ns
13	Right limb angle asym. (min)	A1O3	165	-47.35712743	1.630669541	1.86E-14	0.846534536	0.866372992	ns
14	Hands 3D distance (max)	CT	1004	2.964436455	0.023111761	6.78E-58			
14	Hands 3D distance (max)	A5	2766	3.788195691	0.032269093	6.78E-58	1.49E-90	4.48E-90	****
14	Hands 3D distance (max)	A1	952	3.319962597	0.04014604	6.78E-58	2.94E-14	5.87E-14	****
14	Hands 3D distance (max)	A1O3	168	3.144696741	0.067151447	6.78E-58	0.011871222	0.011871222	*
15	Left limb angle asym. (min)	CT	1013	-52.23488367	0.627043819	4.69E-42			
15	Left limb angle asym. (min)	A5	2750	-67.02386119	0.709026864	4.69E-42	3.89E-53	1.17E-52	****
15	Left limb angle asym. (min)	A1	957	-56.24930235	0.949519257	4.69E-42	0.000430064	0.000859944	***
15	Left limb angle asym. (min)	A1O3	165	-53.41421194	1.614316424	4.69E-42	0.496614541	0.496614541	ns
16	Chest 1D velocity (max)	CT	1023	0.126115621	0.003979424	7.13E-44			
16	Chest 1D velocity (max)	A5	2798	0.075819707	0.001556038	7.13E-44	1.62E-30	4.87E-30	****
16	Chest 1D velocity (max)	A1	967	0.080005088	0.003143136	7.13E-44	2.35E-19	4.70E-19	****
16	Chest 1D velocity (max)	A1O3	171	0.091935497	0.00854018	7.13E-44	0.000346566	0.000346566	***
17	Leg angle asym. (std)	CT	1011	15.78164918	0.204935769	7.65E-67			
17	Leg angle asym. (std)	A5	2793	20.78733556	0.159883993	7.65E-67	9.39E-77	2.82E-76	****
17	Leg angle asym. (std)	A1	951	18.4948376	0.244881491	7.65E-67	3.91E-17	7.81E-17	****
17	Leg angle asym. (std)	A1O3	168	17.71926441	0.484264242	7.65E-67	0.000285215	0.000285215	***
18	Feet 2D distance (mean)	CT	1012	2.649405946	0.010592168	5.20E-50			
18	Feet 2D distance (mean)	A5	2782	2.621668976	0.011022254	5.20E-50	0.069706236	0.069706236	ns
18	Feet 2D distance (mean)	A1	951	2.892390144	0.013639202	5.20E-50	9.23E-43	2.77E-42	****
18	Feet 2D distance (mean)	A1O3	164	2.891877867	0.02886733	5.20E-50	1.71E-13	3.42E-13	****
19	Anus 1D velocity (std)	CT	1017	0.057159963	0.000883982	1.28E-07			
19	Anus 1D velocity (std)	A5	2790	0.051265951	0.000517814	1.28E-07	1.03E-08	3.09E-08	****
19	Anus 1D velocity (std)	A1	958	0.052957039	0.000930699	1.28E-07	0.001077572	0.002153982	**
19	Anus 1D velocity (std)	A1O3	166	0.055731648	0.002280738	1.28E-07	0.559875466	0.559875466	ns
20	Arm angle asym. (mean)	CT	1000	-4.197482711	0.423907327	1.63E-17			
20	Arm angle asym. (mean)	A5	2746	-10.00596094	0.429355191	1.63E-17	1.27E-21	3.81E-21	****
20	Arm angle asym. (mean)	A1	949	-9.427281326	0.573753871	1.63E-17	3.46E-13	6.92E-13	****
20	Arm angle asym. (mean)	A1O3	166	-2.168320228	1.243866831	1.63E-17	0.12409917	0.12409917	ns

Supplementary Table 11. Statistical summary of group comparisons (A1O2) for the top 20 features (identified by the XGB model) at 10 wk. One-way ANOVA was performed, followed by post-hoc comparisons using Welch's t-tests comparing each group to the reference (CT) group. Statistical significance is indicated by asterisks after Holm–Sidak correction for multiple comparisons. This PDF table displays only the top 20 features in ascending rank order for concise presentation. Additional features are included in the full version of the table, available in the Source Data file (see S_Table 11).

Rank	Parameter	Group	n	Mean	SEM	ANOVA p-value	Raw Post-hoc p-value	Corrected Post-hoc p-value	Significance
1	Hands 3D distance (mean)	CT	1003	1.644375387	0.013594462	1.59E-19			
1	Hands 3D distance (mean)	A5	2756	1.890718315	0.019629156	1.59E-19	1.27E-24	3.81E-24	****
1	Hands 3D distance (mean)	A1	954	1.806494177	0.02270016	1.59E-19	1.13E-09	2.26E-09	****
1	Hands 3D distance (mean)	A1O2	779	1.655849422	0.018771137	1.59E-19	0.620627182	0.620627182	ns
2	Motion duration (episode)	CT	1028	7.456485084	0.150142723	2.71E-132			
2	Motion duration (episode)	A5	2826	20.90206416	0.505530649	2.71E-132	5.87E-131	1.76E-130	****
2	Motion duration (episode)	A1	968	8.811329201	0.166990617	2.71E-132	1.92E-09	3.83E-09	****
2	Motion duration (episode)	A1O2	788	7.037817259	0.115963501	2.71E-132	0.027449947	0.027449947	*
3	Body 2D length (min)	CT	1013	4.29673504	0.034882946	3.27E-54			
3	Body 2D length (min)	A5	2797	4.858024465	0.018605098	3.27E-54	3.28E-43	9.85E-43	****
3	Body 2D length (min)	A1	959	4.654991922	0.035344274	3.27E-54	7.70E-13	1.54E-12	****
3	Body 2D length (min)	A1O2	779	4.435511094	0.04043941	3.27E-54	0.009445368	0.009445368	**
4	Tail angle (mean)	CT	1010	128.5831328	0.506514546	3.11E-202			
4	Tail angle (mean)	A5	2784	139.994748	0.217054871	3.11E-202	2.72E-83	8.16E-83	****
4	Tail angle (mean)	A1	961	128.0040494	0.524420471	3.11E-202	0.427143685	0.427143685	ns
4	Tail angle (mean)	A1O2	779	127.4945559	0.561466995	3.11E-202	0.150170582	0.277789961	ns
5	Arm angle asym. (std)	CT	1004	19.70322	0.250072961	1.05E-63			
5	Arm angle asym. (std)	A5	2771	26.45002002	0.258535473	1.05E-63	2.30E-74	6.91E-74	****
5	Arm angle asym. (std)	A1	952	23.02053518	0.382920041	1.05E-63	6.22E-13	1.24E-12	****
5	Arm angle asym. (std)	A1O2	779	20.93645444	0.354171363	1.05E-63	0.004510786	0.004510786	**
6	Motion interval (episode)	CT	951	30.3361374	1.148404362	3.60E-200			
6	Motion interval (episode)	A5	2790	6.540011947	0.194037366	3.60E-200	7.20E-78	2.16E-77	****
6	Motion interval (episode)	A1	938	25.30167022	1.303416925	3.60E-200	0.003798395	0.003798395	**
6	Motion interval (episode)	A1O2	742	38.38773585	1.753517876	3.60E-200	1.28E-04	2.56E-04	***
7	Body angle (min)	CT	1007	9.707072693	0.186658265	5.16E-08			
7	Body angle (min)	A5	2796	10.47089411	0.101318344	5.16E-08	0.000332208	0.000996294	***
7	Body angle (min)	A1	960	9.410668841	0.179847705	5.16E-08	0.252963102	0.441935874	ns
7	Body angle (min)	A1O2	779	9.6330119	0.206341107	5.16E-08	0.790136707	0.790136707	ns
8	Feet 2D distance (max)	CT	1009	3.32565192	0.012419669	2.15E-57			
8	Feet 2D distance (max)	A5	2786	3.648510809	0.01165259	2.15E-57	1.78E-75	5.33E-75	****
8	Feet 2D distance (max)	A1	952	3.661384728	0.016485325	2.15E-57	1.35E-55	2.70E-55	****
8	Feet 2D distance (max)	A1O2	779	3.652808896	0.023155866	2.15E-57	1.42E-33	1.42E-33	****
9	Neck 3D velocity (std)	CT	1005	5.274273039	0.039629475	2.96E-57			
9	Neck 3D velocity (std)	A5	2779	5.409820275	0.026329312	2.96E-57	0.004433004	0.004433004	**
9	Neck 3D velocity (std)	A1	960	4.672596162	0.041934851	2.96E-57	8.18E-25	2.45E-24	****
9	Neck 3D velocity (std)	A1O2	779	4.862038942	0.044861347	2.96E-57	8.04E-12	1.61E-11	****
10	Feet 2D distance (std)	CT	1003	0.354993408	0.005679999	1.77E-101			
10	Feet 2D distance (std)	A5	2784	0.538039994	0.004836098	1.77E-101	2.42E-119	7.26E-119	****
10	Feet 2D distance (std)	A1	962	0.4260558	0.007958734	1.77E-101	5.48E-13	1.10E-12	****
10	Feet 2D distance (std)	A1O2	779	0.431952051	0.009628263	1.77E-101	9.03E-12	9.03E-12	****
11	Chest 3D velocity (mean)	CT	1017	4.436367595	0.047588901	3.80E-153			
11	Chest 3D velocity (mean)	A5	2807	5.569819495	0.036728201	3.80E-153	7.74E-74	2.32E-73	****
11	Chest 3D velocity (mean)	A1	967	4.19557509	0.045325613	3.80E-153	0.000254915	0.000509765	***
11	Chest 3D velocity (mean)	A1O2	779	4.289104347	0.051534031	3.80E-153	0.035929001	0.035929001	*
12	U-body 3D length (min)	CT	1010	2.955434212	0.015108979	1.99E-14			
12	U-body 3D length (min)	A5	2786	3.081868406	0.007527144	1.99E-14	1.15E-13	3.46E-13	****
12	U-body 3D length (min)	A1	970	3.019004064	0.013368844	1.99E-14	0.001651656	0.001651656	**
12	U-body 3D length (min)	A1O2	779	3.042145861	0.017706144	1.99E-14	0.000201646	0.000403251	***
13	Right limb angle asym. (min)	CT	1015	-47.02516898	0.524497915	9.54E-26			
13	Right limb angle asym. (min)	A5	2748	-53.62158509	0.635222849	9.54E-26	1.59E-15	4.76E-15	****
13	Right limb angle asym. (min)	A1	952	-46.56664432	0.809033294	9.54E-26	0.634449719	0.634449719	ns
13	Right limb angle asym. (min)	A1O2	779	-43.14845347	0.6684922	9.54E-26	0.00000545	0.0000109	****
14	Hands 3D distance (max)	CT	1004	2.964436455	0.023111761	1.14E-74			
14	Hands 3D distance (max)	A5	2766	3.788195691	0.032269093	1.14E-74	1.49E-90	4.48E-90	****
14	Hands 3D distance (max)	A1	952	3.319962597	0.04014604	1.14E-74	2.94E-14	5.87E-14	****
14	Hands 3D distance (max)	A1O2	779	3.044716828	0.035709177	1.14E-74	0.059322122	0.059322122	ns
15	Left limb angle asym. (min)	CT	1013	-52.23488367	0.627043819	2.00E-50			
15	Left limb angle asym. (min)	A5	2750	-67.02386119	0.709026864	2.00E-50	3.89E-53	1.17E-52	****
15	Left limb angle asym. (min)	A1	957	-56.24930235	0.949519257	2.00E-50	0.000430064	0.000859944	***
15	Left limb angle asym. (min)	A1O2	779	-53.56420096	0.921594576	2.00E-50	0.233243273	0.233243273	ns
16	Chest 1D velocity (max)	CT	1023	0.126115621	0.003979424	1.28E-54			
16	Chest 1D velocity (max)	A5	2798	0.075819707	0.001556038	1.28E-54	1.62E-30	4.87E-30	****
16	Chest 1D velocity (max)	A1	967	0.080005088	0.003143136	1.28E-54	2.35E-19	4.70E-19	****
16	Chest 1D velocity (max)	A1O2	782	0.119880588	0.004546597	1.28E-54	0.302255233	0.302255233	ns
17	Leg angle asym. (std)	CT	1011	15.78164918	0.204935769	1.13E-80			
17	Leg angle asym. (std)	A5	2793	20.78733556	0.159883993	1.13E-80	9.39E-77	2.82E-76	****
17	Leg angle asym. (std)	A1	951	18.4948376	0.244881491	1.13E-80	3.91E-17	7.81E-17	****
17	Leg angle asym. (std)	A1O2	779	16.68298693	0.268227366	1.13E-80	0.007660495	0.007660495	**
18	Feet 2D distance (mean)	CT	1012	2.649405946	0.010592168	2.82E-64			
18	Feet 2D distance (mean)	A5	2782	2.621668976	0.011022254	2.82E-64	0.069706236	0.069706236	ns
18	Feet 2D distance (mean)	A1	951	2.892390144	0.013639202	2.82E-64	9.23E-43	2.77E-42	****
18	Feet 2D distance (mean)	A1O2	779	2.867606207	0.015985991	2.82E-64	9.34E-29	1.87E-28	****
19	Anus 1D velocity (std)	CT	1017	0.057159963	0.000883982	3.21E-15			
19	Anus 1D velocity (std)	A5	2790	0.051265951	0.000517814	3.21E-15	1.03E-08	3.09E-08	****
19	Anus 1D velocity (std)	A1	958	0.052957039	0.000930699	3.21E-15	0.001077572	0.002153982	**
19	Anus 1D velocity (std)	A1O2	779	0.059545626	0.001046782	3.21E-15	0.081830035	0.081830035	ns
20	Arm angle asym. (mean)	CT	1000	-4.197482711	0.423907327	5.39E-26			
20	Arm angle asym. (mean)	A5	2746	-10.00596094	0.429355191	5.39E-26	1.27E-21	3.81E-21	****
20	Arm angle asym. (mean)	A1	949	-9.427281326	0.573753871	5.39E-26	3.46E-13	6.92E-13	****
20	Arm angle asym. (mean)	A1O2	779	-3.117740307	0.56320718	5.39E-26	0.125793063	0.125793063	ns

Supplementary Table 12. Statistical summary of group comparisons (A1L1) for the top 20 features (identified by the XGB model) at 10 wk. One-way ANOVA was performed, followed by post-hoc comparisons using Welch's t-tests comparing each group to the reference (CT) group. Statistical significance is indicated by asterisks after Holm–Sidak correction for multiple comparisons. This PDF table displays only the top 20 features in ascending rank order for concise presentation. Additional features are included in the full version of the table, available in the Source Data file (see S_Table 12).

Rank	Parameter	Group	n	Mean	SEM	ANOVA p-value	Raw Post-hoc p-value	Corrected Post-hoc p-value	Significance
1	Hands 3D distance (mean)	CT	1003	1.644375387	0.013594462	3.42E-81			
1	Hands 3D distance (mean)	A5	2756	1.890718315	0.019629156	3.42E-81	1.27E-24	2.54E-24	****
1	Hands 3D distance (mean)	A1	954	1.806494177	0.02270016	3.42E-81	1.13E-09	1.13E-09	****
1	Hands 3D distance (mean)	A1L1	1685	1.437116406	0.008075352	3.42E-81	1.80E-37	5.39E-37	****
2	Motion duration (episode)	CT	1028	7.456485084	0.150142723	0			
2	Motion duration (episode)	A5	2826	20.90206416	0.505530649	0	5.87E-131	1.17E-130	****
2	Motion duration (episode)	A1	968	8.811329201	0.166990617	0	1.92E-09	1.92E-09	****
2	Motion duration (episode)	A1L1	1703	133.7172245	4.522502884	0	1.86E-141	5.57E-141	****
3	Body 2D length (min)	CT	1013	4.29673504	0.034882946	8.07E-68			
3	Body 2D length (min)	A5	2797	4.858024465	0.018605098	8.07E-68	3.28E-43	9.85E-43	****
3	Body 2D length (min)	A1	959	4.654991922	0.035344274	8.07E-68	7.70E-13	1.54E-12	****
3	Body 2D length (min)	A1L1	1685	4.433456128	0.022049669	8.07E-68	0.000941068	0.000941068	***
4	Tail angle (mean)	CT	1010	128.5831328	0.506514546	1.30E-142			
4	Tail angle (mean)	A5	2784	139.994748	0.217054871	1.30E-142	2.72E-83	8.16E-83	****
4	Tail angle (mean)	A1	961	128.0040494	0.524420471	1.30E-142	0.427143685	0.427143685	ns
4	Tail angle (mean)	A1L1	1685	132.8484258	0.476769255	1.30E-142	1.01E-09	2.02E-09	****
5	Arm angle asym. (std)	CT	1004	19.70322	0.250072961	7.78E-230			
5	Arm angle asym. (std)	A5	2771	26.45002002	0.258535473	7.78E-230	2.30E-74	6.91E-74	****
5	Arm angle asym. (std)	A1	952	23.02053518	0.382920041	7.78E-230	6.22E-13	6.22E-13	****
5	Arm angle asym. (std)	A1L1	1685	15.41288987	0.11340252	7.78E-230	6.55E-51	1.31E-50	****
6	Motion interval (episode)	CT	951	30.3361374	1.148404362	5.04E-246			
6	Motion interval (episode)	A5	2790	6.540011947	0.194037366	5.04E-246	7.20E-78	1.44E-77	****
6	Motion interval (episode)	A1	938	25.30167022	1.303416925	5.04E-246	0.003798395	0.003798395	**
6	Motion interval (episode)	A1L1	1685	6.081226508	0.275210103	5.04E-246	3.50E-79	1.05E-78	****
7	Body angle (min)	CT	1007	9.707072693	0.186658265	1.53E-45			
7	Body angle (min)	A5	2796	10.47089411	0.101318344	1.53E-45	0.000332208	0.000664306	***
7	Body angle (min)	A1	960	9.410668841	0.179847705	1.53E-45	0.252963102	0.252963102	ns
7	Body angle (min)	A1L1	1685	8.010351972	0.128681039	1.53E-45	1.09E-13	3.27E-13	****
8	Feet 2D distance (max)	CT	1009	3.32565192	0.012419669	2.69E-183			
8	Feet 2D distance (max)	A5	2786	3.648510809	0.01165259	2.69E-183	1.78E-75	3.55E-75	****
8	Feet 2D distance (max)	A1	952	3.661384728	0.016485325	2.69E-183	1.35E-55	1.35E-55	****
8	Feet 2D distance (max)	A1L1	1685	3.961677239	0.012080672	2.69E-183	4.20E-236	1.26E-235	****
9	Neck 3D velocity (std)	CT	1005	5.274273039	0.039629475	0			
9	Neck 3D velocity (std)	A5	2779	5.409820275	0.026329312	0	0.004433004	0.004433004	**
9	Neck 3D velocity (std)	A1	960	4.672596162	0.041934851	0	8.18E-25	1.64E-24	****
9	Neck 3D velocity (std)	A1L1	1685	7.181732064	0.057008177	0	1.67E-146	5.01E-146	****
10	Feet 2D distance (std)	CT	1003	0.354993408	0.005679999	3.40E-120			
10	Feet 2D distance (std)	A5	2784	0.538039994	0.004836098	3.40E-120	2.42E-119	7.26E-119	****
10	Feet 2D distance (std)	A1	962	0.4260558	0.007958734	3.40E-120	5.48E-13	5.48E-13	****
10	Feet 2D distance (std)	A1L1	1685	0.509152707	0.004202374	3.40E-120	5.19E-95	1.04E-94	****
11	Chest 3D velocity (mean)	CT	1017	4.436367595	0.047588901	3.56E-194			
11	Chest 3D velocity (mean)	A5	2807	5.569819495	0.036728201	3.56E-194	7.74E-74	1.55E-73	****
11	Chest 3D velocity (mean)	A1	967	4.19557509	0.045325613	3.56E-194	0.000254915	0.000254915	***
11	Chest 3D velocity (mean)	A1L1	1685	6.38420457	0.066167905	3.56E-194	1.30E-114	3.91E-114	****
12	U-body 3D length (min)	CT	1010	2.955434212	0.015108979	1.28E-139			
12	U-body 3D length (min)	A5	2786	3.081868406	0.007527144	1.28E-139	1.15E-13	2.31E-13	****
12	U-body 3D length (min)	A1	970	3.019004064	0.013368844	1.28E-139	0.001651656	0.001651656	**
12	U-body 3D length (min)	A1L1	1685	2.749324103	0.010160157	1.28E-139	8.57E-29	2.57E-28	****
13	Right limb angle asym. (min)	CT	1015	-47.02516898	0.524497915	7.42E-96			
13	Right limb angle asym. (min)	A5	2748	-53.62158509	0.635222849	7.42E-96	1.59E-15	3.17E-15	****
13	Right limb angle asym. (min)	A1	952	-46.56664432	0.809033294	7.42E-96	0.634449719	0.634449719	ns
13	Right limb angle asym. (min)	A1L1	1685	-36.30625571	0.393958787	7.42E-96	1.45E-56	4.34E-56	****
14	Hands 3D distance (max)	CT	1004	2.964436455	0.023111761	2.54E-108			
14	Hands 3D distance (max)	A5	2766	3.788195691	0.032269093	2.54E-108	1.49E-90	4.48E-90	****
14	Hands 3D distance (max)	A1	952	3.319962597	0.04014604	2.54E-108	2.94E-14	5.87E-14	****
14	Hands 3D distance (max)	A1L1	1685	3.015527435	0.014741477	2.54E-108	0.062517516	0.062517516	ns
15	Left limb angle asym. (min)	CT	1013	-52.23488367	0.627043819	9.83E-107			
15	Left limb angle asym. (min)	A5	2750	-67.02386119	0.709026864	9.83E-107	3.89E-53	1.17E-52	****
15	Left limb angle asym. (min)	A1	957	-56.24930235	0.949519257	9.83E-107	0.000430064	0.000430064	***
15	Left limb angle asym. (min)	A1L1	1685	-46.9563005	0.523711021	9.83E-107	1.27E-10	2.54E-10	****
16	Chest 1D velocity (max)	CT	1023	0.126115621	0.003979424	7.16E-64			
16	Chest 1D velocity (max)	A5	2798	0.075819707	0.001556038	7.16E-64	1.62E-30	4.87E-30	****
16	Chest 1D velocity (max)	A1	967	0.080005088	0.003143136	7.16E-64	2.35E-19	4.70E-19	****
16	Chest 1D velocity (max)	A1L1	1702	0.117193161	0.002701002	7.16E-64	0.063723091	0.063723091	ns
17	Leg angle asym. (std)	CT	1011	15.78164918	0.204935769	1.43E-105			
17	Leg angle asym. (std)	A5	2793	20.78733556	0.159883993	1.43E-105	9.39E-77	2.82E-76	****
17	Leg angle asym. (std)	A1	951	18.4948376	0.244881491	1.43E-105	3.91E-17	7.81E-17	****
17	Leg angle asym. (std)	A1L1	1685	16.91582212	0.111715179	1.43E-105	1.29E-06	1.29E-06	****
18	Feet 2D distance (mean)	CT	1012	2.649405946	0.010592168	2.69E-138			
18	Feet 2D distance (mean)	A5	2782	2.621668976	0.011022254	2.69E-138	0.069706236	0.069706236	ns
18	Feet 2D distance (mean)	A1	951	2.892390144	0.013639202	2.69E-138	9.23E-43	1.85E-42	****
18	Feet 2D distance (mean)	A1L1	1685	2.991827443	0.012287399	2.69E-138	1.41E-91	4.23E-91	****
19	Anus 1D velocity (std)	CT	1017	0.057159963	0.000883982	7.12E-10			
19	Anus 1D velocity (std)	A5	2790	0.051265951	0.000517814	7.12E-10	1.03E-08	3.09E-08	****
19	Anus 1D velocity (std)	A1	958	0.052957039	0.000930699	7.12E-10	0.001077572	0.002153982	**
19	Anus 1D velocity (std)	A1L1	1685	0.055414916	0.000625152	7.12E-10	0.107174168	0.107174168	ns
20	Arm angle asym. (mean)	CT	1000	-4.197482711	0.423907327	2.24E-69			
20	Arm angle asym. (mean)	A5	2746	-10.00596094	0.429355191	2.24E-69	1.27E-21	3.81E-21	****
20	Arm angle asym. (mean)	A1	949	-9.427281326	0.573753871	2.24E-69	3.46E-13	6.92E-13	****
20	Arm angle asym. (mean)	A1L1	1685	-0.679603656	0.230715523	2.24E-69	4.89E-13	6.92E-13	****

Supplementary Table 13. Statistical summary of group comparisons (A1L3) for the top 20 features (identified by the XGB model) at 10 wk. One-way ANOVA was performed, followed by post-hoc comparisons using Welch's t-tests comparing each group to the reference (CT) group. Statistical significance is indicated by asterisks after Holm–Sidak correction for multiple comparisons. This PDF table displays only the top 20 features in ascending rank order for concise presentation. Additional features are included in the full version of the table, available in the Source Data file (see S_Table 13).

Rank	Parameter	Group	n	Mean	SEM	ANOVA p-value	Raw Post-hoc p-value	Corrected Post-hoc p-value	Significance
1	Hands 3D distance (mean)	CT	1003	1.644375387	0.013594462	6.47E-96			
1	Hands 3D distance (mean)	A5	2756	1.890718315	0.019629156	6.47E-96	1.27E-24	2.54E-24	****
1	Hands 3D distance (mean)	A1	954	1.806494177	0.02270016	6.47E-96	1.13E-09	1.13E-09	****
1	Hands 3D distance (mean)	A1L3	1726	1.397125157	0.008789913	6.47E-96	1.17E-49	3.51E-49	****
2	Motion duration (episode)	CT	1028	7.456485084	0.150142723	2.05E-201			
2	Motion duration (episode)	A5	2826	20.90206416	0.505530649	2.05E-201	5.87E-131	1.17E-130	****
2	Motion duration (episode)	A1	968	8.811329201	0.166990617	2.05E-201	1.92E-09	1.92E-09	****
2	Motion duration (episode)	A1L3	1745	36.90492837	0.990676374	2.05E-201	9.44E-156	2.83E-155	****
3	Body 2D length (min)	CT	1013	4.29673504	0.034882946	2.01E-51			
3	Body 2D length (min)	A5	2797	4.858024465	0.018605098	2.01E-51	3.28E-43	9.85E-43	****
3	Body 2D length (min)	A1	959	4.654991922	0.035344274	2.01E-51	7.70E-13	7.70E-13	****
3	Body 2D length (min)	A1L3	1726	4.692933611	0.020230415	2.01E-51	3.39E-22	6.78E-22	****
4	Tail angle (mean)	CT	1010	128.5831328	0.506514546	1.53E-202			
4	Tail angle (mean)	A5	2784	139.994748	0.217054871	1.53E-202	2.72E-83	8.16E-83	****
4	Tail angle (mean)	A1	961	128.0040494	0.524420471	1.53E-202	0.427143685	0.427143685	ns
4	Tail angle (mean)	A1L3	1726	137.3590979	0.243169267	1.53E-202	4.99E-51	9.97E-51	****
5	Arm angle asym. (std)	CT	1004	19.70322	0.250072961	1.11E-212			
5	Arm angle asym. (std)	A5	2771	26.45002002	0.258535473	1.11E-212	2.30E-74	6.91E-74	****
5	Arm angle asym. (std)	A1	952	23.02053518	0.382920041	1.11E-212	6.22E-13	6.22E-13	****
5	Arm angle asym. (std)	A1L3	1726	15.86987761	0.138220477	1.11E-212	5.39E-39	1.08E-38	****
6	Motion interval (episode)	CT	951	30.3361374	1.148404362	1.54E-243			
6	Motion interval (episode)	A5	2790	6.540011947	0.194037366	1.54E-243	7.20E-78	1.56E-77	****
6	Motion interval (episode)	A1	938	25.30167022	1.303416925	1.54E-243	0.003798395	0.003798395	**
6	Motion interval (episode)	A1L3	1719	6.2277293	0.296629255	1.54E-243	5.18E-78	1.56E-77	****
7	Body angle (min)	CT	1007	9.707072693	0.186658265	3.86E-37			
7	Body angle (min)	A5	2796	10.47089411	0.101318344	3.86E-37	0.000332208	0.000664306	***
7	Body angle (min)	A1	960	9.410668841	0.179847705	3.86E-37	0.252963102	0.252963102	ns
7	Body angle (min)	A1L3	1726	8.27069997	0.127397957	3.86E-37	2.58E-10	7.75E-10	****
8	Feet 2D distance (max)	CT	1009	3.32565192	0.012419669	1.17E-74			
8	Feet 2D distance (max)	A5	2786	3.648510809	0.01165259	1.17E-74	1.78E-75	3.55E-75	****
8	Feet 2D distance (max)	A1	952	3.661384728	0.016485325	1.17E-74	1.35E-55	1.35E-55	****
8	Feet 2D distance (max)	A1L3	1726	3.671642588	0.00948565	1.17E-74	8.01E-98	2.40E-97	****
9	Neck 3D velocity (std)	CT	1005	5.274273039	0.039629475	4.50E-171			
9	Neck 3D velocity (std)	A5	2779	5.409820275	0.026329312	4.50E-171	0.004433004	0.004433004	**
9	Neck 3D velocity (std)	A1	960	4.672596162	0.041934851	4.50E-171	8.18E-25	1.64E-24	****
9	Neck 3D velocity (std)	A1L3	1726	6.301795622	0.043295624	4.50E-171	5.22E-65	1.57E-64	****
10	Feet 2D distance (std)	CT	1003	0.354993408	0.005679999	1.29E-116			
10	Feet 2D distance (std)	A5	2784	0.538039994	0.004836098	1.29E-116	2.42E-119	7.26E-119	****
10	Feet 2D distance (std)	A1	962	0.4260558	0.007958734	1.29E-116	5.48E-13	5.48E-13	****
10	Feet 2D distance (std)	A1L3	1726	0.452737241	0.004333191	1.29E-116	7.06E-41	1.41E-40	****
11	Chest 3D velocity (mean)	CT	1017	4.436367595	0.047588901	4.21E-126			
11	Chest 3D velocity (mean)	A5	2807	5.569819495	0.036728201	4.21E-126	7.74E-74	2.32E-73	****
11	Chest 3D velocity (mean)	A1	967	4.19557509	0.045325613	4.21E-126	0.000254915	0.000254915	***
11	Chest 3D velocity (mean)	A1L3	1726	5.402486167	0.046724558	4.21E-126	9.5E-46	1.9E-45	****
12	U-body 3D length (min)	CT	1010	2.955434212	0.015108979	1.43E-49			
12	U-body 3D length (min)	A5	2786	3.081868406	0.007527144	1.43E-49	1.15E-13	3.46E-13	****
12	U-body 3D length (min)	A1	970	3.019004064	0.013368844	1.43E-49	0.001651656	0.001651656	**
12	U-body 3D length (min)	A1L3	1726	2.891692038	0.010089688	1.43E-49	0.000461271	0.00092233	***
13	Right limb angle asym. (min)	CT	1015	-47.02516898	0.524497915	2.95E-52			
13	Right limb angle asym. (min)	A5	2748	-53.62158509	0.635222849	2.95E-52	1.59E-15	3.17E-15	****
13	Right limb angle asym. (min)	A1	952	-46.56664432	0.809033294	2.95E-52	0.634449719	0.634449719	ns
13	Right limb angle asym. (min)	A1L3	1726	-40.95320292	0.466750166	2.95E-52	9.45E-18	2.83E-17	****
14	Hands 3D distance (max)	CT	1004	2.964436455	0.023111761	5.11E-129			
14	Hands 3D distance (max)	A5	2766	3.788195691	0.032269093	5.11E-129	1.49E-90	4.48E-90	****
14	Hands 3D distance (max)	A1	952	3.319962597	0.04014604	5.11E-129	2.94E-14	5.87E-14	****
14	Hands 3D distance (max)	A1L3	1726	2.889187878	0.016779638	5.11E-129	0.008485821	0.008485821	**
15	Left limb angle asym. (min)	CT	1013	-52.23488367	0.627043819	3.88E-186			
15	Left limb angle asym. (min)	A5	2750	-67.02386119	0.709026864	3.88E-186	3.89E-53	7.79E-53	****
15	Left limb angle asym. (min)	A1	957	-56.24930235	0.949519257	3.88E-186	0.000430064	0.000430064	***
15	Left limb angle asym. (min)	A1L3	1726	-40.25531735	0.369997218	3.88E-186	1.33E-56	4E-56	****
16	Chest 1D velocity (max)	CT	1023	0.126115621	0.003979424	1.12E-108			
16	Chest 1D velocity (max)	A5	2798	0.075819707	0.001556038	1.12E-108	1.62E-30	4.87E-30	****
16	Chest 1D velocity (max)	A1	967	0.080005088	0.003143136	1.12E-108	2.35E-19	4.7E-19	****
16	Chest 1D velocity (max)	A1L3	1738	0.139301375	0.002734419	1.12E-108	0.006372626	0.006372626	**
17	Leg angle asym. (std)	CT	1011	15.78164918	0.204935769	3.40E-177			
17	Leg angle asym. (std)	A5	2793	20.78733556	0.159883993	3.40E-177	9.39E-77	2.82E-76	****
17	Leg angle asym. (std)	A1	951	18.4948376	0.244881491	3.40E-177	3.91E-17	7.81E-17	****
17	Leg angle asym. (std)	A1L3	1726	14.77006225	0.110950367	3.40E-177	0.0000151	0.0000151	****
18	Feet 2D distance (mean)	CT	1012	2.649405946	0.010592168	4.40E-130			
18	Feet 2D distance (mean)	A5	2782	2.621668976	0.011022254	4.40E-130	0.069706236	0.069706236	ns
18	Feet 2D distance (mean)	A1	951	2.892390144	0.013639202	4.40E-130	9.23E-43	1.85E-42	****
18	Feet 2D distance (mean)	A1L3	1726	2.947580946	0.008989402	4.40E-130	2.83E-93	8.48E-93	****
19	Anus 1D velocity (std)	CT	1017	0.057159963	0.000883982	1.62E-09			
19	Anus 1D velocity (std)	A5	2790	0.051265951	0.000517814	1.62E-09	1.03E-08	2.06E-08	****
19	Anus 1D velocity (std)	A1	958	0.052957039	0.000930699	1.62E-09	0.001077572	0.001077572	**
19	Anus 1D velocity (std)	A1L3	1726	0.050914761	0.00053126	1.62E-09	1.71E-09	5.13E-09	****
20	Arm angle asym. (mean)	CT	1000	-4.197482711	0.423907327	1.20E-22			
20	Arm angle asym. (mean)	A5	2746	-10.00596094	0.429355191	1.20E-22	1.27E-21	3.81E-21	****
20	Arm angle asym. (mean)	A1	949	-9.427281326	0.573753871	1.20E-22	3.46E-13	6.92E-13	****
20	Arm angle asym. (mean)	A1L3	1726	-5.971498727	0.274008937	1.20E-22	0.000451161	0.000451161	***

Supplementary Table 14. Statistical summary of group comparisons (A1BT) for the top 20 features (identified by the XGB model) at 6 wk. One-way ANOVA was performed, followed by post-hoc comparisons using Welch's t-tests comparing each group to the reference (CT) group. Statistical significance is indicated by asterisks after Holm–Sidak correction for multiple comparisons. This PDF table displays only the top 20 features in ascending rank order for concise presentation. Additional features are included in the full version of the table, available in the Source Data file (see S_Table 14).

Rank	Parameter	Group	n	Mean	SEM	ANOVA p-value	Raw Post-hoc p-value	Corrected Post-hoc p-value	Significance
1	Hands 3D distance (mean)	CT	1069	1.603772965	0.012843997	1.92E-21			
1	Hands 3D distance (mean)	A5	2207	1.83267426	0.01870961	1.92E-21	1.40E-23	4.19E-23	****
1	Hands 3D distance (mean)	A1	672	1.884179871	0.026054661	1.92E-21	3.86E-21	7.71E-21	****
1	Hands 3D distance (mean)	A1BT	275	1.974157067	0.046282984	1.92E-21	1.62E-13	1.62E-13	****
2	Motion duration (episode)	CT	1084	8.053874539	0.144599774	4.38E-93			
2	Motion duration (episode)	A5	2250	17.68625185	0.429481008	4.38E-93	7.19E-93	2.16E-92	****
2	Motion duration (episode)	A1	690	7.938550725	0.157098354	4.38E-93	5.89E-01	5.89E-01	ns
2	Motion duration (episode)	A1BT	278	7.345923261	0.171456564	4.38E-93	0.001664058	0.003325347	**
3	Body 2D length (min)	CT	1076	4.294935601	0.033240268	1.04E-68			
3	Body 2D length (min)	A5	2222	4.953072469	0.020667685	1.04E-68	2.62E-59	7.86E-59	****
3	Body 2D length (min)	A1	677	4.969226882	0.041398275	1.04E-68	4.05E-35	8.09E-35	****
3	Body 2D length (min)	A1BT	275	4.893389703	0.062184591	1.04E-68	3.2E-16	3.2E-16	****
4	Tail angle (mean)	CT	1072	129.5058236	0.511718267	3.76E-45			
4	Tail angle (mean)	A5	2243	136.4137491	0.248165627	3.76E-45	1.57E-32	4.7E-32	****
4	Tail angle (mean)	A1	680	130.3714011	0.632460453	3.76E-45	0.287525885	0.287525885	ns
4	Tail angle (mean)	A1BT	275	135.0937194	0.948876105	3.76E-45	0.000000331	0.000000662	****
5	Arm angle asym. (std)	CT	1068	19.1068203	0.234844945	3.01E-61			
5	Arm angle asym. (std)	A5	2223	26.53300752	0.283883771	3.01E-61	3.82E-85	1.15E-84	****
5	Arm angle asym. (std)	A1	670	23.20498188	0.427951381	3.01E-61	1.46E-16	2.91E-16	****
5	Arm angle asym. (std)	A1BT	275	22.80722024	0.74808557	3.01E-61	0.0000035	0.0000035	****
6	Motion interval (episode)	CT	1007	34.0734194	1.322395702	3.93E-145			
6	Motion interval (episode)	A5	2169	9.22978331	0.319580596	3.93E-145	1.89E-65	5.66E-65	****
6	Motion interval (episode)	A1	635	34.77790026	1.860769164	3.93E-145	0.757675216	0.757675216	ns
6	Motion interval (episode)	A1BT	262	56.98498728	4.697384412	3.93E-145	4.04E-06	8.08E-06	****
7	Body angle (min)	CT	1078	9.415275187	0.17069152	2.43E-13			
7	Body angle (min)	A5	2229	10.75345865	0.114779784	2.43E-13	9.67E-11	2.9E-10	****
7	Body angle (min)	A1	687	10.27424513	0.220561713	2.43E-13	0.002110284	0.004216115	**
7	Body angle (min)	A1BT	275	8.7722321	0.340493681	2.43E-13	0.092092518	0.092092518	ns
8	Feet 2D distance (max)	CT	1069	3.263696693	0.011716439	5.27E-114			
8	Feet 2D distance (max)	A5	2206	3.706958636	0.013667765	5.27E-114	1.53E-122	4.58E-122	****
8	Feet 2D distance (max)	A1	682	3.786898764	0.021444245	5.27E-114	3.94E-85	7.88E-85	****
8	Feet 2D distance (max)	A1BT	275	3.805269456	0.032791501	5.27E-114	9.34E-42	9.34E-42	****
9	Neck 3D velocity (std)	CT	1071	5.146877547	0.036935531	1.12E-20			
9	Neck 3D velocity (std)	A5	2218	5.048163364	0.033055341	1.12E-20	0.046525177	0.046525177	*
9	Neck 3D velocity (std)	A1	679	4.676584494	0.045768185	1.12E-20	2.59E-15	5.17E-15	****
9	Neck 3D velocity (std)	A1BT	275	4.424208859	0.061006811	1.12E-20	4.70E-22	1.41E-21	****
10	Feet 2D distance (std)	CT	1072	0.332793154	0.005362826	8.64E-123			
10	Feet 2D distance (std)	A5	2215	0.540075046	0.005256303	8.64E-123	7.99E-149	2.4E-148	****
10	Feet 2D distance (std)	A1	681	0.460898905	0.009876088	8.64E-123	1.67E-28	3.33E-28	****
10	Feet 2D distance (std)	A1BT	275	0.399309764	0.012838422	8.64E-123	0.00000251	0.00000251	****
11	Chest 3D velocity (mean)	CT	1060	4.288244385	0.043998618	4.16E-75			
11	Chest 3D velocity (mean)	A5	2202	5.20800762	0.040982385	4.16E-75	9.94E-51	2.98E-50	****
11	Chest 3D velocity (mean)	A1	689	4.236451651	0.05336434	4.16E-75	0.454070605	0.454070605	ns
11	Chest 3D velocity (mean)	A1BT	275	3.978245064	0.069353899	4.16E-75	0.000179005	0.000357977	***
12	U-body 3D length (min)	CT	1075	2.995992593	0.015157316	1.23E-07			
12	U-body 3D length (min)	A5	2249	3.077956794	0.008160331	1.23E-07	2.08E-06	6.25E-06	****
12	U-body 3D length (min)	A1	683	3.092802131	0.017993564	1.23E-07	0.0000408	0.0000817	****
12	U-body 3D length (min)	A1BT	275	3.006077724	0.024868417	1.23E-07	0.729272916	0.729272916	ns
13	Right limb angle asym. (min)	CT	1077	-45.91622977	0.519673947	8.79E-20			
13	Right limb angle asym. (min)	A5	2195	-54.0623466	0.708513616	8.79E-20	3.26E-20	9.77E-20	****
13	Right limb angle asym. (min)	A1	674	-44.90232162	0.897592754	8.79E-20	0.32849979	0.32849979	ns
13	Right limb angle asym. (min)	A1BT	275	-48.91168209	1.497266446	8.79E-20	0.059600277	0.115648361	ns
14	Hands 3D distance (max)	CT	1062	2.86732305	0.021809472	4.87E-55			
14	Hands 3D distance (max)	A5	2217	3.671713291	0.0335437	4.87E-55	7.48E-85	2.24E-84	****
14	Hands 3D distance (max)	A1	674	3.397134449	0.045412025	4.87E-55	1.37E-24	2.74E-24	****
14	Hands 3D distance (max)	A1BT	275	3.480316605	0.08015186	4.87E-55	1.42E-12	1.42E-12	****
15	Left limb angle asym. (min)	CT	1067	-51.86531218	0.592560605	1.16E-23			
15	Left limb angle asym. (min)	A5	2216	-63.59344492	0.766107819	1.16E-23	4.82E-33	1.45E-32	****
15	Left limb angle asym. (min)	A1	671	-55.6889893	1.112611708	1.16E-23	0.002478385	0.002478385	**
15	Left limb angle asym. (min)	A1BT	275	-60.093686	1.953142593	1.16E-23	0.0000691	0.000138132	***
16	Chest 1D velocity (max)	CT	1089	0.127478457	0.003795931	1.59E-56			
16	Chest 1D velocity (max)	A5	2227	0.069521559	0.001725213	1.59E-56	1.74E-41	5.23E-41	****
16	Chest 1D velocity (max)	A1	690	0.076083333	0.003555781	1.59E-56	1.94E-22	3.89E-22	****
16	Chest 1D velocity (max)	A1BT	278	0.079627518	0.005883213	1.59E-56	2.26E-11	2.26E-11	****
17	Leg angle asym. (std)	CT	1071	15.23515896	0.210870799	6.02E-93			
17	Leg angle asym. (std)	A5	2196	21.44957666	0.173589443	6.02E-93	3.65E-104	1.1E-103	****
17	Leg angle asym. (std)	A1	676	20.44932951	0.312169298	6.02E-93	1.13E-40	2.26E-40	****
17	Leg angle asym. (std)	A1BT	275	19.67860716	0.559403365	6.02E-93	8.01E-13	8.01E-13	****
18	Feet 2D distance (mean)	CT	1073	2.63770504	0.009408228	1.37E-68			
18	Feet 2D distance (mean)	A5	2196	2.647969824	0.012871051	1.37E-68	0.519722261	0.519722261	ns
18	Feet 2D distance (mean)	A1	674	2.941242674	0.018097523	1.37E-68	1.51E-45	4.53E-45	****
18	Feet 2D distance (mean)	A1BT	275	3.080237852	0.029609185	1.37E-68	3.03E-36	6.07E-36	****
19	Anus 1D velocity (std)	CT	1073	0.053830455	0.000839097	6.01E-03			
19	Anus 1D velocity (std)	A5	2219	0.051477174	0.00059768	6.01E-03	2.25E-02	4.44E-02	*
19	Anus 1D velocity (std)	A1	677	0.051945639	0.001111663	6.01E-03	0.176193576	0.176193576	ns
19	Anus 1D velocity (std)	A1BT	275	0.047472379	0.001618195	6.01E-03	0.000536297	0.001608027	**
20	Arm angle asym. (mean)	CT	1072	-3.074188838	0.401693733	3.03E-18			
20	Arm angle asym. (mean)	A5	2187	-8.545298082	0.459235766	3.03E-18	5.14E-19	1.54E-18	****
20	Arm angle asym. (mean)	A1	669	-4.538058081	0.674366965	3.03E-18	6.24E-02	1.21E-01	ns
20	Arm angle asym. (mean)	A1BT	275	-1.488877625	1.115096387	3.03E-18	0.181918513	0.181918513	ns

Supplementary Table 15. Statistical summary of treatment group comparisons for the top 20 features (identified by the XGB model) at endpoints. One-way ANOVA was performed, followed by post-hoc comparisons using Welch's t-tests comparing each group to the reference (CT) group. Statistical significance is indicated by asterisks after Holm–Sidak correction for multiple comparisons. The top 20 features are listed in ascending rank order, with additional features appended afterwards.

Rank	Parameter	Group	Global_name	Souce_Table#	Week	n	Mean	SEM	Significance	norm_value	norm_log
1	Hands 3D distance (mean)	CT	A1O3_CT	9	10	1003	1.64	0.01		0.43	0.47
1	Hands 3D distance (mean)	A5	A1O3_A5	9	10	2756	1.89	0.02	****	0.86	0.88
1	Hands 3D distance (mean)	A1	A1O3_A1	9	10	954	1.81	0.02	****	0.71	0.74
1	Hands 3D distance (mean)	A1O2	A1O2	10	10	779	1.66	0.02	ns	0.45	0.49
1	Hands 3D distance (mean)	A1O3	A1O3	9	10	164	1.74	0.03	*	0.59	0.63
1	Hands 3D distance (mean)	A1L1	A1L1	11	10	1685	1.44	0.01	****	0.07	0.08
1	Hands 3D distance (mean)	A1L3	A1L3	12	10	1726	1.40	0.01	****	0.00	0.00
1	Hands 3D distance (mean)	CT	A1BT_CT	13	6	1069	1.60	0.01		0.36	0.40
1	Hands 3D distance (mean)	A5	A1BT_A5	13	6	2207	1.83	0.02	****	0.75	0.78
1	Hands 3D distance (mean)	A1	A1BT_A1	13	6	672	1.88	0.03	****	0.84	0.87
1	Hands 3D distance (mean)	A1BT	A1BT	13	6	275	1.97	0.05	****	1.00	1.00
2	Motion duration (episode)	CT	A1O3_CT	9	10	1028	7.46	0.15		0.00	0.02
2	Motion duration (episode)	A5	A1O3_A5	9	10	2826	20.90	0.51	****	0.11	0.37
2	Motion duration (episode)	A1	A1O3_A1	9	10	968	8.81	0.17	****	0.01	0.08
2	Motion duration (episode)	A1O2	A1O2	10	10	788	7.04	0.12	*	0.00	0.00
2	Motion duration (episode)	A1O3	A1O3	9	10	170	7.27	0.23	ns	0.00	0.01
2	Motion duration (episode)	A1L1	A1L1	11	10	1703	133.72	4.52	****	1.00	1.00
2	Motion duration (episode)	A1L3	A1L3	12	10	1745	36.90	0.99	****	0.24	0.56
2	Motion duration (episode)	CT	A1BT_CT	13	6	1084	8.05	0.14		0.01	0.05
2	Motion duration (episode)	A5	A1BT_A5	13	6	2250	17.69	0.43	****	0.08	0.31
2	Motion duration (episode)	A1	A1BT_A1	13	6	690	7.94	0.16	ns	0.01	0.04
2	Motion duration (episode)	A1BT	A1BT	13	6	278	7.35	0.17	**	0.00	0.01
3	Body 2D length (min)	CT	A1O3_CT	9	10	1013	4.30	0.03		0.00	0.00
3	Body 2D length (min)	A5	A1O3_A5	9	10	2797	4.86	0.02	****	0.84	0.84
3	Body 2D length (min)	A1	A1O3_A1	9	10	959	4.65	0.04	****	0.53	0.55
3	Body 2D length (min)	A1O2	A1O2	10	10	779	4.44	0.04	**	0.21	0.22
3	Body 2D length (min)	A1O3	A1O3	9	10	165	4.49	0.09	*	0.29	0.31
3	Body 2D length (min)	A1L1	A1L1	11	10	1685	4.43	0.02	***	0.21	0.22
3	Body 2D length (min)	A1L3	A1L3	12	10	1726	4.69	0.02	****	0.59	0.61
3	Body 2D length (min)	CT	A1BT_CT	13	6	1076	4.29	0.03		0.00	0.00
3	Body 2D length (min)	A5	A1BT_A5	13	6	2222	4.95	0.02	****	0.98	0.98
3	Body 2D length (min)	A1	A1BT_A1	13	6	677	4.97	0.04	****	1.00	1.00
3	Body 2D length (min)	A1BT	A1BT	13	6	275	4.89	0.06	****	0.89	0.89
4	Tail angle (mean)	CT	A1O3_CT	9	10	1010	128.58	0.51		0.21	0.22
4	Tail angle (mean)	A5	A1O3_A5	9	10	2784	139.99	0.22	****	1.00	1.00
4	Tail angle (mean)	A1	A1O3_A1	9	10	961	128.00	0.52	ns	0.17	0.18
4	Tail angle (mean)	A1O2	A1O2	10	10	779	127.49	0.56	ns	0.14	0.14
4	Tail angle (mean)	A1O3	A1O3	9	10	166	125.51	1.23	*	0.00	0.00
4	Tail angle (mean)	A1L1	A1L1	11	10	1685	132.85	0.48	****	0.51	0.52
4	Tail angle (mean)	A1L3	A1L3	12	10	1726	137.36	0.24	****	0.82	0.83
4	Tail angle (mean)	CT	A1BT_CT	13	6	1072	129.51	0.51		0.28	0.29
4	Tail angle (mean)	A5	A1BT_A5	13	6	2243	136.41	0.25	****	0.75	0.76
4	Tail angle (mean)	A1	A1BT_A1	13	6	680	130.37	0.63	ns	0.34	0.35
4	Tail angle (mean)	A1BT	A1BT	13	6	275	135.09	0.95	****	0.66	0.67
5	Arm angle asym. (std)	CT	A1O3_CT	9	10	1004	19.70	0.25		0.39	0.45
5	Arm angle asym. (std)	A5	A1O3_A5	9	10	2771	26.45	0.26	****	0.99	0.99
5	Arm angle asym. (std)	A1	A1O3_A1	9	10	952	23.02	0.38	****	0.68	0.74
5	Arm angle asym. (std)	A1O2	A1O2	10	10	779	20.94	0.35	**	0.50	0.56
5	Arm angle asym. (std)	A1O3	A1O3	9	10	165	22.18	0.61	***	0.61	0.67
5	Arm angle asym. (std)	A1L1	A1L1	11	10	1685	15.41	0.11	****	0.00	0.00
5	Arm angle asym. (std)	A1L3	A1L3	12	10	1726	15.87	0.14	****	0.04	0.05
5	Arm angle asym. (std)	CT	A1BT_CT	13	6	1068	19.11	0.23		0.33	0.40
5	Arm angle asym. (std)	A5	A1BT_A5	13	6	2223	26.53	0.28	****	1.00	1.00
5	Arm angle asym. (std)	A1	A1BT_A1	13	6	670	23.20	0.43	****	0.70	0.75
5	Arm angle asym. (std)	A1BT	A1BT	13	6	275	22.81	0.75	****	0.66	0.72
6	Motion interval (episode)	CT	A1O3_CT	9	10	951	30.34	1.15		0.48	0.72
6	Motion interval (episode)	A5	A1O3_A5	9	10	2790	6.54	0.19	****	0.01	0.03
6	Motion interval (episode)	A1	A1O3_A1	9	10	938	25.30	1.30	**	0.38	0.64
6	Motion interval (episode)	A1O2	A1O2	10	10	742	38.39	1.75	***	0.63	0.82
6	Motion interval (episode)	A1O3	A1O3	9	10	151	54.46	4.29	****	0.95	0.98
6	Motion interval (episode)	A1L1	A1L1	11	10	1685	6.08	0.28	****	0.00	0.00
6	Motion interval (episode)	A1L3	A1L3	12	10	1719	6.23	0.30	****	0.00	0.01
6	Motion interval (episode)	CT	A1BT_CT	13	6	1007	34.07	1.32		0.55	0.77
6	Motion interval (episode)	A5	A1BT_A5	13	6	2169	9.23	0.32	****	0.06	0.19
6	Motion interval (episode)	A1	A1BT_A1	13	6	635	34.78	1.86	ns	0.56	0.78
6	Motion interval (episode)	A1BT	A1BT	13	6	262	56.98	4.70	****	1.00	1.00
7	Body angle (min)	CT	A1O3_CT	9	10	1007	9.71	0.19		0.62	0.65
7	Body angle (min)	A5	A1O3_A5	9	10	2796	10.47	0.10	***	0.90	0.91
7	Body angle (min)	A1	A1O3_A1	9	10	960	9.41	0.18	ns	0.51	0.55
7	Body angle (min)	A1O2	A1O2	10	10	779	9.63	0.21	ns	0.59	0.63
7	Body angle (min)	A1O3	A1O3	9	10	165	8.88	0.47	ns	0.32	0.35
7	Body angle (min)	A1L1	A1L1	11	10	1685	8.01	0.13	****	0.00	0.00
7	Body angle (min)	A1L3	A1L3	12	10	1726	8.27	0.13	****	0.09	0.11
7	Body angle (min)	CT	A1BT_CT	13	6	1078	9.42	0.17		0.51	0.55
7	Body angle (min)	A5	A1BT_A5	13	6	2229	10.75	0.11	****	1.00	1.00
7	Body angle (min)	A1	A1BT_A1	13	6	687	10.27	0.22	**	0.83	0.85
7	Body angle (min)	A1BT	A1BT	13	6	275	8.77	0.34	ns	0.28	0.31
8	Feet 2D distance (max)	CT	A1O3_CT	9	10	1009	3.33	0.01		0.09	0.10
8	Feet 2D distance (max)	A5	A1O3_A5	9	10	2786	3.65	0.01	****	0.55	0.58
8	Feet 2D distance (max)	A1	A1O3_A1	9	10	952	3.66	0.02	****	0.57	0.59
8	Feet 2D distance (max)	A1O2	A1O2	10	10	779	3.65	0.02	****	0.56	0.58
8	Feet 2D distance (max)	A1O3	A1O3	9	10	165	3.61	0.03	****	0.49	0.52
8	Feet 2D distance (max)	A1L1	A1L1	11	10	1685	3.96	0.01	****	1.00	1.00
8	Feet 2D distance (max)	A1L3	A1L3	12	10	1726	3.67	0.01	****	0.58	0.61
8	Feet 2D distance (max)	CT	A1BT_CT	13	6	1069	3.26	0.01		0.00	0.00
8	Feet 2D distance (max)	A5	A1BT_A5	13	6	2206	3.71	0.01	****	0.64	0.66
8	Feet 2D distance (max)	A1	A1BT_A1	13	6	682	3.79	0.02	****	0.75	0.77
8	Feet 2D distance (max)	A1BT	A1BT	13	6	275	3.81	0.03	****	0.78	0.79
9	Neck 3D velocity (std)	CT	A1O3_CT	9	10	1005	5.27	0.04		0.31	0.36
9	Neck 3D velocity (std)	A5	A1O3_A5	9	10	2779	5.41	0.03	**	0.36	0.42
9	Neck 3D velocity (std)	A1	A1O3_A1	9	10	960	4.67	0.04	****	0.09	0.11
9	Neck 3D velocity (std)	A1O2	A1O2	10	10	779	4.86	0.04	****	0.16	0.19
9	Neck 3D velocity (std)	A1O3	A1O3	9	10	169	4.85	0.08	****	0.15	0.19
9	Neck 3D velocity (std)	A1L1	A1L1	11	10	1685	7.18	0.06	****	1.00	1.00
9	Neck 3D velocity (std)	A1L3	A1L3	12	10	1726	6.30	0.04	****	0.68	0.73
9	Neck 3D velocity (std)	CT	A1BT_CT	13	6	1071	5.15	0.04		0.26	0.31
9	Neck 3D velocity (std)	A5	A1BT_A5	13	6	2218	5.05	0.03	*	0.23	0.27
9	Neck 3D velocity (std)	A1	A1BT_A1	13	6	679	4.68	0.05	****	0.09	0.11
9	Neck 3D velocity (std)	A1BT	A1BT	13	6	275	4.42	0.06	****	0.00	0.00
10	Feet 2D distance (std)	CT	A1O3_CT	9	10	1003	0.35	0.01		0.11	0.13
10	Feet 2D distance (std)	A5	A1O3_A5	9	10	2784	0.54	0.00	****	0.99	0.99
10	Feet 2D distance (std)	A1	A1O3_A1	9	10	962	0.43	0.01	****	0.45	0.51
10	Feet 2D distance (std)	A1O2	A1O2	10	10	779	0.43	0.01	****	0.48	0.54
10	Feet 2D distance (std)	A1O3	A1O3	9	10	163	0.42	0.02	***	0.44	0.50
10	Feet 2D distance (std)	A1L1	A1L1	11	10	1685	0.51	0.00	****	0.85	0.88
10	Feet 2D distance (std)	A1L3	A1L3	12	10	1726	0.45	0.00	****	0.58	0.64
10	Feet 2D distance (std)	CT	A1BT_CT	13	6	1072	0.33	0.01		0.00	0.00
10	Feet 2D distance (std)	A5	A1BT_A5	13	6	2215	0.54	0.01	****	1.00	1.00
10	Feet 2D distance (std)	A1	A1BT_A1	13	6	681	0.46	0.01	****	0.62	0.67
10	Feet 2D distance (std)	A1BT	A1BT	13	6	275	0.40	0.01	****	0.32	0.38
11	Chest 3D velocity (mean)	CT	A1O3_CT	9	10	1017	4.44	0.05		0.19	0.23
11	Chest 3D velocity (mean)	A5	A1O3_A5	9	10	2807	5.57	0.04	****	0.66	0.71
11	Chest 3D velocity (mean)	A1	A1O3_A1	9	10	967	4.20	0.05	***	0.09	0.11
11	Chest 3D velocity (mean)	A1O2	A1O2	10	10	779	4.29	0.05	*	0.13	0.16
11	Chest 3D velocity (mean)	A1O3	A1O3	9	10	169	4.24	0.11	ns	0.11	0.13
11	Chest 3D velocity (mean)	A1L1	A1L1	11	10	1685	6.38	0.07	****	1.00	1.00
11	Chest 3D velocity (mean)	A1L3	A1L3	12	10	1726	5.40	0.05	****	0.59	0.65
11	Chest 3D velocity (mean)	CT	A1BT_CT	13	6	1060	4.29	0.04		0.13	0.16
11	Chest 3D velocity (mean)	A5	A1BT_A5	13	6	2202	5.21	0.04	****	0.51	0.57
11	Chest 3D velocity (mean)	A1	A1BT_A1	13	6	689	4.24	0.05	ns	0.11	0.13
11	Chest 3D velocity (mean)	A1BT	A1BT	13	6	275	3.98	0.07	***	0.00	0.00
12	U-body 3D length (min)	CT	A1O3_CT	9	10	1010	2.96	0.02		0.60	0.61
12	U-body 3D length (min)	A5	A1O3_A5	9	10	2786	3.08	0.01	****	0.97	0.97
12	U-body 3D length (min)	A1	A1O3_A1	9	10	970	3.02	0.01	**	0.79	0.79
12	U-body 3D length (min)	A1O2	A1O2	10	10	779	3.04	0.02	***	0.85	0.86
12	U-body 3D length (min)	A1O3	A1O3	9	10	165	3.02	0.03	ns	0.77	0.78
12	U-body 3D length (min)	A1L1	A1L1	11	10	1685	2.75	0.01	****	0.00	0.00
12	U-body 3D length (min)	A1L3	A1L3	12	10	1726	2.89	0.01	***	0.41	0.43
12	U-body 3D length (min)	CT	A1BT_CT	13	6	1075	3.00	0.02		0.72	0.73
12	U-body 3D length (min)	A5	A1BT_A5	13	6	2249	3.08	0.01	****	0.96	0.96
12	U-body 3D length (min)	A1	A1BT_A1	13	6	683	3.09	0.02	****	1.00	1.00
12	U-body 3D length (min)	A1BT	A1BT	13	6	275	3.01	0.02	ns	0.75	0.76
13	Right limb angle asym. (min)	CT	A1O3_CT	9	10	1015	-47.03	0.52		0.40	
13	Right limb angle asym. (min)	A5	A1O3_A5	9	10	2748	-53.62	0.64	****	0.02	
13	Right limb angle asym. (min)	A1	A1O3_A1	9	10	952	-46.57	0.81	ns	0.42	
13	Right limb angle asym. (min)	A1O2	A1O2	10	10	779	-43.15	0.67	****	0.61	
13	Right limb angle asym. (min)	A1O3	A1O3	9	10	165	-47.36	1.63	ns	0.38	
13	Right limb angle asym. (min)	A1L1	A1L1	11	10	1685	-36.31	0.39	****	1.00	
13	Right limb angle asym. (min)	A1L3	A1L3	12	10	1726	-40.95	0.47	****	0.74	
13	Right limb angle asym. (min)	CT	A1BT_CT	13	6	1077	-45.92	0.52		0.46	
13	Right limb angle asym. (min)	A5	A1BT_A5	13	6	2195	-54.06	0.71	****	0.00	
13	Right limb angle asym. (min)	A1	A1BT_A1	13	6	674	-44.90	0.90	ns	0.52	
13	Right limb angle asym. (min)	A1BT	A1BT	13	6	275	-48.91	1.50	ns	0.29	
14	Hands 3D distance (max)	CT	A1O3_CT	9	10	1004	2.96	0.02		0.11	0.12
14	Hands 3D distance (max)	A5	A1O3_A5	9	10	2766	3.79	0.03	****	1.00	1.00
14	Hands 3D distance (max)	A1	A1O3_A1	9	10	952	3.32	0.04	****	0.49	0.53
14	Hands 3D distance (max)	A1O2	A1O2	10	10	779	3.04	0.04	ns	0.19	0.22
14	Hands 3D distance (max)	A1O3	A1O3	9	10	168	3.14	0.07	*	0.30	0.33
14	Hands 3D distance (max)	A1L1	A1L1	11	10	1685	3.02	0.01	ns	0.16	0.18
14	Hands 3D distance (max)	A1L3	A1L3	12	10	1726	2.89	0.02	**	0.02	0.03
14	Hands 3D distance (max)	CT	A1BT_CT	13	6	1062	2.87	0.02		0.00	0.00
14	Hands 3D distance (max)	A5	A1BT_A5	13	6	2217	3.67	0.03	****	0.87	0.89
14	Hands 3D distance (max)	A1	A1BT_A1	13	6	674	3.40	0.05	****	0.58	0.61
14	Hands 3D distance (max)	A1BT	A1BT	13	6	275	3.48	0.08	****	0.67	0.70
15	Left limb angle asym. (min)	CT	A1O3_CT	9	10	1013	-52.23	0.63		0.55	
15	Left limb angle asym. (min)	A5	A1O3_A5	9	10	2750	-67.02	0.71	****	0.00	
15	Left limb angle asym. (min)	A1	A1O3_A1	9	10	957	-56.25	0.95	***	0.40	
15	Left limb angle asym. (min)	A1O2	A1O2	10	10	779	-53.56	0.92	ns	0.50	
15	Left limb angle asym. (min)	A1O3	A1O3	9	10	165	-53.41	1.61	ns	0.51	
15	Left limb angle asym. (min)	A1L1	A1L1	11	10	1685	-46.96	0.52	****	0.75	
15	Left limb angle asym. (min)	A1L3	A1L3	12	10	1726	-40.26	0.37	****	1.00	
15	Left limb angle asym. (min)	CT	A1BT_CT	13	6	1067	-51.87	0.59		0.57	
15	Left limb angle asym. (min)	A5	A1BT_A5	13	6	2216	-63.59	0.77	****	0.13	
15	Left limb angle asym. (min)	A1	A1BT_A1	13	6	671	-55.69	1.11	**	0.42	
15	Left limb angle asym. (min)	A1BT	A1BT	13	6	275	-60.09	1.95	***	0.26	
16	Chest 1D velocity (max)	CT	A1O3_CT	9	10	1023	0.13	0.00		0.81	0.86
16	Chest 1D velocity (max)	A5	A1O3_A5	9	10	2798	0.08	0.00	****	0.09	0.12
16	Chest 1D velocity (max)	A1	A1O3_A1	9	10	967	0.08	0.00	****	0.15	0.20
16	Chest 1D velocity (max)	A1O2	A1O2	10	10	782	0.12	0.00	ns	0.72	0.78
16	Chest 1D velocity (max)	A1O3	A1O3	9	10	171	0.09	0.01	***	0.32	0.40
16	Chest 1D velocity (max)	A1L1	A1L1	11	10	1702	0.12	0.00	ns	0.68	0.75
16	Chest 1D velocity (max)	A1L3	A1L3	12	10	1738	0.14	0.00	**	1.00	1.00
16	Chest 1D velocity (max)	CT	A1BT_CT	13	6	1089	0.13	0.00		0.83	0.87
16	Chest 1D velocity (max)	A5	A1BT_A5	13	6	2227	0.07	0.00	****	0.00	0.00
16	Chest 1D velocity (max)	A1	A1BT_A1	13	6	690	0.08	0.00	****	0.09	0.13
16	Chest 1D velocity (max)	A1BT	A1BT	13	6	278	0.08	0.01	****	0.14	0.20
17	Leg angle asym. (std)	CT	A1O3_CT	9	10	1011	15.78	0.20		0.15	0.18
17	Leg angle asym. (std)	A5	A1O3_A5	9	10	2793	20.79	0.16	****	0.90	0.92
17	Leg angle asym. (std)	A1	A1O3_A1	9	10	951	18.49	0.24	****	0.56	0.60
17	Leg angle asym. (std)	A1O2	A1O2	10	10	779	16.68	0.27	**	0.29	0.33
17	Leg angle asym. (std)	A1O3	A1O3	9	10	168	17.72	0.48	***	0.44	0.49
17	Leg angle asym. (std)	A1L1	A1L1	11	10	1685	16.92	0.11	****	0.32	0.36
17	Leg angle asym. (std)	A1L3	A1L3	12	10	1726	14.77	0.11	****	0.00	0.00
17	Leg angle asym. (std)	CT	A1BT_CT	13	6	1071	15.24	0.21		0.07	0.08
17	Leg angle asym. (std)	A5	A1BT_A5	13	6	2196	21.45	0.17	****	1.00	1.00
17	Leg angle asym. (std)	A1	A1BT_A1	13	6	676	20.45	0.31	****	0.85	0.87
17	Leg angle asym. (std)	A1BT	A1BT	13	6	275	19.68	0.56	****	0.73	0.77
18	Feet 2D distance (mean)	CT	A1O3_CT	9	10	1012	2.65	0.01		0.06	0.07
18	Feet 2D distance (mean)	A5	A1O3_A5	9	10	2782	2.62	0.01	ns	0.00	0.00
18	Feet 2D distance (mean)	A1	A1O3_A1	9	10	951	2.89	0.01	****	0.59	0.61
18	Feet 2D distance (mean)	A1O2	A1O2	10	10	779	2.87	0.02	****	0.54	0.56
18	Feet 2D distance (mean)	A1O3	A1O3	9	10	164	2.89	0.03	****	0.59	0.61
18	Feet 2D distance (mean)	A1L1	A1L1	11	10	1685	2.99	0.01	****	0.81	0.82
18	Feet 2D distance (mean)	A1L3	A1L3	12	10	1726	2.95	0.01	****	0.71	0.73
18	Feet 2D distance (mean)	CT	A1BT_CT	13	6	1073	2.64	0.01		0.03	0.04
18	Feet 2D distance (mean)	A5	A1BT_A5	13	6	2196	2.65	0.01	ns	0.06	0.06
18	Feet 2D distance (mean)	A1	A1BT_A1	13	6	674	2.94	0.02	****	0.70	0.71
18	Feet 2D distance (mean)	A1BT	A1BT	13	6	275	3.08	0.03	****	1.00	1.00
19	Anus 1D velocity (std)	CT	A1O3_CT	9	10	1017	0.06	0.00		0.80	0.82
19	Anus 1D velocity (std)	A5	A1O3_A5	9	10	2790	0.05	0.00	****	0.31	0.34
19	Anus 1D velocity (std)	A1	A1O3_A1	9	10	958	0.05	0.00	**	0.45	0.48
19	Anus 1D velocity (std)	A1O2	A1O2	10	10	779	0.06	0.00	ns	1.00	1.00
19	Anus 1D velocity (std)	A1O3	A1O3	9	10	166	0.06	0.00	ns	0.68	0.71
19	Anus 1D velocity (std)	A1L1	A1L1	11	10	1685	0.06	0.00	ns	0.66	0.68
19	Anus 1D velocity (std)	A1L3	A1L3	12	10	1726	0.05	0.00	****	0.29	0.31
19	Anus 1D velocity (std)	CT	A1BT_CT	13	6	1073	0.05	0.00		0.53	0.55
19	Anus 1D velocity (std)	A5	A1BT_A5	13	6	2219	0.05	0.00	*	0.33	0.36
19	Anus 1D velocity (std)	A1	A1BT_A1	13	6	677	0.05	0.00	ns	0.37	0.40
19	Anus 1D velocity (std)	A1BT	A1BT	13	6	275	0.05	0.00	**	0.00	0.00
20	Arm angle asym. (mean)	CT	A1O3_CT	9	10	1000	-4.20	0.42		0.62	
20	Arm angle asym. (mean)	A5	A1O3_A5	9	10	2746	-10.01	0.43	****	0.00	
20	Arm angle asym. (mean)	A1	A1O3_A1	9	10	949	-9.43	0.57	****	0.06	
20	Arm angle asym. (mean)	A1O2	A1O2	10	10	779	-3.12	0.56	ns	0.74	
20	Arm angle asym. (mean)	A1O3	A1O3	9	10	166	-2.17	1.24	ns	0.84	
20	Arm angle asym. (mean)	A1L1	A1L1	11	10	1685	-0.68	0.23	****	1.00	
20	Arm angle asym. (mean)	A1L3	A1L3	12	10	1726	-5.97	0.27	***	0.43	
20	Arm angle asym. (mean)	CT	A1BT_CT	13	6	1072	-3.07	0.40		0.74	
20	Arm angle asym. (mean)	A5	A1BT_A5	13	6	2187	-8.55	0.46	****	0.16	
20	Arm angle asym. (mean)	A1	A1BT_A1	13	6	669	-4.54	0.67	ns	0.59	
20	Arm angle asym. (mean)	A1BT	A1BT	13	6	275	-1.49	1.12	ns	0.91	

Supplementary Table 16. Statistical summary of the KL divergences for the top 20 features identified by the TSFEL model comparing CT and PD (A5) groups. For each group, the KL divergence of each feature relative to its baseline value (0 wk) was computed across the experimental duration (0–10 wk). Each group consisted of 6 sampling sets, each containing 150 randomly selected clips. A two-way ANOVA (group × week) was performed for each feature, followed by weekly t-tests between groups (CT vs. A5). The resulting p-values from the weekly t-tests were corrected for multiple comparisons using the Bonferroni method. Source data are provided in the Source Data file (See S_Figure 36).

Rank	Feature name	group	Week	n	Mean	SEM	raw_p	corrected_p	significant	ANOVA_p_ Week	ANOVA_p_ Group	ANOVA_p_ Interaction
1	Foot distance_Signal distance	A5	0	6	0	0		1	ns	3.93E-16	6.16E-25	8.67E-14
1	Foot distance_Signal distance	CT	0	6	0	0		1	ns	3.93E-16	6.16E-25	8.67E-14
1	Foot distance_Signal distance	A5	2	6	0.17037522	0.02646104	0.000321265	0.001927588	**	3.93E-16	6.16E-25	8.67E-14
1	Foot distance_Signal distance	CT	2	6	0.023515	0.00720487	0.000321265	0.001927588	**	3.93E-16	6.16E-25	8.67E-14
1	Foot distance_Signal distance	A5	4	6	0.35026564	0.03390482	8.80242E-06	5.28145E-05	****	3.93E-16	6.16E-25	8.67E-14
1	Foot distance_Signal distance	CT	4	6	0.05185242	0.01236075	8.80242E-06	5.28145E-05	****	3.93E-16	6.16E-25	8.67E-14
1	Foot distance_Signal distance	A5	6	6	0.66230744	0.07718672	1.06087E-05	6.36519E-05	****	3.93E-16	6.16E-25	8.67E-14
1	Foot distance_Signal distance	CT	6	6	0.03341345	0.00876266	1.06087E-05	6.36519E-05	****	3.93E-16	6.16E-25	8.67E-14
1	Foot distance_Signal distance	A5	8	6	0.61365844	0.06685874	8.21868E-06	4.93121E-05	****	3.93E-16	6.16E-25	8.67E-14
1	Foot distance_Signal distance	CT	8	6	0.05446035	0.00570742	8.21868E-06	4.93121E-05	****	3.93E-16	6.16E-25	8.67E-14
1	Foot distance_Signal distance	A5	10	6	0.72959533	0.07149887	3.19215E-06	1.91529E-05	****	3.93E-16	6.16E-25	8.67E-14
1	Foot distance_Signal distance	CT	10	6	0.06219027	0.0089115	3.19215E-06	1.91529E-05	****	3.93E-16	6.16E-25	8.67E-14
2	Tail angle_Autocorrelation	A5	0	6	0	0		1	ns	3.45E-13	3.14E-10	4.79E-07
2	Tail angle_Autocorrelation	CT	0	6	0	0		1	ns	3.45E-13	3.14E-10	4.79E-07
2	Tail angle_Autocorrelation	A5	2	6	0.09561193	0.01742607	0.009775118	0.058650709	ns	3.45E-13	3.14E-10	4.79E-07
2	Tail angle_Autocorrelation	CT	2	6	0.03269221	0.00933625	0.009775118	0.058650709	ns	3.45E-13	3.14E-10	4.79E-07
2	Tail angle_Autocorrelation	A5	4	6	0.09137207	0.01363908	0.432993649	1	ns	3.45E-13	3.14E-10	4.79E-07
2	Tail angle_Autocorrelation	CT	4	6	0.06762204	0.02567395	0.432993649	1	ns	3.45E-13	3.14E-10	4.79E-07
2	Tail angle_Autocorrelation	A5	6	6	0.11437181	0.0244785	0.010348152	0.062088912	ns	3.45E-13	3.14E-10	4.79E-07
2	Tail angle_Autocorrelation	CT	6	6	0.03310391	0.00817154	0.010348152	0.062088912	ns	3.45E-13	3.14E-10	4.79E-07
2	Tail angle_Autocorrelation	A5	8	6	0.28049632	0.04531416	0.003065643	0.018393855	*	3.45E-13	3.14E-10	4.79E-07
2	Tail angle_Autocorrelation	CT	8	6	0.1034635	0.00546772	0.003065643	0.018393855	*	3.45E-13	3.14E-10	4.79E-07
2	Tail angle_Autocorrelation	A5	10	6	0.36977884	0.05547362	0.000411656	0.002469938	**	3.45E-13	3.14E-10	4.79E-07
2	Tail angle_Autocorrelation	CT	10	6	0.07717747	0.01051198	0.000411656	0.002469938	**	3.45E-13	3.14E-10	4.79E-07
3	Neck acc._Wavelet std_4	A5	0	6	0	0		1	ns	1.55E-27	2.62E-32	1.07E-25
3	Neck acc._Wavelet std_4	CT	0	6	0	0		1	ns	1.55E-27	2.62E-32	1.07E-25
3	Neck acc._Wavelet std_4	A5	2	6	0.07421688	0.00969439	0.005100132	0.030600789	*	1.55E-27	2.62E-32	1.07E-25
3	Neck acc._Wavelet std_4	CT	2	6	0.03404681	0.0057154	0.005100132	0.030600789	*	1.55E-27	2.62E-32	1.07E-25
3	Neck acc._Wavelet std_4	A5	4	6	0.15359882	0.01805963	2.76E-05	1.66E-04	***	1.55E-27	2.62E-32	1.07E-25
3	Neck acc._Wavelet std_4	CT	4	6	0.02177305	0.00213357	2.76E-05	1.66E-04	***	1.55E-27	2.62E-32	1.07E-25
3	Neck acc._Wavelet std_4	A5	6	6	0.55646892	0.05201572	4.24E-06	2.55E-05	****	1.55E-27	2.62E-32	1.07E-25
3	Neck acc._Wavelet std_4	CT	6	6	0.06793186	0.01604118	4.24E-06	2.55E-05	****	1.55E-27	2.62E-32	1.07E-25
3	Neck acc._Wavelet std_4	A5	8	6	0.66091673	0.03725616	1.17256E-08	7.03534E-08	****	1.55E-27	2.62E-32	1.07E-25
3	Neck acc._Wavelet std_4	CT	8	6	0.02483187	0.0067696	1.17256E-08	7.03534E-08	****	1.55E-27	2.62E-32	1.07E-25
3	Neck acc._Wavelet std_4	A5	10	6	0.73545469	0.04466466	3.27E-08	1.96E-07	****	1.55E-27	2.62E-32	1.07E-25
3	Neck acc._Wavelet std_4	CT	10	6	0.04051667	0.01103669	3.27E-08	1.96E-07	****	1.55E-27	2.62E-32	1.07E-25
4	Body angle velocity_ECDF percentile 0	A5	0	6	0	0		1	ns	3.75E-09	3.73E-06	1.66E-03
4	Body angle velocity_ECDF percentile 0	CT	0	6	0	0		1	ns	3.75E-09	3.73E-06	1.66E-03
4	Body angle velocity_ECDF percentile 0	A5	2	6	0.02404809	0.00528348	0.774706661	1	ns	3.75E-09	3.73E-06	1.66E-03
4	Body angle velocity_ECDF percentile 0	CT	2	6	0.02196881	0.00469833	0.774706661	1	ns	3.75E-09	3.73E-06	1.66E-03
4	Body angle velocity_ECDF percentile 0	A5	4	6	0.05367335	0.01466589	0.21998823	1	ns	3.75E-09	3.73E-06	1.66E-03
4	Body angle velocity_ECDF percentile 0	CT	4	6	0.03258461	0.00668379	0.21998823	1	ns	3.75E-09	3.73E-06	1.66E-03
4	Body angle velocity_ECDF percentile 0	A5	6	6	0.12555558	0.0175272	1.11E-02	6.66E-02	ns	3.75E-09	3.73E-06	1.66E-03
4	Body angle velocity_ECDF percentile 0	CT	6	6	0.05600679	0.01391638	1.11E-02	6.66E-02	ns	3.75E-09	3.73E-06	1.66E-03
4	Body angle velocity_ECDF percentile 0	A5	8	6	0.18448129	0.03012059	8.87E-03	0.053193546	ns	3.75E-09	3.73E-06	1.66E-03
4	Body angle velocity_ECDF percentile 0	CT	8	6	0.05883925	0.02441882	8.87E-03	0.053193546	ns	3.75E-09	3.73E-06	1.66E-03
4	Body angle velocity_ECDF percentile 0	A5	10	6	0.17332734	0.04242629	2.62E-02	1.57E-01	ns	3.75E-09	3.73E-06	1.66E-03
4	Body angle velocity_ECDF percentile 0	CT	10	6	0.05612191	0.01493762	2.62E-02	1.57E-01	ns	3.75E-09	3.73E-06	1.66E-03
5	Body angle_min	A5	0	6	0	0		1	ns	7.38E-18	1.39E-14	1.04E-07
5	Body angle_min	CT	0	6	0	0		1	ns	7.38E-18	1.39E-14	1.04E-07
5	Body angle_min	A5	2	6	0.06455932	0.00910821	0.021637462	0.129824771	ns	7.38E-18	1.39E-14	1.04E-07
5	Body angle_min	CT	2	6	0.03237961	0.00756454	0.021637462	0.129824771	ns	7.38E-18	1.39E-14	1.04E-07
5	Body angle_min	A5	4	6	0.06967051	0.00709253	0.014873619	0.089241714	ns	7.38E-18	1.39E-14	1.04E-07
5	Body angle_min	CT	4	6	0.03530771	0.00930748	0.014873619	0.089241714	ns	7.38E-18	1.39E-14	1.04E-07
5	Body angle_min	A5	6	6	0.20237378	0.01446254	3.41E-05	2.05E-04	***	7.38E-18	1.39E-14	1.04E-07
5	Body angle_min	CT	6	6	0.06577093	0.01281053	3.41E-05	2.05E-04	***	7.38E-18	1.39E-14	1.04E-07
5	Body angle_min	A5	8	6	0.20804828	0.01872075	8.44E-05	5.06E-04	***	7.38E-18	1.39E-14	1.04E-07
5	Body angle_min	CT	8	6	0.0622105	0.01335448	8.44E-05	5.06E-04	***	7.38E-18	1.39E-14	1.04E-07
5	Body angle_min	A5	10	6	0.17717344	0.01876867	5.35E-03	3.21E-02	*	7.38E-18	1.39E-14	1.04E-07
5	Body angle_min	CT	10	6	0.07804112	0.02077591	5.35E-03	3.21E-02	*	7.38E-18	1.39E-14	1.04E-07
6	Left limb angle asym._Histogram 1	A5	0	6	0	0		1	ns	2.31E-07	1.03E-10	5.67E-06
6	Left limb angle asym._Histogram 1	CT	0	6	0	0		1	ns	2.31E-07	1.03E-10	5.67E-06
6	Left limb angle asym._Histogram 1	A5	2	6	0.03731306	0.01206259	0.599282662	1	ns	2.31E-07	1.03E-10	5.67E-06
6	Left limb angle asym._Histogram 1	CT	2	6	0.0299387	0.00626075	0.599282662	1	ns	2.31E-07	1.03E-10	5.67E-06
6	Left limb angle asym._Histogram 1	A5	4	6	0.15195249	0.02211651	0.001464505	0.008787029	**	2.31E-07	1.03E-10	5.67E-06
6	Left limb angle asym._Histogram 1	CT	4	6	0.0419046	0.0123927	0.001464505	0.008787029	**	2.31E-07	1.03E-10	5.67E-06
6	Left limb angle asym._Histogram 1	A5	6	6	0.21067546	0.02563964	2.67443E-05	0.000160466	***	2.31E-07	1.03E-10	5.67E-06
6	Left limb angle asym._Histogram 1	CT	6	6	0.02188173	0.00397691	2.67443E-05	0.000160466	***	2.31E-07	1.03E-10	5.67E-06
6	Left limb angle asym._Histogram 1	A5	8	6	0.35667667	0.09140708	0.006636711	0.039820266	*	2.31E-07	1.03E-10	5.67E-06
6	Left limb angle asym._Histogram 1	CT	8	6	0.04425295	0.00542579	0.006636711	0.039820266	*	2.31E-07	1.03E-10	5.67E-06
6	Left limb angle asym._Histogram 1	A5	10	6	0.32982536	0.063176	0.000755069	0.004530413	**	2.31E-07	1.03E-10	5.67E-06
6	Left limb angle asym._Histogram 1	CT	10	6	0.02782555	0.00378502	0.000755069	0.004530413	**	2.31E-07	1.03E-10	5.67E-06
7	Right limb angle asym._Histogram 1	A5	0	6	0	0		1	ns	2.98E-08	1.29E-09	3.71E-06
7	Right limb angle asym._Histogram 1	CT	0	6	0	0		1	ns	2.98E-08	1.29E-09	3.71E-06
7	Right limb angle asym._Histogram 1	A5	2	6	0.04192758	0.02786576	0.692717259	1	ns	2.98E-08	1.29E-09	3.71E-06
7	Right limb angle asym._Histogram 1	CT	2	6	0.03029413	0.00642412	0.692717259	1	ns	2.98E-08	1.29E-09	3.71E-06
7	Right limb angle asym._Histogram 1	A5	4	6	0.08082089	0.02295092	0.161227658	0.967365951	ns	2.98E-08	1.29E-09	3.71E-06
7	Right limb angle asym._Histogram 1	CT	4	6	0.04439967	0.00726229	0.161227658	0.967365951	ns	2.98E-08	1.29E-09	3.71E-06
7	Right limb angle asym._Histogram 1	A5	6	6	0.16131958	0.01154759	5.02719E-05	0.000301631	***	2.98E-08	1.29E-09	3.71E-06
7	Right limb angle asym._Histogram 1	CT	6	6	0.03665988	0.01440419	5.02719E-05	0.000301631	***	2.98E-08	1.29E-09	3.71E-06
7	Right limb angle asym._Histogram 1	A5	8	6	0.24878075	0.04536917	0.001502661	0.009015968	**	2.98E-08	1.29E-09	3.71E-06
7	Right limb angle asym._Histogram 1	CT	8	6	0.05004002	0.00733203	0.001502661	0.009015968	**	2.98E-08	1.29E-09	3.71E-06
7	Right limb angle asym._Histogram 1	A5	10	6	0.27082717	0.05774028	0.002139157	0.01283494	*	2.98E-08	1.29E-09	3.71E-06
7	Right limb angle asym._Histogram 1	CT	10	6	0.0304976	0.0099679	0.002139157	0.01283494	*	2.98E-08	1.29E-09	3.71E-06
8	Left limb angle asym._Histogram 2	A5	0	6	0	0		1	ns	1.99E-07	1.44E-10	7.91E-06
8	Left limb angle asym._Histogram 2	CT	0	6	0	0		1	ns	1.99E-07	1.44E-10	7.91E-06
8	Left limb angle asym._Histogram 2	A5	2	6	0.02358795	0.00963979	0.963278364	1	ns	1.99E-07	1.44E-10	7.91E-06
8	Left limb angle asym._Histogram 2	CT	2	6	0.02306394	0.00550418	0.963278364	1	ns	1.99E-07	1.44E-10	7.91E-06
8	Left limb angle asym._Histogram 2	A5	4	6	0.09900293	0.02202031	0.017207834	0.103247004	ns	1.99E-07	1.44E-10	7.91E-06
8	Left limb angle asym._Histogram 2	CT	4	6	0.03116195	0.00900894	0.017207834	0.103247004	ns	1.99E-07	1.44E-10	7.91E-06
8	Left limb angle asym._Histogram 2	A5	6	6	0.18666478	0.03640445	0.001657289	0.009943733	**	1.99E-07	1.44E-10	7.91E-06
8	Left limb angle asym._Histogram 2	CT	6	6	0.02907983	0.00645753	0.001657289	0.009943733	**	1.99E-07	1.44E-10	7.91E-06
8	Left limb angle asym._Histogram 2	A5	8	6	0.23598235	0.05225041	0.003176701	0.019060207	*	1.99E-07	1.44E-10	7.91E-06
8	Left limb angle asym._Histogram 2	CT	8	6	0.02918754	0.01203271	0.003176701	0.019060207	*	1.99E-07	1.44E-10	7.91E-06
8	Left limb angle asym._Histogram 2	A5	10	6	0.19336265	0.03426395	5.62E-04	3.37E-03	**	1.99E-07	1.44E-10	7.91E-06
8	Left limb angle asym._Histogram 2	CT	10	6	0.02086569	0.00555713	5.62E-04	3.37E-03	**	1.99E-07	1.44E-10	7.91E-06
9	Neck velocity_FFT mean coeff. 1	A5	0	6	0	0		1	ns	2.90E-09	5.01E-04	3.04E-04
9	Neck velocity_FFT mean coeff. 1	CT	0	6	0	0		1	ns	2.90E-09	5.01E-04	3.04E-04
9	Neck velocity_FFT mean coeff. 1	A5	2	6	0.05668572	0.01064155	0.104364306	0.626185837	ns	2.90E-09	5.01E-04	3.04E-04
9	Neck velocity_FFT mean coeff. 1	CT	2	6	0.03455398	0.00634614	0.104364306	0.626185837	ns	2.90E-09	5.01E-04	3.04E-04
9	Neck velocity_FFT mean coeff. 1	A5	4	6	0.04974213	0.00878447	0.107401426	0.644408558	ns	2.90E-09	5.01E-04	3.04E-04
9	Neck velocity_FFT mean coeff. 1	CT	4	6	0.03218789	0.00462082	0.107401426	0.644408558	ns	2.90E-09	5.01E-04	3.04E-04
9	Neck velocity_FFT mean coeff. 1	A5	6	6	0.03549342	0.00488556	8.76E-01	1.00E+00	ns	2.90E-09	5.01E-04	3.04E-04
9	Neck velocity_FFT mean coeff. 1	CT	6	6	0.03438962	0.00489782	8.76E-01	1.00E+00	ns	2.90E-09	5.01E-04	3.04E-04
9	Neck velocity_FFT mean coeff. 1	A5	8	6	0.0269185	0.00505477	3.95E-01	1	ns	2.90E-09	5.01E-04	3.04E-04
9	Neck velocity_FFT mean coeff. 1	CT	8	6	0.03496651	0.00752219	3.95E-01	1	ns	2.90E-09	5.01E-04	3.04E-04
9	Neck velocity_FFT mean coeff. 1	A5	10	6	0.0809141	0.01338732	0.002488797	0.014932784	*	2.90E-09	5.01E-04	3.04E-04
9	Neck velocity_FFT mean coeff. 1	CT	10	6	0.02558123	0.00338222	0.002488797	0.014932784	*	2.90E-09	5.01E-04	3.04E-04
10	Body length_FFT mean coeff. 1	A5	0	6	0	0		1	ns	2.10E-16	1.67E-17	6.64E-14
10	Body length_FFT mean coeff. 1	CT	0	6	0	0		1	ns	2.10E-16	1.67E-17	6.64E-14
10	Body length_FFT mean coeff. 1	A5	2	6	0.02470774	0.00441722	0.461265352	1	ns	2.10E-16	1.67E-17	6.64E-14
10	Body length_FFT mean coeff. 1	CT	2	6	0.03360955	0.01074579	0.461265352	1	ns	2.10E-16	1.67E-17	6.64E-14
10	Body length_FFT mean coeff. 1	A5	4	6	0.10488117	0.01712052	0.001974196	0.011845174	*	2.10E-16	1.67E-17	6.64E-14
10	Body length_FFT mean coeff. 1	CT	4	6	0.02894534	0.00643419	0.001974196	0.011845174	*	2.10E-16	1.67E-17	6.64E-14
10	Body length_FFT mean coeff. 1	A5	6	6	0.26944426	0.05348753	2.86E-03	1.72E-02	*	2.10E-16	1.67E-17	6.64E-14
10	Body length_FFT mean coeff. 1	CT	6	6	0.05400598	0.01258049	2.86E-03	1.72E-02	*	2.10E-16	1.67E-17	6.64E-14
10	Body length_FFT mean coeff. 1	A5	8	6	0.33301638	0.03190248	3.77174E-06	2.26305E-05	****	2.10E-16	1.67E-17	6.64E-14
10	Body length_FFT mean coeff. 1	CT	8	6	0.03737335	0.00626943	3.77174E-06	2.26305E-05	****	2.10E-16	1.67E-17	6.64E-14
10	Body length_FFT mean coeff. 1	A5	10	6	0.4743452	0.04916658	7.09E-06	4.25E-05	****	2.10E-16	1.67E-17	6.64E-14
10	Body length_FFT mean coeff. 1	CT	10	6	0.04496989	0.01226364	7.09E-06	4.25E-05	****	2.10E-16	1.67E-17	6.64E-14
11	U-body length_Negative turning points	A5	0	6	0	0		1	ns	8.04E-23	5.33E-27	1.26E-21
11	U-body length_Negative turning points	CT	0	6	0	0		1	ns	8.04E-23	5.33E-27	1.26E-21
11	U-body length_Negative turning points	A5	2	6	0.039059	0.007846	0.521097	1	ns	8.04E-23	5.33E-27	1.26E-21
11	U-body length_Negative turning points	CT	2	6	0.033095	0.004346	0.521097	1	ns	8.04E-23	5.33E-27	1.26E-21
11	U-body length_Negative turning points	A5	4	6	0.101707	0.019455	0.019627	0.117765	ns	8.04E-23	5.33E-27	1.26E-21
11	U-body length_Negative turning points	CT	4	6	0.036312	0.013302	0.019627	0.117765	ns	8.04E-23	5.33E-27	1.26E-21
11	U-body length_Negative turning points	A5	6	6	0.285586	0.028675	7.52E-06	4.51E-05	****	8.04E-23	5.33E-27	1.26E-21
11	U-body length_Negative turning points	CT	6	6	0.032165	0.009179	7.52E-06	4.51E-05	****	8.04E-23	5.33E-27	1.26E-21
11	U-body length_Negative turning points	A5	8	6	0.41087	0.029797	1.75E-07	1.05E-06	****	8.04E-23	5.33E-27	1.26E-21
11	U-body length_Negative turning points	CT	8	6	0.028951	0.004569	1.75E-07	1.05E-06	****	8.04E-23	5.33E-27	1.26E-21
11	U-body length_Negative turning points	A5	10	6	0.321488	0.018589	2.24E-08	1.35E-07	****	8.04E-23	5.33E-27	1.26E-21
11	U-body length_Negative turning points	CT	10	6	0.024104	0.00359	2.24E-08	1.35E-07	****	8.04E-23	5.33E-27	1.26E-21
12	Hand distance_Median diff	A5	0	6	0	0		1	ns	1.26E-15	1.84E-20	7.46E-11
12	Hand distance_Median diff	CT	0	6	0	0		1	ns	1.26E-15	1.84E-20	7.46E-11
12	Hand distance_Median diff	A5	2	6	0.070775	0.010509	0.018412	0.11047	ns	1.26E-15	1.84E-20	7.46E-11
12	Hand distance_Median diff	CT	2	6	0.038999	0.004153	0.018412	0.11047	ns	1.26E-15	1.84E-20	7.46E-11
12	Hand distance_Median diff	A5	4	6	0.130757	0.016764	0.000858	0.005146	**	1.26E-15	1.84E-20	7.46E-11
12	Hand distance_Median diff	CT	4	6	0.045904	0.006829	0.000858	0.005146	**	1.26E-15	1.84E-20	7.46E-11
12	Hand distance_Median diff	A5	6	6	0.141016	0.012268	5.24E-05	0.000314	***	1.26E-15	1.84E-20	7.46E-11
12	Hand distance_Median diff	CT	6	6	0.038422	0.009091	5.24E-05	0.000314	***	1.26E-15	1.84E-20	7.46E-11
12	Hand distance_Median diff	A5	8	6	0.192528	0.018743	1.01E-05	6.08E-05	****	1.26E-15	1.84E-20	7.46E-11
12	Hand distance_Median diff	CT	8	6	0.033143	0.005683	1.01E-05	6.08E-05	****	1.26E-15	1.84E-20	7.46E-11
12	Hand distance_Median diff	A5	10	6	0.213521	0.020493	1.52E-05	9.12E-05	****	1.26E-15	1.84E-20	7.46E-11
12	Hand distance_Median diff	CT	10	6	0.038855	0.009243	1.52E-05	9.12E-05	****	1.26E-15	1.84E-20	7.46E-11
13	Leg angle asym._MFCC 3	A5	0	6	0	0		1	ns	1.18E-08	4.87E-07	7.75E-04
13	Leg angle asym._MFCC 3	CT	0	6	0	0		1	ns	1.18E-08	4.87E-07	7.75E-04
13	Leg angle asym._MFCC 3	A5	2	6	0.019574	0.002092	0.442945	1	ns	1.18E-08	4.87E-07	7.75E-04
13	Leg angle asym._MFCC 3	CT	2	6	0.023804	0.004865	0.442945	1	ns	1.18E-08	4.87E-07	7.75E-04
13	Leg angle asym._MFCC 3	A5	4	6	0.070528	0.02153	0.139448	0.836691	ns	1.18E-08	4.87E-07	7.75E-04
13	Leg angle asym._MFCC 3	CT	4	6	0.035059	0.004948	0.139448	0.836691	ns	1.18E-08	4.87E-07	7.75E-04
13	Leg angle asym._MFCC 3	A5	6	6	0.14577	0.024179	0.004812	0.028874	*	1.18E-08	4.87E-07	7.75E-04
13	Leg angle asym._MFCC 3	CT	6	6	0.052653	0.009099	0.004812	0.028874	*	1.18E-08	4.87E-07	7.75E-04
13	Leg angle asym._MFCC 3	A5	8	6	0.15398	0.031758	0.024671	0.148029	ns	1.18E-08	4.87E-07	7.75E-04
13	Leg angle asym._MFCC 3	CT	8	6	0.057834	0.017784	0.024671	0.148029	ns	1.18E-08	4.87E-07	7.75E-04
13	Leg angle asym._MFCC 3	A5	10	6	0.173517	0.037206	5.47E-03	3.28E-02	*	1.18E-08	4.87E-07	7.75E-04
13	Leg angle asym._MFCC 3	CT	10	6	0.039657	0.007463	5.47E-03	3.28E-02	*	1.18E-08	4.87E-07	7.75E-04
14	Chest acc._Wavlet entropy	A5	0	6	0	0		1	ns	2.28E-29	1.12E-31	2.66E-29
14	Chest acc._Wavlet entropy	CT	0	6	0	0		1	ns	2.28E-29	1.12E-31	2.66E-29
14	Chest acc._Wavlet entropy	A5	2	6	0.054689	0.008715	0.87588	1	ns	2.28E-29	1.12E-31	2.66E-29
14	Chest acc._Wavlet entropy	CT	2	6	0.051435	0.018336	0.87588	1	ns	2.28E-29	1.12E-31	2.66E-29
14	Chest acc._Wavlet entropy	A5	4	6	0.104999	0.026778	0.076186	0.457115	ns	2.28E-29	1.12E-31	2.66E-29
14	Chest acc._Wavlet entropy	CT	4	6	0.043263	0.016049	0.076186	0.457115	ns	2.28E-29	1.12E-31	2.66E-29
14	Chest acc._Wavlet entropy	A5	6	6	0.405187	0.032781	1.31E-06	7.85E-06	****	2.28E-29	1.12E-31	2.66E-29
14	Chest acc._Wavlet entropy	CT	6	6	0.037651	0.014838	1.31E-06	7.85E-06	****	2.28E-29	1.12E-31	2.66E-29
14	Chest acc._Wavlet entropy	A5	8	6	0.379141	0.021217	1.22E-08	7.33E-08	****	2.28E-29	1.12E-31	2.66E-29
14	Chest acc._Wavlet entropy	CT	8	6	0.02024	0.003189	1.22E-08	7.33E-08	****	2.28E-29	1.12E-31	2.66E-29
14	Chest acc._Wavlet entropy	A5	10	6	0.740841	0.032101	8.57E-10	5.14E-09	****	2.28E-29	1.12E-31	2.66E-29
14	Chest acc._Wavlet entropy	CT	10	6	0.028569	0.00459	8.57E-10	5.14E-09	****	2.28E-29	1.12E-31	2.66E-29
15	Right limb angle asym._Histogram 2	A5	0	6	0	0		1	ns	9.58E-12	6.49E-12	1.55E-09
15	Right limb angle asym._Histogram 2	CT	0	6	0	0		1	ns	9.58E-12	6.49E-12	1.55E-09
15	Right limb angle asym._Histogram 2	A5	2	6	0.015403	0.001878	0.076061	0.456364	ns	9.58E-12	6.49E-12	1.55E-09
15	Right limb angle asym._Histogram 2	CT	2	6	0.026177	0.005111	0.076061	0.456364	ns	9.58E-12	6.49E-12	1.55E-09
15	Right limb angle asym._Histogram 2	A5	4	6	0.036049	0.005231	0.035506	0.213033	ns	9.58E-12	6.49E-12	1.55E-09
15	Right limb angle asym._Histogram 2	CT	4	6	0.022162	0.002306	0.035506	0.213033	ns	9.58E-12	6.49E-12	1.55E-09
15	Right limb angle asym._Histogram 2	A5	6	6	0.17861	0.027466	1.07E-03	6.39E-03	**	9.58E-12	6.49E-12	1.55E-09
15	Right limb angle asym._Histogram 2	CT	6	6	0.030687	0.017452	1.07E-03	6.39E-03	**	9.58E-12	6.49E-12	1.55E-09
15	Right limb angle asym._Histogram 2	A5	8	6	0.18814	0.028467	3.87E-04	2.32E-03	**	9.58E-12	6.49E-12	1.55E-09
15	Right limb angle asym._Histogram 2	CT	8	6	0.032858	0.008538	3.87E-04	2.32E-03	**	9.58E-12	6.49E-12	1.55E-09
15	Right limb angle asym._Histogram 2	A5	10	6	0.235295	0.040348	4.41E-04	2.65E-03	**	9.58E-12	6.49E-12	1.55E-09
15	Right limb angle asym._Histogram 2	CT	10	6	0.025884	0.00592	4.41E-04	2.65E-03	**	9.58E-12	6.49E-12	1.55E-09
16	Chest 3D velocity_LPCC 9	A5	0	6	0	0		1	ns	1.74E-15	1.07E-21	1.84E-13
16	Chest 3D velocity_LPCC 9	CT	0	6	0	0		1	ns	1.74E-15	1.07E-21	1.84E-13
16	Chest 3D velocity_LPCC 9	A5	2	6	0.059188	0.017796	0.108104	0.648626	ns	1.74E-15	1.07E-21	1.84E-13
16	Chest 3D velocity_LPCC 9	CT	2	6	0.025432	0.007019	0.108104	0.648626	ns	1.74E-15	1.07E-21	1.84E-13
16	Chest 3D velocity_LPCC 9	A5	4	6	0.055623	0.007038	0.000432	0.002594	**	1.74E-15	1.07E-21	1.84E-13
16	Chest 3D velocity_LPCC 9	CT	4	6	0.014538	0.003762	0.000432	0.002594	**	1.74E-15	1.07E-21	1.84E-13
16	Chest 3D velocity_LPCC 9	A5	6	6	0.129634	0.016514	4.05E-05	0.000243	***	1.74E-15	1.07E-21	1.84E-13
16	Chest 3D velocity_LPCC 9	CT	6	6	0.012828	0.003395	4.05E-05	0.000243	***	1.74E-15	1.07E-21	1.84E-13
16	Chest 3D velocity_LPCC 9	A5	8	6	0.195161	0.014186	3.20E-07	1.92E-06	****	1.74E-15	1.07E-21	1.84E-13
16	Chest 3D velocity_LPCC 9	CT	8	6	0.018782	0.00437	3.20E-07	1.92E-06	****	1.74E-15	1.07E-21	1.84E-13
16	Chest 3D velocity_LPCC 9	A5	10	6	0.126311	0.009566	1.06E-06	6.35E-06	****	1.74E-15	1.07E-21	1.84E-13
16	Chest 3D velocity_LPCC 9	CT	10	6	0.01528	0.004621	1.06E-06	6.35E-06	****	1.74E-15	1.07E-21	1.84E-13
17	Arm angle asym._MFCC 0	A5	0	6	0	0		1	ns	1.05E-04	4.78E-04	1.29E-03
17	Arm angle asym._MFCC 0	CT	0	6	0	0		1	ns	1.05E-04	4.78E-04	1.29E-03
17	Arm angle asym._MFCC 0	A5	2	6	0.022273	0.006665	0.940663	1	ns	1.05E-04	4.78E-04	1.29E-03
17	Arm angle asym._MFCC 0	CT	2	6	0.021678	0.004037	0.940663	1	ns	1.05E-04	4.78E-04	1.29E-03
17	Arm angle asym._MFCC 0	A5	4	6	0.047456	0.014203	0.347056	1	ns	1.05E-04	4.78E-04	1.29E-03
17	Arm angle asym._MFCC 0	CT	4	6	0.03133	0.008085	0.347056	1	ns	1.05E-04	4.78E-04	1.29E-03
17	Arm angle asym._MFCC 0	A5	6	6	0.075729	0.014673	1.53E-03	9.16E-03	**	1.05E-04	4.78E-04	1.29E-03
17	Arm angle asym._MFCC 0	CT	6	6	0.012276	0.000999	1.53E-03	9.16E-03	**	1.05E-04	4.78E-04	1.29E-03
17	Arm angle asym._MFCC 0	A5	8	6	0.029162	0.007074	7.51E-01	1.00E+00	ns	1.05E-04	4.78E-04	1.29E-03
17	Arm angle asym._MFCC 0	CT	8	6	0.034025	0.013122	7.51E-01	1.00E+00	ns	1.05E-04	4.78E-04	1.29E-03
17	Arm angle asym._MFCC 0	A5	10	6	0.050053	0.013172	1.71E-02	1.03E-01	ns	1.05E-04	4.78E-04	1.29E-03
17	Arm angle asym._MFCC 0	CT	10	6	0.011615	0.002818	1.71E-02	1.03E-01	ns	1.05E-04	4.78E-04	1.29E-03
18	H-body length_Skewness	A5	0	6	0	0		1	ns	1.56E-07	0.038306	1.58E-01
18	H-body length_Skewness	CT	0	6	0	0		1	ns	1.56E-07	0.038306	1.58E-01
18	H-body length_Skewness	A5	2	6	0.039736	0.006958	0.063265	0.379591	ns	1.56E-07	0.038306	1.58E-01
18	H-body length_Skewness	CT	2	6	0.023625	0.00333	0.063265	0.379591	ns	1.56E-07	0.038306	1.58E-01
18	H-body length_Skewness	A5	4	6	0.053795	0.007698	0.065813	0.394878	ns	1.56E-07	0.038306	1.58E-01
18	H-body length_Skewness	CT	4	6	0.033625	0.006011	0.065813	0.394878	ns	1.56E-07	0.038306	1.58E-01
18	H-body length_Skewness	A5	6	6	0.020339	0.004223	0.380354	1	ns	1.56E-07	0.038306	1.58E-01
18	H-body length_Skewness	CT	6	6	0.025027	0.002875	0.380354	1	ns	1.56E-07	0.038306	1.58E-01
18	H-body length_Skewness	A5	8	6	0.031774	0.005491	0.677133	1	ns	1.56E-07	0.038306	1.58E-01
18	H-body length_Skewness	CT	8	6	0.035276	0.006045	0.677133	1	ns	1.56E-07	0.038306	1.58E-01
18	H-body length_Skewness	A5	10	6	0.070068	0.020108	0.20906	1	ns	1.56E-07	0.038306	1.58E-01
18	H-body length_Skewness	CT	10	6	0.03939	0.01085	0.20906	1	ns	1.56E-07	0.038306	1.58E-01
19	Hand distance_Histogram 5	A5	0	6	0	0		1	ns	1.01E-02	0.000816	0.226766
19	Hand distance_Histogram 5	CT	0	6	0	0		1	ns	1.01E-02	0.000816	0.226766
19	Hand distance_Histogram 5	A5	2	6	0.165501	0.040488	0.038404	0.230424	ns	1.01E-02	0.000816	0.226766
19	Hand distance_Histogram 5	CT	2	6	0.060799	0.017056	0.038404	0.230424	ns	1.01E-02	0.000816	0.226766
19	Hand distance_Histogram 5	A5	4	6	0.132035	0.045357	0.058749	0.352494	ns	1.01E-02	0.000816	0.226766
19	Hand distance_Histogram 5	CT	4	6	0.034883	0.004221	0.058749	0.352494	ns	1.01E-02	0.000816	0.226766
19	Hand distance_Histogram 5	A5	6	6	0.119739	0.058809	0.970284	1	ns	1.01E-02	0.000816	0.226766
19	Hand distance_Histogram 5	CT	6	6	0.122269	0.030473	0.970284	1	ns	1.01E-02	0.000816	0.226766
19	Hand distance_Histogram 5	A5	8	6	0.22416	0.059609	0.028396	0.170378	ns	1.01E-02	0.000816	0.226766
19	Hand distance_Histogram 5	CT	8	6	0.065449	0.01708	0.028396	0.170378	ns	1.01E-02	0.000816	0.226766
19	Hand distance_Histogram 5	A5	10	6	0.192487	0.084701	0.146375	0.878248	ns	1.01E-02	0.000816	0.226766
19	Hand distance_Histogram 5	CT	10	6	0.058493	0.008075	0.146375	0.878248	ns	1.01E-02	0.000816	0.226766
20	Chest velocity_LPCC 5	A5	0	6	0	0		1	ns	6.26E-11	1.19E-16	5.33E-09
20	Chest velocity_LPCC 5	CT	0	6	0	0		1	ns	6.26E-11	1.19E-16	5.33E-09
20	Chest velocity_LPCC 5	A5	2	6	0.052329	0.012647	0.089924	0.539546	ns	6.26E-11	1.19E-16	5.33E-09
20	Chest velocity_LPCC 5	CT	2	6	0.024124	0.008111	0.089924	0.539546	ns	6.26E-11	1.19E-16	5.33E-09
20	Chest velocity_LPCC 5	A5	4	6	0.092321	0.01086	0.000896	0.005373	**	6.26E-11	1.19E-16	5.33E-09
20	Chest velocity_LPCC 5	CT	4	6	0.032215	0.006964	0.000896	0.005373	**	6.26E-11	1.19E-16	5.33E-09
20	Chest velocity_LPCC 5	A5	6	6	0.210752	0.013355	2.04E-07	1.22E-06	****	6.26E-11	1.19E-16	5.33E-09
20	Chest velocity_LPCC 5	CT	6	6	0.021175	0.007272	2.04E-07	1.22E-06	****	6.26E-11	1.19E-16	5.33E-09
20	Chest velocity_LPCC 5	A5	8	6	0.171336	0.028653	5.65E-04	3.39E-03	**	6.26E-11	1.19E-16	5.33E-09
20	Chest velocity_LPCC 5	CT	8	6	0.02469	0.007139	5.65E-04	3.39E-03	**	6.26E-11	1.19E-16	5.33E-09
20	Chest velocity_LPCC 5	A5	10	6	0.163518	0.03107	1.06E-03	6.35E-03	**	6.26E-11	1.19E-16	5.33E-09
20	Chest velocity_LPCC 5	CT	10	6	0.021435	0.003092	1.06E-03	6.35E-03	**	6.26E-11	1.19E-16	5.33E-09

Supplementary Table 17. Statistical summary of the feature values for the top 20 features identified by the TSFEL model, comparing the CT and PD (A5) groups. For each feature, the values at each week were divided by the group's corresponding baseline (0 wk) mean value, and the normalised data were analysed over the experimental duration (0–10 wk). Each group consisted of 6 sampling sets, each containing 150 randomly selected clips. Two-way ANOVA (group × week) was performed for each feature on the normalised data, followed by weekly t-tests comparing the CT and A5 groups. P-values from weekly t-tests were corrected for multiple comparisons using the Bonferroni method. Source data are provided in the Source Data file (See S_Figure 37).

Rank	Feature name	Week	group	n	Mean	SEM	raw_p	corrected_p	significant	ANOVA_p_Week	ANOVA_p_Group	ANOVA_p_Interaction
1	Foot distance_Signal distance	0	CT	746	1	0.00029934	1	1	ns	1.00E+00	1.00E+00	0.884164
1	Foot distance_Signal distance	2	CT	622	1.00005993	0.00032513	5.93018E-21	3.55811E-20	****	1.00E+00	1.00E+00	0.884164
1	Foot distance_Signal distance	4	CT	489	1.00162711	0.00035107	1.87823E-27	1.12694E-26	****	1.00E+00	1.00E+00	0.884164
1	Foot distance_Signal distance	6	CT	444	1.00001093	0.0003976	9.00327E-79	5.40196E-78	****	1.00E+00	1.00E+00	0.884164
1	Foot distance_Signal distance	8	CT	443	1.00223356	0.00044519	1.00425E-58	6.02551E-58	****	1.00E+00	1.00E+00	0.884164
1	Foot distance_Signal distance	10	CT	414	1.00226097	0.00042607	4.25965E-64	2.55579E-63	****	1.00E+00	1.00E+00	0.884164
1	Foot distance_Signal distance	0	A5	927	1	0.00038159	1	1	ns	1.00E+00	1.00E+00	0.884164
1	Foot distance_Signal distance	2	A5	962	1.00587274	0.00044336	5.93018E-21	3.55811E-20	****	1.00E+00	1.00E+00	0.884164
1	Foot distance_Signal distance	4	A5	797	1.00918904	0.00048756	1.87823E-27	1.12694E-26	****	1.00E+00	1.00E+00	0.884164
1	Foot distance_Signal distance	6	A5	1000	1.01590043	0.00049886	9.00327E-79	5.40196E-78	****	1.00E+00	1.00E+00	0.884164
1	Foot distance_Signal distance	8	A5	1286	1.01682259	0.0004863	1.00425E-58	6.02551E-58	****	1.00E+00	1.00E+00	0.884164
1	Foot distance_Signal distance	10	A5	1233	1.01680069	0.00045484	4.25965E-64	2.55579E-63	****	1.00E+00	1.00E+00	0.884164
2	Tail angle_Autocorrelation	0	CT	728	1	0.0063287	1	1	ns	1.00E+00	1	0.320196
2	Tail angle_Autocorrelation	2	CT	626	0.97293909	0.00664421	9.26049E-23	5.5563E-22	****	1.00E+00	1	0.320196
2	Tail angle_Autocorrelation	4	CT	488	0.97030063	0.0073709	6.67576E-23	4.00546E-22	****	1.00E+00	1	0.320196
2	Tail angle_Autocorrelation	6	CT	449	0.97148527	0.00813478	1.45067E-20	8.70403E-20	****	1.00E+00	1	0.320196
2	Tail angle_Autocorrelation	8	CT	450	0.95330551	0.00731066	1.51629E-79	9.09771E-79	****	1.00E+00	1	0.320196
2	Tail angle_Autocorrelation	10	CT	417	0.95285739	0.00854282	4.02882E-69	2.41729E-68	****	1.00E+00	1	0.320196
2	Tail angle_Autocorrelation	0	A5	931	1	0.00527932	1	1	ns	1.00E+00	1	0.320196
2	Tail angle_Autocorrelation	2	A5	939	1.06154048	0.00574272	9.26049E-23	5.5563E-22	****	1.00E+00	1	0.320196
2	Tail angle_Autocorrelation	4	A5	798	1.06050024	0.00538551	6.67576E-23	4.00546E-22	****	1.00E+00	1	0.320196
2	Tail angle_Autocorrelation	6	A5	993	1.05082005	0.00429118	1.45067E-20	8.70403E-20	****	1.00E+00	1	0.320196
2	Tail angle_Autocorrelation	8	A5	1313	1.11973982	0.0042107	1.51629E-79	9.09771E-79	****	1.00E+00	1	0.320196
2	Tail angle_Autocorrelation	10	A5	1231	1.10554855	0.00385684	4.02882E-69	2.41729E-68	****	1.00E+00	1	0.320196
3	Neck acc._Wavelet std_4	0	CT	730	1	0.00887594	1	1	ns	1.00E+00	1.00E+00	8.44E-01
3	Neck acc._Wavelet std_4	2	CT	623	1.0474096	0.00966708	8.80932E-14	5.28559E-13	****	1.00E+00	1.00E+00	8.44E-01
3	Neck acc._Wavelet std_4	4	CT	492	0.98932775	0.0107384	5.72811E-20	3.43687E-19	****	1.00E+00	1.00E+00	8.44E-01
3	Neck acc._Wavelet std_4	6	CT	453	0.94041074	0.01037503	1.77859E-55	1.06715E-54	****	1.00E+00	1.00E+00	8.44E-01
3	Neck acc._Wavelet std_4	8	CT	446	0.9577791	0.0107154	6.31E-59	3.78E-58	****	1.00E+00	1.00E+00	8.44E-01
3	Neck acc._Wavelet std_4	10	CT	414	0.96375573	0.01231025	1.28E-71	7.69E-71	****	1.00E+00	1.00E+00	8.44E-01
3	Neck acc._Wavelet std_4	0	A5	953	1	0.00780846	1.00E+00	1.00E+00	ns	1.00E+00	1.00E+00	8.44E-01
3	Neck acc._Wavelet std_4	2	A5	953	0.94371546	0.00917521	8.81E-14	5.29E-13	****	1.00E+00	1.00E+00	8.44E-01
3	Neck acc._Wavelet std_4	4	A5	792	0.86197114	0.00848128	5.72811E-20	3.43687E-19	****	1.00E+00	1.00E+00	8.44E-01
3	Neck acc._Wavelet std_4	6	A5	979	0.73218132	0.00718421	1.77859E-55	1.06715E-54	****	1.00E+00	1.00E+00	8.44E-01
3	Neck acc._Wavelet std_4	8	A5	1290	0.7547281	0.00605492	6.31E-59	3.78E-58	****	1.00E+00	1.00E+00	8.44E-01
3	Neck acc._Wavelet std_4	10	A5	1238	0.75282912	0.00501445	1.28E-71	7.69E-71	****	1.00E+00	1.00E+00	8.44E-01
4	Body angle velocity_ECDF percentile 0	0	CT	733	1	0.01174164	1	1	ns	1.00E+00	2.56E-04	8.95E-01
4	Body angle velocity_ECDF percentile 0	2	CT	619	0.99349508	0.01290055	0.950392836	1	ns	1.00E+00	2.56E-04	8.95E-01
4	Body angle velocity_ECDF percentile 0	4	CT	487	0.96836973	0.01366167	0.028137735	0.16882641	ns	1.00E+00	2.56E-04	8.95E-01
4	Body angle velocity_ECDF percentile 0	6	CT	448	0.93831997	0.01430956	2.67539E-09	1.60523E-08	****	1.00E+00	2.56E-04	8.95E-01
4	Body angle velocity_ECDF percentile 0	8	CT	447	0.93811006	0.01474517	1.90816E-08	1.1449E-07	****	1.00E+00	2.56E-04	8.95E-01
4	Body angle velocity_ECDF percentile 0	10	CT	424	0.9393209	0.01469519	2.68923E-09	1.61354E-08	****	1.00E+00	2.56E-04	8.95E-01
4	Body angle velocity_ECDF percentile 0	0	A5	940	1	0.00999375	1.00E+00	1.00E+00	ns	1.00E+00	2.56E-04	8.95E-01
4	Body angle velocity_ECDF percentile 0	2	A5	950	0.99455936	0.01095283	9.50E-01	1.00E+00	ns	1.00E+00	2.56E-04	8.95E-01
4	Body angle velocity_ECDF percentile 0	4	A5	790	0.92951253	0.01103418	2.81E-02	0.16882641	ns	1.00E+00	2.56E-04	8.95E-01
4	Body angle velocity_ECDF percentile 0	6	A5	987	0.83327743	0.00987281	2.68E-09	1.60523E-08	****	1.00E+00	2.56E-04	8.95E-01
4	Body angle velocity_ECDF percentile 0	8	A5	1299	0.84927352	0.00770961	1.91E-08	1.14E-07	****	1.00E+00	2.56E-04	8.95E-01
4	Body angle velocity_ECDF percentile 0	10	A5	1239	0.84042631	0.00826035	2.69E-09	1.61E-08	****	1.00E+00	2.56E-04	8.95E-01
5	Body angle_min	0	CT	739	1	0.02730972	1	1	ns	8.65E-01	1.00E+00	8.91E-01
5	Body angle_min	2	CT	622	1.03882994	0.02990078	4.39966E-06	2.6398E-05	****	8.65E-01	1.00E+00	8.91E-01
5	Body angle_min	4	CT	481	1.11350007	0.0346349	0.030615211	0.183691268	ns	8.65E-01	1.00E+00	8.91E-01
5	Body angle_min	6	CT	450	1.19404807	0.0376951	1.46313E-10	8.77877E-10	****	8.65E-01	1.00E+00	8.91E-01
5	Body angle_min	8	CT	453	1.1949386	0.03766088	5.03378E-09	3.02027E-08	****	8.65E-01	1.00E+00	8.91E-01
5	Body angle_min	10	CT	413	1.25551707	0.04209797	5.49168E-06	3.29501E-05	****	8.65E-01	1.00E+00	8.91E-01
5	Body angle_min	0	A5	942	1	0.0253374	1.00E+00	1.00E+00	ns	8.65E-01	1.00E+00	8.91E-01
5	Body angle_min	2	A5	955	1.21861548	0.02474501	4.40E-06	2.64E-05	****	8.65E-01	1.00E+00	8.91E-01
5	Body angle_min	4	A5	790	1.21117181	0.02819752	3.06E-02	1.84E-01	ns	8.65E-01	1.00E+00	8.91E-01
5	Body angle_min	6	A5	989	1.49528508	0.02638764	1.46E-10	8.78E-10	****	8.65E-01	1.00E+00	8.91E-01
5	Body angle_min	8	A5	1290	1.45509204	0.02266055	5.03E-09	3.02E-08	****	8.65E-01	1.00E+00	8.91E-01
5	Body angle_min	10	A5	1239	1.47371043	0.02379651	5.49E-06	3.30E-05	****	8.65E-01	1.00E+00	8.91E-01
6	Left limb angle asym._Histogram 1	0	CT	740	1	0.02807589	1	1	ns	1.46E-02	4.65E-30	0.774775
6	Left limb angle asym._Histogram 1	2	CT	629	1.02487049	0.0328808	0.001405871	0.008435228	**	1.46E-02	4.65E-30	0.774775
6	Left limb angle asym._Histogram 1	4	CT	490	1.12041828	0.03687113	2.48932E-22	1.49359E-21	****	1.46E-02	4.65E-30	0.774775
6	Left limb angle asym._Histogram 1	6	CT	449	0.96050556	0.03546293	1.15474E-20	6.92842E-20	****	1.46E-02	4.65E-30	0.774775
6	Left limb angle asym._Histogram 1	8	CT	457	1.15317203	0.03904622	3.73618E-53	2.24171E-52	****	1.46E-02	4.65E-30	0.774775
6	Left limb angle asym._Histogram 1	10	CT	427	1.06615263	0.03868835	4.66618E-38	2.79971E-37	****	1.46E-02	4.65E-30	0.774775
6	Left limb angle asym._Histogram 1	0	A5	936	1	0.02651958	1	1	ns	1.46E-02	4.65E-30	0.774775
6	Left limb angle asym._Histogram 1	2	A5	957	0.89343873	0.02534477	0.001405871	0.008435228	**	1.46E-02	4.65E-30	0.774775
6	Left limb angle asym._Histogram 1	4	A5	800	0.69257718	0.02516747	2.48932E-22	1.49359E-21	****	1.46E-02	4.65E-30	0.774775
6	Left limb angle asym._Histogram 1	6	A5	1003	0.6031925	0.0196513	1.15474E-20	6.92842E-20	****	1.46E-02	4.65E-30	0.774775
6	Left limb angle asym._Histogram 1	8	A5	1312	0.58004658	0.01640516	3.73618E-53	2.24171E-52	****	1.46E-02	4.65E-30	0.774775
6	Left limb angle asym._Histogram 1	10	A5	1261	0.58874219	0.01642568	4.66618E-38	2.79971E-37	****	1.46E-02	4.65E-30	0.774775
7	Right limb angle asym._Histogram 1	0	CT	742	1	0.03317324	1	1	ns	1	1.00E+00	0.598228
7	Right limb angle asym._Histogram 1	2	CT	625	1.00655937	0.03588926	0.137621953	0.825731721	ns	1	1.00E+00	0.598228
7	Right limb angle asym._Histogram 1	4	CT	497	1.021066	0.03837517	3.82707E-11	2.29624E-10	****	1	1.00E+00	0.598228
7	Right limb angle asym._Histogram 1	6	CT	453	1.01078752	0.04464375	2.43938E-19	1.46363E-18	****	1	1.00E+00	0.598228
7	Right limb angle asym._Histogram 1	8	CT	453	1.24511345	0.04674309	3.15813E-59	1.89488E-58	****	1	1.00E+00	0.598228
7	Right limb angle asym._Histogram 1	10	CT	425	1.07655899	0.04395167	3.755E-31	2.253E-30	****	1	1.00E+00	0.598228
7	Right limb angle asym._Histogram 1	0	A5	939	1	0.03045825	1	1	ns	1	1.00E+00	0.598228
7	Right limb angle asym._Histogram 1	2	A5	960	0.93766326	0.02923248	0.137621953	0.825731721	ns	1	1.00E+00	0.598228
7	Right limb angle asym._Histogram 1	4	A5	801	0.71303356	0.02751704	3.82707E-11	2.29624E-10	****	1	1.00E+00	0.598228
7	Right limb angle asym._Histogram 1	6	A5	999	0.61376192	0.02120728	2.43938E-19	1.46363E-18	****	1	1.00E+00	0.598228
7	Right limb angle asym._Histogram 1	8	A5	1320	0.58014228	0.016646	3.15813E-59	1.89488E-58	****	1	1.00E+00	0.598228
7	Right limb angle asym._Histogram 1	10	A5	1257	0.5986892	0.01815118	3.755E-31	2.253E-30	****	1	1.00E+00	0.598228
8	Left limb angle asym._Histogram 2	0	CT	742	1	0.02168668	1	1	ns	1.00E+00	1.00E+00	5.28E-01
8	Left limb angle asym._Histogram 2	2	CT	623	1.07786378	0.02532496	0.002216794	0.013300766	*	1.00E+00	1.00E+00	5.28E-01
8	Left limb angle asym._Histogram 2	4	CT	490	1.13144679	0.02974029	8.65395E-22	5.19237E-21	****	1.00E+00	1.00E+00	5.28E-01
8	Left limb angle asym._Histogram 2	6	CT	448	1.07721274	0.02873923	2.6854E-37	1.61124E-36	****	1.00E+00	1.00E+00	5.28E-01
8	Left limb angle asym._Histogram 2	8	CT	456	1.07235598	0.02851367	3.99475E-41	2.39685E-40	****	1.00E+00	1.00E+00	5.28E-01
8	Left limb angle asym._Histogram 2	10	CT	422	1.0469387	0.03083233	9.77222E-27	5.86333E-26	****	1.00E+00	1.00E+00	5.28E-01
8	Left limb angle asym._Histogram 2	0	A5	938	1	0.02105648	1	1	ns	1.00E+00	1.00E+00	5.28E-01
8	Left limb angle asym._Histogram 2	2	A5	959	0.97769291	0.0205772	0.002216794	0.013300766	*	1.00E+00	1.00E+00	5.28E-01
8	Left limb angle asym._Histogram 2	4	A5	800	0.7903888	0.02037109	8.65395E-22	5.19237E-21	****	1.00E+00	1.00E+00	5.28E-01
8	Left limb angle asym._Histogram 2	6	A5	1001	0.65278762	0.01738146	2.6854E-37	1.61124E-36	****	1.00E+00	1.00E+00	5.28E-01
8	Left limb angle asym._Histogram 2	8	A5	1318	0.65247247	0.01496114	3.99E-41	2.40E-40	****	1.00E+00	1.00E+00	5.28E-01
8	Left limb angle asym._Histogram 2	10	A5	1253	0.69138393	0.01584709	9.77E-27	5.86E-26	****	1.00E+00	1.00E+00	5.28E-01
9	Neck velocity_FFT mean coeff. 1	0	CT	736	1	0.03376391	1	1	ns	1.00E+00	4.06E-01	7.34E-01
9	Neck velocity_FFT mean coeff. 1	2	CT	620	1.06225131	0.03970176	0.006623869	0.039743213	*	1.00E+00	4.06E-01	7.34E-01
9	Neck velocity_FFT mean coeff. 1	4	CT	491	0.98209504	0.04215344	3.91203E-06	2.34722E-05	****	1.00E+00	4.06E-01	7.34E-01
9	Neck velocity_FFT mean coeff. 1	6	CT	449	0.9626069	0.04249771	0.009185566	0.055113396	ns	1.00E+00	4.06E-01	7.34E-01
9	Neck velocity_FFT mean coeff. 1	8	CT	447	1.05131447	0.04580083	0.181105255	1	ns	1.00E+00	4.06E-01	7.34E-01
9	Neck velocity_FFT mean coeff. 1	10	CT	415	1.00647523	0.04765655	3.02313E-07	1.81388E-06	****	1.00E+00	4.06E-01	7.34E-01
9	Neck velocity_FFT mean coeff. 1	0	A5	949	1	0.03204915	1.00E+00	1.00E+00	ns	1.00E+00	4.06E-01	7.34E-01
9	Neck velocity_FFT mean coeff. 1	2	A5	936	1.22240843	0.04008433	6.62E-03	3.97E-02	*	1.00E+00	4.06E-01	7.34E-01
9	Neck velocity_FFT mean coeff. 1	4	A5	790	1.27851717	0.04305883	3.91E-06	2.34722E-05	****	1.00E+00	4.06E-01	7.34E-01
9	Neck velocity_FFT mean coeff. 1	6	A5	983	1.11965618	0.03575718	9.19E-03	0.055113396	ns	1.00E+00	4.06E-01	7.34E-01
9	Neck velocity_FFT mean coeff. 1	8	A5	1302	1.12672352	0.02904117	0.181105255	1	ns	1.00E+00	4.06E-01	7.34E-01
9	Neck velocity_FFT mean coeff. 1	10	A5	1245	1.35381533	0.0356058	3.02313E-07	1.81388E-06	****	1.00E+00	4.06E-01	7.34E-01
10	Body length_FFT mean coeff. 1	0	CT	732	1	0.03352943	1	1	ns	1.00E+00	2.04E-02	7.69E-01
10	Body length_FFT mean coeff. 1	2	CT	620	0.95020333	0.03384413	0.365788319	1	ns	1.00E+00	2.04E-02	7.69E-01
10	Body length_FFT mean coeff. 1	4	CT	490	0.90229104	0.03845192	4.13904E-07	2.48342E-06	****	1.00E+00	2.04E-02	7.69E-01
10	Body length_FFT mean coeff. 1	6	CT	448	0.8690146	0.0410015	8.38754E-16	5.03253E-15	****	1.00E+00	2.04E-02	7.69E-01
10	Body length_FFT mean coeff. 1	8	CT	451	0.86282848	0.04019393	7.36182E-24	4.41709E-23	****	1.00E+00	2.04E-02	7.69E-01
10	Body length_FFT mean coeff. 1	10	CT	417	0.83750533	0.03837667	2.14201E-27	1.28521E-26	****	1.00E+00	2.04E-02	7.69E-01
10	Body length_FFT mean coeff. 1	0	A5	921	1	0.02940079	1.00E+00	1.00E+00	ns	1.00E+00	2.04E-02	7.69E-01
10	Body length_FFT mean coeff. 1	2	A5	959	0.91133772	0.02673068	3.66E-01	1.00E+00	ns	1.00E+00	2.04E-02	7.69E-01
10	Body length_FFT mean coeff. 1	4	A5	791	0.67974449	0.02484779	4.13904E-07	2.48342E-06	****	1.00E+00	2.04E-02	7.69E-01
10	Body length_FFT mean coeff. 1	6	A5	990	0.55073999	0.01864069	8.38754E-16	5.03253E-15	****	1.00E+00	2.04E-02	7.69E-01
10	Body length_FFT mean coeff. 1	8	A5	1295	0.49055743	0.01631295	7.36E-24	4.42E-23	****	1.00E+00	2.04E-02	7.69E-01
10	Body length_FFT mean coeff. 1	10	A5	1249	0.46815026	0.01448021	2.14E-27	1.29E-26	****	1.00E+00	2.04E-02	7.69E-01
11	U-body length_Negative turning points	0	CT	741	1	0.006075	1	1	ns	1.00E+00	9.63E-01	1.74E-01
11	U-body length_Negative turning points	2	CT	629	0.987775	0.006558	0.000135	0.000811	***	1.00E+00	9.63E-01	1.74E-01
11	U-body length_Negative turning points	4	CT	494	1.010186	0.007116	2.79E-09	1.68E-08	****	1.00E+00	9.63E-01	1.74E-01
11	U-body length_Negative turning points	6	CT	451	0.995584	0.007209	4.78E-42	2.87E-41	****	1.00E+00	9.63E-01	1.74E-01
11	U-body length_Negative turning points	8	CT	453	1.006387	0.007282	4.06E-54	2.44E-53	****	1.00E+00	9.63E-01	1.74E-01
11	U-body length_Negative turning points	10	CT	422	1.002038	0.007831	3.21E-42	1.93E-41	****	1.00E+00	9.63E-01	1.74E-01
11	U-body length_Negative turning points	0	A5	958	1	0.005434	1	1	ns	1.00E+00	9.63E-01	1.74E-01
11	U-body length_Negative turning points	2	A5	963	1.020922	0.005538	0.000135	0.000811	***	1.00E+00	9.63E-01	1.74E-01
11	U-body length_Negative turning points	4	A5	798	1.065464	0.005779	2.79E-09	1.68E-08	****	1.00E+00	9.63E-01	1.74E-01
11	U-body length_Negative turning points	6	A5	994	1.133608	0.00576	4.78E-42	2.87E-41	****	1.00E+00	9.63E-01	1.74E-01
11	U-body length_Negative turning points	8	A5	1304	1.154967	0.004839	4.06E-54	2.44E-53	****	1.00E+00	9.63E-01	1.74E-01
11	U-body length_Negative turning points	10	A5	1254	1.143866	0.005251	3.21E-42	1.93E-41	****	1.00E+00	9.63E-01	1.74E-01
12	Hand distance_Median diff	0	CT	728	1	0.117539	1	1	ns	1.00E+00	1.00E+00	9.70E-01
12	Hand distance_Median diff	2	CT	626	0.910835	0.119244	8.05E-05	0.000483	***	1.00E+00	1.00E+00	9.70E-01
12	Hand distance_Median diff	4	CT	482	1.232448	0.14327	4.81E-08	2.89E-07	****	1.00E+00	1.00E+00	9.70E-01
12	Hand distance_Median diff	6	CT	451	0.878286	0.146231	1.02E-10	6.14E-10	****	1.00E+00	1.00E+00	9.70E-01
12	Hand distance_Median diff	8	CT	452	1.129434	0.15207	1.41E-15	8.46E-15	****	1.00E+00	1.00E+00	9.70E-01
12	Hand distance_Median diff	10	CT	419	0.954634	0.139985	2.17E-14	1.3E-13	****	1.00E+00	1.00E+00	9.70E-01
12	Hand distance_Median diff	0	A5	958	1	0.110098	1.00E+00	1	ns	1.00E+00	1.00E+00	9.70E-01
12	Hand distance_Median diff	2	A5	963	1.687542	0.138147	8.05E-05	0.000483	***	1.00E+00	1.00E+00	9.70E-01
12	Hand distance_Median diff	4	A5	794	2.534609	0.162997	4.81E-08	2.89E-07	****	1.00E+00	1.00E+00	9.70E-01
12	Hand distance_Median diff	6	A5	984	2.46328	0.150514	1.02E-10	6.14E-10	****	1.00E+00	1.00E+00	9.70E-01
12	Hand distance_Median diff	8	A5	1279	3.47654	0.164539	1.41E-15	8.46E-15	****	1.00E+00	1.00E+00	9.70E-01
12	Hand distance_Median diff	10	A5	1227	3.190061	0.16253	2.17E-14	1.3E-13	****	1.00E+00	1.00E+00	9.70E-01
13	Leg angle asym._MFCC 3	0	CT	739	1	0.032479	1	1	ns	1.00E+00	1.00E+00	2.02E-01
13	Leg angle asym._MFCC 3	2	CT	619	1.125009	0.035868	0.001601	0.009604	**	1.00E+00	1.00E+00	2.02E-01
13	Leg angle asym._MFCC 3	4	CT	483	1.020833	0.040609	2.93E-07	1.76E-06	****	1.00E+00	1.00E+00	2.02E-01
13	Leg angle asym._MFCC 3	6	CT	444	0.875474	0.04166	2.63E-09	1.58E-08	****	1.00E+00	1.00E+00	2.02E-01
13	Leg angle asym._MFCC 3	8	CT	452	0.838696	0.042406	7.47E-08	4.48E-07	****	1.00E+00	1.00E+00	2.02E-01
13	Leg angle asym._MFCC 3	10	CT	421	0.83959	0.042569	4.5E-08	2.7E-07	****	1.00E+00	1.00E+00	2.02E-01
13	Leg angle asym._MFCC 3	0	A5	942	1	0.028724	1	1	ns	1.00E+00	1.00E+00	2.02E-01
13	Leg angle asym._MFCC 3	2	A5	948	0.98162	0.028193	0.001601	0.009604	**	1.00E+00	1.00E+00	2.02E-01
13	Leg angle asym._MFCC 3	4	A5	791	0.761251	0.030549	2.93E-07	1.76E-06	****	1.00E+00	1.00E+00	2.02E-01
13	Leg angle asym._MFCC 3	6	A5	988	0.592545	0.025527	2.63E-09	1.58E-08	****	1.00E+00	1.00E+00	2.02E-01
13	Leg angle asym._MFCC 3	8	A5	1303	0.601596	0.021256	7.47E-08	4.48E-07	****	1.00E+00	1.00E+00	2.02E-01
13	Leg angle asym._MFCC 3	10	A5	1233	0.584443	0.022907	4.50E-08	2.70E-07	****	1.00E+00	1.00E+00	2.02E-01
14	Chest acc._Wavlet entropy	0	CT	733	1	0.00016	1	1	ns	1.00E+00	1.00E+00	9.74E-01
14	Chest acc._Wavlet entropy	2	CT	620	1.000342	0.000169	7.71E-07	4.63E-06	****	1.00E+00	1.00E+00	9.74E-01
14	Chest acc._Wavlet entropy	4	CT	488	1.000398	0.000185	1.16E-11	6.98E-11	****	1.00E+00	1.00E+00	9.74E-01
14	Chest acc._Wavlet entropy	6	CT	451	1.000631	0.000181	4.44E-48	2.67E-47	****	1.00E+00	1.00E+00	9.74E-01
14	Chest acc._Wavlet entropy	8	CT	449	0.999948	0.000212	7.94E-39	4.76E-38	****	1.00E+00	1.00E+00	9.74E-01
14	Chest acc._Wavlet entropy	10	CT	417	0.999246	0.000248	1.31E-62	7.84E-62	****	1.00E+00	1.00E+00	9.74E-01
14	Chest acc._Wavlet entropy	0	A5	944	1	0.000184	1	1	ns	1.00E+00	1.00E+00	9.74E-01
14	Chest acc._Wavlet entropy	2	A5	951	0.998736	0.000237	7.71E-07	4.63E-06	****	1.00E+00	1.00E+00	9.74E-01
14	Chest acc._Wavlet entropy	4	A5	794	0.998114	0.000235	1.16E-11	6.98E-11	****	1.00E+00	1.00E+00	9.74E-01
14	Chest acc._Wavlet entropy	6	A5	999	0.993007	0.000329	4.44E-48	2.67E-47	****	1.00E+00	1.00E+00	9.74E-01
14	Chest acc._Wavlet entropy	8	A5	1300	0.993489	0.000275	7.94E-39	4.76E-38	****	1.00E+00	1.00E+00	9.74E-01
14	Chest acc._Wavlet entropy	10	A5	1217	0.987694	0.000378	1.31E-62	7.84E-62	****	1.00E+00	1.00E+00	9.74E-01
15	Right limb angle asym._Histogram 2	0	CT	746	1	0.02441	1	1	ns	1.00E+00	1.00E+00	6.90E-01
15	Right limb angle asym._Histogram 2	2	CT	629	1.085867	0.027856	0.01407	0.084421	ns	1.00E+00	1.00E+00	6.90E-01
15	Right limb angle asym._Histogram 2	4	CT	489	1.10528	0.031726	1.21E-12	7.28E-12	****	1.00E+00	1.00E+00	6.90E-01
15	Right limb angle asym._Histogram 2	6	CT	450	1.076792	0.031418	5.63E-37	3.38E-36	****	1.00E+00	1.00E+00	6.90E-01
15	Right limb angle asym._Histogram 2	8	CT	453	1.103594	0.030802	9.32E-46	5.59E-45	****	1.00E+00	1.00E+00	6.90E-01
15	Right limb angle asym._Histogram 2	10	CT	424	1.082349	0.032664	2.05E-45	1.23E-44	****	1.00E+00	1.00E+00	6.90E-01
15	Right limb angle asym._Histogram 2	0	A5	943	1	0.022431	1.00E+00	1.00E+00	ns	1.00E+00	1.00E+00	6.90E-01
15	Right limb angle asym._Histogram 2	2	A5	967	0.99825	0.022317	1.41E-02	8.44E-02	ns	1.00E+00	1.00E+00	6.90E-01
15	Right limb angle asym._Histogram 2	4	A5	803	0.832776	0.022475	1.21E-12	7.28E-12	****	1.00E+00	1.00E+00	6.90E-01
15	Right limb angle asym._Histogram 2	6	A5	1000	0.640472	0.017372	5.63E-37	3.38E-36	****	1.00E+00	1.00E+00	6.90E-01
15	Right limb angle asym._Histogram 2	8	A5	1309	0.638066	0.015408	9.32E-46	5.59E-45	****	1.00E+00	1.00E+00	6.90E-01
15	Right limb angle asym._Histogram 2	10	A5	1258	0.613384	0.015089	2.05E-45	1.23E-44	****	1.00E+00	1.00E+00	6.90E-01
16	Chest 3D velocity_LPCC 9	0	CT	734	1	0.021714	1	1	ns	1.00E+00	1.00E+00	7.63E-01
16	Chest 3D velocity_LPCC 9	2	CT	623	0.928259	0.022614	1.63E-12	9.8E-12	****	1.00E+00	1.00E+00	7.63E-01
16	Chest 3D velocity_LPCC 9	4	CT	485	0.977264	0.025629	4.06E-11	2.44E-10	****	1.00E+00	1.00E+00	7.63E-01
16	Chest 3D velocity_LPCC 9	6	CT	449	1.023722	0.027127	2.15E-18	1.29E-17	****	1.00E+00	1.00E+00	7.63E-01
16	Chest 3D velocity_LPCC 9	8	CT	446	1.053387	0.028706	5.14E-24	3.08E-23	****	1.00E+00	1.00E+00	7.63E-01
16	Chest 3D velocity_LPCC 9	10	CT	421	1.030512	0.028468	6.13E-17	3.68E-16	****	1.00E+00	1.00E+00	7.63E-01
16	Chest 3D velocity_LPCC 9	0	A5	943	1	0.01938	1.00E+00	1	ns	1.00E+00	1.00E+00	7.63E-01
16	Chest 3D velocity_LPCC 9	2	A5	953	1.155642	0.021169	1.63E-12	9.8E-12	****	1.00E+00	1.00E+00	7.63E-01
16	Chest 3D velocity_LPCC 9	4	A5	797	1.215691	0.023166	4.06E-11	2.44E-10	****	1.00E+00	1.00E+00	7.63E-01
16	Chest 3D velocity_LPCC 9	6	A5	983	1.344048	0.02103	2.15E-18	1.29E-17	****	1.00E+00	1.00E+00	7.63E-01
16	Chest 3D velocity_LPCC 9	8	A5	1286	1.417317	0.018364	5.14E-24	3.08E-23	****	1.00E+00	1.00E+00	7.63E-01
16	Chest 3D velocity_LPCC 9	10	A5	1243	1.333998	0.018537	6.13E-17	3.68E-16	****	1.00E+00	1.00E+00	7.63E-01
17	Arm angle asym._MFCC 0	0	CT	736	1	0.009946	1	1	ns	1.00E+00	9.51E-01	9.07E-01
17	Arm angle asym._MFCC 0	2	CT	626	1.024516	0.010218	0.036582	0.219493	ns	1.00E+00	9.51E-01	9.07E-01
17	Arm angle asym._MFCC 0	4	CT	488	1.011569	0.011626	0.0032	0.019198	*	1.00E+00	9.51E-01	9.07E-01
17	Arm angle asym._MFCC 0	6	CT	443	1.006793	0.012997	1.49E-09	8.96E-09	****	1.00E+00	9.51E-01	9.07E-01
17	Arm angle asym._MFCC 0	8	CT	448	1.021233	0.011826	3.14E-06	1.89E-05	****	1.00E+00	9.51E-01	9.07E-01
17	Arm angle asym._MFCC 0	10	CT	417	1.003022	0.013434	2.59E-06	1.55E-05	****	1.00E+00	9.51E-01	9.07E-01
17	Arm angle asym._MFCC 0	0	A5	943	1	0.010689	1.00E+00	1.00E+00	ns	1.00E+00	9.51E-01	9.07E-01
17	Arm angle asym._MFCC 0	2	A5	953	0.993044	0.010177	3.66E-02	2.19E-01	ns	1.00E+00	9.51E-01	9.07E-01
17	Arm angle asym._MFCC 0	4	A5	788	0.961153	0.01134	3.20E-03	1.92E-02	*	1.00E+00	9.51E-01	9.07E-01
17	Arm angle asym._MFCC 0	6	A5	989	0.898239	0.010424	1.49E-09	8.96E-09	****	1.00E+00	9.51E-01	9.07E-01
17	Arm angle asym._MFCC 0	8	A5	1298	0.938279	0.009588	3.14E-06	1.89E-05	****	1.00E+00	9.51E-01	9.07E-01
17	Arm angle asym._MFCC 0	10	A5	1234	0.916253	0.009679	2.59E-06	1.55E-05	****	1.00E+00	9.51E-01	9.07E-01
18	H-body length_Skewness	0	CT	743	1	0.095419	1	1	ns	1.00E+00	1	2.18E-01
18	H-body length_Skewness	2	CT	617	0.89791	0.111793	0.008415	0.05049	ns	1.00E+00	1	2.18E-01
18	H-body length_Skewness	4	CT	486	1.011603	0.122049	0.001964	0.011781	*	1.00E+00	1	2.18E-01
18	H-body length_Skewness	6	CT	449	0.863021	0.124652	0.777941	1	ns	1.00E+00	1	2.18E-01
18	H-body length_Skewness	8	CT	448	1.145095	0.134525	0.309427	1	ns	1.00E+00	1	2.18E-01
18	H-body length_Skewness	10	CT	415	0.867671	0.140424	1.14E-05	6.83E-05	****	1.00E+00	1	2.18E-01
18	H-body length_Skewness	0	A5	942	1	0.077317	1	1	ns	1.00E+00	1	2.18E-01
18	H-body length_Skewness	2	A5	953	1.244959	0.077232	0.008415	0.05049	ns	1.00E+00	1	2.18E-01
18	H-body length_Skewness	4	A5	786	1.449432	0.081379	0.001964	0.011781	*	1.00E+00	1	2.18E-01
18	H-body length_Skewness	6	A5	977	0.903032	0.077244	0.777941	1	ns	1.00E+00	1	2.18E-01
18	H-body length_Skewness	8	A5	1305	1.281443	0.063577	0.309427	1	ns	1.00E+00	1	2.18E-01
18	H-body length_Skewness	10	A5	1242	1.502094	0.068831	1.14E-05	6.83E-05	****	1.00E+00	1	2.18E-01
19	Hand distance_Histogram 5	0	CT	743	1	0.104291	1	1	ns	1.00E+00	0.678929	0.999235
19	Hand distance_Histogram 5	2	CT	628	1.299942	0.129338	0.110574	0.663444	ns	1.00E+00	0.678929	0.999235
19	Hand distance_Histogram 5	4	CT	496	1.255663	0.147624	0.037	0.222003	ns	1.00E+00	0.678929	0.999235
19	Hand distance_Histogram 5	6	CT	452	1.015292	0.120198	7.9E-05	0.000474	***	1.00E+00	0.678929	0.999235
19	Hand distance_Histogram 5	8	CT	457	1.608743	0.166447	0.002996	0.017975	*	1.00E+00	0.678929	0.999235
19	Hand distance_Histogram 5	10	CT	418	1.336868	0.152667	1.06E-05	6.35E-05	****	1.00E+00	0.678929	0.999235
19	Hand distance_Histogram 5	0	A5	958	1	0.087633	1	1	ns	1.00E+00	0.678929	0.999235
19	Hand distance_Histogram 5	2	A5	964	1.592019	0.121259	0.110574	0.663444	ns	1.00E+00	0.678929	0.999235
19	Hand distance_Histogram 5	4	A5	794	1.660756	0.122515	0.037	0.222003	ns	1.00E+00	0.678929	0.999235
19	Hand distance_Histogram 5	6	A5	1000	1.752362	0.112759	7.9E-05	0.000474	***	1.00E+00	0.678929	0.999235
19	Hand distance_Histogram 5	8	A5	1308	2.26891	0.117696	0.002996	0.017975	*	1.00E+00	0.678929	0.999235
19	Hand distance_Histogram 5	10	A5	1256	2.292011	0.113886	1.06E-05	6.35E-05	****	1.00E+00	0.678929	0.999235
20	Chest velocity_LPCC 5	0	CT	743	1	0.01769	1	1	ns	1.00E+00	1.00E+00	9.63E-02
20	Chest velocity_LPCC 5	2	CT	618	0.950989	0.018433	1.63E-08	9.79E-08	****	1.00E+00	1.00E+00	9.63E-02
20	Chest velocity_LPCC 5	4	CT	489	0.989179	0.021315	3.32E-13	1.99E-12	****	1.00E+00	1.00E+00	9.63E-02
20	Chest velocity_LPCC 5	6	CT	448	1.021529	0.023068	9.92E-22	5.95E-21	****	1.00E+00	1.00E+00	9.63E-02
20	Chest velocity_LPCC 5	8	CT	444	1.039295	0.02302	1.1E-19	6.59E-19	****	1.00E+00	1.00E+00	9.63E-02
20	Chest velocity_LPCC 5	10	CT	416	1.000219	0.025121	6.38E-21	3.83E-20	****	1.00E+00	1.00E+00	9.63E-02
20	Chest velocity_LPCC 5	0	A5	948	1	0.015317	1.00E+00	1	ns	1.00E+00	1.00E+00	9.63E-02
20	Chest velocity_LPCC 5	2	A5	953	1.098294	0.017138	1.63E-08	9.79E-08	****	1.00E+00	1.00E+00	9.63E-02
20	Chest velocity_LPCC 5	4	A5	782	1.207204	0.019267	3.32E-13	1.99E-12	****	1.00E+00	1.00E+00	9.63E-02
20	Chest velocity_LPCC 5	6	A5	978	1.305891	0.016707	9.92E-22	5.95E-21	****	1.00E+00	1.00E+00	9.63E-02
20	Chest velocity_LPCC 5	8	A5	1298	1.289041	0.013809	1.10E-19	6.59E-19	****	1.00E+00	1.00E+00	9.63E-02
20	Chest velocity_LPCC 5	10	A5	1246	1.270816	0.014139	6.38E-21	3.83E-20	****	1.00E+00	1.00E+00	9.63E-02

Supplementary Table 18. Statistical summary of group comparisons (A1O3) for the top 20 features (TSFEL model) at 10 wk. One-way ANOVA was performed, followed by post-hoc comparisons using Welch's t-tests comparing each group to the reference (CT) group. Statistical significance is indicated by asterisks after Holm–Sidak correction for multiple comparisons. This PDF table displays only the top 20 features in ascending rank order for concise presentation. Additional features are included in the full version of the table, available in the Source Data file (see S_Table 18). The underlying source data for this table is provided in the same Source Data file (see S_Figure 39).

Rank	Parameter	Group	n	Mean	SEM	ANOVA p-value	Raw Post-hoc p-value	Corrected Post-hoc p-value	Significance
1	Foot distance_Signal distance	CT	414	120.9610938	0.05142103	1.32E-126			
1	Foot distance_Signal distance	A5	1233	122.8990073	0.054976337	1.32E-126	9.45E-119	2.83E-118	****
1	Foot distance_Signal distance	A1	405	121.0505208	0.059591425	1.32E-126	0.256231259	0.256231259	ns
1	Foot distance_Signal distance	A1O3	71	121.2484047	0.140981552	1.32E-126	0.058739901	0.114029426	ns
2	Tail angle_Autocorrelation	CT	417	621.0410193	5.567925837	4.43E-118			
2	Tail angle_Autocorrelation	A5	1231	725.598803	2.531338775	4.43E-118	1.26E-53	3.77E-53	****
2	Tail angle_Autocorrelation	A1	408	617.4904705	5.921770687	4.43E-118	0.662361773	0.662361773	ns
2	Tail angle_Autocorrelation	A1O3	73	573.3920942	14.08472479	4.43E-118	0.002204392	0.004403924	**
3	Neck acc._Wavelet std_4	CT	414	82.6602058	1.055835852	6.90E-37			
3	Neck acc._Wavelet std_4	A5	1238	71.22936308	0.474445343	6.90E-37	2.20E-21	4.40E-21	****
3	Neck acc._Wavelet std_4	A1	409	66.48778717	0.982806596	6.90E-37	3.09E-27	9.26E-27	****
3	Neck acc._Wavelet std_4	A1O3	69	74.18110168	1.797033519	6.90E-37	8.49E-05	8.49E-05	****
4	Body angle velocity_ECDF percentile 0	CT	424	-0.664142414	0.010390164	5.44E-05			
4	Body angle velocity_ECDF percentile 0	A5	1239	-0.609791259	0.005993496	5.44E-05	6.86E-06	2.06E-05	****
4	Body angle velocity_ECDF percentile 0	A1	408	-0.642360398	0.011623455	5.44E-05	0.162750099	0.287653734	ns
4	Body angle velocity_ECDF percentile 0	A1O3	72	-0.627719176	0.023269171	5.44E-05	0.155993918	0.287653734	ns
5	Body angle_min	CT	413	6.500888549	0.217977295	6.34E-11			
5	Body angle_min	A5	1239	7.74169535	0.125007834	6.34E-11	9.87E-07	2.96E-06	****
5	Body angle_min	A1	404	6.462713521	0.205533146	6.34E-11	0.898638274	0.898638274	ns
5	Body angle_min	A1O3	69	5.596534226	0.468529268	6.34E-11	0.083178079	0.159437565	ns
6	Left limb angle asym._Histogram 1	CT	427	11.33723653	0.411403553	1.73E-63			
6	Left limb angle asym._Histogram 1	A5	1261	5.154639175	0.143812472	1.73E-63	5.43E-39	1.63E-38	****
6	Left limb angle asym._Histogram 1	A1	415	7.575903614	0.352441318	1.73E-63	7.77E-12	1.55E-11	****
6	Left limb angle asym._Histogram 1	A1O3	72	9.819444444	0.972896871	1.73E-63	0.153930463	0.153930463	ns
7	Right limb angle asym._Histogram 1	CT	425	9.249411765	0.377617115	7.03E-56			
7	Right limb angle asym._Histogram 1	A5	1257	4.161495625	0.12616908	7.03E-56	9.98E-33	2.99E-32	****
7	Right limb angle asym._Histogram 1	A1	413	5.644067797	0.270177399	7.03E-56	2.63E-14	5.26E-14	****
7	Right limb angle asym._Histogram 1	A1O3	73	6.356164384	0.605771915	7.03E-56	8.48E-05	8.48E-05	****
8	Left limb angle asym._Histogram 2	CT	422	18.1492891	0.53449628	5.24E-44			
8	Left limb angle asym._Histogram 2	A5	1253	10.56903432	0.242250967	5.24E-44	7.13E-34	2.14E-33	****
8	Left limb angle asym._Histogram 2	A1	414	12.78019324	0.475856098	5.24E-44	1.62E-13	3.24E-13	****
8	Left limb angle asym._Histogram 2	A1O3	72	15.23611111	1.136513946	5.24E-44	0.022300566	0.022300566	*
9	Neck velocity_FFT mean coeff. 1	CT	415	12.65441844	0.599186101	6.24E-32			
9	Neck velocity_FFT mean coeff. 1	A5	1245	20.32873493	0.534652572	6.24E-32	7.60E-21	2.28E-20	****
9	Neck velocity_FFT mean coeff. 1	A1	409	10.56500463	0.487137409	6.24E-32	0.006962391	0.013876307	*
9	Neck velocity_FFT mean coeff. 1	A1O3	69	12.54483707	1.376983242	6.24E-32	0.941981228	0.941981228	ns
10	Body length_FFT mean coeff. 1	CT	417	1.241565677	0.056891772	5.78E-48			
10	Body length_FFT mean coeff. 1	A5	1249	0.737821489	0.022821329	5.78E-48	1.46E-15	4.39E-15	****
10	Body length_FFT mean coeff. 1	A1	405	1.595640281	0.07316708	5.78E-48	0.000144069	0.000288117	***
10	Body length_FFT mean coeff. 1	A1O3	71	1.043731885	0.115222877	5.78E-48	0.1266215	0.1266215	ns
11	U-body length_Negative turning points	CT	422	15.86492891	0.123990241	4.02E-37			
11	U-body length_Negative turning points	A5	1254	17.87559809	0.082052092	4.02E-37	9.39E-38	2.82E-37	****
11	U-body length_Negative turning points	A1	402	17.01243781	0.131344057	4.02E-37	3.50E-10	7.01E-10	****
11	U-body length_Negative turning points	A1O3	73	16.34246575	0.294688636	4.02E-37	0.138439864	0.138439864	ns
12	Hand distance_Median diff	CT	419	0.001098357	0.00016106	1.43E-18			
12	Hand distance_Median diff	A5	1227	0.004283188	0.000218223	1.43E-18	1.42E-30	4.26E-30	****
12	Hand distance_Median diff	A1	402	0.002470199	0.000254182	1.43E-18	6.09E-06	1.22E-05	****
12	Hand distance_Median diff	A1O3	71	0.00171892	0.000525653	1.43E-18	0.262226236	0.262226236	ns
13	Leg angle asym._MFCC 3	CT	421	-46.29394915	2.347180078	6.17E-07			
13	Leg angle asym._MFCC 3	A5	1233	-32.50746682	1.274105511	6.17E-07	3.20E-07	9.61E-07	****
13	Leg angle asym._MFCC 3	A1	411	-34.74954104	2.207896049	6.17E-07	0.000360166	0.000653557	***
13	Leg angle asym._MFCC 3	A1O3	72	-27.16222923	4.594470222	6.17E-07	0.000326832	0.000653557	***
14	Chest acc._Wavlet entropy	CT	417	2.183488911	0.00054217	3.56E-76			
14	Chest acc._Wavlet entropy	A5	1217	2.157286841	0.000825998	3.56E-76	6.31E-129	1.89E-128	****
14	Chest acc._Wavlet entropy	A1	405	2.16464008	0.001143628	3.56E-76	1.09E-42	2.18E-42	****
14	Chest acc._Wavlet entropy	A1O3	72	2.18134925	0.001357257	3.56E-76	0.146499226	0.146499226	ns
15	Right limb angle asym._Histogram 2	CT	424	16.54716981	0.499381038	1.77E-60			
15	Right limb angle asym._Histogram 2	A5	1258	8.46899841	0.208330629	1.77E-60	7.43E-43	2.23E-42	****
15	Right limb angle asym._Histogram 2	A1	413	11.08474576	0.449708957	1.77E-60	1.58E-15	3.16E-15	****
15	Right limb angle asym._Histogram 2	A1O3	72	13.22222222	1.084140584	1.77E-60	0.006355385	0.006355385	**
16	Chest 3D velocity_LPCC 9	CT	421	0.194585315	0.005375514	2.13E-13			
16	Chest 3D velocity_LPCC 9	A5	1243	0.246761648	0.003428944	2.13E-13	1.11E-15	3.32E-15	****
16	Chest 3D velocity_LPCC 9	A1	401	0.228902479	0.006015333	2.13E-13	2.35E-05	4.70E-05	****
16	Chest 3D velocity_LPCC 9	A1O3	71	0.216593161	0.012385624	2.13E-13	0.106305247	0.106305247	ns
17	Arm angle asym._MFCC 0	CT	417	46.06249976	0.61693644	1.01E-24			
17	Arm angle asym._MFCC 0	A5	1234	38.23429325	0.403915516	1.01E-24	1.02E-24	3.05E-24	****
17	Arm angle asym._MFCC 0	A1	404	42.25425361	0.683829417	1.01E-24	3.92E-05	7.84E-05	****
17	Arm angle asym._MFCC 0	A1O3	71	45.77172474	1.450269276	1.01E-24	0.854007905	0.854007905	ns
18	H-body length_Skewness	CT	415	-0.179018497	0.028972309	1.97E-08			
18	H-body length_Skewness	A5	1242	-0.364531523	0.016704044	1.97E-08	4.10E-08	1.23E-07	****
18	H-body length_Skewness	A1	407	-0.242841757	0.026443056	1.97E-08	0.104104441	0.197371147	ns
18	H-body length_Skewness	A1O3	70	-0.210574464	0.073431285	1.97E-08	0.690272774	0.690272774	ns
19	Hand distance_Histogram 5	CT	418	0.85645933	0.097805237	1.04E-06			
19	Hand distance_Histogram 5	A5	1256	1.442675159	0.07168403	1.04E-06	1.57E-06	4.71E-06	****
19	Hand distance_Histogram 5	A1	412	0.86407767	0.099380013	1.04E-06	0.956440827	0.956440827	ns
19	Hand distance_Histogram 5	A1O3	71	1.070422535	0.31865155	1.04E-06	0.522687242	0.772172531	ns
20	Chest velocity_LPCC 5	CT	416	0.235399593	0.005912266	1.65E-17			
20	Chest velocity_LPCC 5	A5	1246	0.294604769	0.003277813	1.65E-17	1.54E-17	4.61E-17	****
20	Chest velocity_LPCC 5	A1	402	0.279363262	0.005664607	1.65E-17	1.03E-07	2.06E-07	****
20	Chest velocity_LPCC 5	A1O3	72	0.260991255	0.013280089	1.65E-17	0.081344886	0.081344886	ns

Supplementary Table 19. Statistical summary of group comparisons (A1O3) for the turning features at 10 wk. One-way ANOVA was performed, followed by post-hoc comparisons using Welch's t-tests comparing each group to the reference (CT) group. Statistical significance is indicated by asterisks after Benjamini–Hochberg correction for multiple comparisons. The top 20 features are listed in ascending rank order, with additional features appended afterwards. Source data are provided in the Source Data file (See S_Figure 40).

Parameter	Group	Mean	SEM	n	ANOVA p-value	Raw Post-hoc p-value	Corrected Post-hoc p-value	Significance
Turning angle	CT	7.71	0.30	170	0.1624			
Turning angle	A5	8.20	0.26	160	0.1624	0.2176	0.4480	ns
Turning angle	A1	7.31	0.49	97	0.1624	0.4890	0.4890	ns
Turning angle	A1O3	7.22	0.37	98	0.1624	0.2987	0.4480	ns
Turning duration	CT	0.25	0.01	173	0.0053			
Turning duration	A5	0.31	0.02	162	0.0053	0.0081	0.0244	*
Turning duration	A1	0.26	0.02	98	0.0053	0.7744	0.7744	ns
Turning duration	A1O3	0.23	0.02	98	0.0053	0.2873	0.4310	ns
Turning velocity	CT	40.72	2.32	170	0.0424			
Turning velocity	A5	34.37	1.74	160	0.0424	0.0291	0.0436	*
Turning velocity	A1	33.72	1.60	97	0.0424	0.0135	0.0405	*
Turning velocity	A1O3	37.51	1.81	98	0.0424	0.2757	0.2757	ns
Turning direction	CT	-0.01	0.08	174	0.1582			
Turning direction	A5	-0.06	0.08	164	0.1582	0.6503	0.9270	ns
Turning direction	A1	-0.27	0.10	99	0.1582	0.0354	0.1063	ns
Turning direction	A1O3	0.00	0.10	102	0.1582	0.9270	0.9270	ns

Supplementary Table 20. Statistical summary of group comparisons (A1O3) for the rearing features at 10 wk. One-way ANOVA was performed, followed by post-hoc comparisons using Welch's t-tests comparing each group to the reference (CT) group. Statistical significance is indicated by asterisks after Holm–Sidak correction for multiple comparisons. The top 20 features are listed in ascending rank order, with additional features appended afterwards. Source data are provided in the Source Data file (See S_Figure 41).

Parameter	Group	Mean	SEM	n	ANOVA p-value	Raw Post-hoc p-value	Corrected Post-hoc p-value	Significance
Chest z velocity (mean)_in rearing period	CT	3.02	0.23	18	0.4003			
Chest z velocity (mean)_in rearing period	A5	3.22	0.23	11	0.4003	0.5385	0.7870	ns
Chest z velocity (mean)_in rearing period	A1	3.71	0.28	6	0.4003	0.0775	0.2149	ns
Chest z velocity (mean)_in rearing period	A1O3	3.00	0.41	5	0.4003	0.9792	0.9792	ns
Chest z velocity (max)_in rearing period	CT	3.46	0.30	18	0.0724			
Chest z velocity (max)_in rearing period	A5	4.23	0.29	11	0.0724	0.0727	0.1401	ns
Chest z velocity (max)_in rearing period	A1	4.73	0.34	6	0.0724	0.0143	0.0423	*
Chest z velocity (max)_in rearing period	A1O3	3.57	0.48	5	0.0724	0.8473	0.8473	ns
Chest z velocity (min)_in rearing period	CT	16.00	0.14	18	0.0426			
Chest z velocity (min)_in rearing period	A5	1.57	0.13	11	0.0426	0.0133	0.0395	*
Chest z velocity (min)_in rearing period	A1	2.11	0.14	6	0.0426	0.8984	0.8984	ns
Chest z velocity (min)_in rearing period	A1O3	1.71	0.13	5	0.0426	0.0746	0.1437	ns
Chest z velocity (std)_in rearing period	CT	0.46	0.07	18	0.0030			
Chest z velocity (std)_in rearing period	A5	0.81	0.10	11	0.0030	0.0100	0.0199	*
Chest z velocity (std)_in rearing period	A1	0.92	0.09	6	0.0030	0.0018	0.0055	**
Chest z velocity (std)_in rearing period	A1O3	0.53	0.13	5	0.0030	0.6583	0.6583	ns
Rearing duration	CT	0.67	0.11	18	0.0071			
Rearing duration	A5	1.24	0.12	11	0.0071	0.0014	0.0042	**
Rearing duration	A1	1.03	0.14	6	0.0071	0.0694	0.1340	ns
Rearing duration	A1O3	0.68	0.21	5	0.0071	0.9566	0.9566	ns
Chest z velocity (mean)_in upward phase	CT	5.27	0.44	9	0.0506			
Chest z velocity (mean)_in upward phase	A5	3.00	0.25	2	0.0506	0.0023	0.0047	**
Chest z velocity (mean)_in upward phase	A1	7.13		1	0.0506			
Chest z velocity (mean)_in upward phase	A1O3	3.47	0.92	2	0.0506	0.2603	0.2603	ns
Chest z velocity (max)_in upward phase	CT	9.70	0.29	9	0.0002			
Chest z velocity (max)_in upward phase	A5	6.17	0.31	2	0.0002	0.0029	0.0029	**
Chest z velocity (max)_in upward phase	A1	12.39		1	0.0002			
Chest z velocity (max)_in upward phase	A1O3	8.06	0.01	2	0.0002	0.0005	0.0009	***
Chest z velocity (std)_in upward phase	CT	2.59	0.14	9	0.0498			
Chest z velocity (std)_in upward phase	A5	1.82	0.07	2	0.0498	0.0011	0.0022	**
Chest z velocity (std)_in upward phase	A1	3.17		1	0.0498			
Chest z velocity (std)_in upward phase	A1O3	2.31	0.01	2	0.0498	0.0718	0.0718	ns
Chest z velocity (mean)_in doward phase	CT	3.13	0.44	5	0.1948			
Chest z velocity (mean)_in doward phase	A5	2.33	0.24	8	0.1948	0.1589	0.4049	ns
Chest z velocity (mean)_in doward phase	A1	2.91	0.29	5	0.1948	0.6814	0.8872	ns
Chest z velocity (mean)_in doward phase	A1O3	3.34	0.04	2	0.1948	0.6642	0.8872	ns
Chest z velocity (max)_in doward phase	CT	12.11	1.00	5	0.0002			
Chest z velocity (max)_in doward phase	A5	6.90	0.47	8	0.0002	0.0036	0.0106	*
Chest z velocity (max)_in doward phase	A1	11.16	0.79	5	0.0002	0.4800	0.7296	ns
Chest z velocity (max)_in doward phase	A1O3	12.06	1.59	2	0.0002	0.9809	0.9809	ns
Chest z velocity (std)_in doward phase	CT	3.70	0.35	5	0.0002			
Chest z velocity (std)_in doward phase	A5	1.75	0.10	8	0.0002	0.0036	0.0108	*
Chest z velocity (std)_in doward phase	A1	3.25	0.42	5	0.0002	0.4400	0.6864	ns
Chest z velocity (std)_in doward phase	A1O3	3.77	0.39	2	0.0002	0.8945	0.8945	ns

Supplementary Table 21. Definitions of the Gait model top 20 features.

Feature name	Unit	Definition
Stride length	cm	Distance covered during a full sequence of a paw's gait (stance and swing phases)
Stance distance	cm	Distance covered during a stance phase of a foot
Feet distance	cm	Distance between a foot at start of a stride and the controlateral foot's strike point
Stance_chest velocity	cm/s	2D chest velocity during a stance phase
Swing velocity	cm/s	Distance covered during a swing phase of a foot devided by swing time
Stride_chest velocity	cm/s	2D chest velocity during a full sequence of a stride
Stride leg angle_min	deg	The minimum leg angle during a full sequence of a stride of the foot
Stance width	cm	Distance between feet during full stance (perpendicular distance from the controlateral foot's stance point to the line, connecting the start and end points of a stride)
Stance_leg angle anticorr.	n/a	Correlation efficiency of legs angles during a stance phase
Swing_chest velocity	cm/s	2D chest velocity during a swing phase
Stance velocity	cm/s	Stance distance devided by stance time
Swing distance	cm	Distance covered during a swing phase of a foot
Feet angle	cm	Angle between feet; foot position at the end of a stride, anus position and the controlateral foot poisition at the contrateral foot strike
Stride leg angle_max	deg	The maximum leg angle during a full sequence of a stride of the foot
Stride leg angle_std	deg	The std leg angle during a full sequence of a stride of the foot
Spatial diff._stance/swing	%	(stance_dist/stride_length)/(swing_dist/stride_length)*100
Stride leg angle_mean	deg	The mean leg angle during a full sequence of a stride of the foot
Stance leg angle_velocity	deg/s	Mean angular velocity of a leg during a stance phase
Stride leg angle_anticorr.	n/a	Correlation efficiency of legs angles during a stride phase
Swing leg angle_anticorr.	n/a	Correlation efficiency of legs angles during a swing phase

Supplementary Table 22. Statistical summary of group comparisons (A1O3) for the top 20 features (Gait model) at 10 wk. One-way ANOVA was performed, followed by post-hoc comparisons using Welch's t-tests comparing each group to the reference (CT) group. Statistical significance is indicated by asterisks after Holm–Sidak correction for multiple comparisons. This PDF table displays only the top 20 features in ascending rank order for concise presentation. Additional features are included in the full version of the table, available in the Source Data file (see S_Table 22). The underlying source data for this table is provided in the same Source Data file (see S_Figure 44).

	Parameter	Group	Mean	SEM	n	ANOVA p-value	Raw Post-hoc p-value	Corrected Post-hoc p-value	Significance
1	Stride length	CT	3.67	0.07	421	0.0000			
1	Stride length	A5	4.73	0.07	344	0.0000	0.0000	0.0000	****
1	Stride length	A1	3.59	0.10	194	0.0000	0.5100	0.5100	ns
1	Stride length	A1O3	3.52	0.10	174	0.0000	0.1989	0.3583	ns
2	Stance distance	CT	0.63	0.02	421	0.0000			
2	Stance distance	A5	1.08	0.04	348	0.0000	0.0000	0.0000	****
2	Stance distance	A1	0.83	0.04	194	0.0000	0.0000	0.0000	****
2	Stance distance	A1O3	0.74	0.03	174	0.0000	0.0106	0.0106	*
3	Feet distance	CT	2.98	0.02	421	0.0000			
3	Feet distance	A5	3.34	0.04	344	0.0000	0.0000	0.0000	****
3	Feet distance	A1	3.23	0.04	194	0.0000	0.0000	0.0000	****
3	Feet distance	A1O3	3.16	0.04	174	0.0000	0.0001	0.0001	****
4	Stance_chest velocity	CT	6.34	0.09	421	0.0000			
4	Stance_chest velocity	A5	7.37	0.05	344	0.0000	0.0000	0.0000	****
4	Stance_chest velocity	A1	7.15	0.08	194	0.0000	0.0000	0.0000	****
4	Stance_chest velocity	A1O3	6.17	0.11	174	0.0000	0.2304	0.2304	ns
5	Swing velocity	CT	13.01	0.26	421	0.0000			
5	Swing velocity	A5	15.25	0.34	344	0.0000	0.0000	0.0000	****
5	Swing velocity	A1	11.08	0.44	194	0.0000	0.0002	0.0004	***
5	Swing velocity	A1O3	12.00	0.45	174	0.0000	0.0535	0.0535	ns
6	Stride_chest velocity	CT	6.46	0.08	421	0.0000			
6	Stride_chest velocity	A5	7.45	0.05	344	0.0000	0.0000	0.0000	****
6	Stride_chest velocity	A1	7.18	0.08	194	0.0000	0.0000	0.0000	****
6	Stride_chest velocity	A1O3	6.28	0.10	174	0.0000	0.1607	0.1607	ns
7	Stride leg angle_min	CT	37.56	0.40	421	0.0177			
7	Stride leg angle_min	A5	39.61	0.71	344	0.0177	0.0121	0.0359	*
7	Stride leg angle_min	A1	39.73	0.79	194	0.0177	0.0150	0.0359	*
7	Stride leg angle_min	A1O3	39.71	0.81	174	0.0177	0.0183	0.0359	*
8	Stance width	CT	2.29	0.03	421	0.0000			
8	Stance width	A5	2.45	0.04	344	0.0000	0.0026	0.0026	**
8	Stance width	A1	2.59	0.05	194	0.0000	0.0000	0.0000	****
8	Stance width	A1O3	2.59	0.05	174	0.0000	0.0000	0.0000	****
9	Stance_leg angle anticorr.	CT	0.50	0.03	416	0.0376			
9	Stance_leg angle anticorr.	A5	0.40	0.04	338	0.0376	0.0477	0.0622	ns
9	Stance_leg angle anticorr.	A1	0.37	0.05	188	0.0376	0.0316	0.0622	ns
9	Stance_leg angle anticorr.	A1O3	0.36	0.05	173	0.0376	0.0200	0.0588	ns
10	Swing_chest velocity	CT	6.51	0.09	421	0.0000			
10	Swing_chest velocity	A5	7.48	0.05	344	0.0000	0.0000	0.0000	****
10	Swing_chest velocity	A1	7.14	0.09	194	0.0000	0.0000	0.0000	****
10	Swing_chest velocity	A1O3	6.40	0.11	174	0.0000	0.4425	0.4425	ns
11	Stance velocity	CT	2.76	0.14	420	0.0000			
11	Stance velocity	A5	3.95	0.18	339	0.0000	0.0000	0.0000	****
11	Stance velocity	A1	2.59	0.17	193	0.0000	0.4414	0.4414	ns
11	Stance velocity	A1O3	2.23	0.14	173	0.0000	0.0083	0.0166	*
12	Swing distance	CT	2.44	0.06	421	0.0000			
12	Swing distance	A5	2.87	0.07	344	0.0000	0.0000	0.0000	****
12	Swing distance	A1	2.03	0.10	194	0.0000	0.0004	0.0008	***
12	Swing distance	A1O3	2.13	0.09	174	0.0000	0.0058	0.0058	**
13	Feet angle	CT	47.39	1.10	421	0.0000			
13	Feet angle	A5	58.60	2.05	344	0.0000	0.0000	0.0000	****
13	Feet angle	A1	64.46	2.25	194	0.0000	0.0000	0.0000	****
13	Feet angle	A1O3	54.24	1.90	174	0.0000	0.0020	0.0020	**
14	Stride leg angle_max	CT	68.34	0.80	421	0.0000			
14	Stride leg angle_max	A5	83.99	1.18	344	0.0000	0.0000	0.0000	****
14	Stride leg angle_max	A1	78.07	1.36	194	0.0000	0.0000	0.0000	****
14	Stride leg angle_max	A1O3	70.90	1.38	174	0.0000	0.1121	0.1121	ns
15	Stride leg angle_std	CT	10.06	0.23	421	0.0000			
15	Stride leg angle_std	A5	14.20	0.36	344	0.0000	0.0000	0.0000	****
15	Stride leg angle_std	A1	12.08	0.41	194	0.0000	0.0000	0.0001	****
15	Stride leg angle_std	A1O3	9.88	0.40	174	0.0000	0.6980	0.6980	ns
16	Spatial diff._stance/swing	CT	46.22	3.76	420	0.0000			
16	Spatial diff._stance/swing	A5	114.86	22.51	348	0.0000	0.0028	0.0056	**
16	Spatial diff._stance/swing	A1	230.64	49.99	194	0.0000	0.0003	0.0009	***
16	Spatial diff._stance/swing	A1O3	62.87	7.87	172	0.0000	0.0574	0.0574	ns
17	Stride leg angle_mean	CT	52.63	0.53	421	0.0000			
17	Stride leg angle_mean	A5	61.41	0.83	344	0.0000	0.0000	0.0000	****
17	Stride leg angle_mean	A1	58.49	0.94	194	0.0000	0.0000	0.0000	****
17	Stride leg angle_mean	A1O3	54.87	0.94	174	0.0000	0.0390	0.0390	*
18	Stance leg angle_velocity	CT	53.50	0.59	420	0.0000			
18	Stance leg angle_velocity	A5	63.06	0.91	339	0.0000	0.0000	0.0000	****
18	Stance leg angle_velocity	A1	60.38	1.10	193	0.0000	0.0000	0.0000	****
18	Stance leg angle_velocity	A1O3	55.28	1.02	173	0.0000	0.1309	0.1309	ns
19	Stride leg angle_anticorr.	CT	0.41	0.02	421	0.1300			
19	Stride leg angle_anticorr.	A5	0.38	0.03	344	0.1300	0.3742	0.3742	ns
19	Stride leg angle_anticorr.	A1	0.31	0.04	194	0.1300	0.0257	0.0751	ns
19	Stride leg angle_anticorr.	A1O3	0.34	0.04	174	0.1300	0.1507	0.2786	ns
20	Swing leg angle_anticorr.	CT	0.67	0.03	421	0.1387			
20	Swing leg angle_anticorr.	A5	0.62	0.03	344	0.1387	0.2152	0.3841	ns
20	Swing leg angle_anticorr.	A1	0.61	0.05	194	0.1387	0.2237	0.3841	ns
20	Swing leg angle_anticorr.	A1O3	0.55	0.05	173	0.1387	0.0369	0.1067	ns

Supplementary Notes

List of Supplementary Notes:

1. Comparative evaluation of lesion types affecting the DA system
2. Model evaluation methods
3. Comparative evaluation of AI-based and conventional behavioural assessments
4. Stability and generalisability of the feature selection
5. Assessment of body size as a potential confounding factor
6. Basal activity of optoRET in A5 PD mice
7. Interpretation of a RET agonist (BT44) in the hA53T overexpression mouse PD model
8. Interpretation of L-DOPA in the hA53T overexpression mouse PD model

1. Comparative evaluation of lesion types affecting the DA system

Dopaminergic (DA) neurons are among the most vulnerable neuronal populations in the brain, partly due to their intrinsic properties, including high melanin content and the oxidative metabolism of dopamine, which contributes to elevated oxidative stress. In experimental settings – such as viral injections used in this study – DA neuron loss can be relatively high, primarily due to two distinct mechanisms: mechanical damage to the substantia nigra pars compacta (SNc) and protein overload per se, both of which are independent of the specific effects of the exogenous genes introduced.

To provide a better insight and improve the reproducibility of our study, we explicitly included and carefully compared control groups to distinguish the specific effects attributable to: mechanically induced lesion (MIL, i.e., EV group), protein overload lesion (POL, i.e., R1 or R5 groups), and bilateral alpha-Synuclein-induced lesion (AIL, i.e., A1 or A5 groups).

Comparative analysis of the Control (CT) subgroups suggested that MIL did not significantly impair the motor function nor reduce the nigral DA cell counts, showing only ~10% difference compared to the No injection (NI) group ($p = 0.6853$; Fig. 1h; Table 1). Contrarily, the POL – particularly the R5 group – showed evidence of toxicity affecting motor function in rotarod test score (RRS; $p = 0.0254$) and DA neuron integrity. Notably, the R5 group showed approximately 29% SNc DA neuronal loss ($p = 0.0325$), despite no significant decrease in the striatal TH fibre density ($p = 0.9129$), which is in line with the previous studies[1, 2] (Fig. 1g,h).

Additionally, beam walking test score (BWS) and AI-predicted PD score (APS) analyses revealed no significant differences among the subgroups within the CT animals (Fig. 1f,k). The varied outcomes the behavioural assessments suggest differing sensitivities: RRS may reflect generalised motor coordination, while BWS likely assesses fine coordination and balance[3].

Collectively, these results inform the interpretation of our PD models: the A1 group most likely reflects the effects of AIL, whilst the A5 group may present a combination of AIL and POL. As our primary objective was to distinguish PD-related (AIL) features from non-PD (NP) features, we trained the non-PD (NP) class using data from the NI, R1, and R5 groups. Accordingly, our XGB model and the derived APS (AI-predicted PD score) metric are specifically tuned to capture behavioural features associated with AIL, rather than those arising from POL.

2. Model evaluation methods

In our machine learning (ML) framework, we employed a cross-view (CV) evaluation, training models on CT (n = 10) and A5 (n = 11) mice and validating on unseen subjects from the same groups (CT, n = 4; A5, n = 6). Acknowledging that cross-subject (CS) evaluation is widely regarded as a more stringent test of generalisation, yet both methods are highly valid in the machine learning (ML) field, we repeated the ML processes to develop models with a CS method. Specifically, we included nine

additional mice (CT, n = 4; A5, n = 5) to maintain a subject-level split with approximately 70% training and 30% validation.

Notably, the compared model performances were almost the same with both validation methods (Supplementary Fig. 7a). A direct comparison of the top 20 features between the XGB-CV and XGB-CS models revealed a Spearman correlation of ≈ 0.9789 ($p \approx 7.15e-14$), indicating that the feature ranking is nearly identical with 100% of the top 20 features overlapping (Supplementary Fig. 7b). In addition, the absolute SHAP values (as measured by Pearson correlation) were nearly identical between the two protocols ($r \approx 0.9999$, $p \approx 4.81e-114$), further supporting the consistency of feature importance. Furthermore, one-way ANOVA with Holm–Sidak multiple comparisons showed no significant differences in prediction outcomes across all groups between the XGB-CV and XGB-CS models and highly correlated ($p > 0.05$; Pearson's correlation, $r = 0.99$; Supplementary Fig. 7c,d). Importantly, our original XGB-CV model consistently assessed all unseen A5 mice into the severe PD category (APS > 75% in rounded values), providing additional rationale for retaining our initial evaluation method.

Collectively, these findings robustly confirm the generalisability and stability of the CV-based XGB model. Given the close agreement in both feature ranking and predictive performance between the CV and CS evaluations, we remain confident in our model choice.

3. Comparative evaluation of AI-based and conventional behavioural assessments

We performed direct statistical comparisons between the AI approach (AI-predicted PD score [APS]) and traditional task-dependent assessments (rotarod test score [RRS] and beam walking test score [BWS]). These comparisons include: (1) sensitivity in discriminating between the two PD cohorts at the endpoint, (2) early detection of PD cohorts, and (3) correlation with histological data. The results of these comparisons are summarised in Fig. 1l.

(1) We compared the *p*-values for differences between the A1 and A5 groups at the 10 wk endpoint in RRS, BWS, and APS assessments (Fig. 1d,f,k). The *p*-values were 0.1896 (RRS), 0.0020 (BWS), and <0.0001 (APS), indicating that APS provides the most sensitive discrimination between PD severity cohorts.

(2) We assessed early-stage (2 wk) detection of the PD cohorts using RRS, BWS, and APS (Supplementary Fig. 8). At 2 wk post-surgery, RRS failed to distinguish any PD groups from EV, although the A5 group showed a trend toward significance ($p = 0.0521$). BWS significantly distinguished the A5 group from EV ($p = 0.0018$), but not the A1 group ($p = 0.0965$). In contrast, APS successfully distinguished both PD groups from EV (A1: $p = 0.0074$; A5: $p < 0.0001$) and further detected a significant difference between the A1 and A5 groups ($p = 0.0015$). These results indicate that APS enables earlier and more sensitive detection of PD compared to RRS and BWS.

(3) We evaluated the absolute Pearson correlation coefficients (|*r*|) between behavioural assessments (RRS, BWS, and APS) and histological data (Supplementary Figs. 10,11). For striatal TH intensity, |*r*| values were 0.61 (RRS), 0.74 (BWS), and 0.80 (APS). For nigral DA cell count, the values were 0.78 (RRS), 0.79 (BWS), and 0.85 (APS). Together, these results demonstrate that APS provides the strongest association with underlying PD pathology across all behavioural metrics.

4. Stability and generalisability of the feature selection

To assess the robustness of our feature selection, we compared the top 20 behavioural features in XGB model, derived from the combined train-validation (TV) dataset with those obtained from the unseen dataset (CT, n = 40; A5, n = 13).

The analysis revealed a 100% overlap in the top 20 features, with a Spearman correlation of ≈ 0.9835 ($p \approx 8.29e-15$). A separate comparison of the absolute SHAP values for these top 20 features yielded a Pearson correlation of about 0.2013 ($p \approx 0.3948$), indicating some variability in the absolute magnitudes; however, given the near-perfect consistency in ranking, this variation in magnitudes is considered of lesser concern.

Together, these results provide robust quantitative evidence that the top 20 features remain highly relevant across the TV and unseen datasets, thereby confirming the stability and generalisability of the feature selection approach.

5. Assessment of body size as a potential confounding factor

Our feature creation process yielded many distance-based behavioural features, raising potential confounds related to group-specific body size differences in PD classification by our models. To address this concern, we conducted an extensive analysis of body lengths using both the raw pose dataset and the motion filtered dataset (i.e. the primary dataset, see Motion processing in Methods). For this purpose, the body length was defined as the sum of the forebody (fbody; neck-chest distance) and hindbody (hbody; chest-anus distance) lengths in 3D, filtering for frames where the body angle was within ± 10 degree before averaging the body lengths for each mouse at each week. Please note that this measure differs from the 'body length (neck-anus distance)' presented in the manuscript (see, Fig. 2a and Supplementary Fig. 5b).

In the raw pose dataset (Supplementary Fig. 13a, left), mean body lengths at 0 wk were highly consistent across groups (CT: 6.58 ± 0.02, A1: 6.58 ± 0.04, A5: 6.6 ± 0.04 cm; n = 46, 15, 16, respectively). However, significant differences emerged at 10 wk (CT: 6.45 ± 0.04, A1: 6.76 ± 0.07, A5: 6.99 ± 0.06 cm; n = 48, 15, 18, respectively), likely due to variations in body posture during non-moving (freeze) states (accounting approximately 60% of the raw pose dataset). Crucially, analysis of the move dataset (Supplementary Fig. 13a, right) showed no significant differences in body lengths

between groups at either 0 wk (CT: 6.95 ± 0.05, A1: 6.99 ± 0.12, A5: 7.06 ± 0.05 cm; n = 45, 14, 13) or 10 wk (CT: 7.02 ± 0.06, A1: 7.28 ± 0.12, A5: 7.13 ± 0.21 cm; n = 41, 9, 11). The 2-way ANOVA tests supported these observations (Supplementary Fig. 13b): the pose dataset showed significant effects for time (p = 0.0006), group (p < 0.0001), and their interactions (p <0.0001), whereas the move dataset showed no significant effects for group (time: p = 0.0456; group: p = 0.2201; interaction: p = 0.4359).

Additionally, we re-analysed preliminary data to further assess the robustness of our APS (AI-prediction of PD scores). In this exploratory analysis, we compared data from three individual mice: R1(50), low dose RFP injection at 50 wk; A1(10), low dose A53T injection at 10 wk; and A1(50), low dose A53T injection at 50 wk. Although these analyses involved only a single mouse per condition (thus preliminary), the results were consistent with our expectations: A1(10) exhibited a smaller body length consistent with younger age, whilst R1(50) and A1(50) had similar lengths to our primary dataset (Supplementary Fig. 13c). Importantly, despite the variations in body lengths, APS measurements remained robust: R1(50) maintained APS within non-PD status, whilst both A1(10) and A1(50) exhibited APS consistent with mild PD health status (Supplementary Fig. 13d).

Collectively, these findings provide robust evidence that our distance-based features are unlikely to be confounded by body size (neck-chest-anus) differences, particularly within the move dataset used for our primary analysis. Although the raw pose dataset exhibited differences (likely related to postural variations in the non-moving periods), our core analyses on the move dataset did not reflect significant confounds related to body size.

6. Basal activity of optoRET in A5 PD mice

Statistically, optoRET activation was ineffective in the A5 mice (see Supplementary Table 8), although a subtle, non-significant trend was observed in the A5OD (A5+optoRET+dark) and A5O2 (A5+optoRET+S#1) groups. Our *in vitro* studies suggest that the effects of optoRET are non-linear, with optimal light conditions yielding the most beneficial effects[4]. Although the light-sensitive domain of optoRET has been engineered to minimise basal activity, some residual effect remains. In severe PD states (A5), this basal activity may confer a modest benefit compared to daily, full light activation (A5O1), whereas in milder PD stages the effect appears negligible. We postulate that the basal effect may have protected A5 mice from the high protein overload lesion (POL) rather than the alpha-Synuclein-induced lesion (AIL; see Fig.1 and Supplementary Note 1). Further studies are required to elucidate these state-dependent dynamics; for instance, future experiments could investigate whether overexpression of R5 with optoRET can prevent motor dysfunction caused by POL.

Given that these trends did not reach statistical significance, our detailed analyses focused on the condition exhibiting the most robust response (A1O3).

7. Interpretation of a RET agonist (BT44) in the hA53T overexpression mouse PD model

Although optogenetic activation of RET signalling using optoRET provides valuable proof-of-concept and mechanistic insights, its direct clinical translation remains challenging (despite the FDA approval of channelrhodopsin and the clinical use of deep-brain implanted devices). Alternative RET-targeted therapeutics, such as pharmacologically validated RET agonists, may offer more immediate translational promise. However, currently available RET agonists lack the capacity for temporal control and face substantial limitations regarding pharmacokinetics[5-7], including poor systemic delivery and potential off-target effects – particularly in tissues with high RET expression, such as the gastrointestinal tract. One of the most extensively validated preclinical RET agonists, BT44, has thus far only been shown to be effective in a rat 6-OHDA model when delivered directly to the brain via infusion[6].

To test the efficacy of a RET agonist in our hA53T overexpression mouse PD model, we included an experimental group, A1BT, in which BT44 (120 μM) was infused bilaterally into the brains of A1 mice at a constant flow rate of 0.18 μL/hr using osmotic pumps (see Methods). Due to the technical limitations of the pumps' capacity, the experiment concluded at 6 wk post-virus injection. Despite these constraints, we evaluated the therapeutic efficacy of BT44 relative to the optoRET group (A1O3).

Our findings indicated that BT44 was overall ineffective in the A1 PD model (Supplementary Figs. 26, 31). An initial beneficial trend was observed in the elevated beam walking test score (BWS) and AI-predicted PD score (APS), albeit smaller in magnitude than in the A1O2 group (Supplementary Fig. 25f). Additionally, the rotarod test score (RRS) showed improvement, although this did not reach statistical significance compared to the A1 group (Supplementary Fig. 26d). These results suggest that BT44 may moderately improve general motor coordination (RRS) but fails to significantly enhance fine motor control or balance (BWS) or broader PD-related features (APS).

The early beneficial trend could reflect the agonist's dependency on endogenous RET receptors, which are known to be downregulated during PD progression – particularly in aSyn overexpression models[8-10]. We propose that the limited efficacy results from a combination of factors: (1) a reduction in endogenous RET expression in our model, where aSyn overexpression likely exceeds the levels typically seen in human PD pathology[10]; and (2) diminished neuroprotective efficacy following prolonged ligand exposure[11, 12]. To distinguish whether these effects arise primarily from receptor downregulation or prolonged stimulation, further studies are warranted using alternative aSyn models, such as the aSyn-preformed fibril model, in which GDNF/RET signalling has shown therapeutic benefit[13].

Finally, treatment response evaluation (TRE) for the A1BT group indicated that only approximately 10% of behavioural features were marked as treated (Supplementary Fig. 31), further supporting the limited efficacy of BT44 in this context. A comparative TRE analysis across optoRET (A1O2, A1O3) and RET agonist (A1BT) groups also revealed common off-target effects on

locomotion, potentially reflecting RET signalling activation in brain circuits that influence motor behaviour. Collectively, these results highlight the comparative efficacy of optoRET over pharmacological RET agonists and offer mechanistic insights that may help inform future RET-targeted therapeutic strategies for PD.

8. Interpretation of L-DOPA in the hA53T overexpression mouse PD model

Levodopa (L-DOPA; 3,4-Dihydroxy-L-phenylalanine), a dopamine precursor, remains the gold-standard symptomatic treatment for PD, particularly effective in alleviating akinesia and rigidity[14-16]. However, L-DOPA is less effective for certain axial and non-motor symptoms, and its clinical utility becomes increasingly limited as PD progresses, primarily due to the progressive loss of dopaminergic neurons, which narrows the therapeutic window[14]. Over time, the therapeutic benefits of L-DOPA often diminish ('wearing-off' between medications), whilst side effects such as L-DOPA-induced dyskinesia (LID) emerge at peak concentrations[14, 16]. There is general agreement that LID arises from pronounced, intermittent fluctuations in brain DA levels, leading to abnormal stimulation of striatal dopamine receptors[16].

We evaluated L-DOPA in our hA53T overexpression mouse PD model if the intermittent administrations of a high dose (50 mg/kg) could improve PD symptoms. We included two L-DOPA treatment cohorts of A1 mice (A1L1 and A1L3 groups), in which L-DOPA (50 mg/kg) was administered intraperitoneally either daily or on alternate days, respectively. In our hA53T overexpression PD mouse model, we observed no significant therapeutic improvements across all assessed metrics, including RRS, BWT, and APS (Supplementary Fig. 26). Notably, the rotarod test score (RRS) showed an early trend towards worsening, although this effect was not significant at the endpoint. Furthermore, a significant increase in neck/trunk dystonia was observed, measured by the axial bending angle[16] (Supplementary Fig. 26j–m). These findings may reflect the consequences of our high-dose intermittent dosing protocols, potentially exacerbating side effects through dramatic fluctuations in striatal dopamine levels. Finally, treatment response evaluation (TRE) further revealed notable off-target effects on overall motor coordination (Supplementary Fig. 31).

Collectively, these results may highlight the importance of selecting appropriate medication protocols based on therapeutic strategy – continuous delivery may be more suitable for L-DOPA[17], whereas intermittent activation appears more appropriate for c-RET stimulation[11, 12] via optoRET.

Supplementary References

1. Koprich, J.B., Johnston, T.H., Reyes, M.G., Sun, X. & Brotchie, J.M. Expression of human A53T alpha-synuclein in the rat substantia nigra using a novel AAV1/2 vector produces a rapidly evolving pathology with protein aggregation, dystrophic neurite architecture and nigrostriatal degeneration with potential to model the pat. *Molecular Neurodegeneration* **5**, 43 (2010).
2. Sanchez-Guajardo, V., Febbraro, F., Kirik, D. & Romero-Ramos, M. Microglia acquire distinct activation profiles depending on the degree of alpha-synuclein neuropathology in a rAAV based model of Parkinson's disease. *PLoS One* **5**, e8784 (2010).
3. Aniszewska, A., Bergstrom, J., Ingelsson, M. & Ekmark-Lewen, S. Modeling Parkinson's disease-related symptoms in alpha-synuclein overexpressing mice. *Brain Behav* **12**, e2628 (2022).
4. Hyeon, B., Lee, H., Kim, N. & Heo, W.D. Optogenetic dissection of RET signaling reveals robust activation of ERK and enhanced filopodia-like protrusions of regenerating axons. *Mol Brain* **16**, 56 (2023).
5. Viisanen, H. et al. Novel RET agonist for the treatment of experimental neuropathies. *Mol Pain* **16**, 1744806920950866 (2020).
6. Renko, J.M. et al. Neuroprotective Potential of a Small Molecule RET Agonist in Cultured Dopamine Neurons and Hemiparkinsonian Rats. *J Parkinsons Dis* **11**, 1023-1046 (2021).
7. Haider, M.S. et al. Biological Activity In Vitro, Absorption, BBB Penetration, and Tolerability of Nanoformulation of BT44:RET Agonist with Disease-Modifying Potential for the Treatment of Neurodegeneration. *Biomacromolecules* **24**, 4348-4365 (2023).
8. Decressac, M. et al. alpha-Synuclein-induced down-regulation of Nurr1 disrupts GDNF signaling in nigral dopamine neurons. *Sci Transl Med* **4**, 163ra156 (2012).
9. Decressac, M., Volakakis, N., Bjorklund, A. & Perlmann, T. NURR1 in Parkinson disease--from pathogenesis to therapeutic potential. *Nat Rev Neurol* **9**, 629-636 (2013).
10. Conway, J.A. & Kramer, E.R. Is activation of GDNF/RET signaling the answer for successful treatment of Parkinson's disease? A discussion of data from the culture dish to the clinic. *Neural Regeneration Research* **17**, 1462-1467 (2022).
11. Mesa-Infante, V., Afonso-Oramas, D., Salas-Hernandez, J., Rodriguez-Nunez, J. & Barroso-Chinea, P. Long-term exposure to GDNF induces dephosphorylation of Ret, AKT, and ERK1/2, and is ineffective at protecting midbrain dopaminergic neurons in cellular models of Parkinson's disease. *Mol Cell Neurosci* **118**, 103684 (2022).
12. Azevedo, M.D. et al. Oxidative stress induced by sustained supraphysiological intrastriatal GDNF delivery is prevented by dose regulation. *Molecular Therapy Methods & Clinical Development* **31** (2023).
13. Chmielarz, P. et al. GDNF/RET Signaling Pathway Activation Eliminates Lewy Body Pathology in Midbrain Dopamine Neurons. *Mov Disord* **35**, 2279-2289 (2020).
14. Armstrong, M.J. & Okun, M.S. Diagnosis and Treatment of Parkinson Disease: A Review. *JAMA* **323**, 548-560 (2020).
15. Blosser, J.A., Podolsky, E. & Lee, D. L-DOPA-Induced Dyskinesia in a Genetic Drosophila Model of Parkinson's Disease. *Exp Neurobiol* **29**, 273-284 (2020).
16. Andreoli, L., Abbaszadeh, M., Cao, X. & Cenci, M.A. Distinct patterns of dyskinetic and dystonic features following D1 or D2 receptor stimulation in a mouse model of parkinsonism. *Neurobiol Dis* **157**, 105429 (2021).
17. Olanow, C.W., Calabresi, P. & Obeso, J.A. Continuous Dopaminergic Stimulation as a Treatment for Parkinson's Disease: Current Status and Future Opportunities. *Mov Disord* **35**, 1731-1744 (2020).
