# Supplementary material for: Integrating artificial intelligence and optogenetics for Parkinson’s disease diagnosis and therapeutics in male mice
Source: Nat Commun. 2025 Aug 21;16:7797. doi: 10.1038/s41467-025-63025-w (PMC12370958; doi:10.1038/s41467-025-63025-w)
Supplement: Supplementary file 2 — Description of Additional Supplementary Files [file 41467_2025_63025_MOESM2_ESM.pdf]

Description of Additional Supplementary Files

Supplementary Movie 1
This video shows representative forelimb tremor observed in A53T Parkinson’s disease model mice.

Supplementary Movie 2
This video shows representative ataxic rearing behaviour observed in A53T Parkinson’s disease model mice.

Supplementary Movie 3
This video shows representative hindlimb trailing behaviour observed in A53T Parkinson’s disease model mice.

Supplementary Movie 4
This video shows representative performance in the elevated beam walking test observed in control and A53T Parkinson’s disease model mice.

Supplementary Movie 5
This video shows representative hindlimb clasping behaviour during the tail suspension test observed in A53T Parkinson’s disease model mice.

Supplementary Movie 6
This video shows representative walking behaviour observed in control mice, A53T Parkinson’s disease model mice, and A53T mice with optogenetic stimulation.
