# Supplementary material for: Integrating artificial intelligence and optogenetics for Parkinson’s disease diagnosis and therapeutics in male mice
Source: Nat Commun. 2025 Aug 21;16:7797. doi: 10.1038/s41467-025-63025-w (PMC12370958; doi:10.1038/s41467-025-63025-w)
Supplement: Supplementary file 9 — Reporting Summary [file 41467_2025_63025_MOESM9_ESM.pdf]

nature portfolio

Corresponding author(s): C. Justin Lee, Daesoo Kim, Won Do Heo

Last updated by author(s): Jul 27, 2025

Reporting Summary

Nature Portfolio wishes to improve the reproducibility of the work that we publish. This form provides structure for consistency and transparency in reporting. For further information on Nature Portfolio policies, see our Editorial Policies and the Editorial Policy Checklist.

Statistics

For all statistical analyses, confirm that the following items are present in the figure legend, table legend, main text, or Methods section.

n/a	Confirmed	
☐	☒	The exact sample size (*n*) for each experimental group/condition, given as a discrete number and unit of measurement
☐	☒	A statement on whether measurements were taken from distinct samples or whether the same sample was measured repeatedly
☐	☒	The statistical test(s) used AND whether they are one- or two-sided<br>*Only common tests should be described solely by name; describe more complex techniques in the Methods section.*
☐	☒	A description of all covariates tested
☐	☒	A description of any assumptions or corrections, such as tests of normality and adjustment for multiple comparisons
☐	☒	A full description of the statistical parameters including central tendency (e.g. means) or other basic estimates (e.g. regression coefficient) AND variation (e.g. standard deviation) or associated estimates of uncertainty (e.g. confidence intervals)
☐	☒	For null hypothesis testing, the test statistic (e.g. *F*, *t*, *r*) with confidence intervals, effect sizes, degrees of freedom and *P* value noted<br>*Give P values as exact values whenever suitable.*
☒	☐	For Bayesian analysis, information on the choice of priors and Markov chain Monte Carlo settings
☐	☒	For hierarchical and complex designs, identification of the appropriate level for tests and full reporting of outcomes
☐	☒	Estimates of effect sizes (e.g. Cohen's *d*, Pearson's *r*), indicating how they were calculated

Our web collection on statistics for biologists contains articles on many of the points above.

Software and code

Policy information about availability of computer code

Data collection	AVATARnet(demo)
Data analysis	AVATARpy(v0.1.9), TSFEL(0.1.3), PyCaret(v2.3.10), SHAP(v0.41.0), Python(v3.8), Prism(v10.5)

For manuscripts utilizing custom algorithms or software that are central to the research but not yet described in published literature, software must be made available to editors and reviewers. We strongly encourage code deposition in a community repository (e.g. GitHub). See the Nature Portfolio guidelines for submitting code & software for further information.

Data

Policy information about availability of data

All manuscripts must include a data availability statement. This statement should provide the following information, where applicable:

- Accession codes, unique identifiers, or web links for publicly available datasets
- A description of any restrictions on data availability
- For clinical datasets or third party data, please ensure that the statement adheres to our policy

The datasets generated and analysed during this study have been deposited in Figshare and are publicly available at: [DOI to be provided in the next revision]69. The archive includes the processed, feature-engineered datasets used for machine learning and analysis. Due to data size constraints, the full raw video recordings and complete 3D pose datasets are not included. However, a representative subset of the 3D reconstructed pose data, along with a sample multi-view video, has been made available for demonstration purposes and to support reproducibility. Data supporting the findings of this study are included in the main manuscript and Supplementary Information. A high-resolution version of the Supplementary Figures file is available in the same archive. Source data are provided with this paper as

a Source Data file, including the full version of the Supplementary Tables.

Research involving human participants, their data, or biological material

Policy information about studies with human participants or human data. See also policy information about sex, gender (identity/presentation), and sexual orientation and race, ethnicity and racism.

Reporting on sex and gender	*Use the terms sex (biological attribute) and gender (shaped by social and cultural circumstances) carefully in order to avoid confusing both terms. Indicate if findings apply to only one sex or gender; describe whether sex and gender were considered in study design; whether sex and/or gender was determined based on self-reporting or assigned and methods used. Provide in the source data disaggregated sex and gender data, where this information has been collected, and if consent has been obtained for sharing of individual-level data; provide overall numbers in this Reporting Summary. Please state if this information has not been collected. Report sex- and gender-based analyses where performed, justify reasons for lack of sex- and gender-based analysis.*
Reporting on race, ethnicity, or other socially relevant groupings	*Please specify the socially constructed or socially relevant categorization variable(s) used in your manuscript and explain why they were used. Please note that such variables should not be used as proxies for other socially constructed/relevant variables (for example, race or ethnicity should not be used as a proxy for socioeconomic status). Provide clear definitions of the relevant terms used, how they were provided (by the participants/respondents, the researchers, or third parties), and the method(s) used to classify people into the different categories (e.g. self-report, census or administrative data, social media data, etc.) Please provide details about how you controlled for confounding variables in your analyses.*
Population characteristics	*Describe the covariate-relevant population characteristics of the human research participants (e.g. age, genotypic information, past and current diagnosis and treatment categories). If you filled out the behavioural & social sciences study design questions and have nothing to add here, write "See above."*
Recruitment	*Describe how participants were recruited. Outline any potential self-selection bias or other biases that may be present and how these are likely to impact results.*
Ethics oversight	*Identify the organization(s) that approved the study protocol.*

Note that full information on the approval of the study protocol must also be provided in the manuscript.

Field-specific reporting

Please select the one below that is the best fit for your research. If you are not sure, read the appropriate sections before making your selection.

[x] Life sciences [ ] Behavioural & social sciences [ ] Ecological, evolutionary & environmental sciences

For a reference copy of the document with all sections, see nature.com/documents/nr-reporting-summary-flat.pdf

Life sciences study design

All studies must disclose on these points even when the disclosure is negative.

Sample size	No statistical method was used to pre-determine sample size. Instead, sample sizes were selected based on the number of animals necessary to enable robust statistical analysis and detect biologically meaningful effects.
Data exclusions	Data cleaning was conducted using two pre-defined outlier detection methods to ensure analytical integrity: (1) quantile-based trimming (lower quantile = 0.01, upper quantile = 0.99) in Python, and (2) the ROUT method (Q = 5%) in GraphPad Prism (v10.5). These were incorporated as part of the analysis pipeline to minimise the influence of extreme data points.
Replication	All experiments were conducted with an average of two independent biological replicates. Some supplementary groups were not independently replicated (see Supplementary Table 1). Each experiment included appropriate control and treatment groups.
Randomization	Mice were allocated to groups based on injection conditions, with an effort to balance experimental variables across groups. Mice used for model training and those retained for validation were randomly selected.
Blinding	Investigators were not blinded to group allocation due to procedural constraints, including the need for appropriate handling and post-experimental care that differed between groups. Efforts were made to minimise potential bias through standardised protocols.

Reporting for specific materials, systems and methods

We require information from authors about some types of materials, experimental systems and methods used in many studies. Here, indicate whether each material, system or method listed is relevant to your study. If you are not sure if a list item applies to your research, read the appropriate section before selecting a response.

Materials & experimental systems

n/a	Involved in the study	
[ ]	[x]	Antibodies
[x]	[ ]	Eukaryotic cell lines
[x]	[ ]	Palaeontology and archaeology
[ ]	[x]	Animals and other organisms
[x]	[ ]	Clinical data
[x]	[ ]	Dual use research of concern
[x]	[ ]	Plants

Methods

n/a	Involved in the study	
[x]	[ ]	ChIP-seq
[x]	[ ]	Flow cytometry
[x]	[ ]	MRI-based neuroimaging

Antibodies

Antibodies used	All primary are diluted as 1:1000. Sheep anti-TH antibody (Abcam, Cat# ab113), rabbit anti-aSyn (Abcam, Cat# ab52168), or rabbit anti-phospho(S129)-aSyn (Abcam, Cat# ab51253).
Validation	All antibodies used in this study were pre-validated by their manufacturers.<br>https://www.abcam.com/en-us/products/primary-antibodies/tyrosine-hydroxylase-antibody-neuronal-marker-ab113<br>https://www.abcam.com/en-us/products/primary-antibodies/alpha-synuclein-antibody-ab52168<br>https://www.abcam.com/en-us/products/primary-antibodies/alpha-synuclein-phospho-s129-antibody-ep1536y-ab51253

Animals and other research organisms

Policy information about studies involving animals; ARRIVE guidelines recommended for reporting animal research, and Sex and Gender in Research

Laboratory animals	The study involved laboratory mice. Three distinct lines on the C57BL/6J background were used: wild-type (WT), DAT-CRE, and SOD1-G93A mice, all obtained from The Jackson Laboratory (strains #006302 and #002726). DAT-CRE and SOD1-G93A mice were maintained as B6.SJL × C57BL/6J hybrids. Mice were aged 8–10 weeks at the time of surgery or experimental onset. Male mice were used in most groups, except for the motor neuron disease (MND) cohorts, where female WT and SOD1-G93A mice were used for the NALS and ALS groups, respectively.
Wild animals	The study did not involve wild animals.
Reporting on sex	Sex was considered in the study design. Male mice were used in all groups except for the motor neuron disease (MND) cohorts, which included female wild-type (NALS group) and female SOD1-G93A (ALS group) mice. No sex-based statistical comparisons were performed, as sex was not a variable of primary interest in this study. Detailed information regarding mouse line, sex, and group assignment is provided in Supplementary Table 1.
Field-collected samples	The study did not involve samples collected from the field.
Ethics oversight	Animal experiments and treatment followed the guidelines of the Institutional Animal Care and Use Committees (IACUC) at KAIST.

Note that full information on the approval of the study protocol must also be provided in the manuscript.

Plants

Seed stocks	*Report on the source of all seed stocks or other plant material used. If applicable, state the seed stock centre and catalogue number. If plant specimens were collected from the field, describe the collection location, date and sampling procedures.*
Novel plant genotypes	*Describe the methods by which all novel plant genotypes were produced. This includes those generated by transgenic approaches, gene editing, chemical/radiation-based mutagenesis and hybridization. For transgenic lines, describe the transformation method, the number of independent lines analyzed and the generation upon which experiments were performed. For gene-edited lines, describe the editor used, the endogenous sequence targeted for editing, the targeting guide RNA sequence (if applicable) and how the editor was applied.*
Authentication	*Describe any authentication procedures for each seed stock used or novel genotype generated. Describe any experiments used to assess the effect of a mutation and, where applicable, how potential secondary effects (e.g. second site T-DNA insertions, mosiacism, off-target gene editing) were examined.*
